# From Single Injection to Dual Insights: Integrating EAD and CID Acquisition for Spectral Library Generation and EAD–CID Spectral Matching

**DOI:** 10.1021/acs.analchem.6c01204

**Published:** 2026-10-02

**Authors:** Lisa Panzenboeck, Christina Brenner, Veronika Fitz, Mingxun Wang, Patrick Mueller, Evelyn Rampler, Yasin El Abiead, Gunda Koellensperger

**Affiliations:** † Department of Analytical Chemistry, Faculty of Chemistry, 31285 University of Vienna, 1090 Vienna, Austria; ‡ Vienna Doctoral School in Chemistry (DoSChem), University of Vienna, 1090 Vienna, Austria; § Department of Computer Science and Engineering, 8790 University of California Riverside, Riverside, California 92521, United States; ∥ BOKU University, Department of Chemistry, Institute of Analytical Chemistry, Muthgasse 18, 1190 Vienna, Austria; ⊥ Vienna Metabolomics Center (VIME), University of Vienna, 1090 Vienna, Austria

## Abstract

Mass spectral MS/MS libraries are essential in nontargeted metabolomics as they facilitate metabolite annotation and provide the basis for in silico annotation tools and fragmentation rules. While significant progress has been made toward more comprehensive MS/MS libraries, those efforts have focused mainly on collision-induced dissociation (CID)- and higher-energy collisional dissociation (HCD)-based approaches. In this work, we introduce a flow injection-high-resolution targeted MS/MS-based acquisition strategy that acquires electron-activated dissociation (EAD) and CID across a wide range of fragmentation energies within a single injection. The workflow supports both nontargeted and targeted analysis, through efficient library generation and MS/MS parameter optimization, respectively. We apply the workflow via a set of 45 steroid standards, known for their important role in human health, disease, and doping, expanding the coverage of public libraries for both CID and EAD. Finally, we utilize public MS/MS libraries and raw data to reveal that EAD MS/MS spectra can, to some extent, be annotated via existing MS/MS libraries, governed by CID and HCD, and show that the acquired CID library enables novel annotations in publicly available clinical data, demonstrating its utility for biological studies.

## Graphical abstract

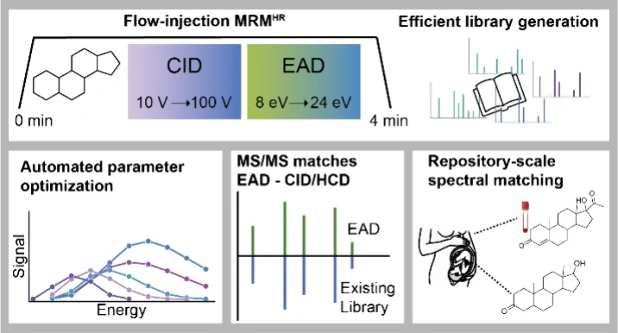

## Introduction

The identification of metabolites from nontargeted metabolomics experiments is almost entirely dependent on mass spectrometric fragmentation libraries. Public MS/MS libraries have aggregated MS/MS spectra for more than 200,000 molecules, enabling the annotation of these compounds across diverse sample types.^1,2^ The collection of these spectra, which took more than ten years, resulted in annotation rates more than tripling over that time.^3^ However, while the overall annotation rate has increased, the identification of structural isomers has remained challenging, as the differentiation of similar molecules is often not enabled by conventional fragmentation techniques such as collision induced dissociation (CID) and higher-energy collisional dissociation (HCD).^4,5^

For this reason, radical-driven fragmentation techniques have recently gained prominence. One such technique is electron-activated dissociation (EAD), which uses electrons with tunable kinetic energy to cover a wide range of fragmentation mechanisms.^4,6–8^ At electron energies ≤ 5 eV, electron-capture dissociation (ECD), and ≤ 10 eV, hot ECD (hECD), of multiply charged ions dominate, forming charge-reduced radical cations that subsequently fragment. For singly charged analytes, energies above 10 eV are required to induce excitation of organic ions (EIEIO), leading to dissociation from excited ions or intermediate doubly charged radical cations. EAD improves structural elucidation for a wide range of molecules, including steroids,^9,10^ drugs and metabolites,^11^ pesticides,^12^ lipids,^13–17^ acylcarnitines,^18^ and bile acids,^19^ enabling the discrimination of stereoisomers and positional isomers, as well as the rational localization of double-bond and methyl-branching positions. In recent years, technical advancements have improved the fragmentation efficiency of singly charged ions^6,7,20^ and increased instrument sensitivity^18,21,22^ enabling detailed structural elucidation at low compound concentrations^12^ and in complex biological samples, including mouse serum and tissue,^9^ human plasma,^18,19,23^ and single cells.^24^

To date, its applicability has mainly been demonstrated for compounds with repetitive structural patterns such as lipids, for which rule-based approaches and/or in silico tools can be used to decipher fragment information.^17,23,25^

However, implementing new fragmentation techniques, such as EAD, for metabolomics comes with trade-offs. Most importantly, traditional metabolomics annotation workflows, which rely on spectral matching to existing CID/HCD libraries, may not be applicable if MS/MS spectra of the same molecule differ too strongly between techniques. The unavailability of this routine annotation concept would drastically reduce the practical utility of structural informative and fragmentation-rich EAD-based nontargeted metabolomics experiments. While recently some efforts have been made to increase the number of EAD library MS/MS spectra in the public domain (e.g., MS-DIAL 5,^17^ MultiMS2 ^200,26^ and CIeaD^27^), the number of research groups contributing spectra of non-CID/HCD fragmentation techniques to public MS/MS libraries is still low.

Here, we present a flow injection (FI) – high-resolution targeted MS/MS (high-resolution multiple reaction monitoring (MRM^HR^)) method that enables the quasi-simultaneous creation of comprehensive EAD- and CID-MS/MS libraries from analytical standards across a wide range of fragmentation parameters. Employing high-resolution targeted MS/MS, we ensure that precursors of interest are fragmented under user-defined parameters, resulting in comprehensive MS/MS spectra acquired at high resolution. The workflow provides a high degree of spectral purity by minimizing carryover, which is especially important for compound classes characterized by a high number of structurally similar molecules. With 150–1500 high-quality MS/MS scans per injection and compound, the proposed strategy can be exploited to cover a variety of adducts and fragmentation energies within a single run. We showcase the developed workflow by acquiring fragment spectra of more than 40 steroid standards, six of which have previously not been available in public MS/MS libraries (MoNA,^28^ MassBankEU,^29^ or the GNPS library^30^) in any fragmentation mode. When matched against existing spectral libraries, comparable annotation rates were found for the generated CID and EAD spectra, suggesting the utility of existing public MS/MS libraries for EAD spectra in the case of steroids. To demonstrate the applicability of the generated MS/MS spectra for biological samples, we show that putative assignments of MS/MS spectra in publicly available raw data reveal biologically plausible patterns, including the identification of 17-α-hydroxyprogesterone in the blood serum of pregnant women and 5-α-androstan-17-β-ol-3-one in human placenta – annotations that were not found using previously existing public spectral libraries. The array of fragmentation energies accessible via the proposed methodology makes it applicable not only for spectral library generation, but also for optimizing high-resolution targeted MS/MS-fragmentation parameters required for targeted metabolomics experiments to achieve high sensitivity. We provide a processing pipeline that supports the selection of optimal fragmentation parameters based on breakdown curves generated from the MS/MS library data set. The FI-high-resolution targeted MS/MS workflow consequently increases efficiency and automation compared to current parameter optimization workflows, often based on liquid chromatography (LC) or direct infusion via syringe pumps. Finally, we have adapted the GNPSDashboard^31^ to support files acquired using this workflow, enabling collaborative sharing and interactive plotting of raw data.

## Experimental Section

### Solvents and Standards

Methanol (MeOH) and acetonitrile (ACN) were of LC-MS grade and purchased from Honeywell. Water was purified on site with an ELGA Water purification system (18.2 MΩ cm, Purelab Ultra MK2). Formic acid (FA), used as sample- and eluent-additive, was bought at VWR. A comprehensive overview of steroid standards and respective vendors is provided in the Supporting Information Table (SA1) (SMILES, InChI, and InChIKey entries were retrieved from PubChem^32^ based on the vendor-provided CAS). All standards were dissolved in MeOH, diluted with MeOH + 0.1% FA to reach a final concentration of 5 μM, and stored at −20 °C prior to analysis.

### FI-MS Setup

A 1260 Bio Flexible quaternary pump from Agilent (G7131C) was connected to a 1290 Bio Multisampler (G7137A, Agilent) via a MP35N capillary (internal diameter: 0.17 mm, length: 400 mm) and an Agilent inline filter was installed directly on the autosampler valve. Another capillary (internal diameter: 0.12 mm, length: 580 mm) led from the autosampler to a union. From the union, a PEEK capillary (internal diameter: 0.13 mm, length: 70 mm) was connected to a biocompatible PEEK back pressure regulator assembly (1000 psi, IDEX). Another PEEK capillary (length: 710 mm) was installed between the back pressure regulator and the switching valve of the ZenoTOF 7600 (SCIEX). The connection to the TurboV ion source (SCIEX), equipped with an ESI twin spray electrode (SCIEX), was completed with another PEEK capillary (length: 575 mm). The autosampler temperature was set to 10 °C and a 1:1 mixture of ACN:H_2_O was used as needle-wash.

Flow injection was performed with H_2_O + 0.1% FA as eluent A and MeOH as eluent B with an injection volume of 10 μL. Prior to each standard injection, data was acquired for a solvent blank (MeOH + 0.1% FA) using a flow rate of 0.25 mL/min and the following gradient: 0–2 min 50% B, 2.01–3 min 100% A, 3.01–4 min 100% B, 4.01–10 min 50% B. For data acquisition of standards, isocratic conditions of 50% B were applied, and the flow rate was changed as follows: 0–0.5 min 0.25 mL/min, 0.5–0.56 min ramp down to 0.05 mL/min and 0.005 mL/min at 0.57 min, from 0.57 to 3.7 min constant flow of 0.005 mL/min, 3.7 to 3.72 min increase to 0.25 mL/min and from 3.72 to 4 min 0.25 mL/min.

The source parameters of the ZenoTOF 7600 were set as follows: curtain gas: 35, CAD gas: 7, ion source gas 1:30 psi, ion source gas 2:20 psi, temperature: 200 °C. All FI-MS measurements were performed with MRM^HR^ in positive ion mode with a spray voltage of 5000 V. The TOFMS scan range was set to 65–450 Da (19 Da above the highest precursor *m*/*z* of the steroids under investigation), with an accumulation time of 0.1 s, a declustering potential (DP) of 100 V, and a collision energy (CE) of 10 V. For the TOFMSMS scans, Q1 resolution was set to unit, Zeno pulsing was activated for all ions below 80000 cps. TOF Start and Stop Masses of 50 and 440 Da were set in the MRM^HR^ transition table along with a DP of 100 V and accumulation times of 0.995 s unless denoted differently.

Separate MRM^HR^ acquisition methods were created for every standard by adapting the Compound IDs, Group Names, and Precursor Ion masses ([M + H]^+^) accordingly. CID and EAD fragmentations were performed within the same injection by setting the fragmentation mode in the MRM^HR^ transition table. Scheduling was not applied, resulting in consecutive loops across the whole transition table along the total measurement run time. For CID, the CE was ramped from 10 to 100 V in increments of 10 V. For EAD, the electron beam current was set to 5000 nA, a CE of 12 V was applied, and the electron kinetic energy was ramped from 8 to 24 eV in increments of 2 eV. The electron transfer current was set to 100%, the reaction time to 30 ms, and the EAD RF to 80 Da.

Quick and reproducible generation of transition tables was enabled by an in-house written R script (R: 4.4.1, R-Studio: ″Cranberry Hibiscus″ 2024.09.0 Build 375), which generates a .csv file containing a series of transitions for a user-defined compound across various fragmentation modes and energies, and can also be easily modified if necessary. A system stability and quick status check was performed prior to data acquisition, and autocalibration was performed approximately every hour throughout the sequence run.

### LC-MS Measurements

Chromatographic separation was performed on a biphenyl reversed phase column (Kinetex 2.1 mm × 100 mm, 2.6 μm, Phenomenex) equipped with a SecurityGuard ULTRA precolumn (2.1 mm × 2 mm, Phenomenex). Eluent A consisted of H_2_O + 0.1% FA and eluent B of MeOH + 0.1% FA. The following gradient was applied: 0–1 min: 60% B, 1–6 min: ramp to 80% B, 6–9 min: 80% B, 9–11 min: ramp to 100% B, 11–15 min: 100% B (all 0.2 mL/min), 15–17 min: 60% B (0.3 mL/min), 17–18 min: 60% B (0.2 mL/min). The column temperature was set to 40 °C and the injection volume to 1 or 5 μL, depending on the concentration of the standard (5 μM or 1 μM).

### Data Preprocessing and Analysis

Profile mode spectra were manually evaluated with the Explorer function of SCIEX OS (Version 3.3.1.43). To ensure the absence of carry-over between standard injections, the standard files, as well as the corresponding blank files acquired pre- and post the respective standard injection, were opened with SCIEX OS Explorer, and signal intensities in extracted ion chromatograms of the respective precursors in TOFMS-scans were manually evaluated. To assess spectral quality, 30 V CID and 14 eV EAD fragmentation spectra were matched against in silico predictions in SCIEX OS. The ratio of matched fragments was evaluated, setting a lower intensity threshold of ∼50 cps for both fragmentation modes and limiting mass errors to a maximum of 20 ppm. Mol files for evaluation in the SCIEX OS Explorer Fragments Pane were downloaded from NIST Chemistry WebBook,^33^ ChemicalBook,^34^ or ChEBI.^35^

Conversion of .wiff2 files to .mzML file format was performed using MSConvert^36^ (Version: 3.0.25218–15b0739 (automated build)). CentroidR^37^ was applied for subsequent centroiding. Spectral libraries were created using mzmine^38,39^ version 4.7.8 (custom version, see Supporting Information for processing parameters). Centroided .mzML files were used to evaluate the reproducibility of MS/MS scans across different triggering events via Hungarian cosine similarities, calculated for spectra originating from the same experiment (same CE in CID/same electron kinetic energy in EAD) based on their relative intensities. For this purpose, centroided spectra were processed with the help of Matchms^40,41^ using Python (version 3.11.13). Additionally, spectral comparison was also performed for .mgf files exported from mzmine. Breakdown curves were generated in Python based on preprocessing with Matchms.^40,41^ Data analysis scripts were written with the assistance of ChatGPT (the free online version), including thorough evaluation and adaptation of suggested code snippets.

Based on the spectral library .mgf file exported from mzmine, public library MS/MS spectra and data sets were searched for spectral matches using FASST,^42,43^ applying a cosine score threshold of 0.6 and requiring at least 6 matched peaks. Detailed information on processing parameters and the downstream data analysis can be found in the Supporting Information. When searched against publicly available raw data sets, FASST matches were generated separately for the existing public MS/MS libraries and our novel CID-EAD MS/MS libraries and filtered for samples of human origin. To unify thresholds for these searches a minimum cosine score of 0.7 was set and a minimum of 6 matched fragments were required. For demonstration purposes, we limited the output to data sets of human origin for which the sample matrix was specified. The resulting output was further reduced by retaining only unique combinations of a specific steroid found in a particular sample and body part, biological sex, and life stage. For the novel CID-EAD MS/MS library, the results were limited to CID matches within the scope of this comparison. Data analysis was performed using R and R-Studio (R: 4.4.1 and R-Studio: 2024.09.0 + 375 ″Cranberry Hibiscus″ or R: 4.4.2 and RStudio 2024.12.0 + 467 ″Kousa Dogwood″). A schematic overview of the workflow is presented in the Supporting Information (Figure S1).

## Results and Discussion

### Flow Injection-High-Resolution Targeted MS/MS Enables the Efficient Generation of High-Quality MS/MS Spectra

Flow injection is a versatile technique for library generation,^39,44^ providing stable signals over a longer timespan than chromatographic peaks and allowing fast data acquisition by avoiding the need for (extensive) gradient elution and column equilibration. By incorporating a backpressure regulator and fine-tuning injection volumes, capillary length, and flow rates, we achieved a stable signal plateau over 2.6 min using only a single pump system. The scan speed of the latest generation QTOF instruments,^45^ combined with a >2 min signal plateau, allows users to perform a variety of experiments within a single injection, while requiring low sample volumes (10 μL) of micromolar standards. Recognizing the need for broad coverage of fragmentation techniques and energies for efficient parameter optimization, mechanistic studies, high-quality MS/MS library generation, and the development of computational methods trained on MS/MS libraries, we acquired data for 19 different fragmentation settings within the stable signal region, covering both CID and EAD, as illustrated in Figure 1. The ample range of fragmentation energies included is not only beneficial for spectral libraries but also enables the efficient optimization of parameters required for sensitive high-resolution targeted MS/MS analysis. In more detail, we ramped collision energies from 10 to 100 V in increments of 10 V and electron kinetic energies from 8 to 24 eV in increments of 2 eV. These 19 transitions are triggered sequentially after each TOFMS scan, as presented in Figure 1. The resulting distribution of eight replicates per transition across the 2.6 min time window compensates for small variations in signal stability. Further, accumulation times were set to ∼ 1 s, to ensure high signal-to-noise ratios and ion statistics.^46^ For future applications, transition lists could also be expanded, e.g., by balancing accumulation times, the number of replicate MS/MS scans per fragmentation energy, and potential adducts of interest such as [M+NH_4_]^+^. It is important to note that certain adducts, such as [M + Na]^+^ or [M+K]^+^, can provide electron impact (EI)-like fragmentation spectra, suggesting an orthogonal use of EI spectral libraries.^11^ All data files acquired with the presented FI-high-resolution targeted MS/MS workflow can be easily visualized and shared via the GNPS dashboard, as explained in more detail in the Supporting Information.

**Figure 1:**
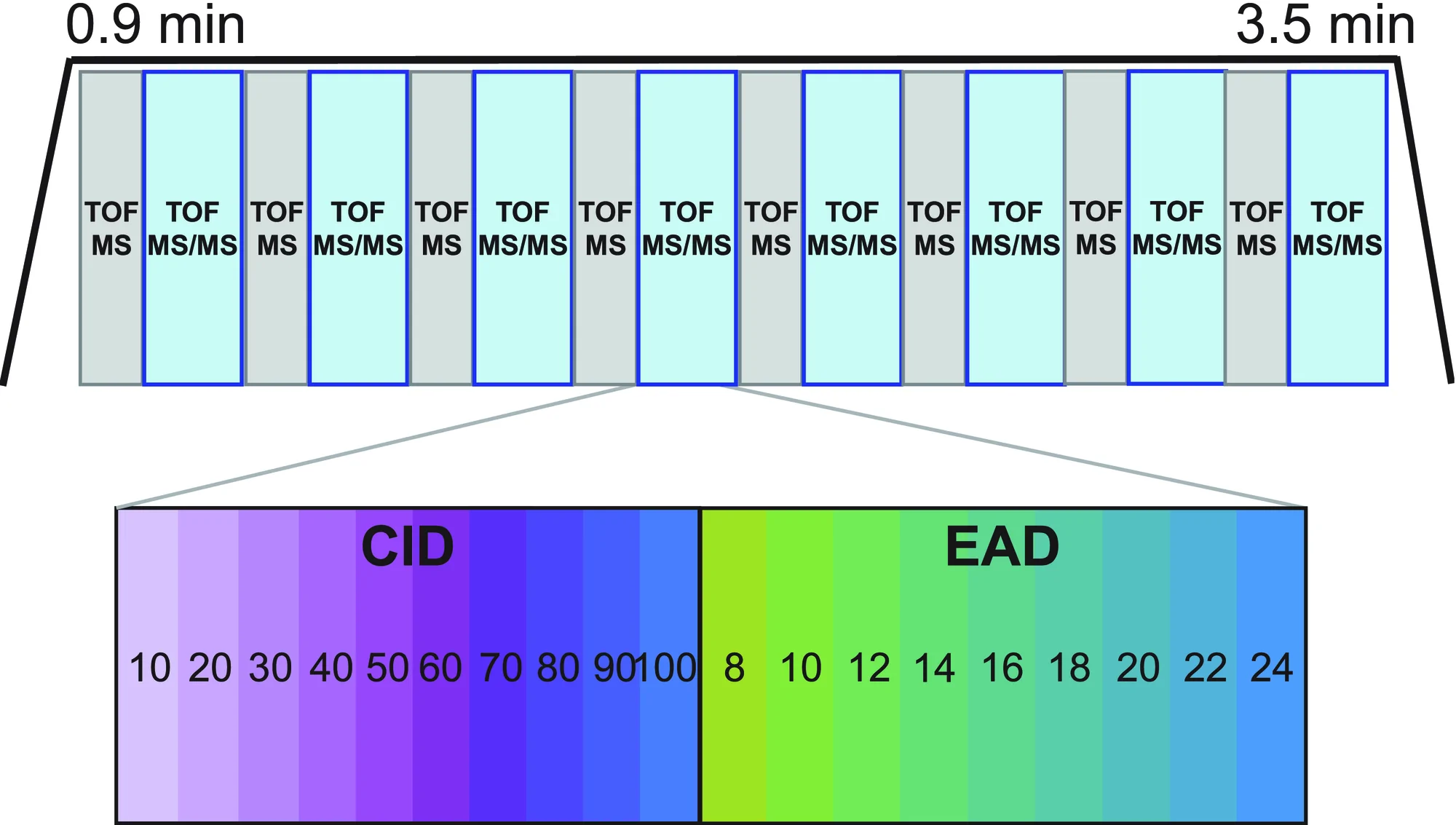
Schematic representation of the flow injection-high-resolution targeted MS/MS method enabling the generation of high-quality MS/MS spectra across a wide range of collision energies (CID) and electron kinetic energies (EAD). Each TOFMS scan is followed by 10 CID MS/MS scans (10–100 V) and 9 EAD MS/MS scans (8–24 eV) across the whole run time, resulting in eight triggering events per energy in the stable signal region of 0.9–3.5 min.

To demonstrate the developed FI-high-resolution targeted MS/MS workflow, we applied the acquisition strategy to steroids, creating approximately 850 unique MS/MS spectra from more than 40 single steroid standards (two-thirds of them exogenous). Experimental fragments were structurally annotated via an in silico fragmenter for an exemplary collision energy of 30 V in CID and an electron kinetic energy of 14 eV in EAD, as described in more detail in the Experimental section and in the Supporting Information. Setting a mass accuracy threshold of 20 ppm, more than 90% of the experimentally observed fragments were structurally annotated for most steroids, as explained in greater detail in the Supporting Information (Figures S2 and S3). Reproducibility across the eight triggering events was assessed by pairwise spectral comparisons using cosine scores. When comparing all MS/MS spectra from the same standard acquired under the same fragmentation parameters, cosine scores ≥ 0.95 were observed (see Supporting Information Figure S4 and Supporting Information Table (SA2)). Also, when matching spectral library entries generated by averaging across the eight triggering events against a corresponding scan, cosine scores were always ≥ 0.95. To avoid cross-contamination of standards, blank injections were performed between standard runs using an extensive flushing gradient with different solvent compositions, as outlined in the Experimental section. Carry-overs below 2% and excellent signal stability (deviation ≤ 25%) over approximately 2.6 min make the proposed flow injection-high-resolution targeted MS/MS acquisition strategy fit for purpose. Blank samples were included and checked for background fragments (Supporting Information Table (SA3) for EAD). Overall, the workflow produced high-quality spectra, characterized by high mass accuracies and a high signal-to-noise ratio.

### Automated Optimization of MS/MS Parameters

The proposed strategy provides a partially automated alternative to state-of-the-art fragmentation-parameter optimization workflows. Currently, parameter optimization, especially for EAD, often involves either multiple LC-MS runs or manual instrument operation. LC-MS approaches are prone to being limited by the inherent trade-off between the number of fragmentation energies and cycle times, which is influenced by the interplay among accumulation times, the number of transitions, and peak widths. Other workflows for parameter optimization rely on direct infusion via a syringe pump, representing a limiting factor for high-throughput analysis. These dedicated workflows are often not designed for spectral library generation.

We acquired nearly 100 breakdown curves (see Supporting Information – Breakdown curves) for steroids for CID and EAD across a wide range of collision energies and electron kinetic energies. Each breakdown curve plot depicts the 12 most abundant average mass to charge ratios and their intensities with respect to the applied energy, as displayed in Figure 2 for CID (**A**) and EAD (**B**) for prednisolone. In both fragmentation modes the precursor at *m*/*z* 361.2010 (C_21_H_29_O_5_^+^) produces the most intense signal (CID:∼120,000 cps, EAD: ∼ 23,000 cps). In CID, the impact of fragmentation energies on signal intensities can be readily deduced in the presence of the precursor; however, in EAD, the precursor’s dominance, driven by the underlying fragmentation mechanism,^20,47^ masks fragment intensities, and is excluded for data visualization (see Supporting Information – Breakdown curves), while the underlying data remains unchanged. Fragments originating from water losses (C_21_H_27_O_4_^+^, C_21_H_25_O_3_^+^, C_21_H_23_O_2_^+^), and the 4a-methyl-5,6,7,8-tetrahydronaphthalen-2-one backbone (C_12_H_13_O^+^, C_12_H_11_O^+^, C_10_H_11_O^+^, C_8_H_9_O^+^) are among the highest ones for both techniques. While in EAD all of the top 12 fragments are found across the electron kinetic energy range, a strong energy dependence can be seen for CID, where some fragments are not observed at lower/higher collision energies. This implies that the selectivity of EAD fragments is less affected by the electron kinetic energy, and that electron kinetic energies can be tuned more uniformly than collision energies in CID to cover a wide range of fragments.

**Figure 2:**
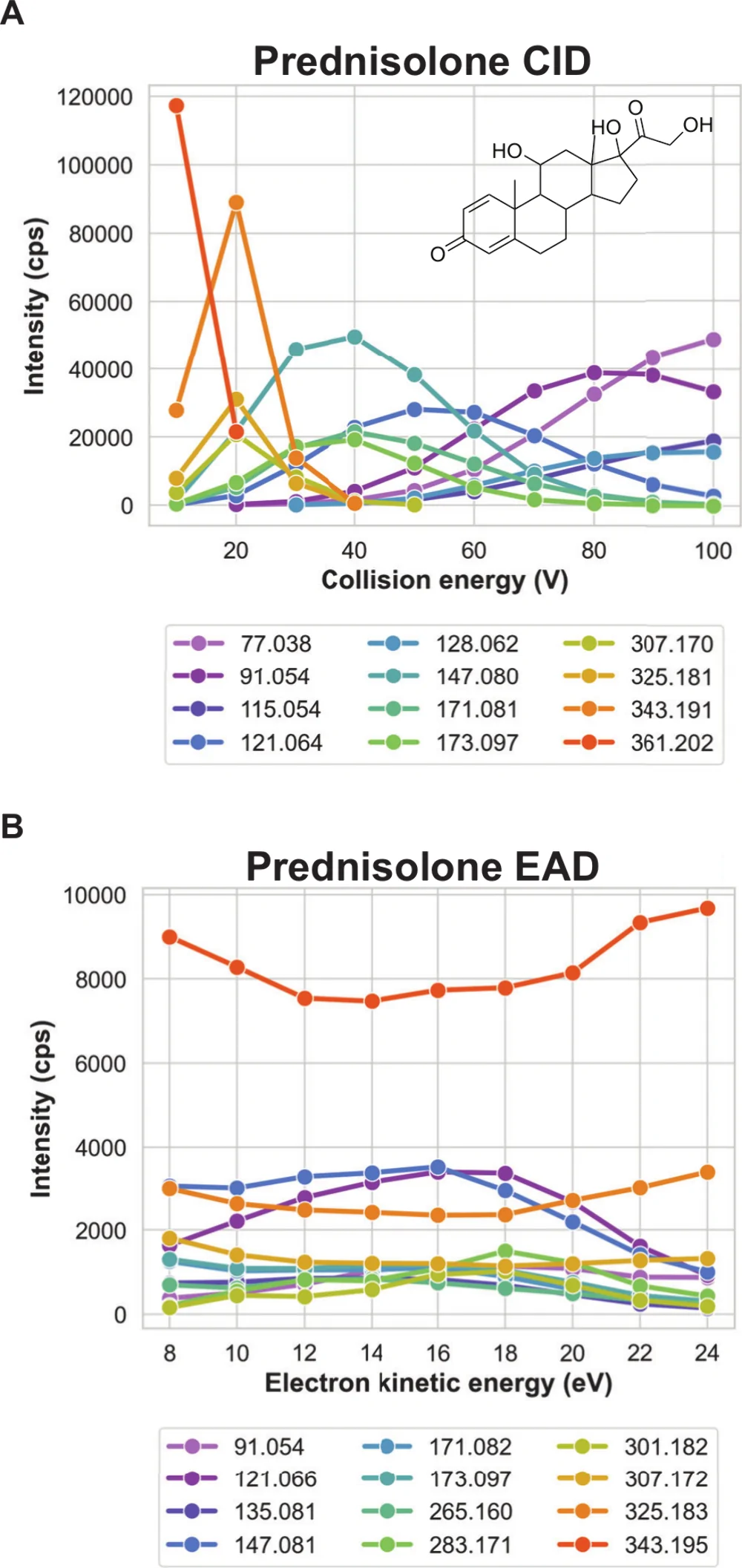
Exemplary breakdown curves of prednisolone ([M + H]^+^, *m*/*z* 361.2010) for (A) CID and (B) EAD. To illustrate the impact of electron kinetic energies on fragment intensities for EAD, the precursor was removed for visualization. The corresponding breakdown curve, including the precursor, is available in Supporting Information - Breakdown curves.

In addition to automated data acquisition and analysis, we recommend verifying fragment ions using in silico tools and/or high-quality spectral libraries, paired with the evaluation of blank samples, especially for EAD fragmentation as mentioned previously. While we recognize the need for automated parameter optimization, we also emphasize the importance of informed manual curation of fragment spectra during LC-MS method development for biological samples. Moreover, it should not be overlooked that the lack of chromatographic separation inherent to flow injection-based workflows increases the risk of interference from coeluting species. To assess impurities, we acquired LC-MS data for all steroids. Among these, only androst-4-ene-3, 6, 17-trione (retention times: 6.5 and 8.2 min) and budesonide (retention times: 8.5 and 9.0 min) showed impurities with clearly separated chromatographic peaks (second highest peak > 40% of base peak).

### Evaluating the Significance of the Steroid CID-EAD MS/MS Library through Public Metabolomics Raw Data

Steroids are essential given their importance for reproductive functions, the immune system, and stress regulation^48^ – to name only a few aspects – in addition to their wide application for the treatment of various diseases^49^ and their abuse in doping.^50^ Successful structural annotation of steroids in nontargeted metabolomics data sets, however, is inherently dependent on the coverage of these molecules in spectral libraries. In this sense, the term coverage is not limited to the number of steroids in MS/MS libraries but includes the diversity of instruments, fragmentation modes, and energies. Through this work, we expanded the set of steroids with publicly available EAD spectra by 36 compared to the MS-DIAL 5 and/or MultiMS2 libraries,^17,26^ and we added six steroids for which not even CID/HCD spectra had previously been available in MoNA,^28^ MassBankEU,^29^ or GNPS.^30^

After acquiring more than 850 MS/MS spectra for 45 steroid standards across 19 fragmentation conditions, we assessed spectral quality by searching our CID and EAD spectra against public MS/MS libraries using the FASST matching algorithm (see Experimental section). We then examined all library matches meeting our thresholds (cosine score ≥ 0.6 and ≥ 6 shared peaks) and compared the library annotations to the identity of the queried steroid to evaluate whether differences between public spectra and spectra generated by our workflow could plausibly lead to misannotations. Using this approach, we confirmed that most of the acquired steroids (36) matched at least one public library spectrum from a compound with the same 2D structure. Notably, this was observed not only for CID spectra (90%) but also for EAD spectra (85%). Figure 3A provides an overview of true positive (green) and false positive (shades of red) library matches for both CID and EAD fragmentation for all steroids for which true-/false positive library matches were found. Steroids for which public library spectra would have been available, but that did not return any kind of match were metenolone and calusterone. We further assessed potential sources of misannotation by quantifying matches to compounds other than the queried steroid, and found that for CID 66% and for EAD 89% of these mismatches corresponded to steroid-like structures, as shown for a subset of molecules in Figure 3A, and in greater detail in the Supporting Information Table SA4. While putatively identifying a feature of interest as a steroid can already provide valuable insights, more detailed structural information is required to differentiate isomers, which remains challenging with state-of-the-art library matching. For example, our library search for 17-α-hydroxyprogesterone also returned false positive matches for one of its isomers 21-hydroxyprogesterone, differing solely in the position of a hydroxy group. However, unequivocal differentiation of these steroids is key to distinguishing subtypes of congenital adrenal hyperplasia (CAH), a genetic disorder that affects steroid synthesis in the adrenal glands.^51,52^ While elevated levels of 17-hydroxyprogesterone are observed in patients suffering from the most common type of CAH, 21-hydroxylase deficiency, also 21-hydroxyprogesterone is increased in the less prevalent 11β-hydroxylase deficiency subtype. When performing this kind of repository-scale MS/MS matching, users need to be aware that annotation confidence is limited to level 2 as defined by the Metabolomics Standards Initiative (MSI).^53^ Despite recent advancements in reducing false-positive results in steroid isomer identification,^54^ library matching should never be considered proof of a chemical identity, which requires additional data such as retention time matching of an analytical standard, isolation, and NMR.^53^ By adjusting thresholds for cosine scores and shared peaks, the number of false positive assignments could be reduced in many cases, as presented in Supporting Information – Threshold evaluation. As there is no one-size-fits-all solution for setting threshold values, assessments like this can serve as a starting point for per-compound threshold optimization. For example, for CID false positive matches for trenbolone could be eliminated at a cosine score threshold as low as 0.7, but even high cosine score thresholds of 0.9 were not sufficient to avoid mismatches for fluoxymesterone, which could only be removed by applying a threshold of eight shared peaks. The original output of the library search is provided in Supporting Information Table SA5.

**Figure 3:**
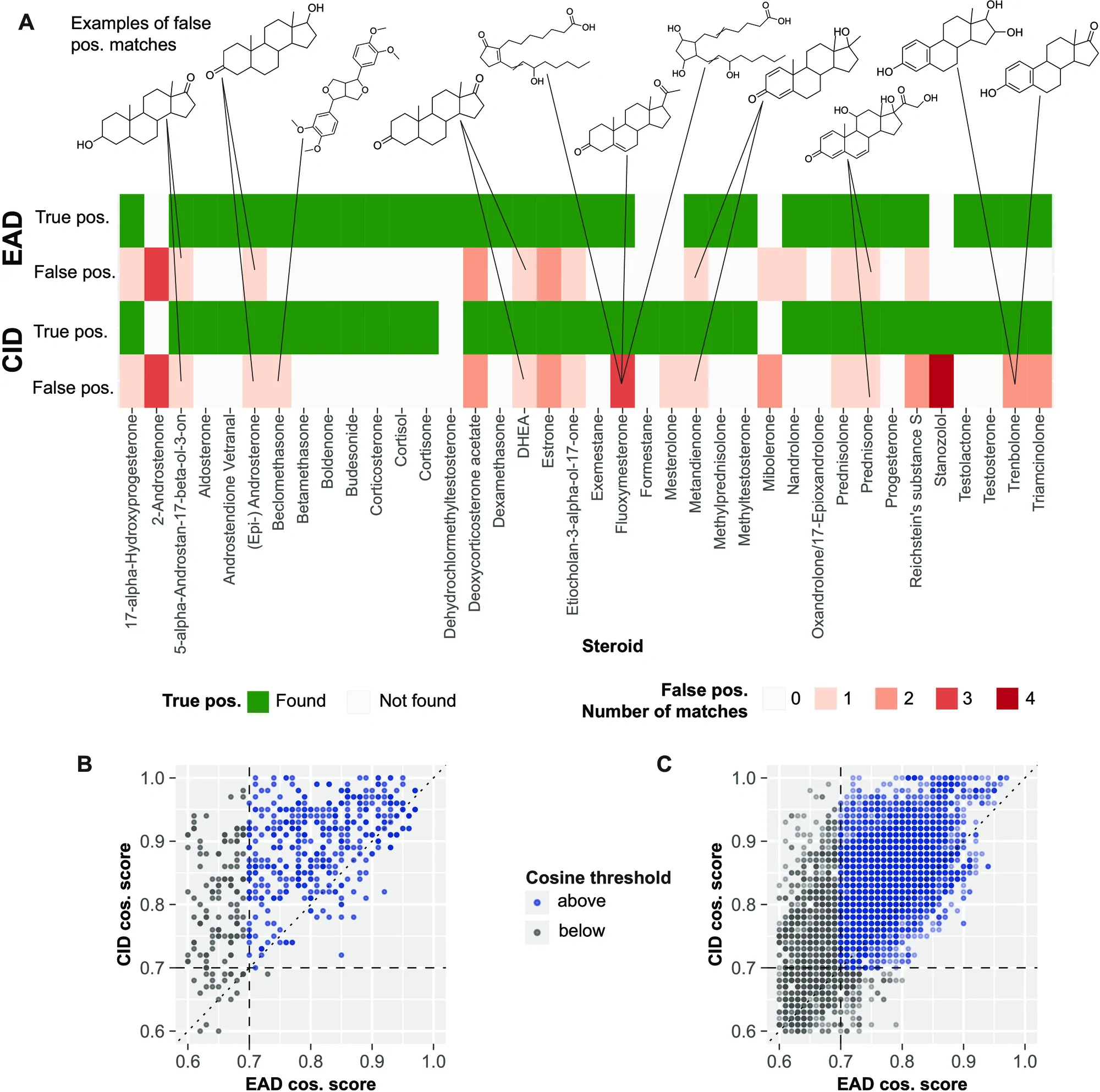
Results of public library and repository searches using the novel MS/MS CID-EAD steroid library. (A) When searched against existing spectral libraries using a cosine score threshold of 0.6 and a minimum number of six shared peaks, the novel MS/MS CID-EAD steroid library returns true positive matches (green) for both fragmentation modes. Between zero and four false positive matches, defined by unique InChIKeys, were found per steroid (colored in shades of red). Most false positive matches showed high structural similarity to steroids. Steroids with neither true nor false positive matches were excluded from the graph. (B and C) Matches to spectral library entries that were associated with both fragmentation modes are indicated by dots. The position of each dot is defined by the maximum cosine score found for EAD (*x*-axis) and CID (*y*-axis). If both scores exceed a cosine score threshold (th) of 0.69, points are colored blue; otherwise, they are colored gray. True positive library matches are represented in panel B (477 matches > th, 140 ≤ th), and matches to biological samples in panel C (4760 matches > th, 1484 ≤ th).

The observation that EAD spectra of steroids can be matched to public CID/HCD spectra using cosine-based MS/MS similarity is important, since public MS/MS libraries are primarily composed of CID/HCD data. Demonstrating that these libraries can still support annotation of EAD spectra means EAD data could potentially be integrated into standard library-search metabolomics workflows without requiring dedicated EAD reference libraries, while maintaining structurally informative EAD fragments for manual interpretation. To further assess this prospect, we compared the cosine scores obtained through the matching of the steroid spectra acquired here with their counterparts in public libraries (Figure 3B). While we observed almost universally higher scores for CID-CID than for EAD–CID matches (84%), most spectral matches that led to scores ≥ 0.7 for CID spectra also did so for EAD spectra 477/617 (77%). This suggests that while current CID/HCD libraries are indeed a valuable resource for steroid-EAD data, some compromise on relative performance is to be expected. For many steroids, true positive matches are found at electron kinetic energies yielding good coverage of EAD fragments with near-maximal signal intensities (Figure S5), thus maintaining EAD’s versatility for structural elucidation while at the same time offering potential for successful library matching. To ensure efficient ion transmission, low collision energies (10–12 V) are applied for MS1 and EAD scans. Consequently, spectral similarities may arise from low-energy CID data embedded in EAD MS/MS spectra.^55^ Exemplary mirror plots of acquired spectra and existing MS/MS library entries are depicted in Figures S6–S11.

To assess the validity of our findings beyond steroids, we used publicly available CID and EAD data of pesticide standards.^12,56^ True positive matches against existing libraries were returned for both fragmentation modes (see Figure S12). For EAD only three false positive matches were found. Yet, both for CID and for EAD, fewer compounds had library matches compared to steroids, an effect that could potentially originate from acquisition parameters and/or the substance class under investigation. For example, the public pesticide EAD spectra were acquired at lower collision energies (10 V compared to 12 V for steroids) and at an electron kinetic energy of 18 eV. The ratio of true positive EAD to true positive CID matches at the same electron kinetic energy (18 eV), is comparable between steroids (49% (Figure S5)) and pesticides (34%). While the discovery of true positive library matches for pesticides supports our hypothesis, the generalizability of matching EAD data to CID/HCD spectra across diverse compound classes requires generating much larger EAD libraries, facilitated by the workflows described here, and will be addressed in future work.

Although observations based on the comparison of MS/MS acquired from analytical standards are informative, a central purpose of spectral libraries is their utility for the annotation of biological data. Therefore, we employed the here acquired steroid library to annotate biological MS/MS raw data available in public metabolomics repositories using the FASST algorithm. One goal of this was to assess whether the generated EAD and CID libraries would show comparable performance as they did in matching public libraries. As presented in Figure 3C, spectral matches to both fragmentation modes can be found for more than 6200 data points representing unique combinations of a steroid, data file, sample matrix, age, and biological sex found in data sets of human origin. With 76% of the matches exceeding the cosine score threshold of 0.69 in both fragmentation modes, the ratio of matches above the threshold, represented in blue, is almost the same as for the spectral library comparison. This strengthens the evidence that cross-fragmentation mode-based library matching holds utility also for biological data.

To assess the reliability of the retrieved raw-data matches across the diverse set of public metabolomics raw data, we integrated harmonized (pan-ReDU^57^) metadata and evaluated whether informative biodistributions could be observed. This utility is essential, as public libraries are increasingly used to retrieve matches for this purpose.^58,59^ In Figure 4, we illustrate the relative matching frequencies across samples from human body parts for the generated CID library (top) and previously existing MS/MS library spectra (bottom). The similarity of the relative distributions across body parts between the data sets demonstrates that spectra collected via the presented pipeline show comparable utility to other public spectra found in MoNA,^28^ MassBankEU,^29^ or GNPS.^30^ We hypothesize that the wide range of energies covered by the novel steroid MS/MS library could potentially enhance the number of spectral matches (see Figure S13), as it has recently been shown that MS/MS acquisition over a wide range of energies can improve the identification of metabolites.^60^ For a comprehensive overview of all FASST matches, the interested reader is referred to the Supporting Information Tables SA6 and SA7, where the FASST output files of both searches are available. At this point, we would like to emphasize that we do not aim to provide any in-depth biological interpretation of the presented results, but instead focus on a few examples highlighting how these results – which are based on MSI - level 2 annotations^53^ – can be interpreted.

**Figure 4:**
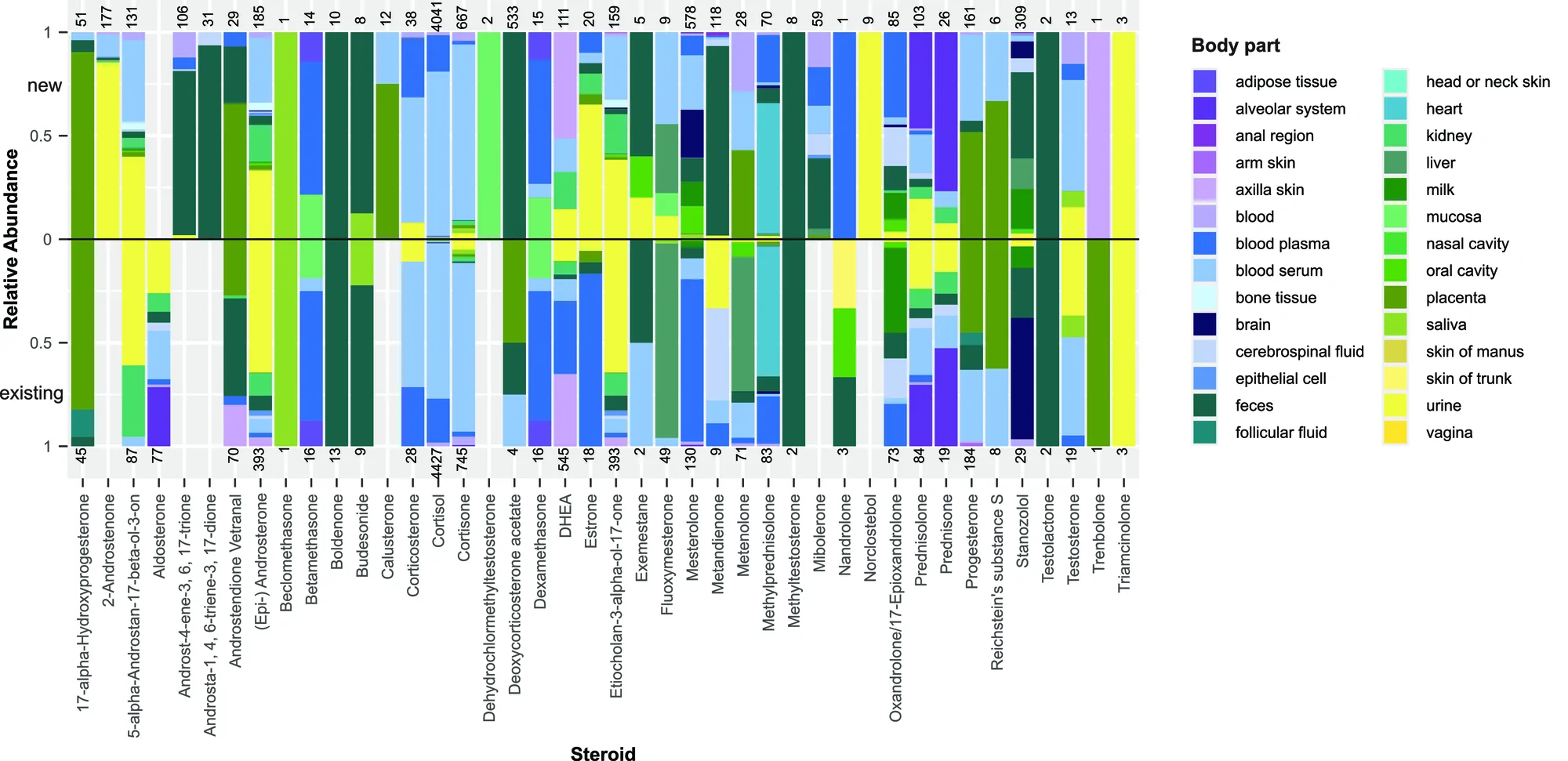
By searching previously existing (bottom) and new (top) MS/MS libraries against public raw data, similar relative distribution patterns can be deduced across steroids. However, some matches are only enabled using the novel steroid library. The absolute number of matches defined by unique combinations of steroids found in a particular sample, body part, biological sex, and life stage is indicated at the top/bottom of the respective bars. Lower thresholds for matches were set to 0.7 for cosine scores and six matched fragments.

Several matches were retrieved only with the novel library generated by the presented pipeline. Given the important role of steroids in human pregnancy,^61^ we will focus on two studies investigating pregnancy metabolomics (MSV000098454 ^62^) and exposomics (ST003616 ^63^). Both data sets were acquired using reversed-phase chromatography coupled to Orbitrap systems (Q Exactive Plus/Exploris 240) and analyzed placenta and/or blood serum samples. For instance, 17-α-hydroxyprogesterone was detected in human blood serum, and 5-α-androstan-17-β-ol-3-one in placenta samples. In the data set focusing on pregnancy metabolomics, we also found corticosterone, cortisol, cortisone, and progesterone in blood serum solely through the presented library. A recent study by Karahoda et al.^61^ supports the presence of these steroids in these sample matrices. While the ability of spectral matching to annotate molecules accurately has been demonstrated across countless metabolomics and proteomics studies it is well-known that incorrect annotations can occur. Library matches, including those presented in this study, need to be evaluated with care and should be regarded solely as a tool for hypothesis generation, limited to putative annotations. Confident identification of features of interest can only be achieved by additional experiments using reference standards, corresponding retention times, and, if applicable, even orthogonal methods.^53^

For example, in both studies calusterone, a steroid that was explored as a breast cancer drug^64^ and that is used in doping,^50^ is shown to match the placenta/blood serum samples of pregnant women. A more likely scenario, however, is that this is a mismatch of this molecule with a structurally similar isomer, such as pregnenolone, an endogenous metabolite known to be increased during pregnancy.^61,65^ While pregnenolone could not be retrieved as a potential interference in the analysis presented in Figure 3A, a follow-up search confirmed an MS/MS match between calusterone and a public library entry of pregnenolone at a cosine score of 0.5. Other matches, which were not retrieved with previously available libraries such as norclostebol in urine samples, could be plausible from a biological point of view. However, retention-time confirmation, an orthogonal line of evidence considered state-of-the-art for validating MS/MS-based matches in analytical chemistry, is not possible for the type of public-data-driven MS/MS matching performed here. We therefore opted for a different kind of orthogonal evidence based on prior biological knowledge, a strategy previously suggested for taxonomically enhanced confidence scores.^66^

Specifically, the enzymatic conversion of cortisol to cortisone in the placenta was described as early as the 1960s.^67,68^ In this regard the data set MSV000094962,^69^ which contained matched maternal blood and placenta samples, was investigated more closely. In this data set, cortisol matches were exclusively associated with blood samples, while cortisone matches were solely associated with placenta samples. Based on this observation, peak areas of cortisol and cortisone were integrated in Skyline.^70^ As visualized in Figure 5, cortisone was observed to be more abundant in the placenta and cortisol in maternal blood. The alignment between the MS/MS-based annotations and the known abundance patterns, therefore, provides supporting evidence for the annotations.

**Figure 5:**
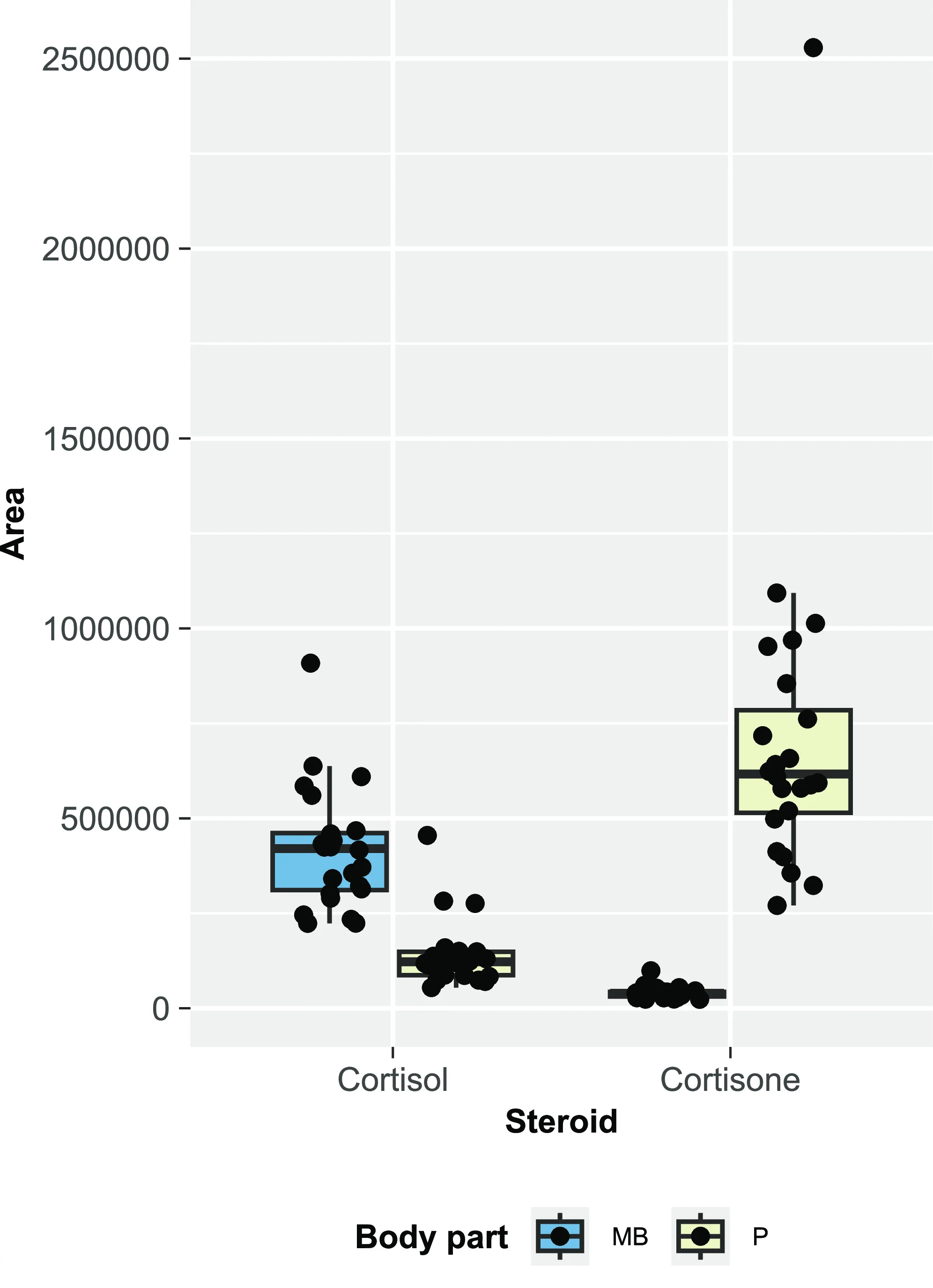
Comparison of peak areas of cortisol and cortisone in maternal blood (MB) and placenta samples (P).

## Conclusion

The presented FI-high-resolution targeted MS/MS workflow enables quasi-simultaneous acquisition of EAD and CID fragment spectra across a wide energy range, requiring low injection volumes. The resulting high-quality MS/MS spectra support both targeted and nontargeted analysis, given their potential for fragmentation-parameter optimization, MS/MS spectral libraries, and the development of computational tools for MS/MS-based structural elucidation.^71,72^ Comprehensive MS/MS libraries incorporating different fragmentation modes and energies hold great potential for the detailed structural elucidation of isomers, the identification of characteristic fragments, and the localization of structural modification sites. To this end, we have published a library containing 459 CID MS/MS spectra and 414 EAD MS/MS spectra acquired in positive ion mode.

Currently, many nontargeted studies are performed using CID even when EAD fragmentation is supported on the instrument in question, applying EAD, if required, only as a secondary method for detailed structural elucidation. Our observation that EAD spectra of steroids can be annotated via existing CID/HCD-based public library entries suggests that matching EAD raw data to those libraries could in-fact supplement manual interpretation and traditional fragmentation rule-based approaches for EAD spectra. While the broader validity of this finding across diverse chemical classes remains to be assessed, it suggests that the utility of untargeted EAD-based metabolomics is greater than previously thought.

Overall, while we describe the acquisition workflow for a state-of-the-art QTOF, the acquisition strategy can in principle be adopted to other instruments, which allow for the specification of user- defined transitions and MS/MS parameters and the acquisition of high-resolution MS/MS spectra across a wide mass range. High-quality MS/MS libraries and breakdown curves can be easily generated from vendor-neutral data files that contain fragment mass-to-charge ratios and corresponding signal intensities for each compound, along with acquisition parameters. Beyond CID and EAD, the proposed flow-injection high-resolution targeted MS/MS workflow could also be adjusted to accommodate other library modalities such as UVPD. This way, the user-friendly and efficient workflow will support the community effort to increase coverage in public spectral libraries, providing a valuable resource for future nontargeted small molecule omics studies.

## Supplementary Material









## Data Availability

Spectra were contributed to public libraries via GNPS. Data files are publicly available on MassIVE (MSV000100658).

## Notes

Summary of all generated breakdown curves for CID and EAD (PDF)
Overview of the impact of different cosine scores and minimum matched peak thresholds on the total number of hits and the relative distribution of true and false positive hits (PDF)
A list of steroid standards that were analyzed with the presented flow injection-high-resolution targeted MS/MS-approach (Table SA1), mean cosine scores per steroid and fragmentation mode (Table SA2), a list of common background fragments observed for EAD fragmentation (Table SA3), a list of false positive library matches (Table SA4), output of the FASST searches of the novel CID-EAD MS/MS library against existing spectral libraries (Table SA5), original output of the FASST search of the novel CID MS/MS library against repository raw data (Table SA6), and filtered output of the FASST search of previously existing libraries against repository raw data (Table SA7) (due to the file size, Table SA7 is limited to the results presented in Figure 4) (XLSX)
Supporting experimental section and results that include additional LC-MS data acquisition and SCIEX OS processing parameters, as well as additional details about data processing in mzmine and for the FASST-search, a schematic overview of the workflow, additional information about the GNPS-dashboard, an overview of fragment numbers across steroids, and observed fragment mass errors, exemplary spectral library matches to CID and EAD spectra, results of spectral library matching of pesticides, and the importance of wide energy coverage for spectral matching (PDF)
