# Supplementary material for: From Single Injection to Dual Insights: Integrating EAD and CID Acquisition for Spectral Library Generation and EAD–CID Spectral Matching
Source: Anal Chem. 2026 Oct 2;98(41):29433–44. doi: 10.1021/acs.analchem.6c01204 (PMC13689410; doi:10.1021/acs.analchem.6c01204)
Supplement: Supplementary file 1 [file ac6c01204_si_001.pdf]

Supporting Information- Breakdown curves

From Single Injection to Dual Insights: Integrating EAD and CID acquisition for Spectral Library Generation and EAD–CID Spectral Matching

Lisa Panzenboeck[1,2], Christina Brenner[1,2], Veronika Fitz[1,2], Mingxun Wang[3], Patrick Mueller[1], Evelyn Rampler[1,2], Yasin El Abiead[4]* and Gunda Koellensperger[1,5]*

1 Department of Analytical Chemistry, Faculty of Chemistry, University of Vienna, 1090 Vienna, Austria

2 Vienna Doctoral School in Chemistry (DoSChem), University of Vienna, 1090 Vienna, Austria

3 Department of Computer Science and Engineering, University of California Riverside, Riverside, California 92521, United States

4 BOKU University, Department of Chemistry, Institute of Analytical Chemistry, Muthgasse 18, 1190, Vienna, Austria

5 Vienna Metabolomics Center (VIME), University of Vienna, 1090, Vienna, Austria

* Corresponding authors:

Gunda Koellensperger: gunda.koellensperger@univie.ac.at, +43-1-0277-52303

Yasin El Abiead: yasin.el-abiead@boku.ac.at, +43-1-47654-77109

All mass to charge ratios represent average values.

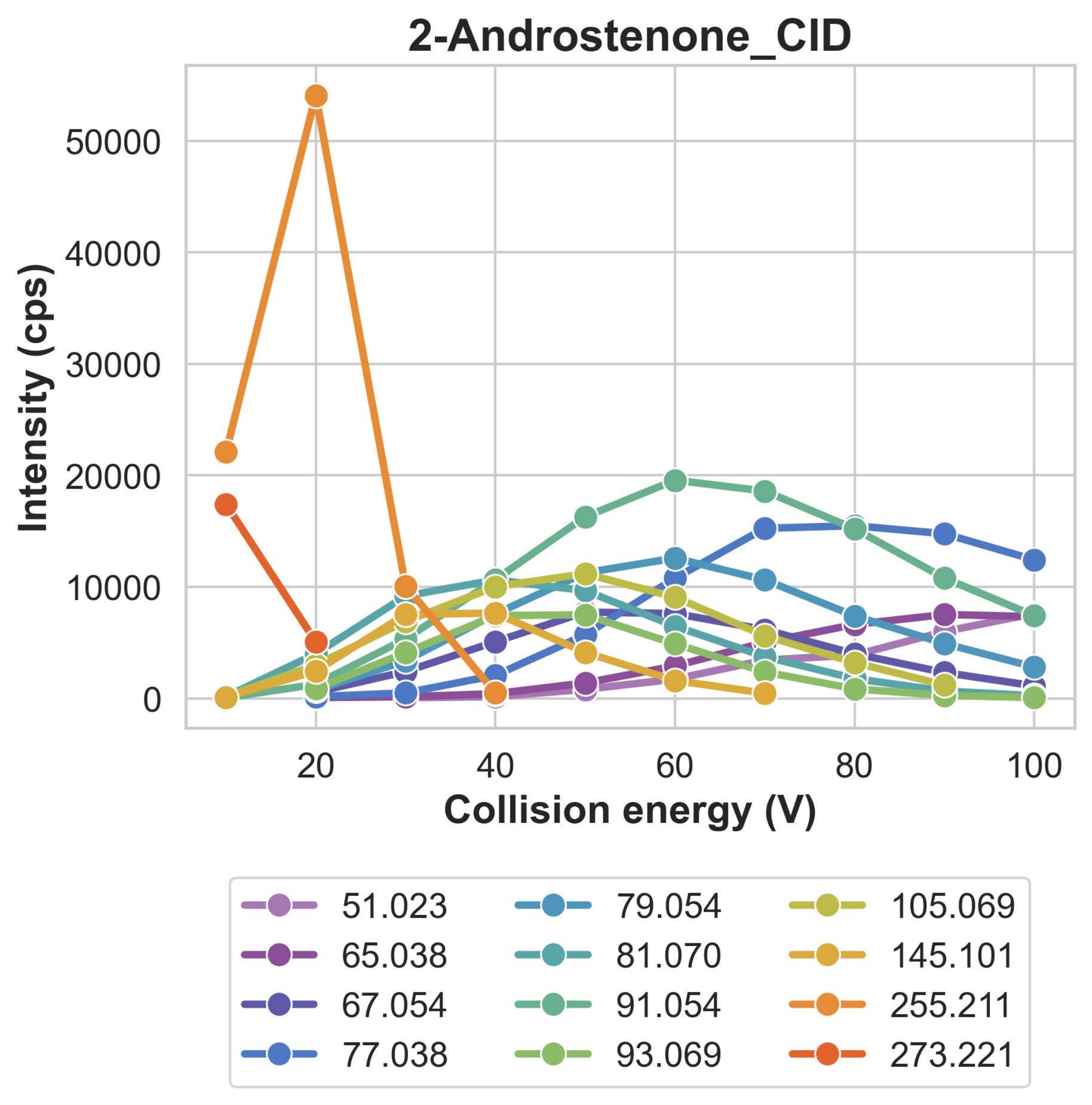
2-Androstenone_CID
Intensity (cps)
50000
40000
30000
20000
10000
0
20
40
60
80
100
Collision energy (V)
51.023
65.038
67.054
77.038
79.054
81.070
91.054
93.069
105.069
145.101
255.211
273.221

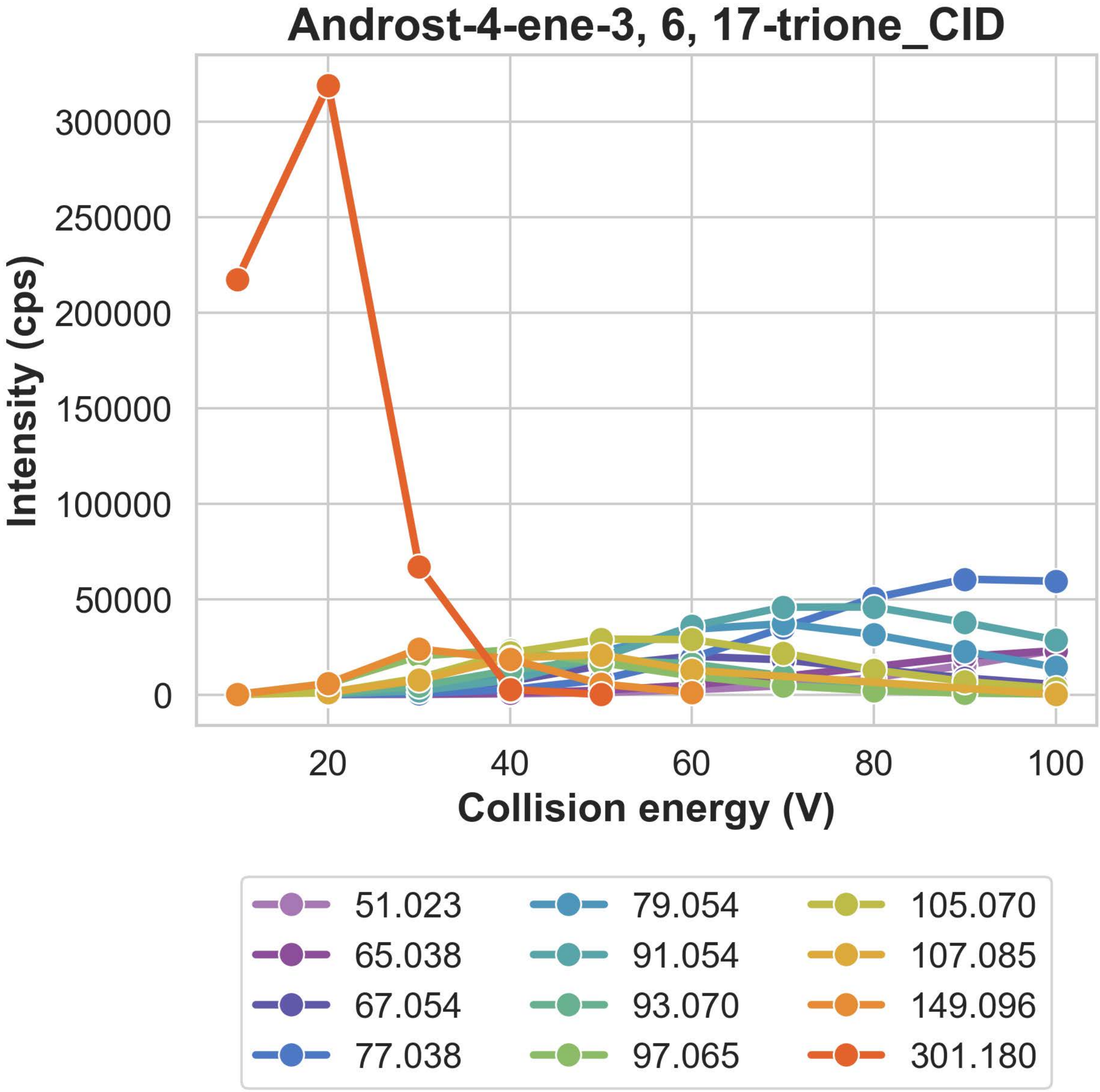

Androst-4-ene-3, 6, 17-trione_CID
Intensity (cps)
300000
250000
200000
150000
100000
50000
0
20
40
60
80
100
Collision energy (V)
51.023
65.038
67.054
77.038
79.054
91.054
93.070
97.065
105.070
107.085
149.096
301.180

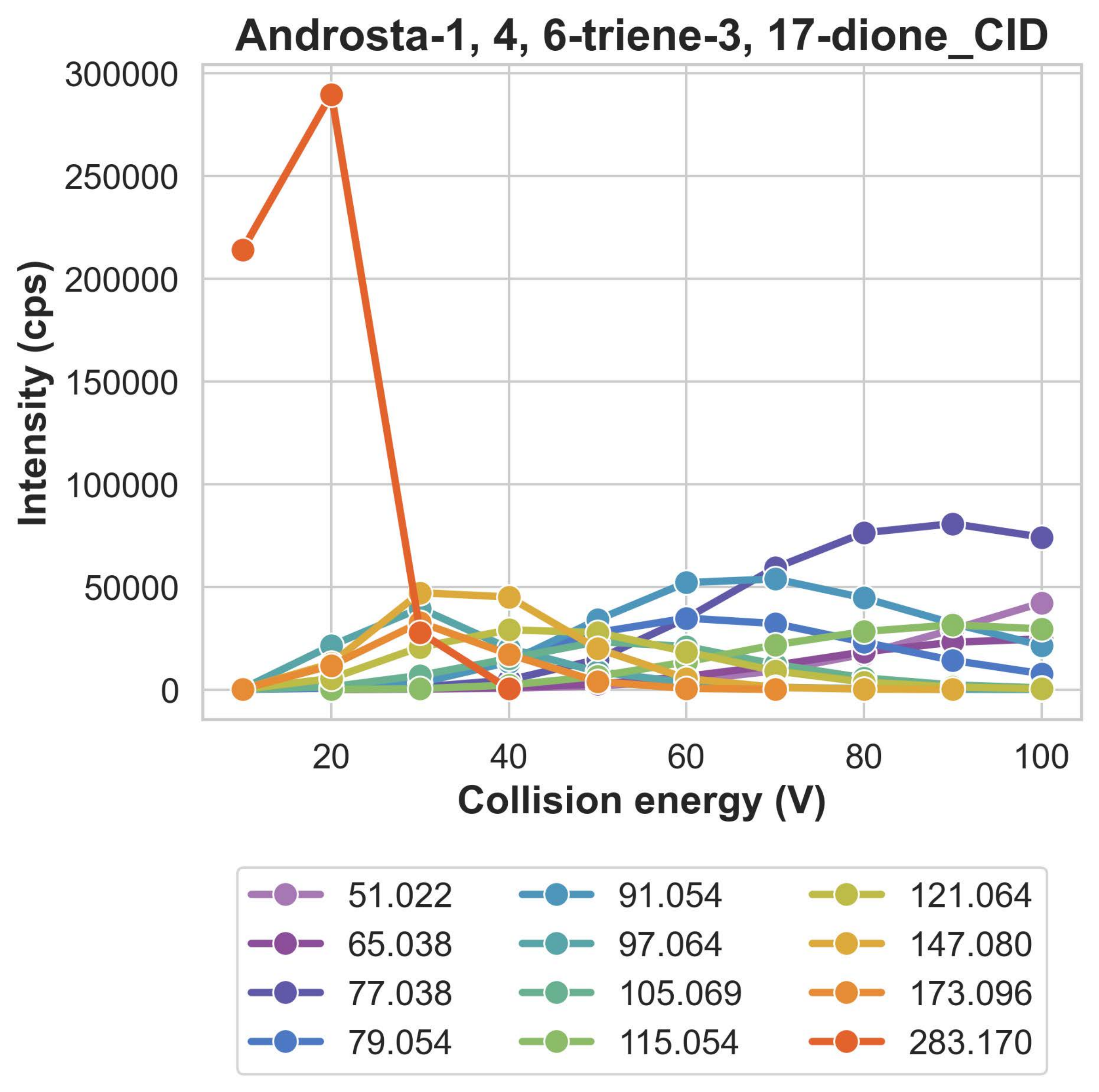
Androsta-1, 4, 6-triene-3, 17-dione_CID
Intensity (cps)
300000
250000
200000
150000
100000
50000
0
20
40
60
80
100
Collision energy (V)
51.022
65.038
77.038
79.054
91.054
97.064
105.069
115.054
121.064
147.080
173.096
283.170

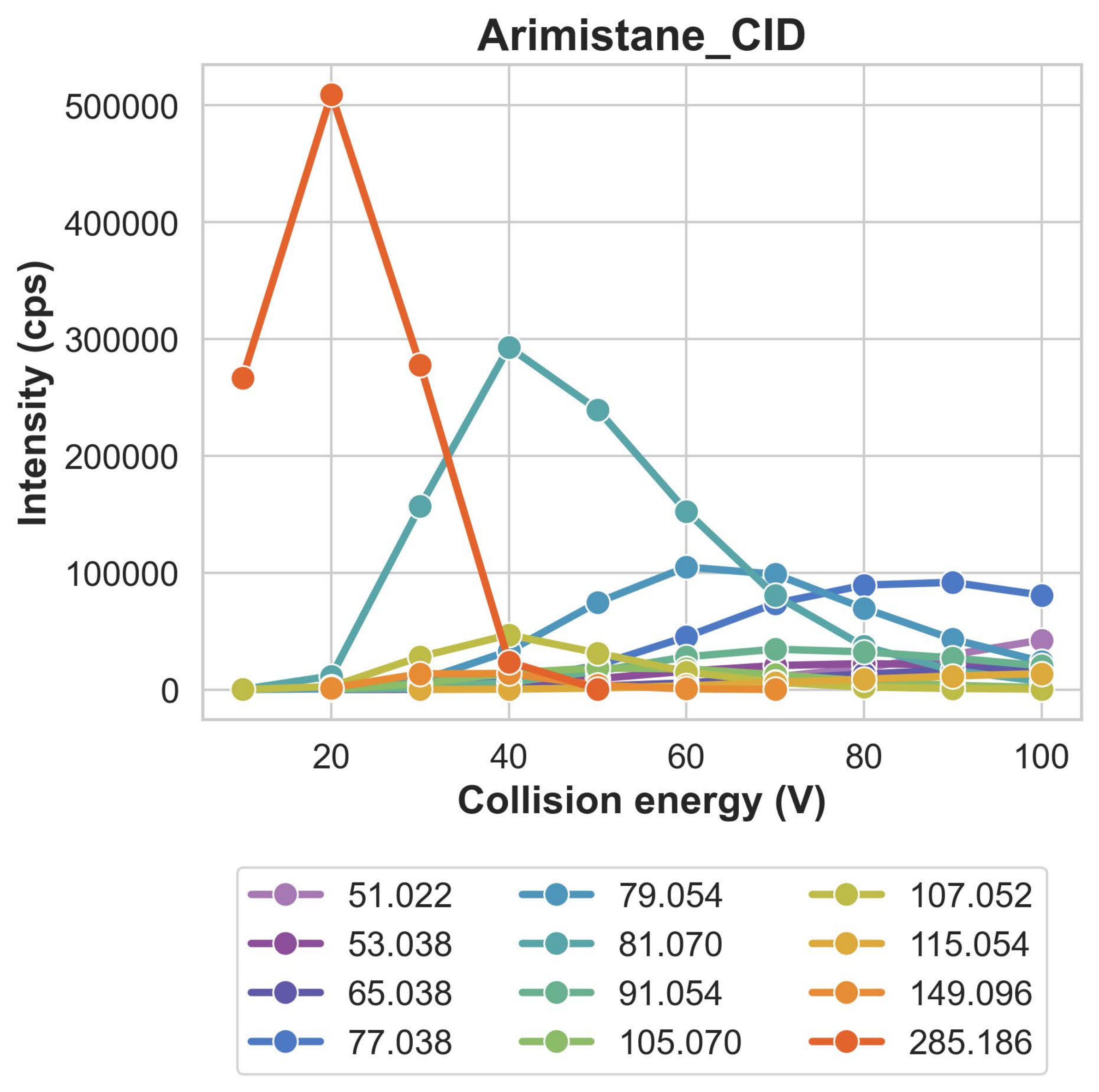
Arimistane_CID
Intensity (cps)
500000
400000
300000
200000
100000
0
20
40
60
80
100
Collision energy (V)
51.022
53.038
65.038
77.038
79.054
81.070
91.054
105.070
107.052
115.054
149.096
285.186

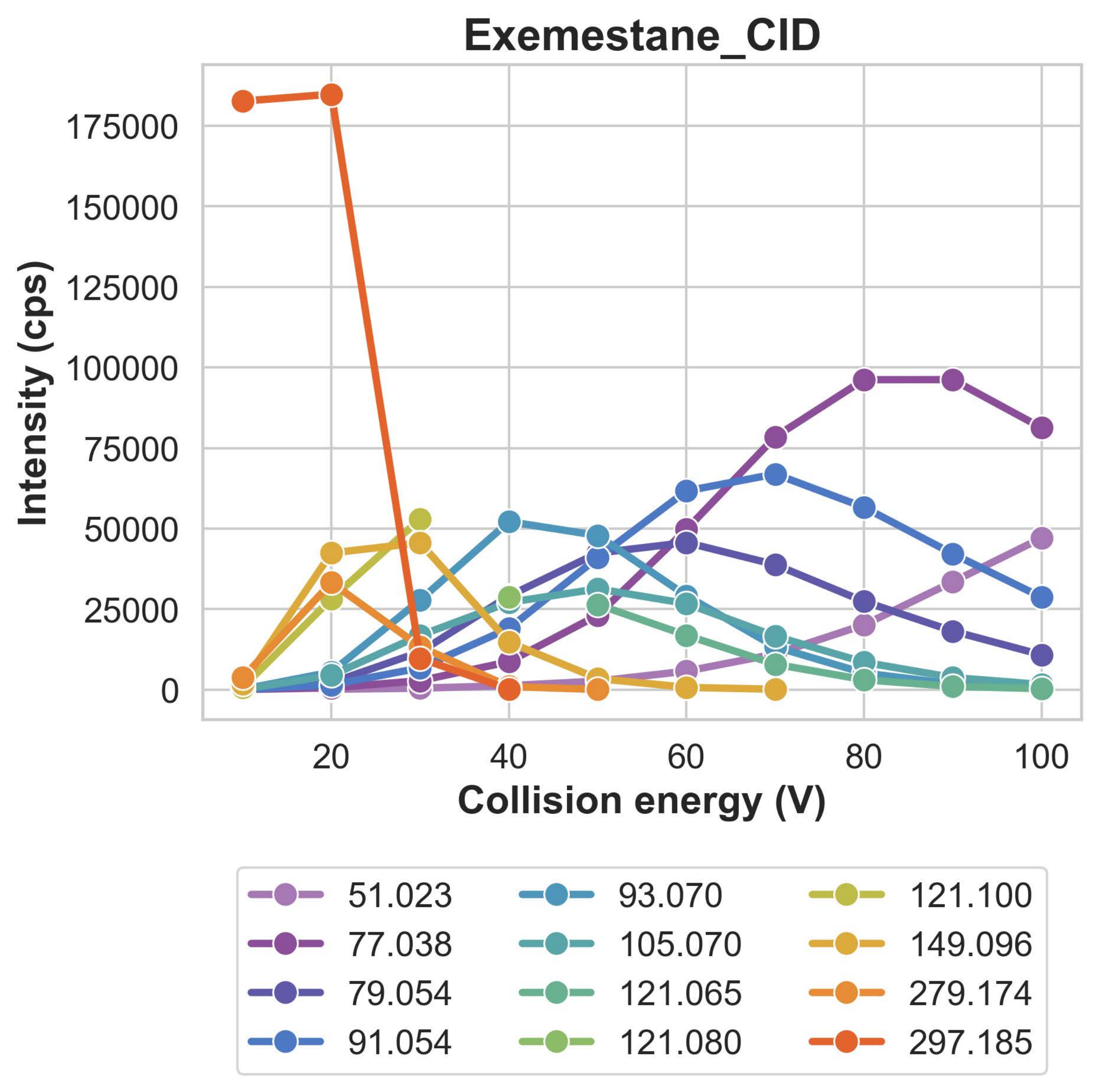

Exemestane_CID
Intensity (cps)
175000
150000
125000
100000
75000
50000
25000
0
20
40
60
80
100
Collision energy (V)
51.023
77.038
79.054
91.054
93.070
105.070
121.065
121.080
121.100
149.096
279.174
297.185

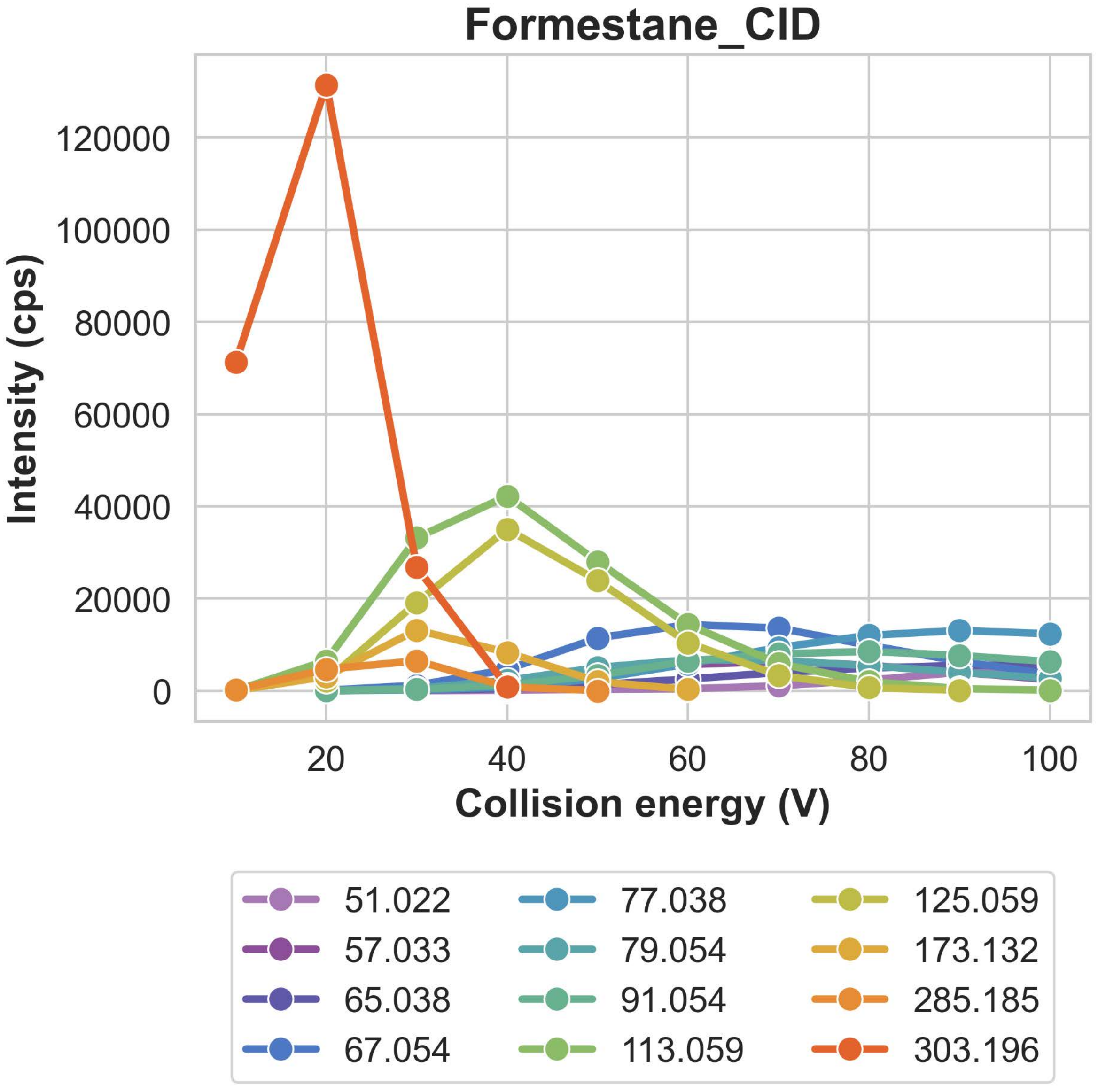

Formestane_CID
Intensity (cps)
0
20000
40000
60000
80000
100000
120000
20
40
60
80
100
Collision energy (V)
51.022
57.033
65.038
67.054
77.038
79.054
91.054
113.059
125.059
173.132
285.185
303.196

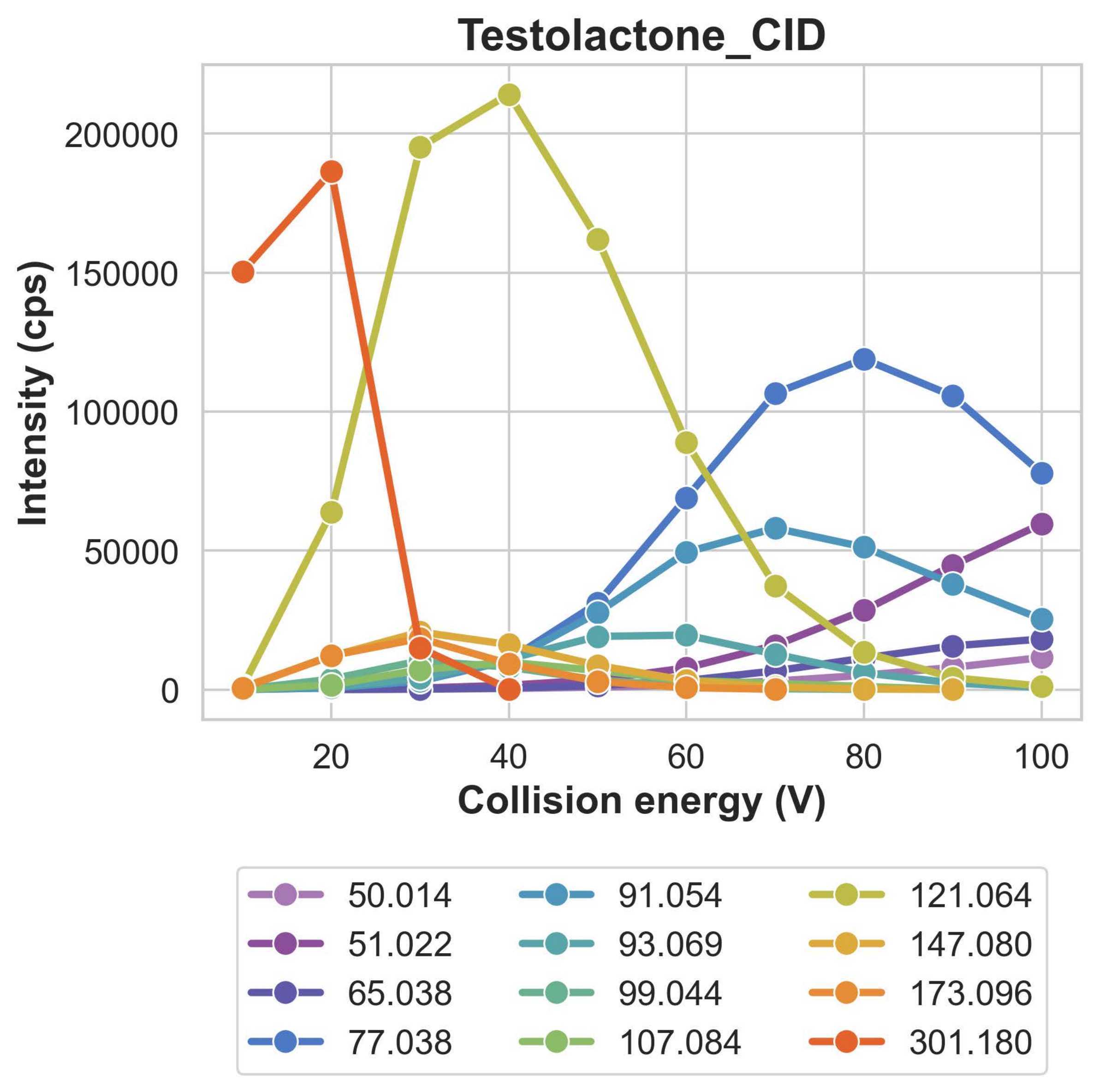
Testolactone_CID
Intensity (cps)
200000
150000
100000
50000
0
20
40
60
80
100
Collision energy (V)
50.014
51.022
65.038
77.038
91.054
93.069
99.044
107.084
121.064
147.080
173.096
301.180

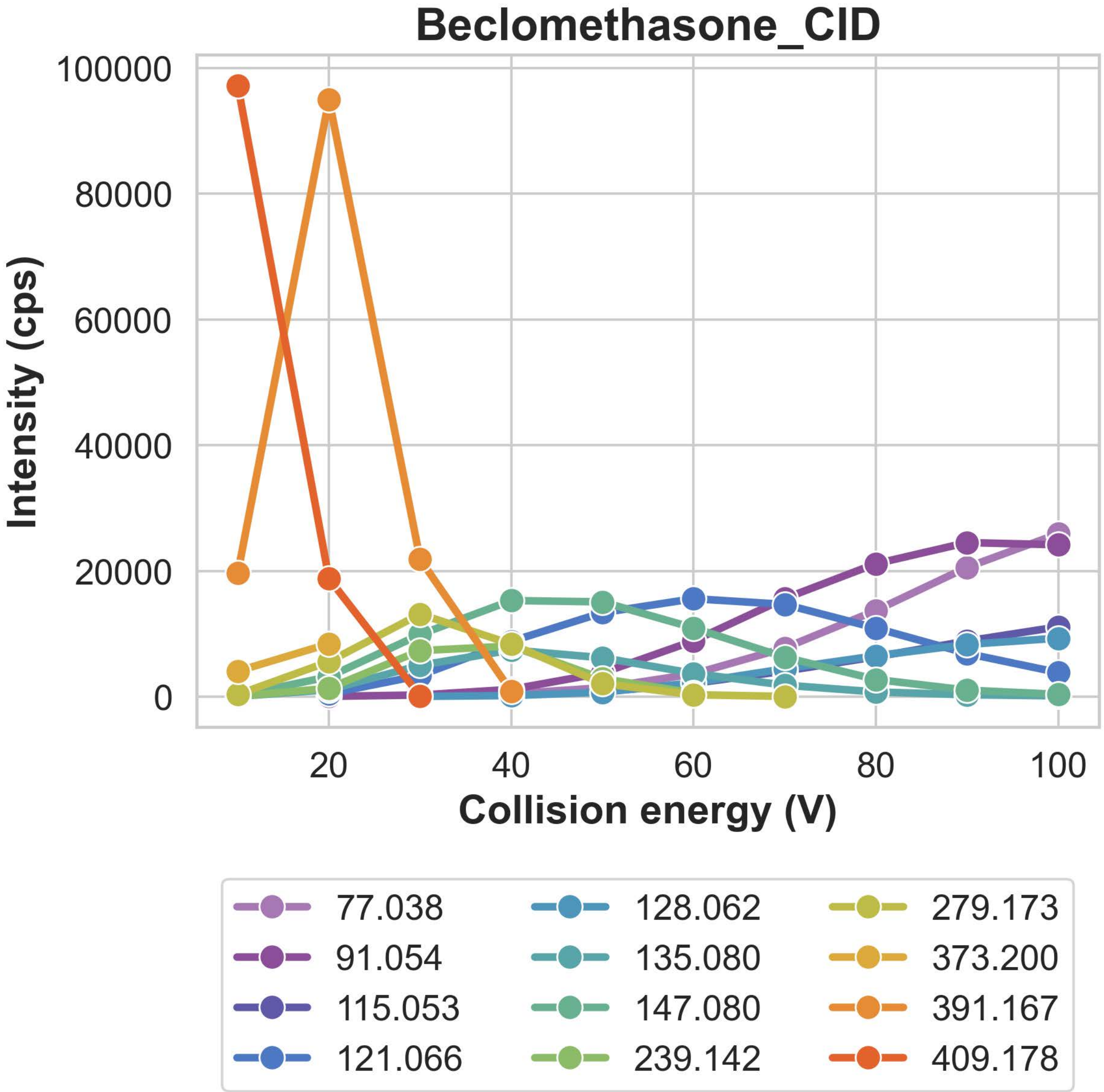
Beclomethasone_CID
Intensity (cps)
100000
80000
60000
40000
20000
0
20
40
60
80
100
Collision energy (V)
77.038
91.054
115.053
121.066
128.062
135.080
147.080
239.142
279.173
373.200
391.167
409.178

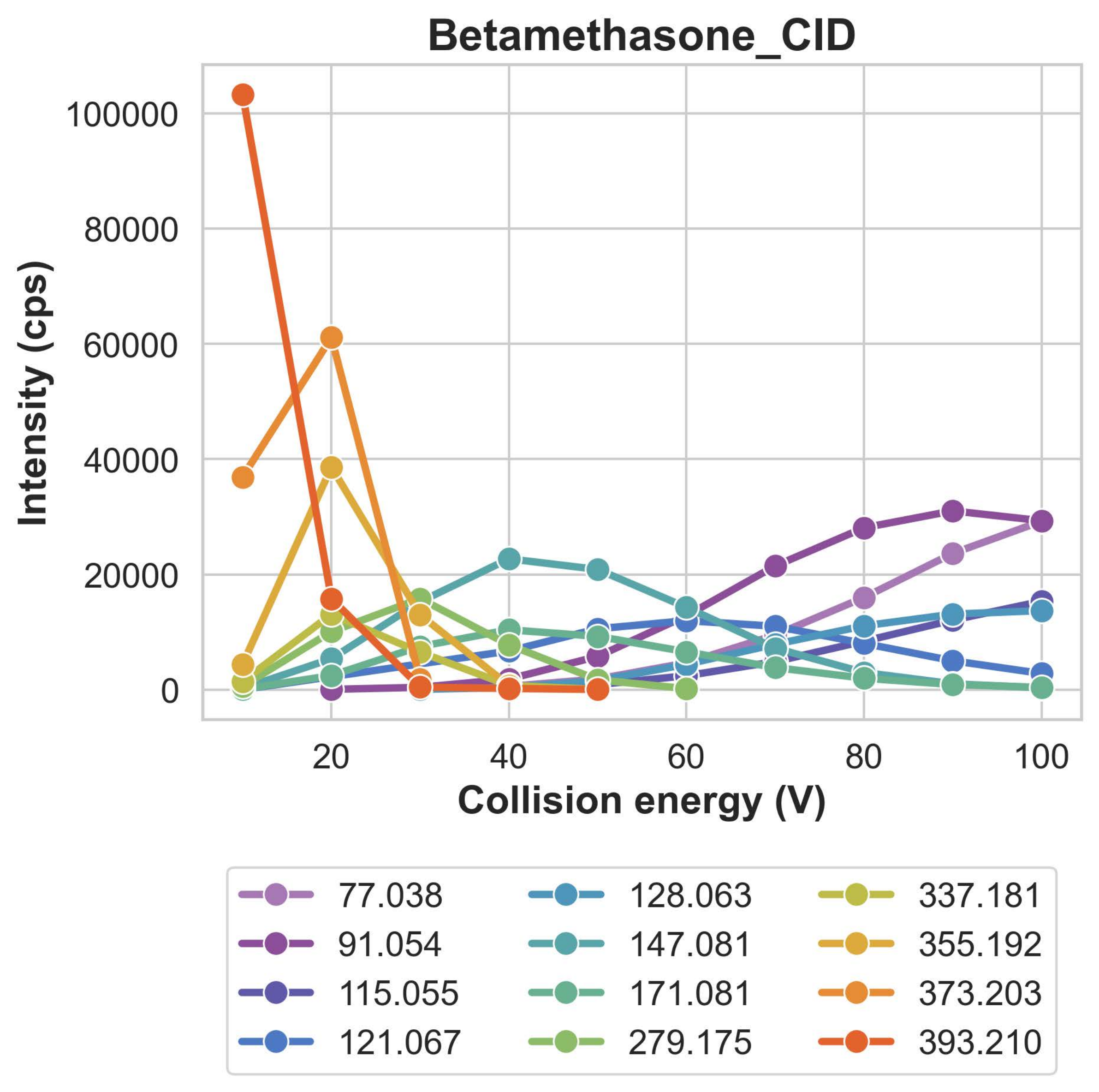

Betamethasone_CID
Intensity (cps)
Collision energy (V)
0
20000
40000
60000
80000
100000
20
40
60
80
100
77.038
91.054
115.055
121.067
128.063
147.081
171.081
279.175
337.181
355.192
373.203
393.210

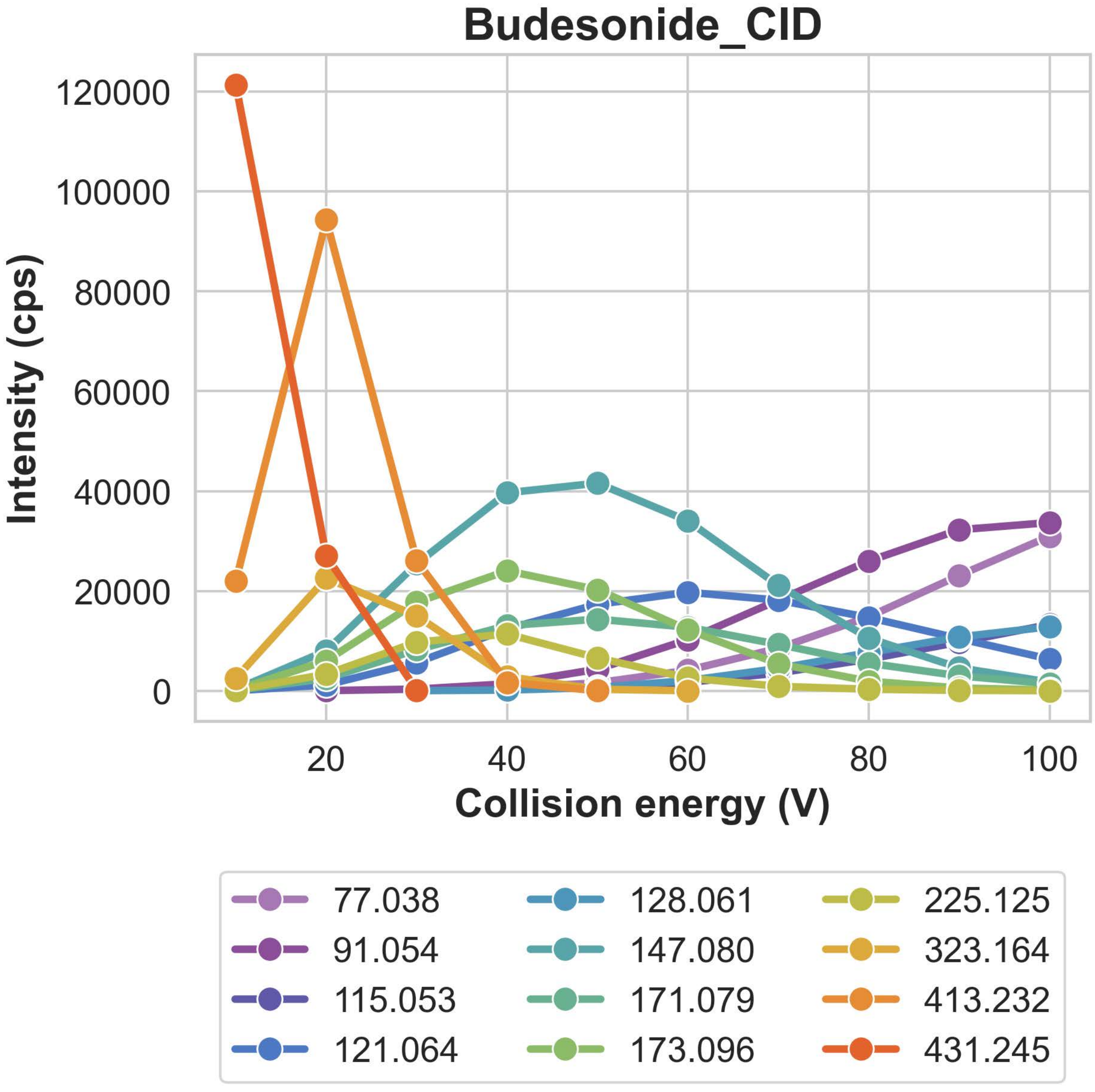
Budesonide_CID
Intensity (cps)
120000
100000
80000
60000
40000
20000
0
20
40
60
80
100
Collision energy (V)
77.038
91.054
115.053
121.064
128.061
147.080
171.079
173.096
225.125
323.164
413.232
431.245

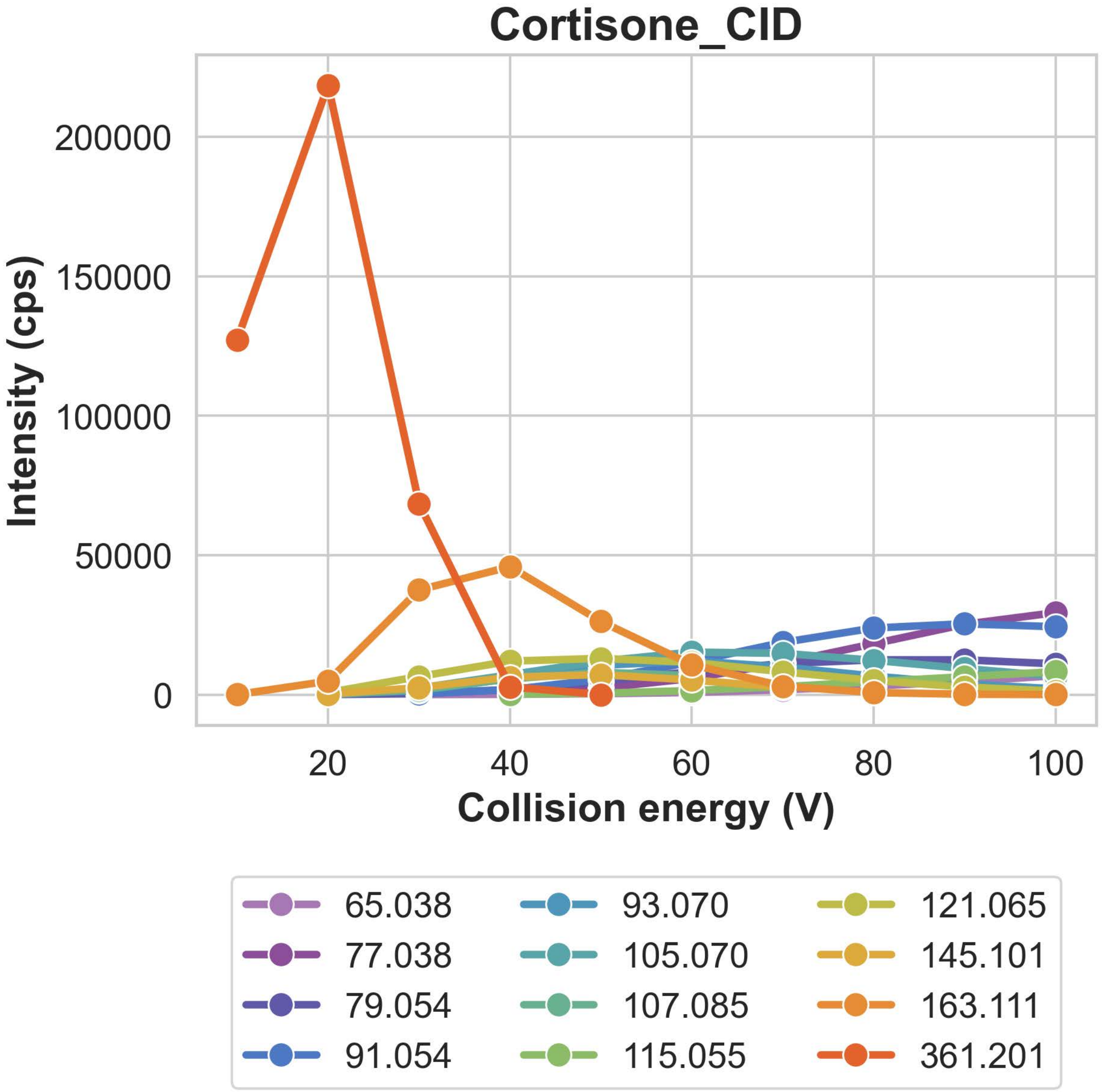
Cortisone_CID
Intensity (cps)
200000
150000
100000
50000
0
20
40
60
80
100
Collision energy (V)
65.038
77.038
79.054
91.054
93.070
105.070
107.085
115.055
121.065
145.101
163.111
361.201

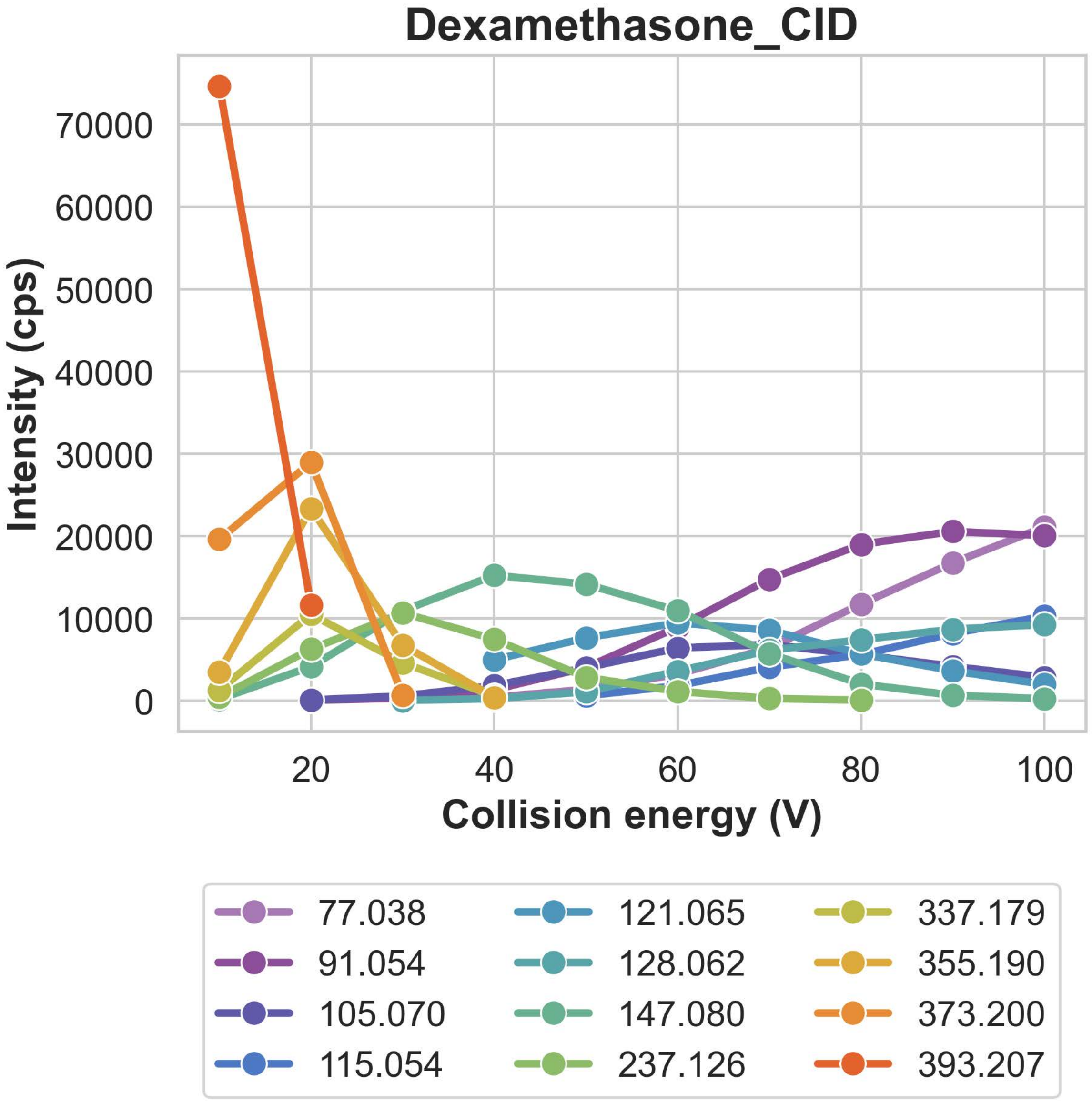

Dexamethasone_CID
Intensity (cps)
70000
60000
50000
40000
30000
20000
10000
0
20
40
60
80
100
Collision energy (V)
77.038
91.054
105.070
115.054
121.065
128.062
147.080
237.126
337.179
355.190
373.200
393.207

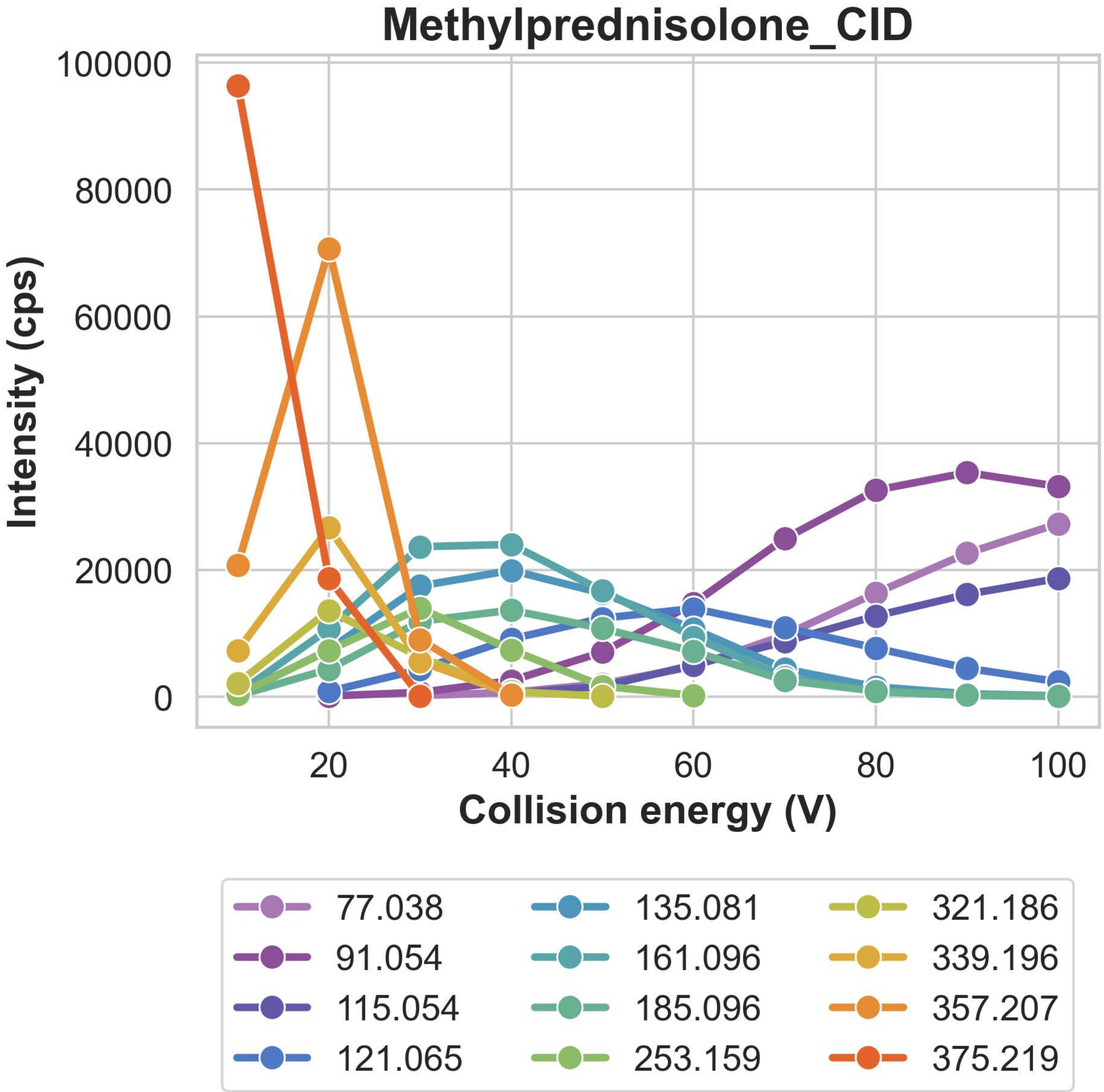
Methylprednisolone_CID
Intensity (cps)
100000
80000
60000
40000
20000
0
20
40
60
80
100
Collision energy (V)
77.038
91.054
115.054
121.065
135.081
161.096
185.096
253.159
321.186
339.196
357.207
375.219

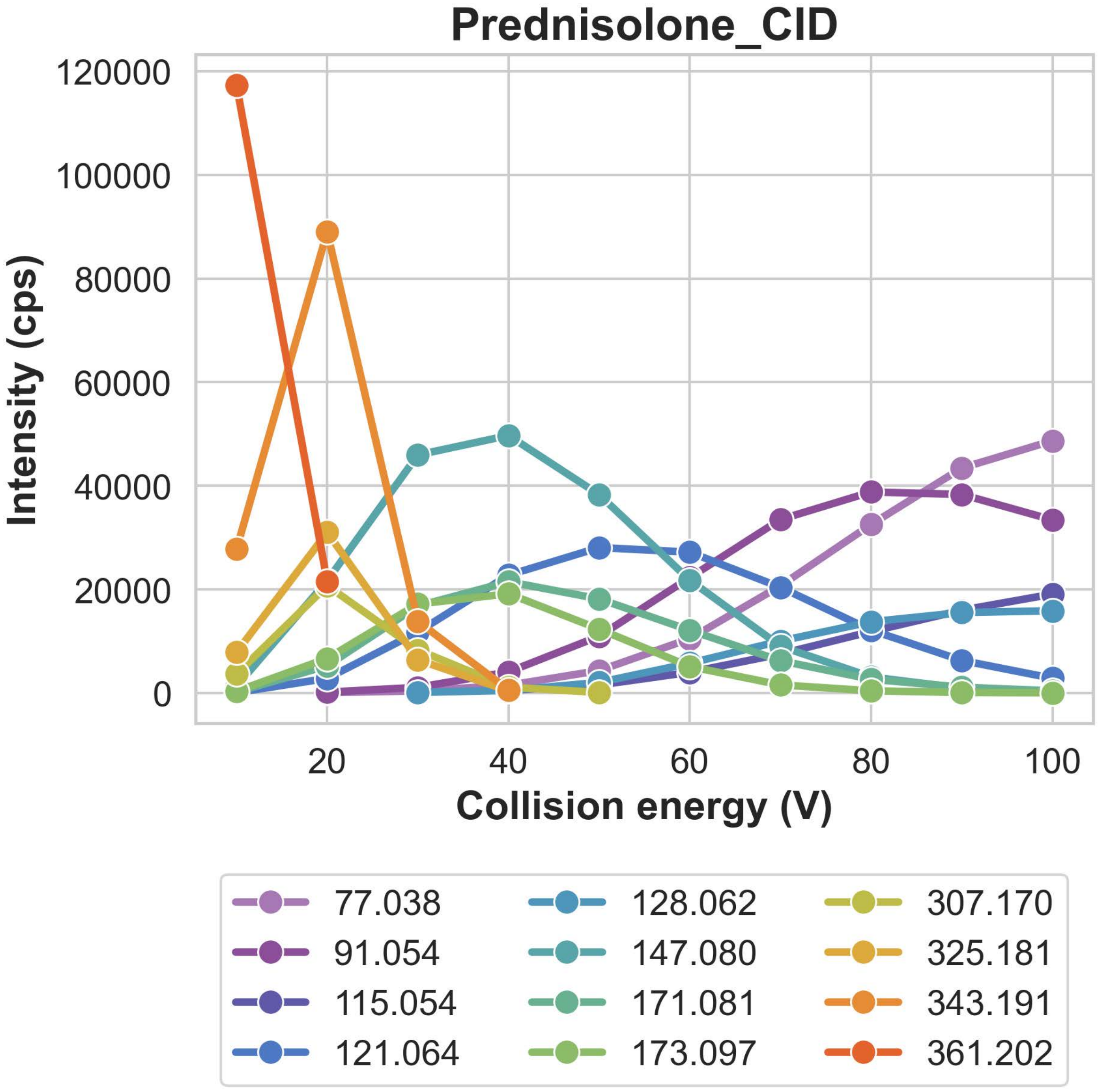
Prednisolone_CID
Intensity (cps)
120000
100000
80000
60000
40000
20000
0
20
40
60
80
100
Collision energy (V)
77.038
91.054
115.054
121.064
128.062
147.080
171.081
173.097
307.170
325.181
343.191
361.202

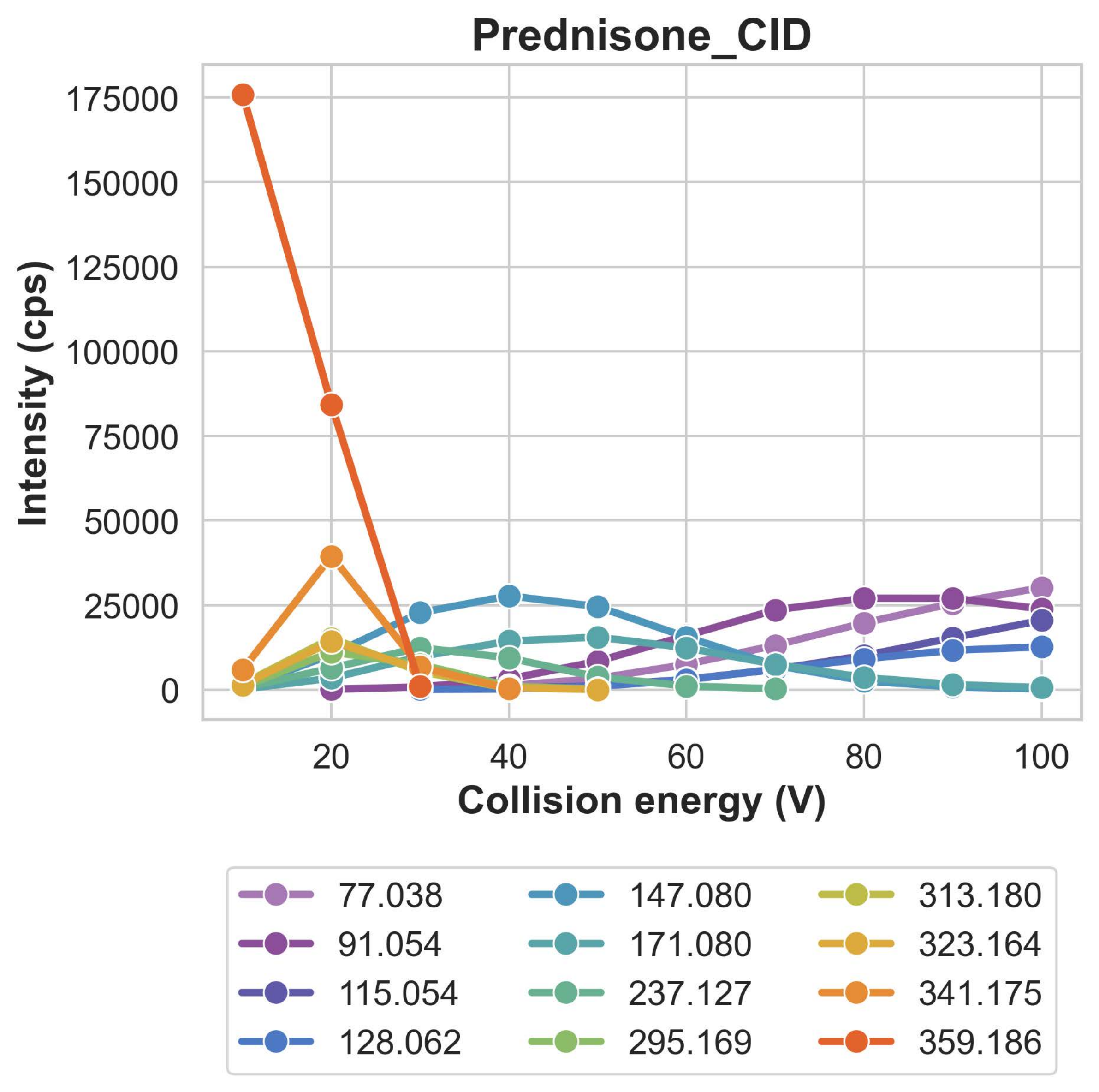
Prednisone_CID
Intensity (cps)
175000
150000
125000
100000
75000
50000
25000
0
20
40
60
80
100
Collision energy (V)
77.038
91.054
115.054
128.062
147.080
171.080
237.127
295.169
313.180
323.164
341.175
359.186

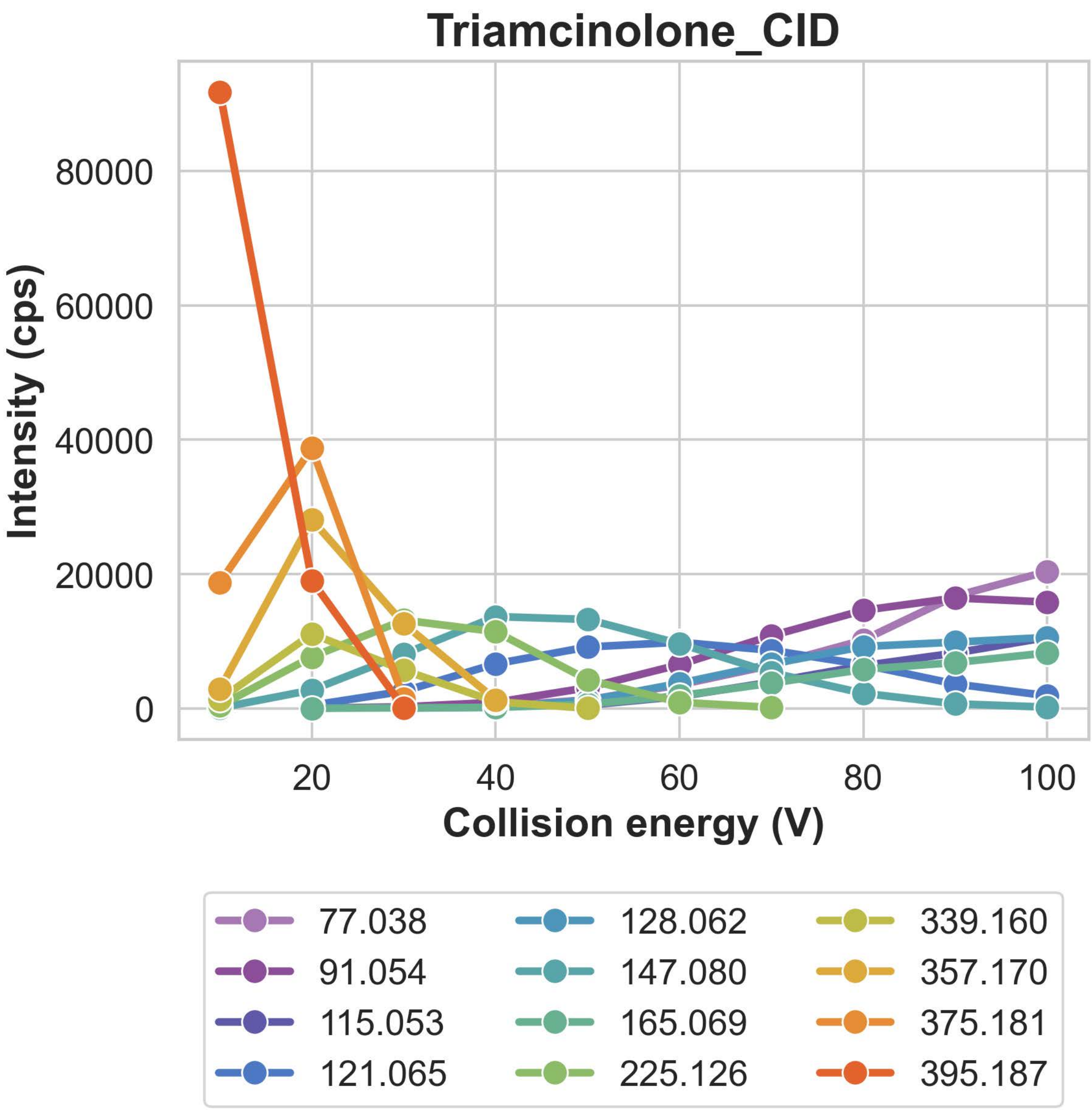

Triamcinolone_CID
Intensity (cps)
80000
60000
40000
20000
0
20
40
60
80
100
Collision energy (V)
77.038
91.054
115.053
121.065
128.062
147.080
165.069
225.126
339.160
357.170
375.181
395.187

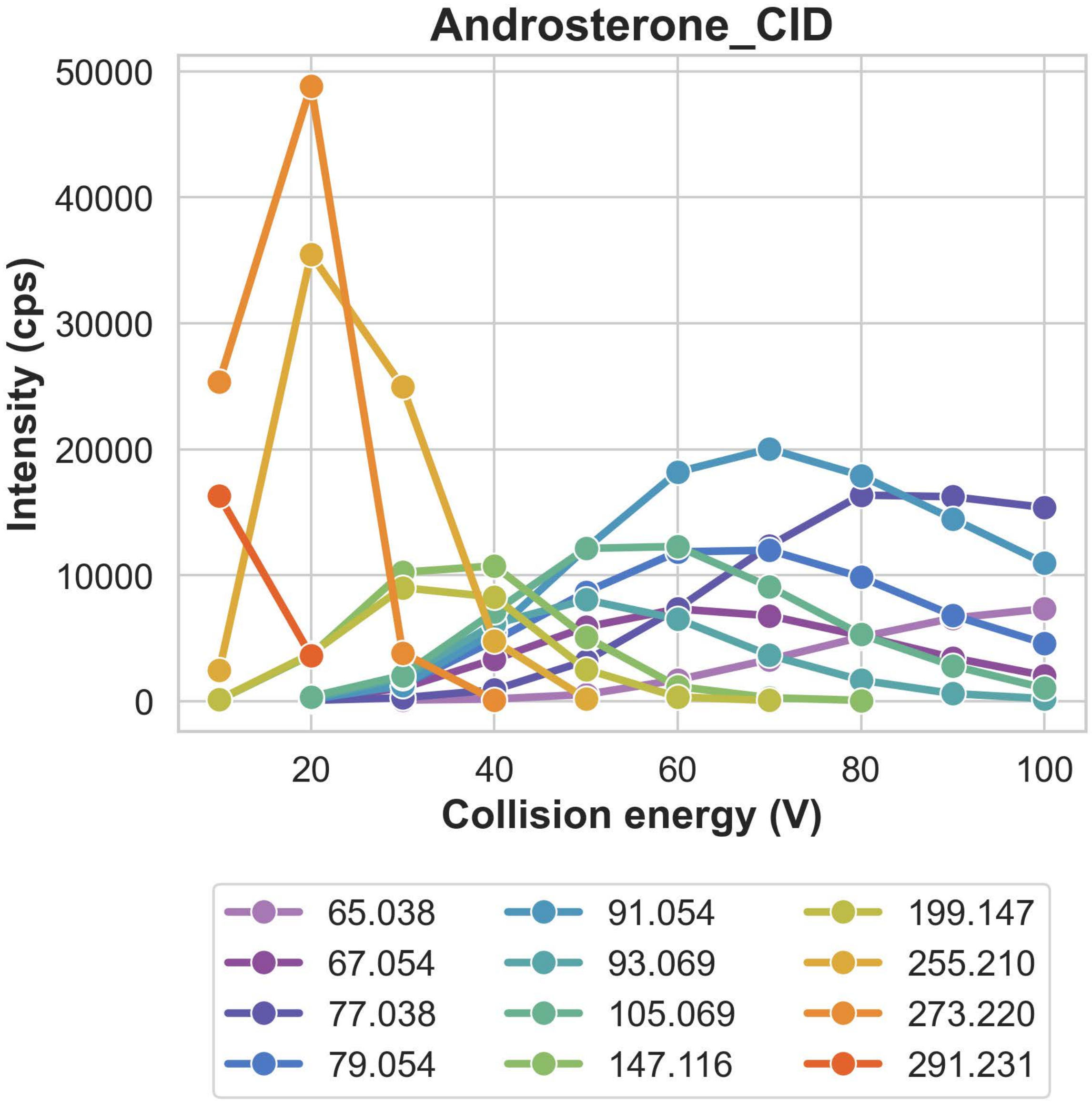

Androsterone_CID
Intensity (cps)
50000
40000
30000
20000
10000
0
20
40
60
80
100
Collision energy (V)
65.038
67.054
77.038
79.054
91.054
93.069
105.069
147.116
199.147
255.210
273.220
291.231

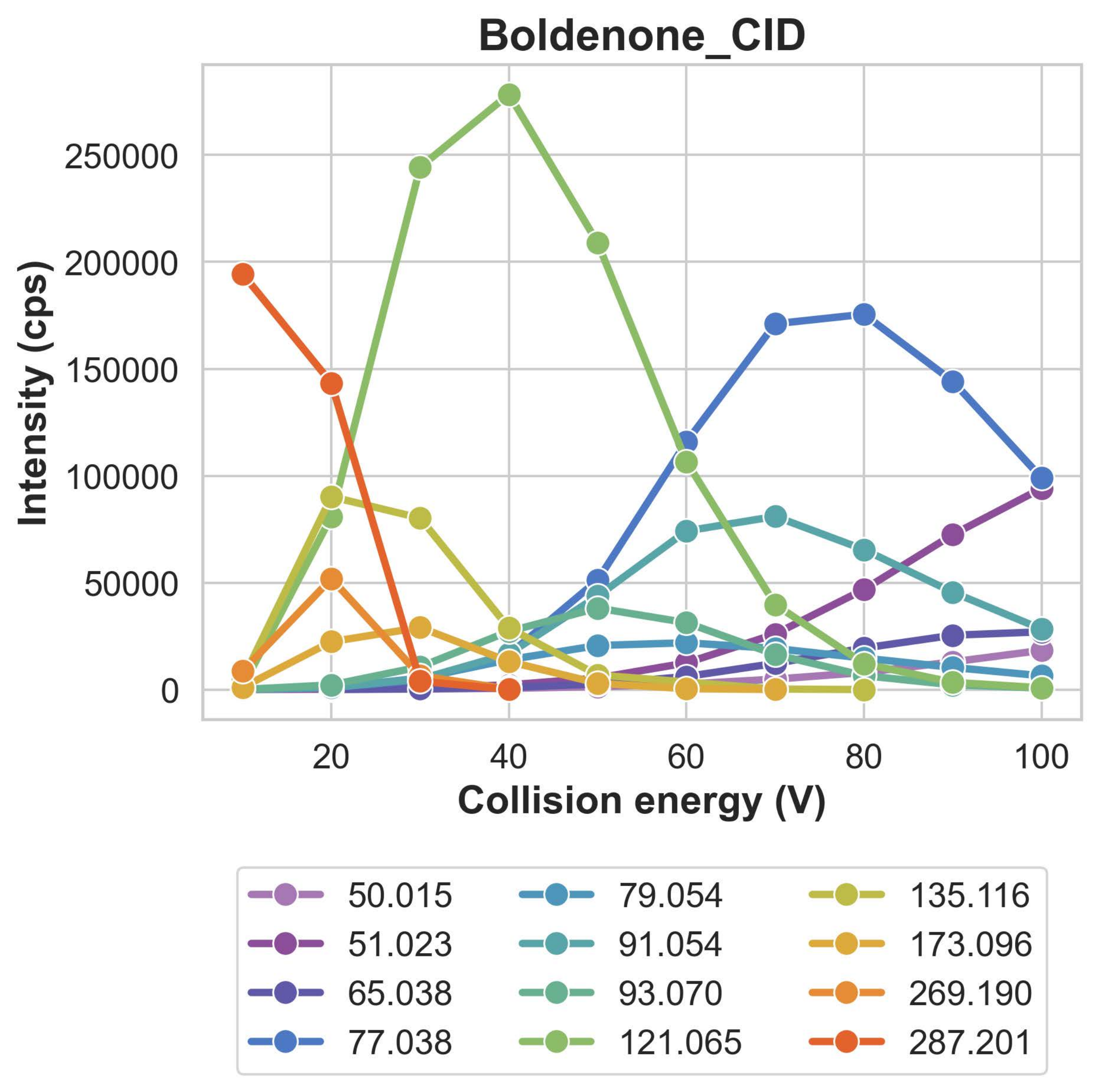

Boldenone_CID
Intensity (cps)
250000
200000
150000
100000
50000
0
20
40
60
80
100
Collision energy (V)
50.015
51.023
65.038
77.038
79.054
91.054
93.070
121.065
135.116
173.096
269.190
287.201

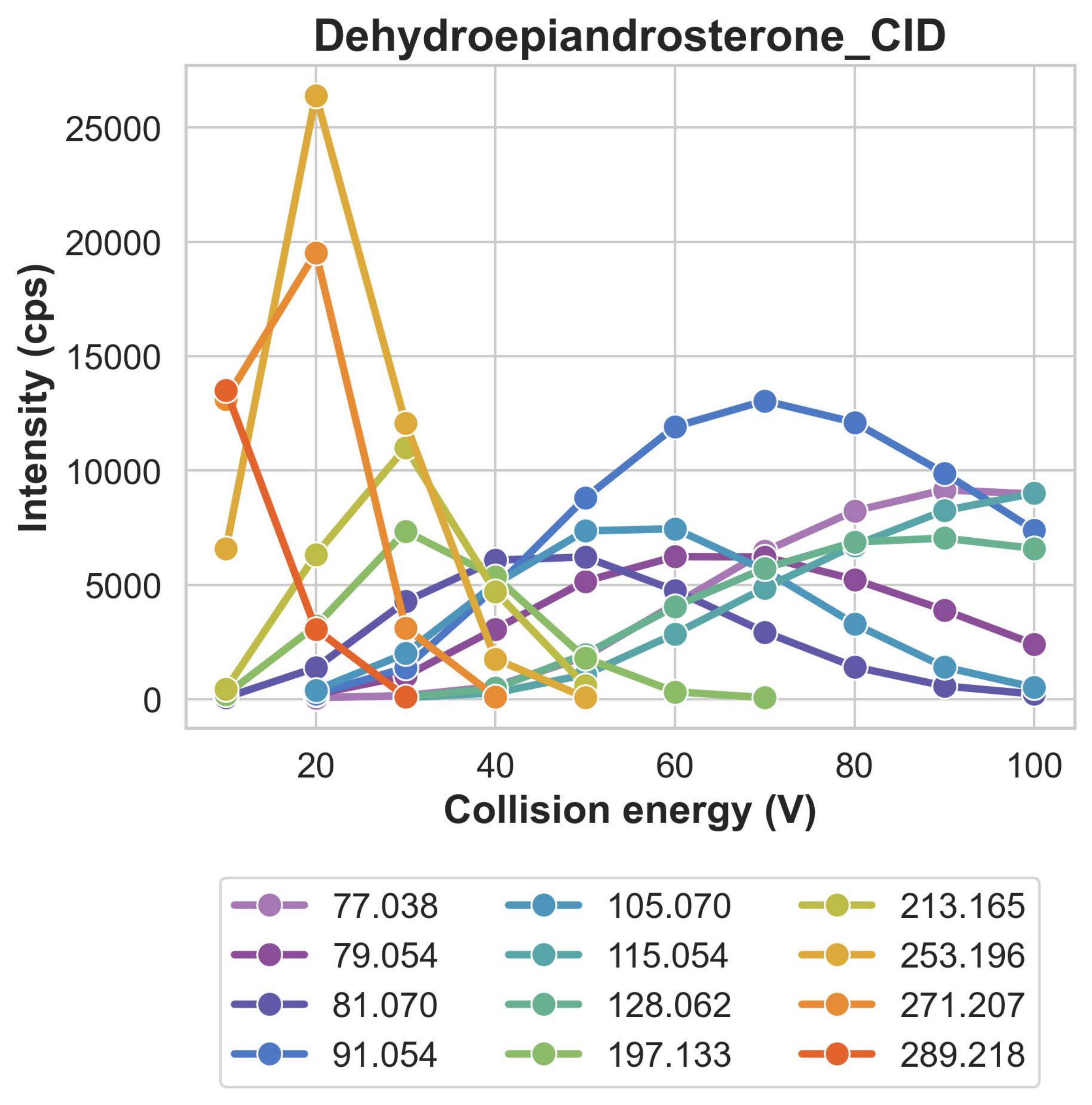

Dehydroepiandrosterone_CID
Intensity (cps)
25000
20000
15000
10000
5000
0
20
40
60
80
100
Collision energy (V)
77.038
79.054
81.070
91.054
105.070
115.054
128.062
197.133
213.165
253.196
271.207
289.218

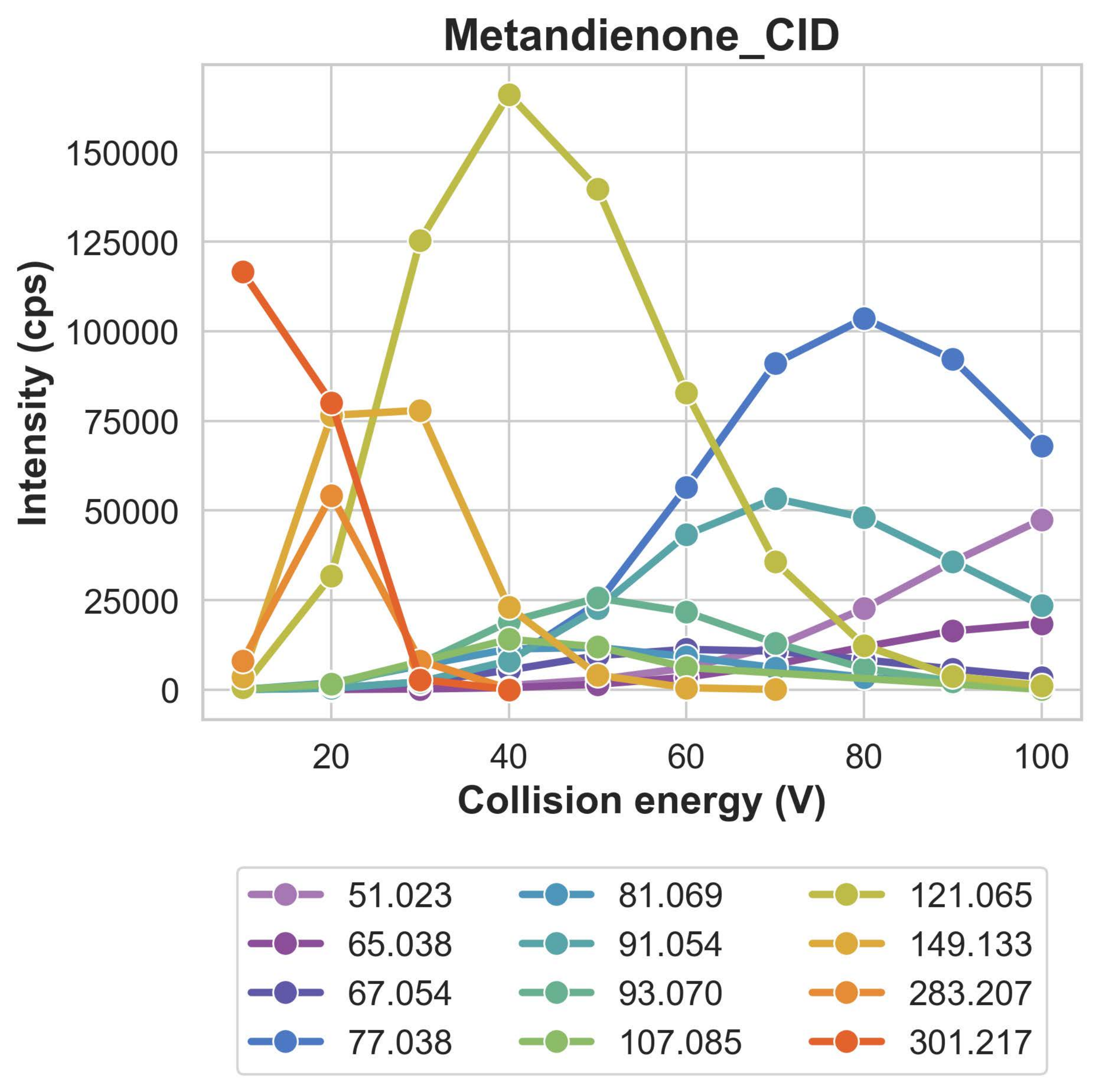

Metandienone_CID
Intensity (cps)
Collision energy (V)
150000
125000
100000
75000
50000
25000
0
20
40
60
80
100
51.023
65.038
67.054
77.038
81.069
91.054
93.070
107.085
121.065
149.133
283.207
301.217

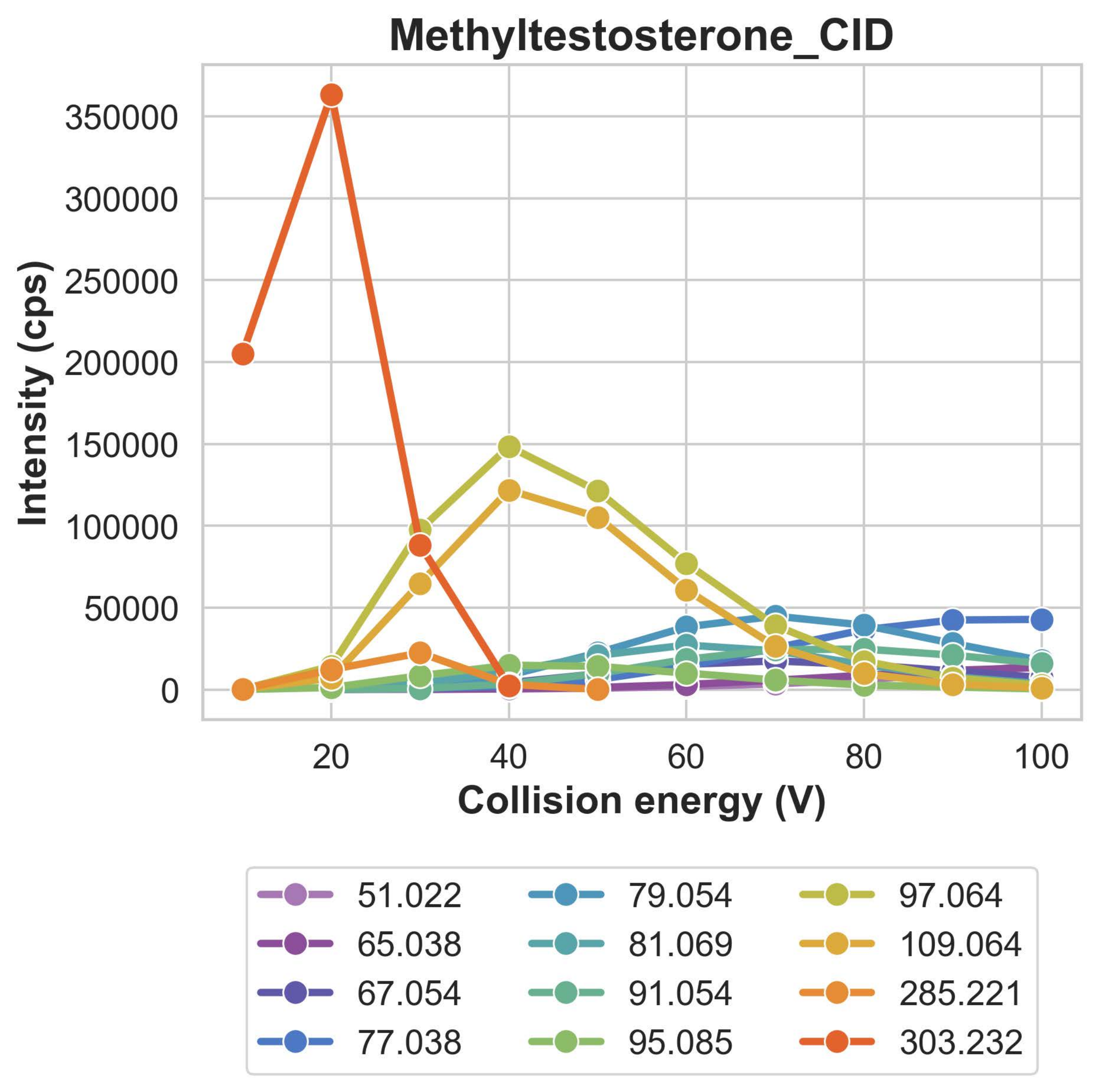
Methyltestosterone_CID
Intensity (cps)
350000
300000
250000
200000
150000
100000
50000
0
20
40
60
80
100
Collision energy (V)
51.022
65.038
67.054
77.038
79.054
81.069
91.054
95.085
97.064
109.064
285.221
303.232

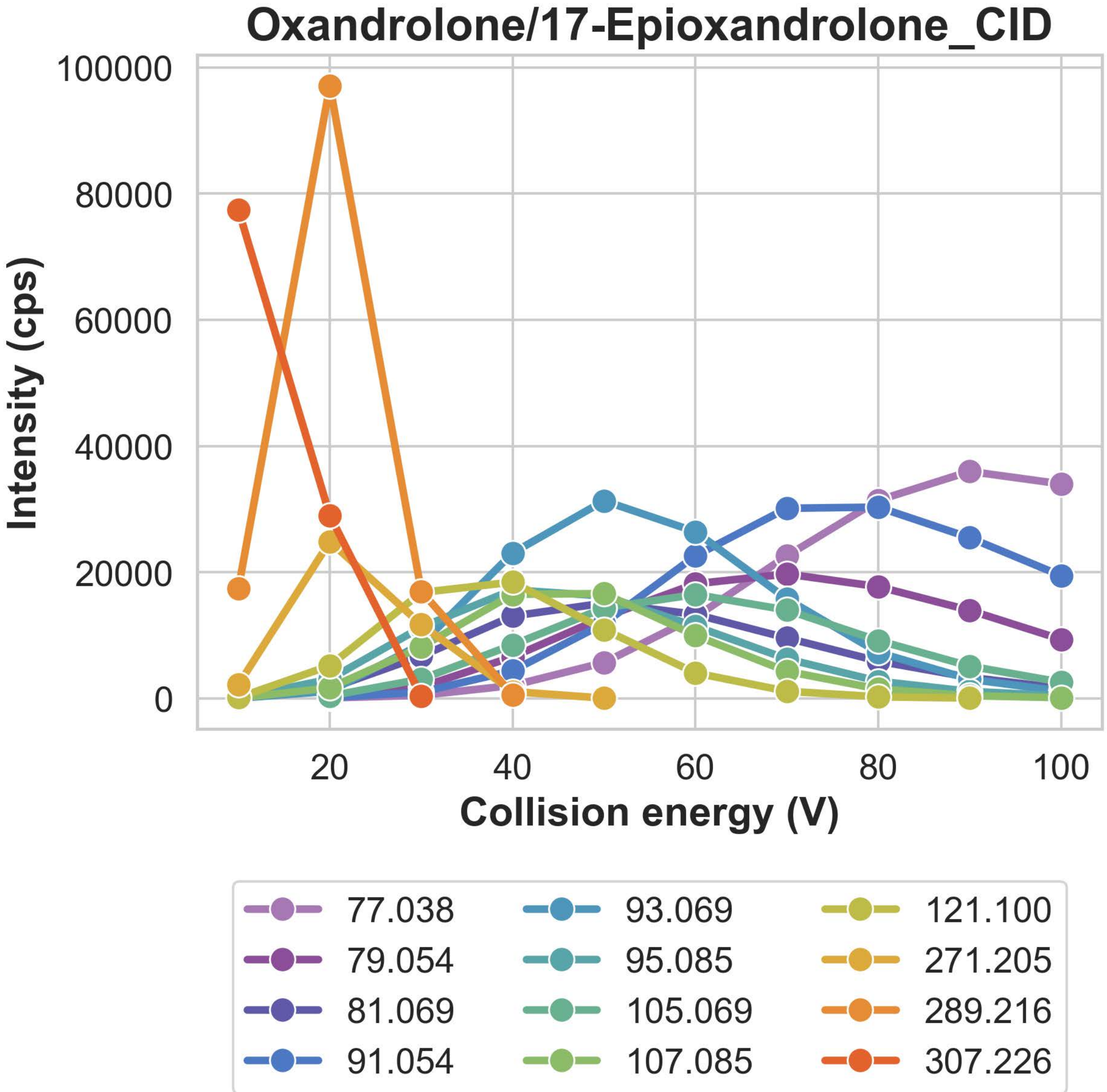

Oxandrolone/17-Epioxandrolone_CID
Intensity (cps)
100000
80000
60000
40000
20000
0
20
40
60
80
100
Collision energy (V)
77.038
79.054
81.069
91.054
93.069
95.085
105.069
107.085
121.100
271.205
289.216
307.226

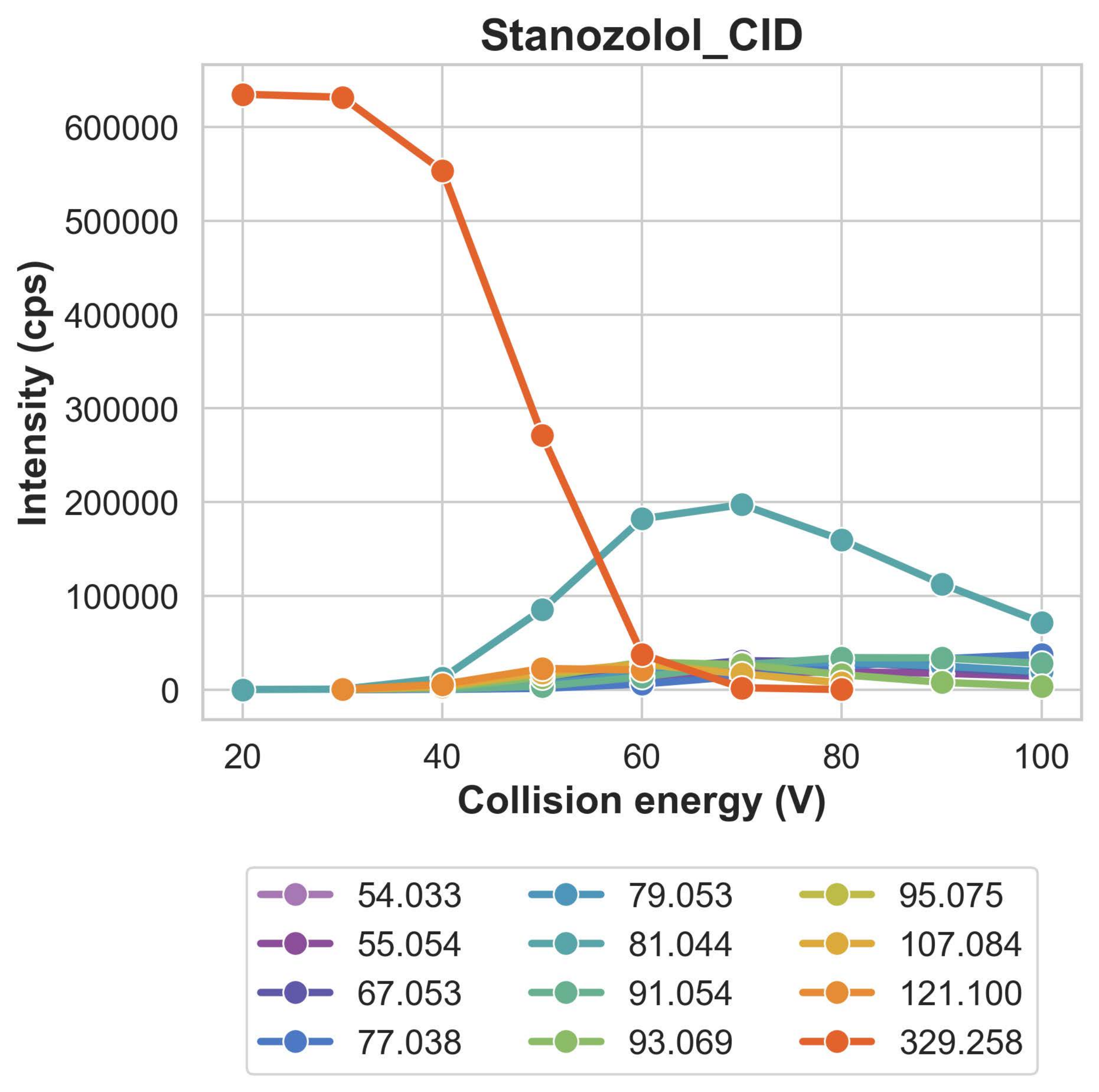
Stanozolol_CID
Intensity (cps)
600000
500000
400000
300000
200000
100000
0
20
40
60
80
100
Collision energy (V)
54.033
55.054
67.053
77.038
79.053
81.044
91.054
93.069
95.075
107.084
121.100
329.258

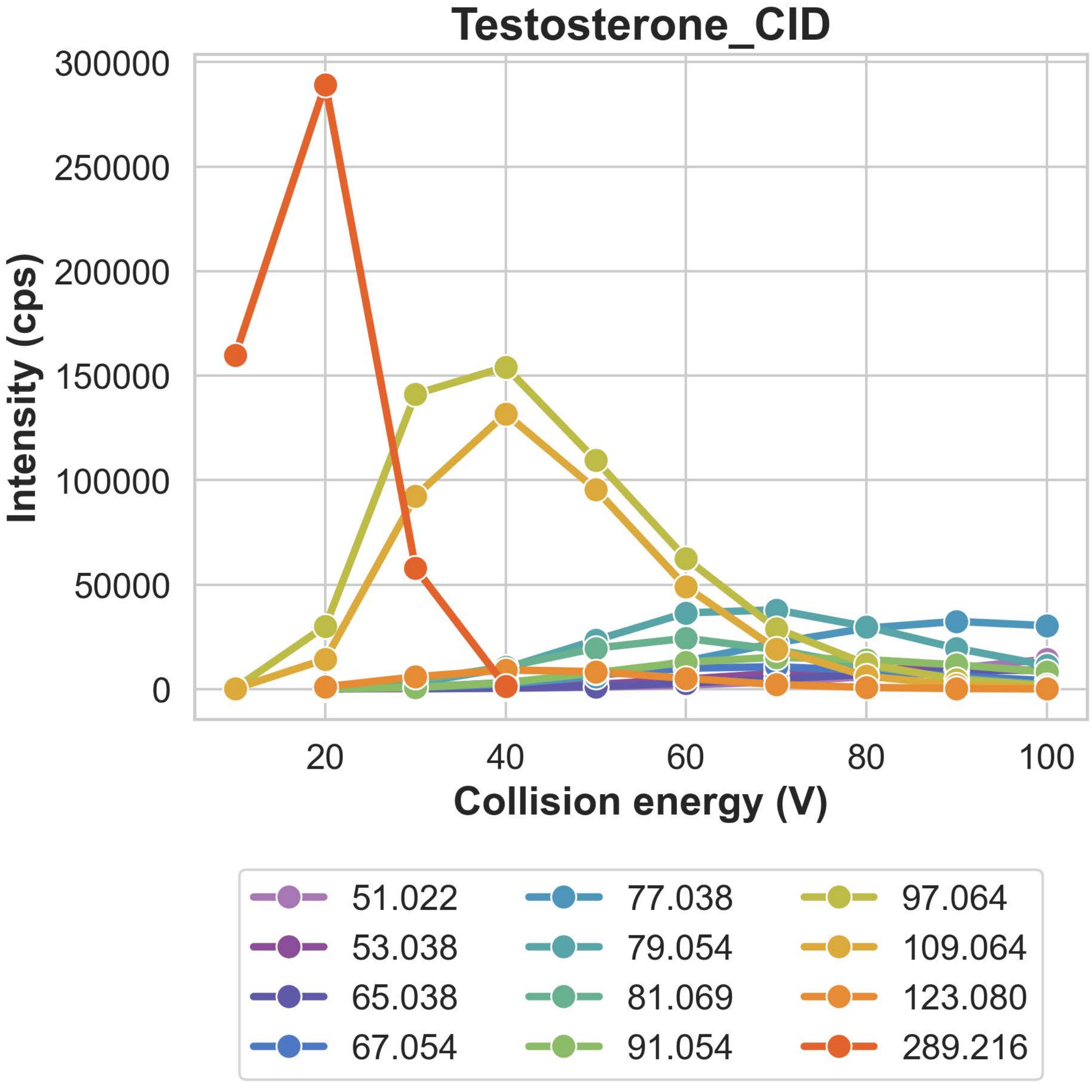
Testosterone_CID
Intensity (cps)
300000
250000
200000
150000
100000
50000
0
20
40
60
80
100
Collision energy (V)
51.022
53.038
65.038
67.054
77.038
79.054
81.069
91.054
97.064
109.064
123.080
289.216

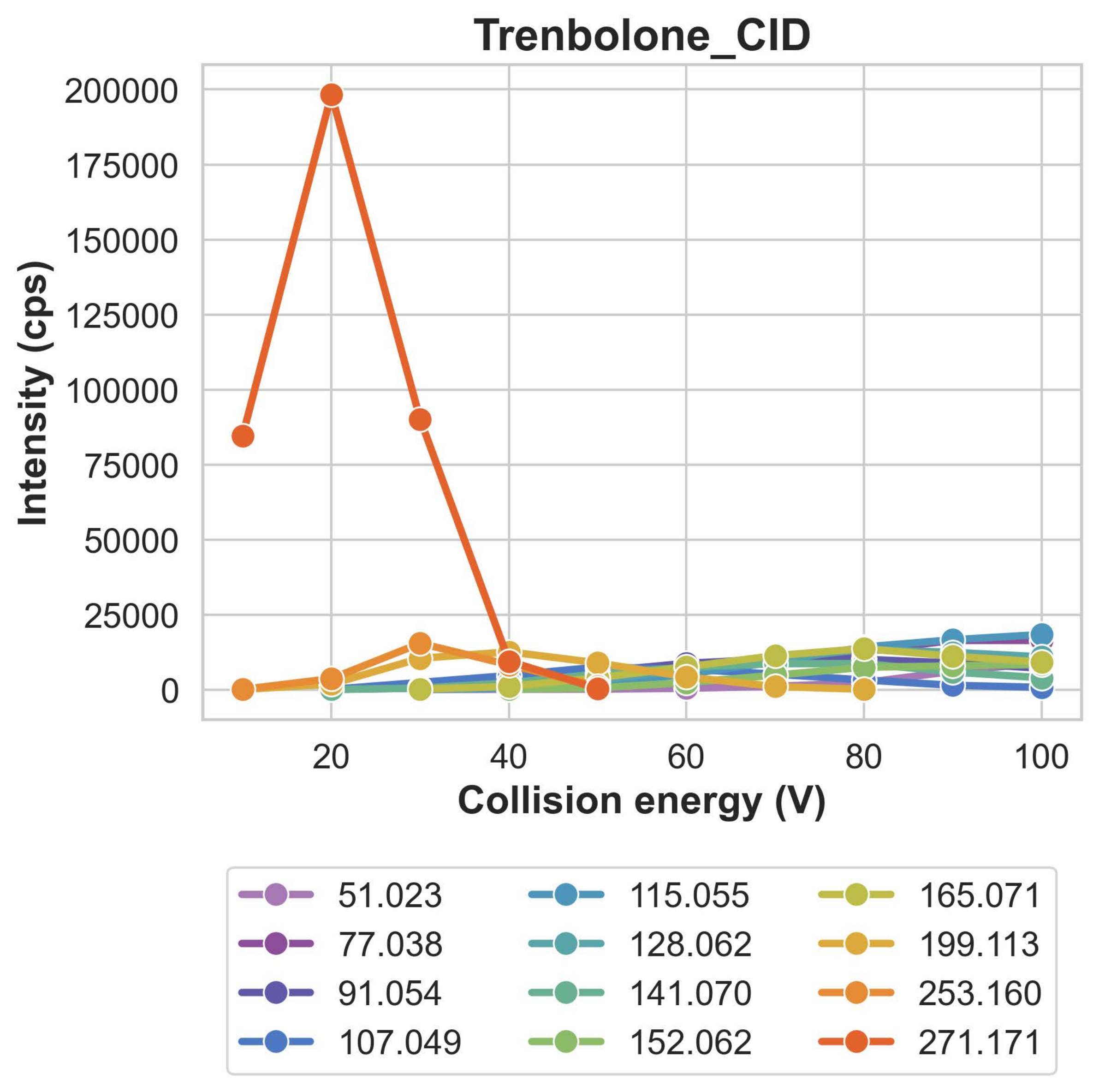

Trenbolone_CID
Intensity (cps)
200000
175000
150000
125000
100000
75000
50000
25000
0
20
40
60
80
100
Collision energy (V)
51.023
77.038
91.054
107.049
115.055
128.062
141.070
152.062
165.071
199.113
253.160
271.171

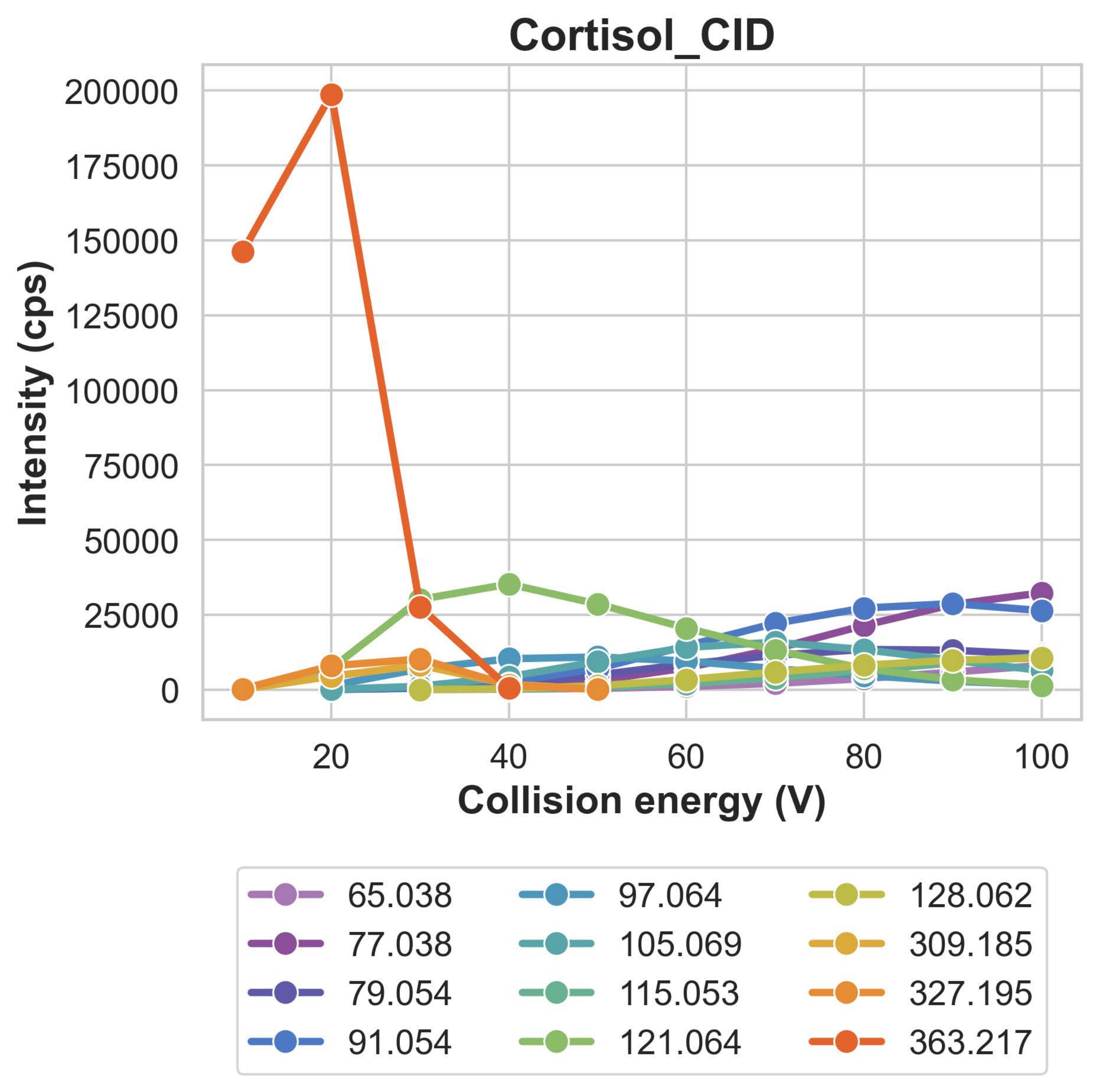

Cortisol_CID
Intensity (cps)
200000
175000
150000
125000
100000
75000
50000
25000
0
20
40
60
80
100
Collision energy (V)
65.038
77.038
79.054
91.054
97.064
105.069
115.053
121.064
128.062
309.185
327.195
363.217

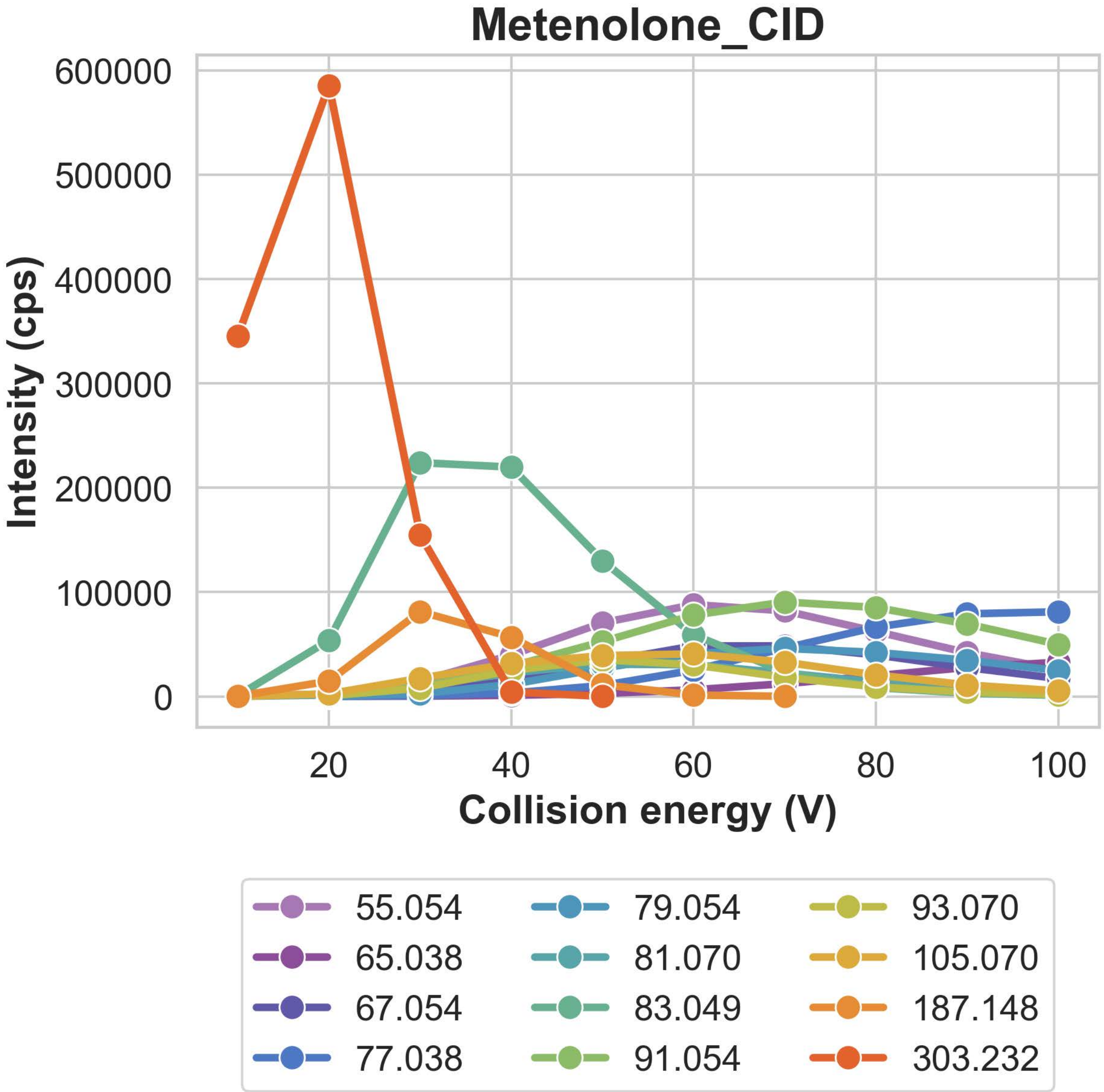

Metenolone_CID
Intensity (cps)
600000
500000
400000
300000
200000
100000
0
20
40
60
80
100
Collision energy (V)
55.054
65.038
67.054
77.038
79.054
81.070
83.049
91.054
93.070
105.070
187.148
303.232

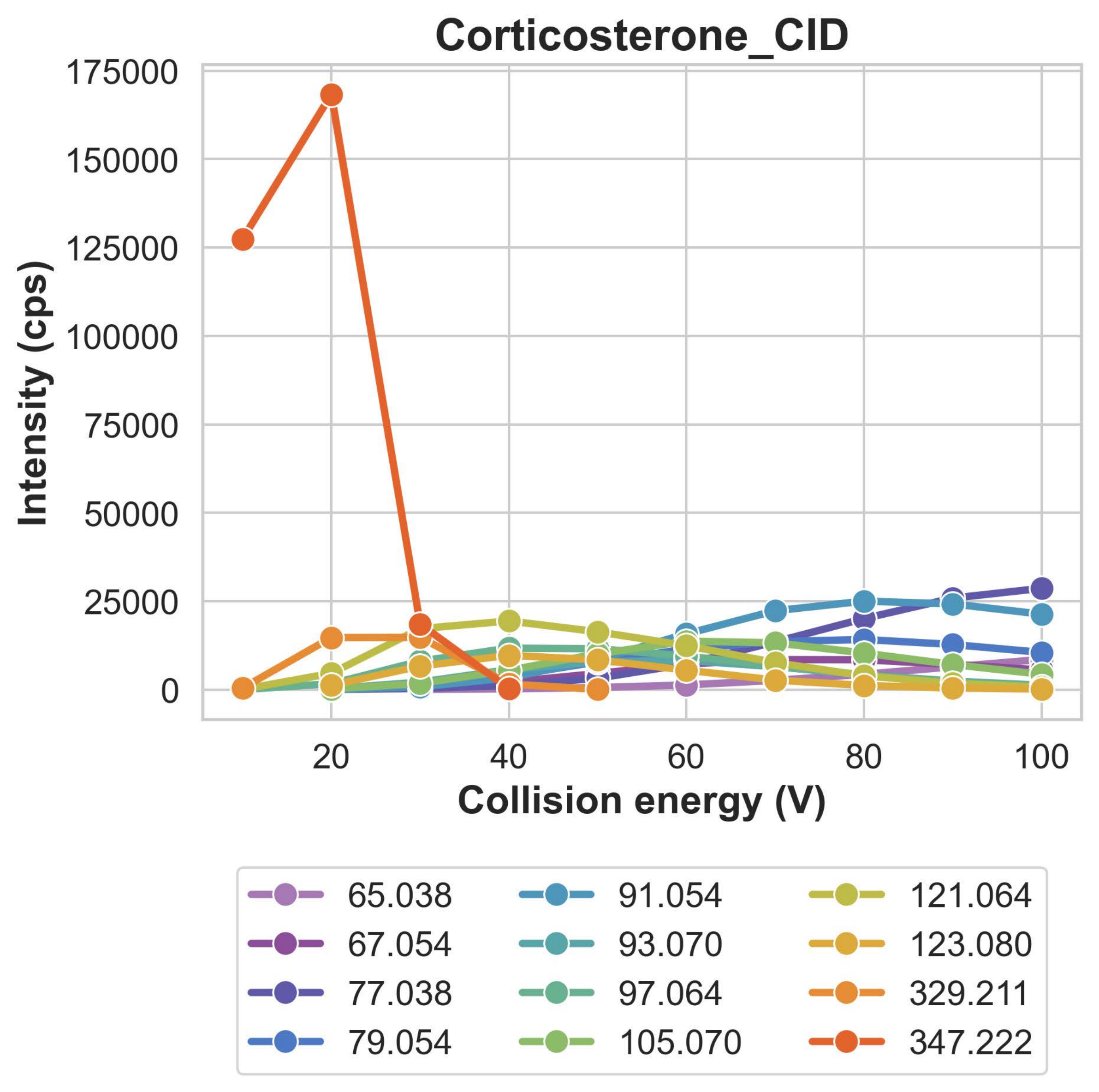
Corticosterone_CID
Intensity (cps)
175000
150000
125000
100000
75000
50000
25000
0
20
40
60
80
100
Collision energy (V)
65.038
67.054
77.038
79.054
91.054
93.070
97.064
105.070
121.064
123.080
329.211
347.222

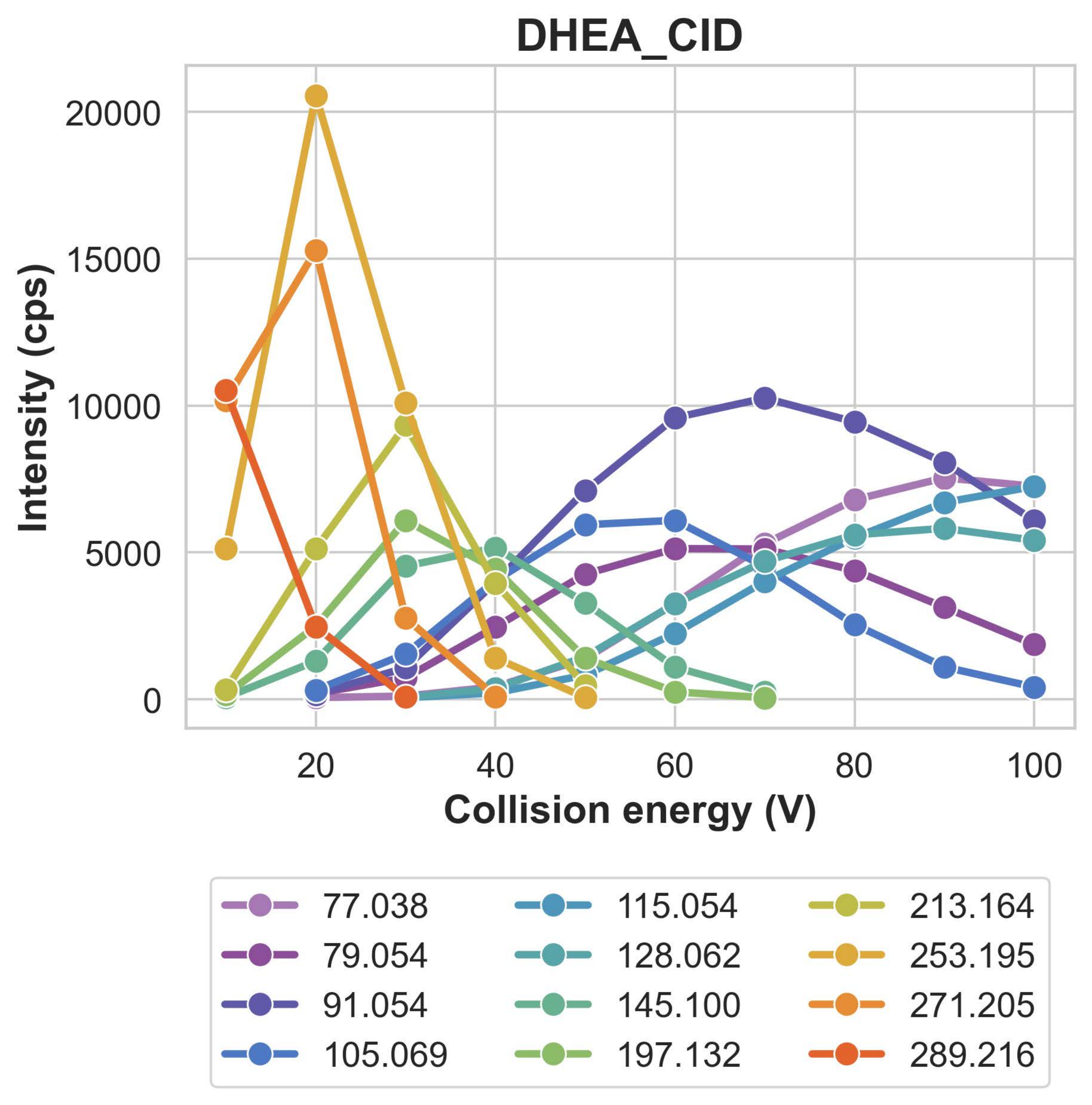
DHEA_CID
Intensity (cps)
20000
15000
10000
5000
0
20
40
60
80
100
Collision energy (V)
77.038
79.054
91.054
105.069
115.054
128.062
145.100
197.132
213.164
253.195
271.205
289.216

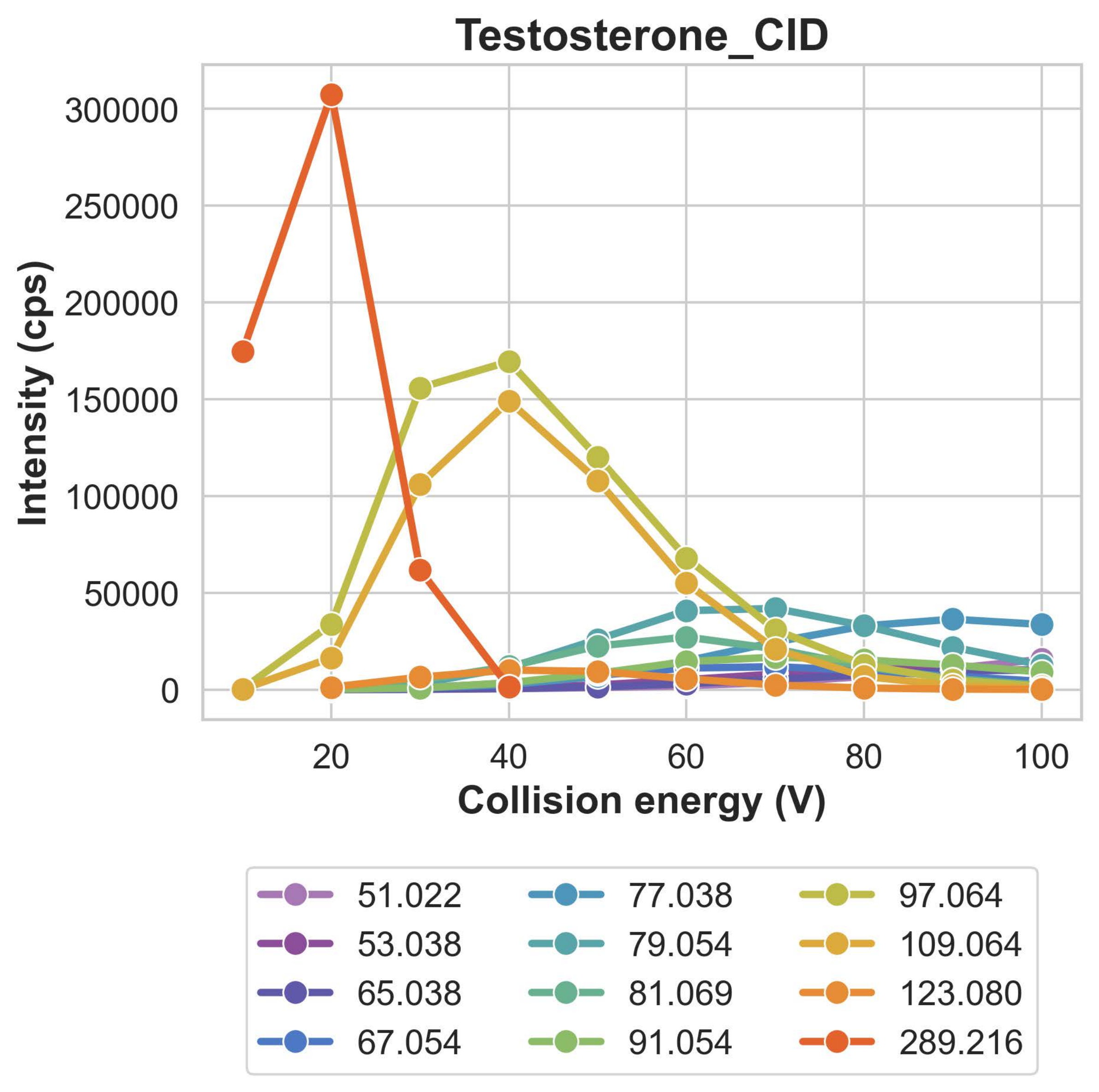

Testosterone_CID
Intensity (cps)
300000
250000
200000
150000
100000
50000
0
20
40
60
80
100
Collision energy (V)
51.022
53.038
65.038
67.054
77.038
79.054
81.069
91.054
97.064
109.064
123.080
289.216

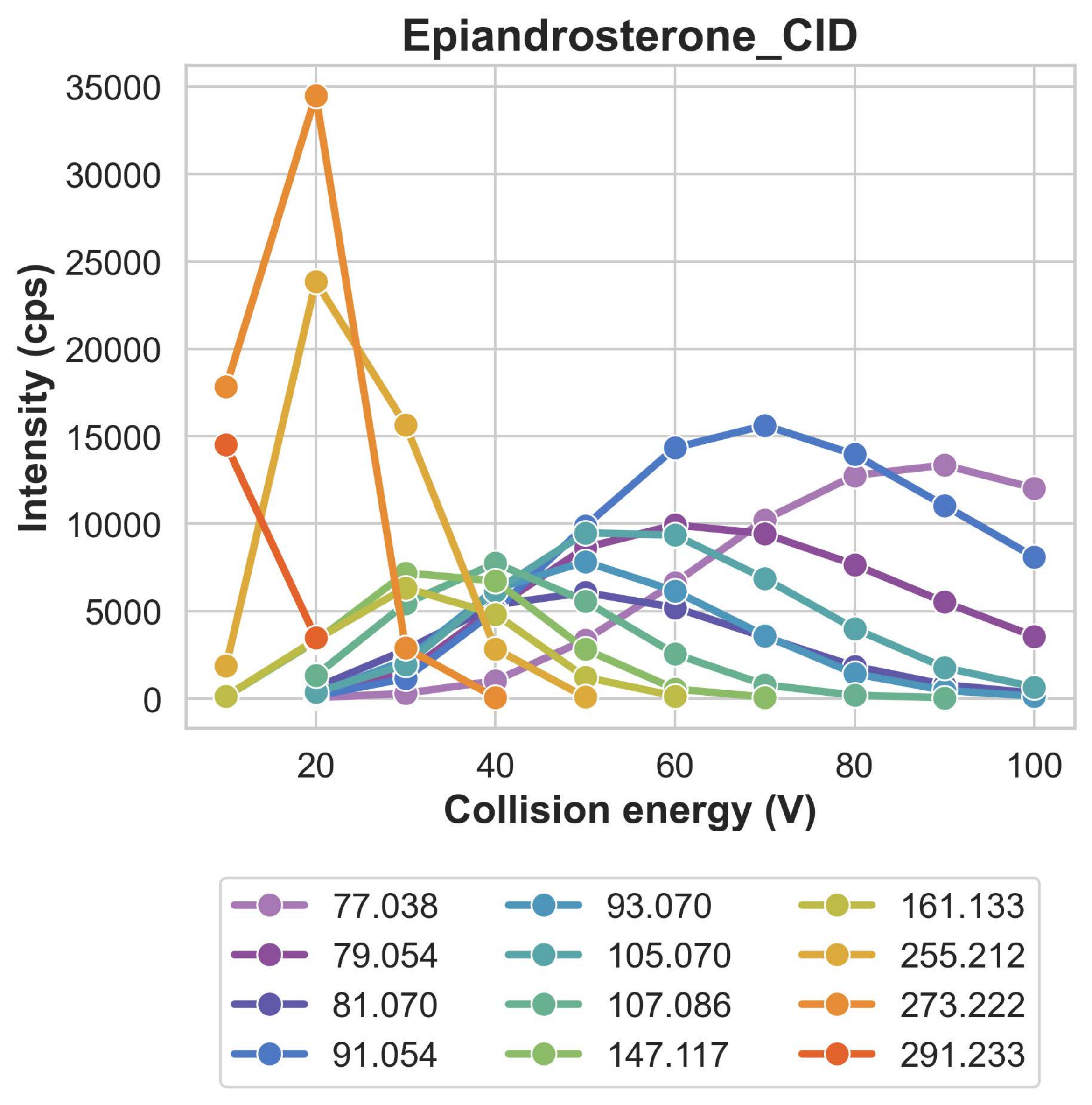

Epiandrosterone_CID
Intensity (cps)
35000
30000
25000
20000
15000
10000
5000
0
20
40
60
80
100
Collision energy (V)
77.038
79.054
81.070
91.054
93.070
105.070
107.086
147.117
161.133
255.212
273.222
291.233

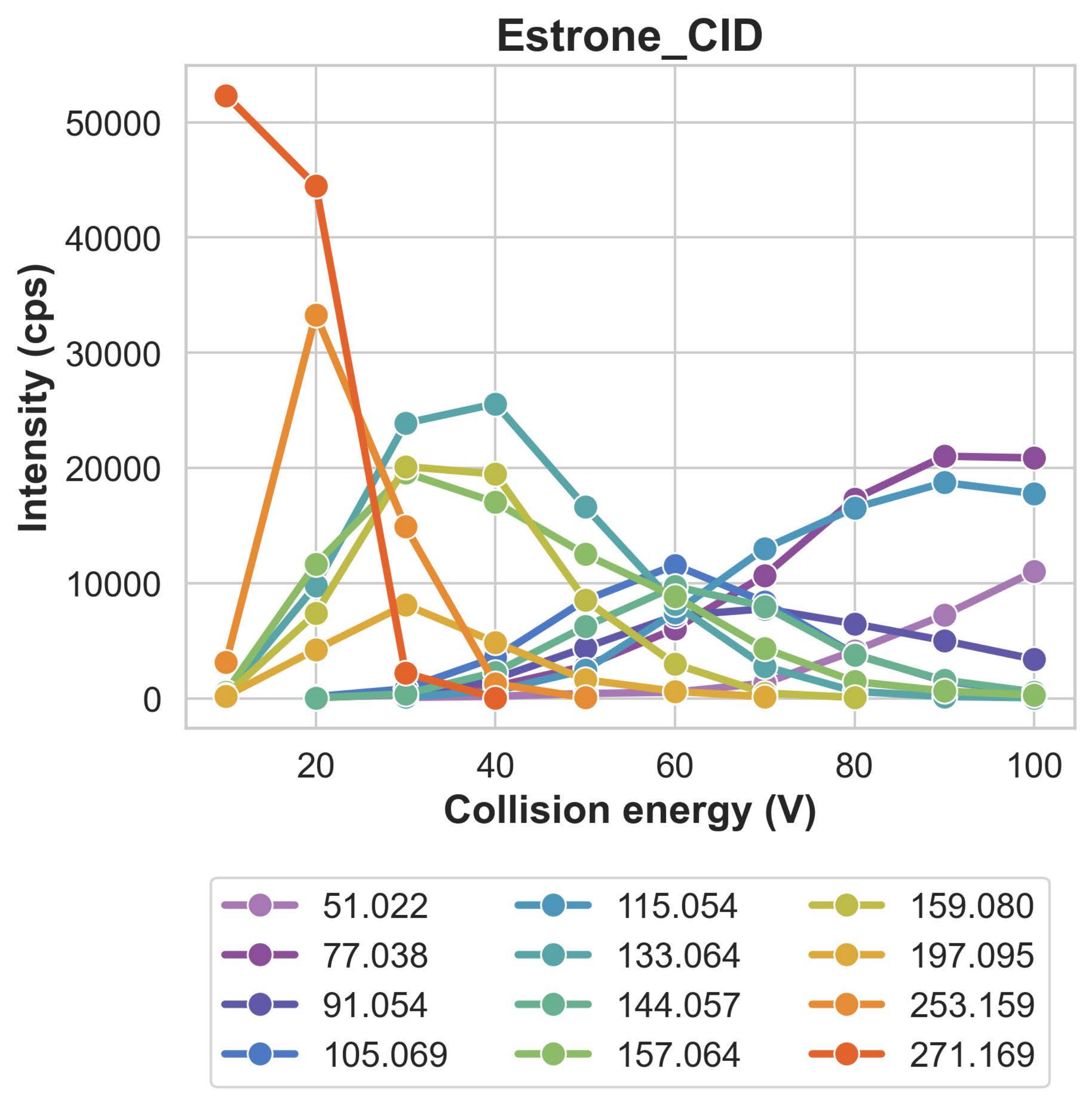
Estrone_CID
Intensity (cps)
50000
40000
30000
20000
10000
0
20
40
60
80
100
Collision energy (V)
51.022
77.038
91.054
105.069
115.054
133.064
144.057
157.064
159.080
197.095
253.159
271.169

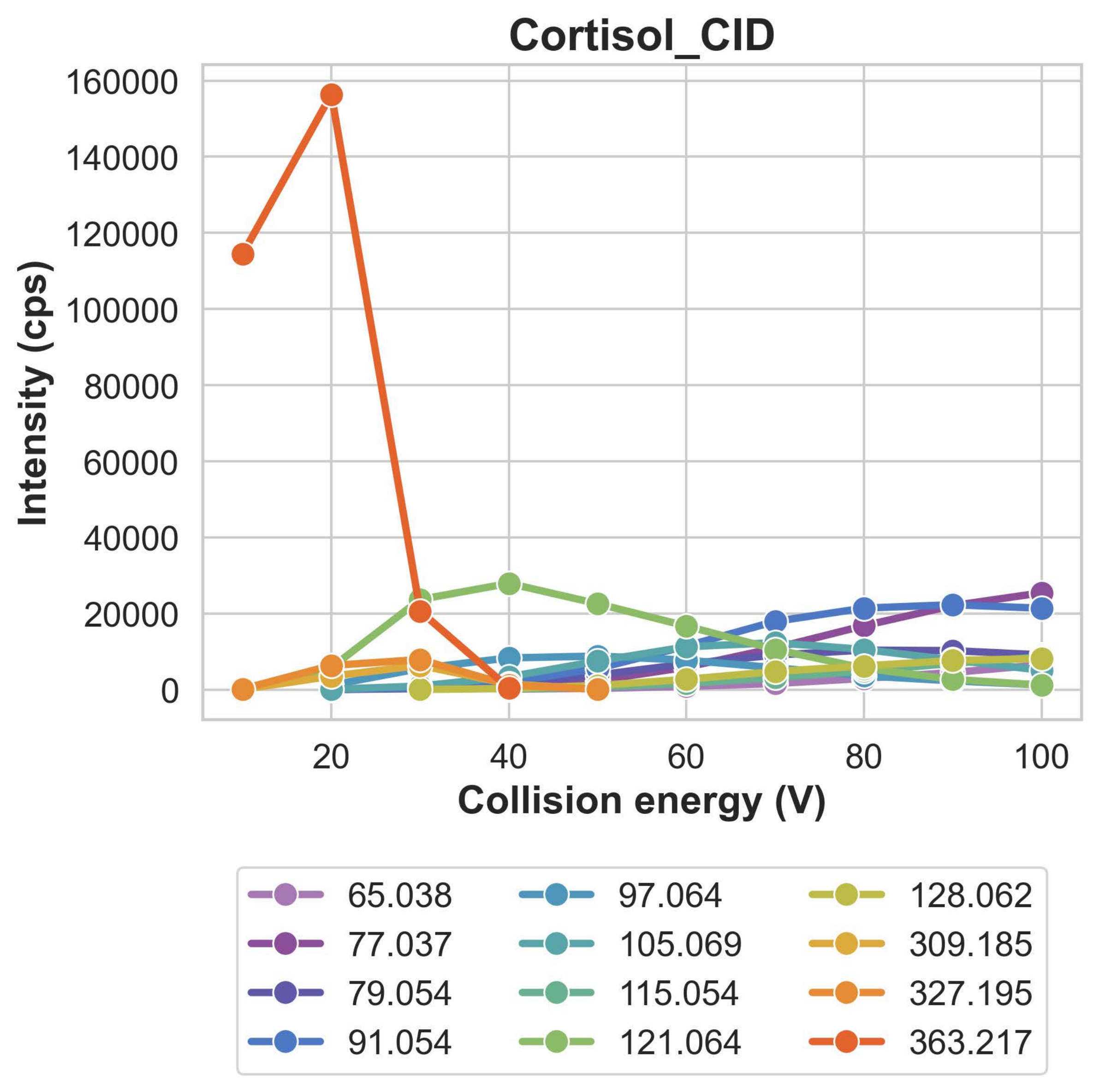

Cortisol_CID
Intensity (cps)
160000
140000
120000
100000
80000
60000
40000
20000
0
20
40
60
80
100
Collision energy (V)
65.038
77.037
79.054
91.054
97.064
105.069
115.054
121.064
128.062
309.185
327.195
363.217

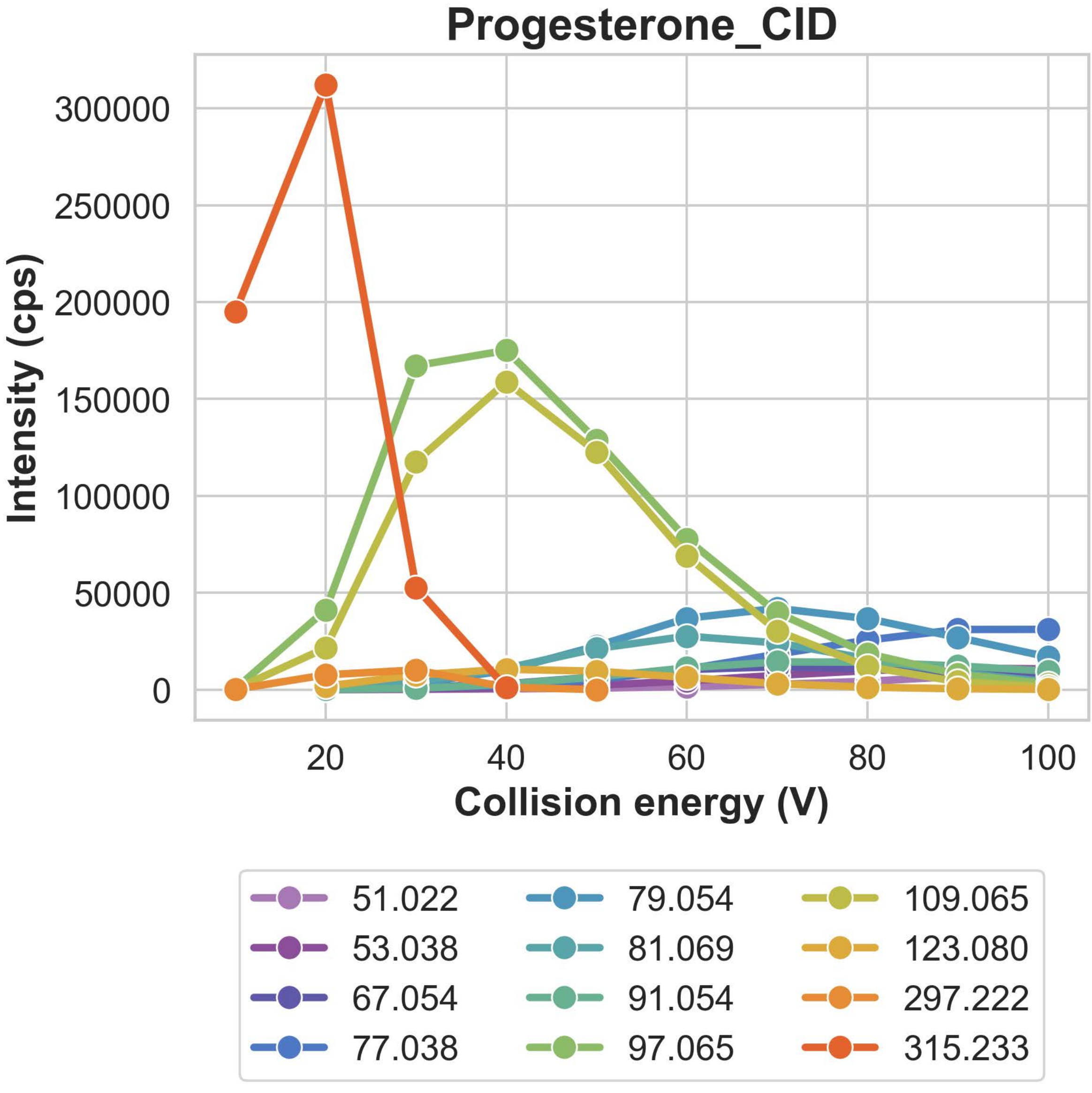
Progesterone_CID
Intensity (cps)
300000
250000
200000
150000
100000
50000
0
20
40
60
80
100
Collision energy (V)
51.022
53.038
67.054
77.038
79.054
81.069
91.054
97.065
109.065
123.080
297.222
315.233

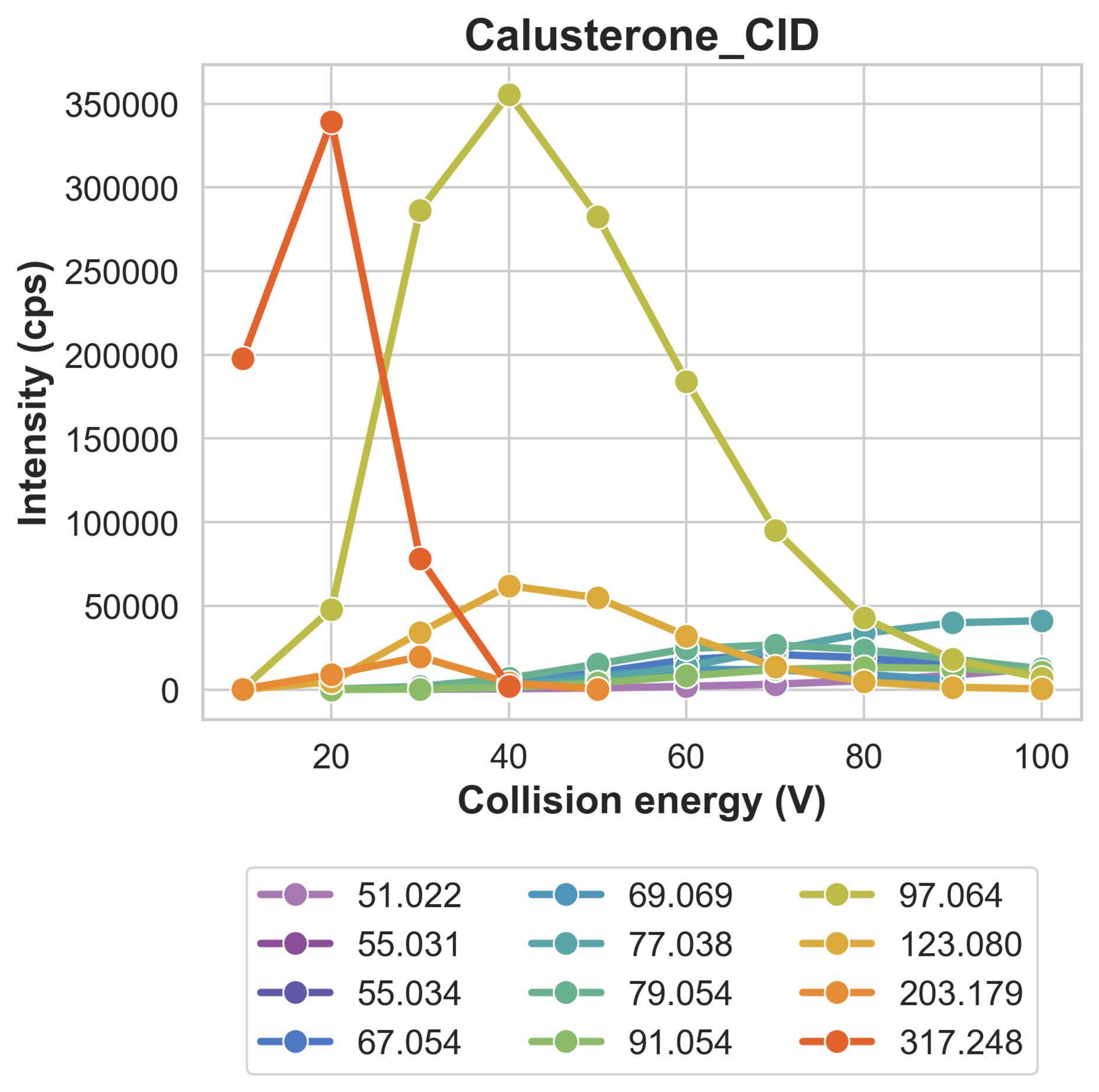
Calusterone_CID
Intensity (cps)
350000
300000
250000
200000
150000
100000
50000
0
20
40
60
80
100
Collision energy (V)
51.022
55.031
55.034
67.054
69.069
77.038
79.054
91.054
97.064
123.080
203.179
317.248

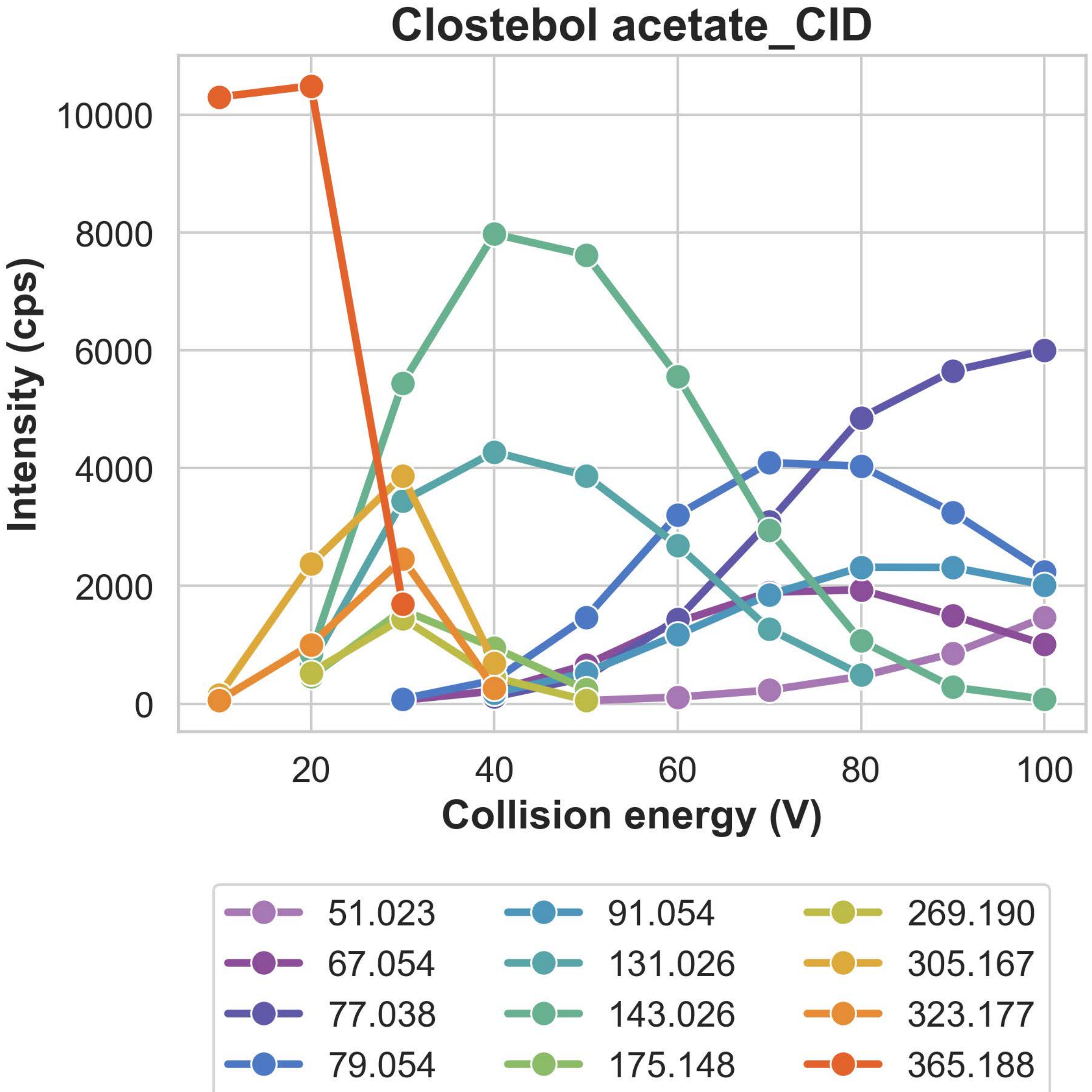
Clostebol acetate_CID
Intensity (cps)
0
2000
4000
6000
8000
10000
20
40
60
80
100
Collision energy (V)
51.023
67.054
77.038
79.054
91.054
131.026
143.026
175.148
269.190
305.167
323.177
365.188

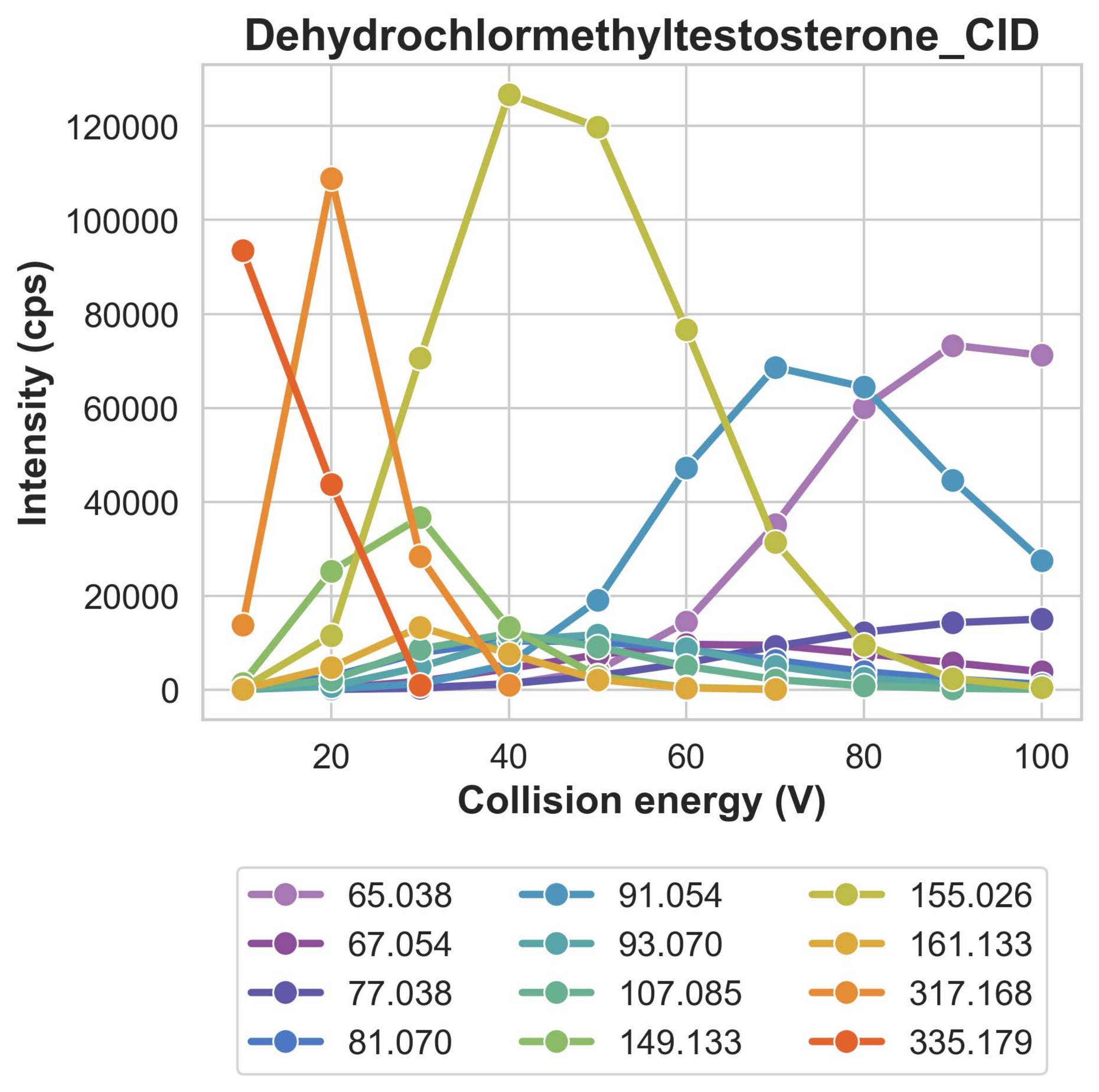

Dehydrochlormethyltestosterone_CID
Intensity (cps)
0
20000
40000
60000
80000
100000
120000
20
40
60
80
100
Collision energy (V)
65.038
67.054
77.038
81.070
91.054
93.070
107.085
149.133
155.026
161.133
317.168
335.179

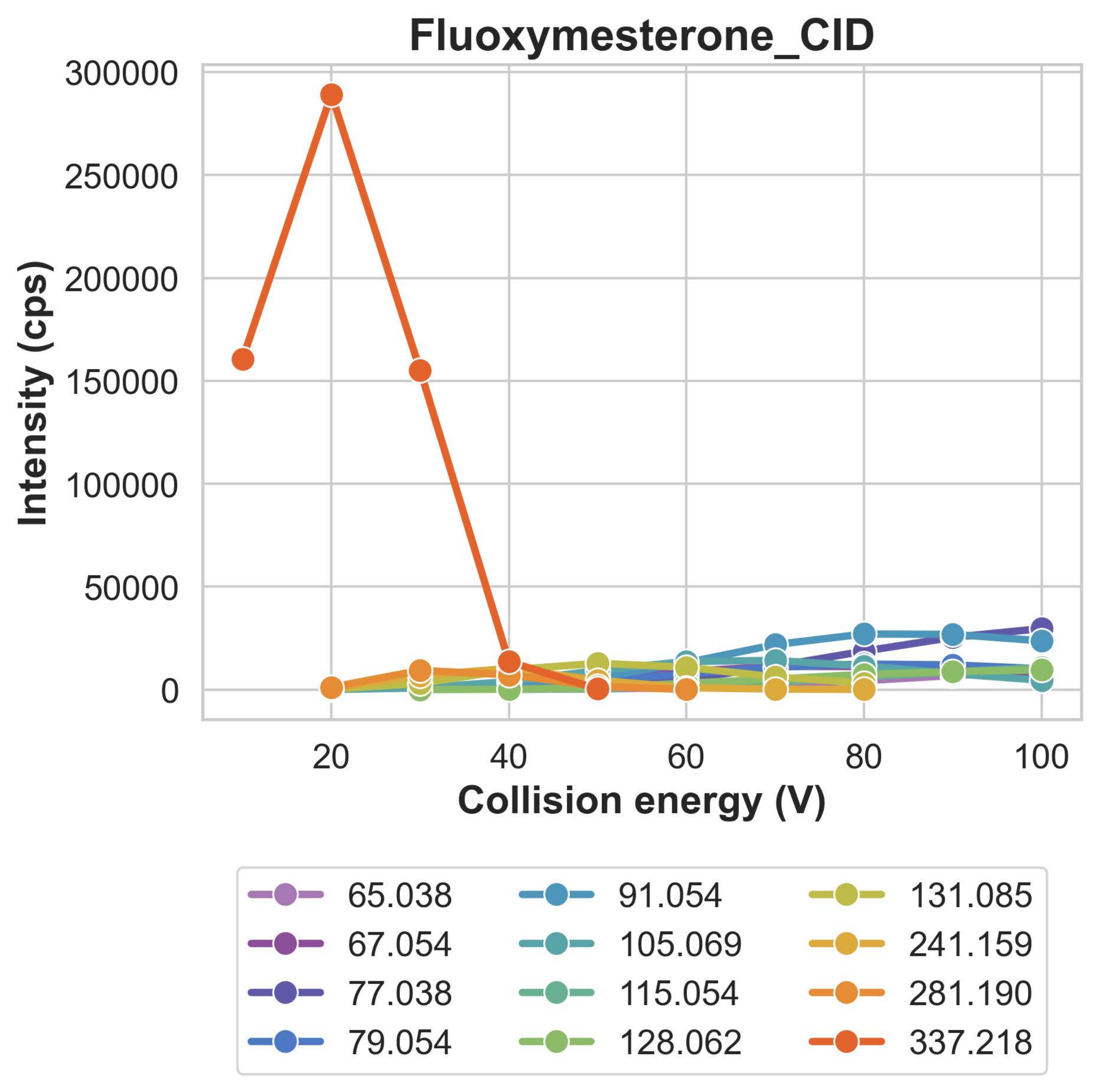
Fluoxymesterone_CID
Intensity (cps)
300000
250000
200000
150000
100000
50000
0
20
40
60
80
100
Collision energy (V)
65.038
67.054
77.038
79.054
91.054
105.069
115.054
128.062
131.085
241.159
281.190
337.218

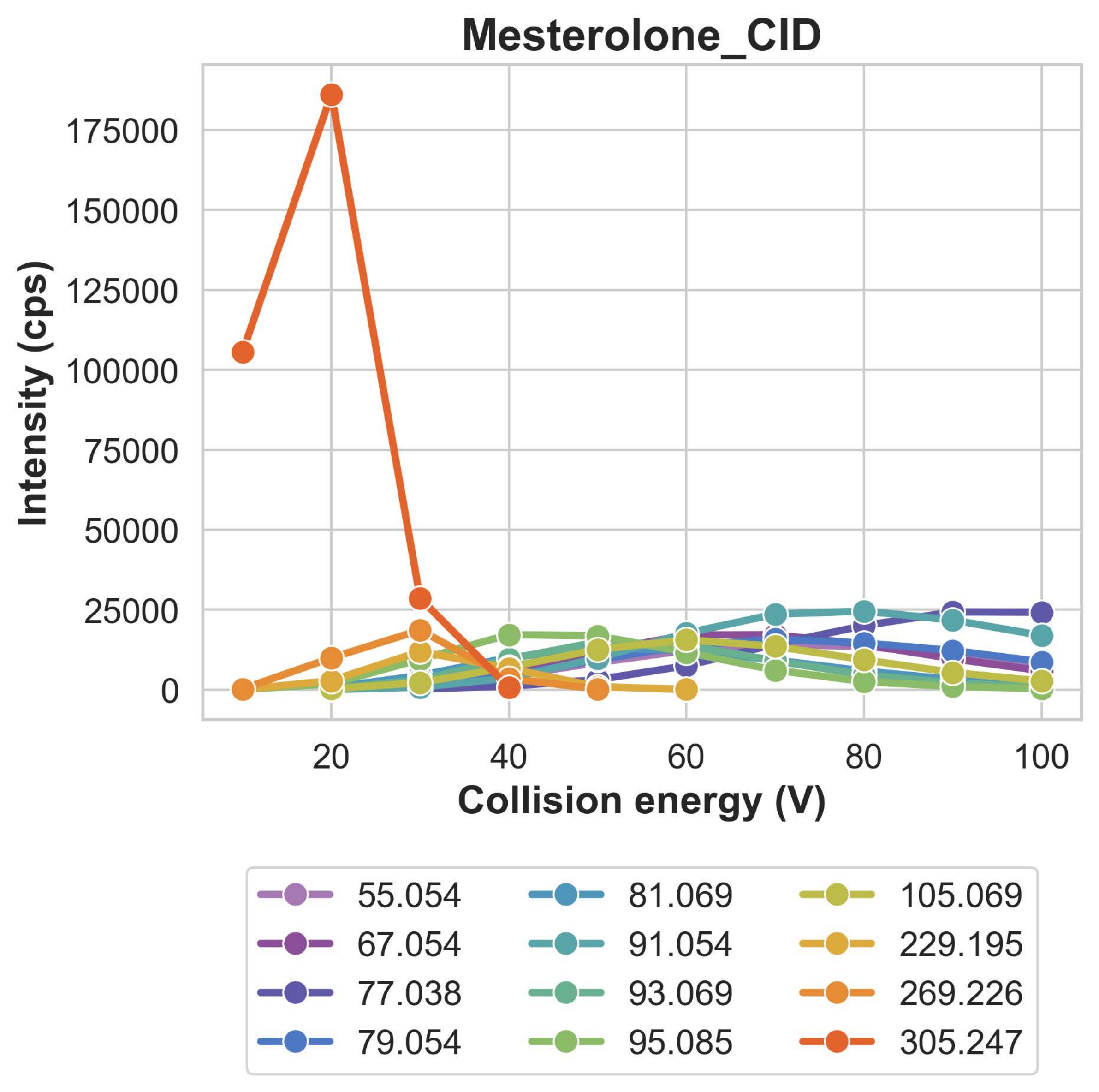
Mesterolone_CID
Intensity (cps)
175000
150000
125000
100000
75000
50000
25000
0
20
40
60
80
100
Collision energy (V)
55.054
67.054
77.038
79.054
81.069
91.054
93.069
95.085
105.069
229.195
269.226
305.247

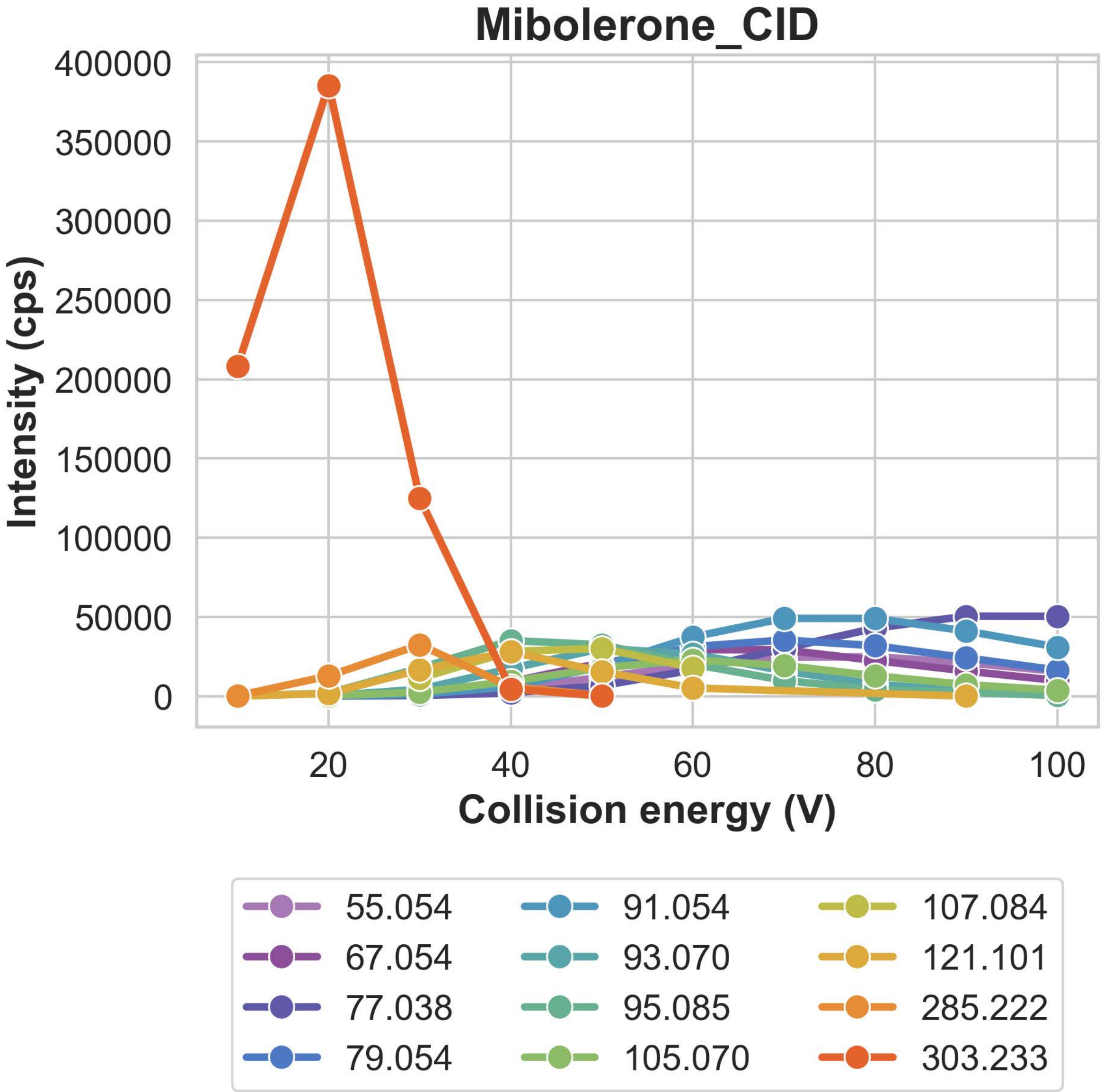
Mibolerone_CID
Intensity (cps)
400000
350000
300000
250000
200000
150000
100000
50000
0
20
40
60
80
100
Collision energy (V)
55.054
67.054
77.038
79.054
91.054
93.070
95.085
105.070
107.084
121.101
285.222
303.233

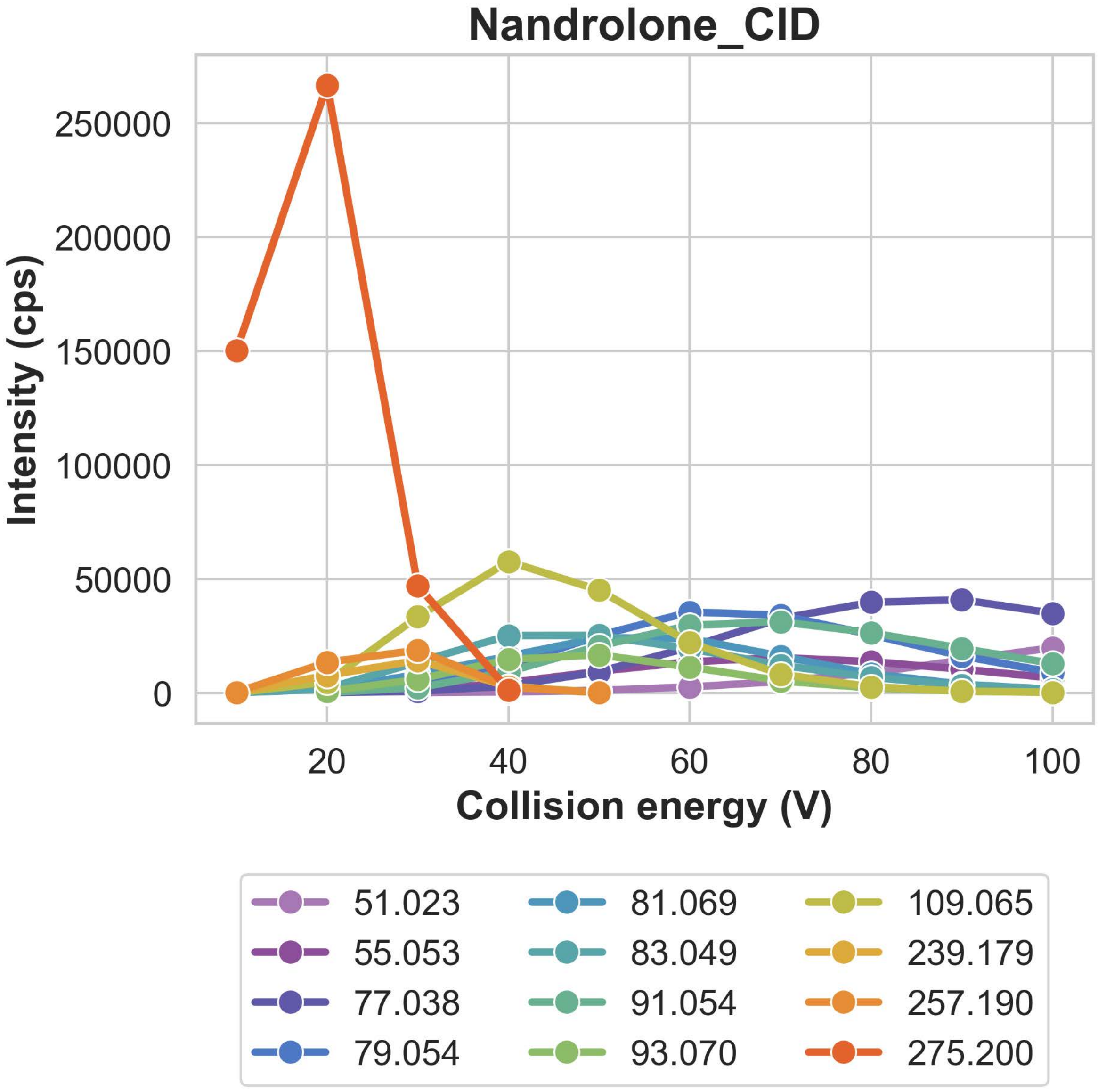

Nandrolone_CID
Intensity (cps)
250000
200000
150000
100000
50000
0
20
40
60
80
100
Collision energy (V)
51.023
55.053
77.038
79.054
81.069
83.049
91.054
93.070
109.065
239.179
257.190
275.200

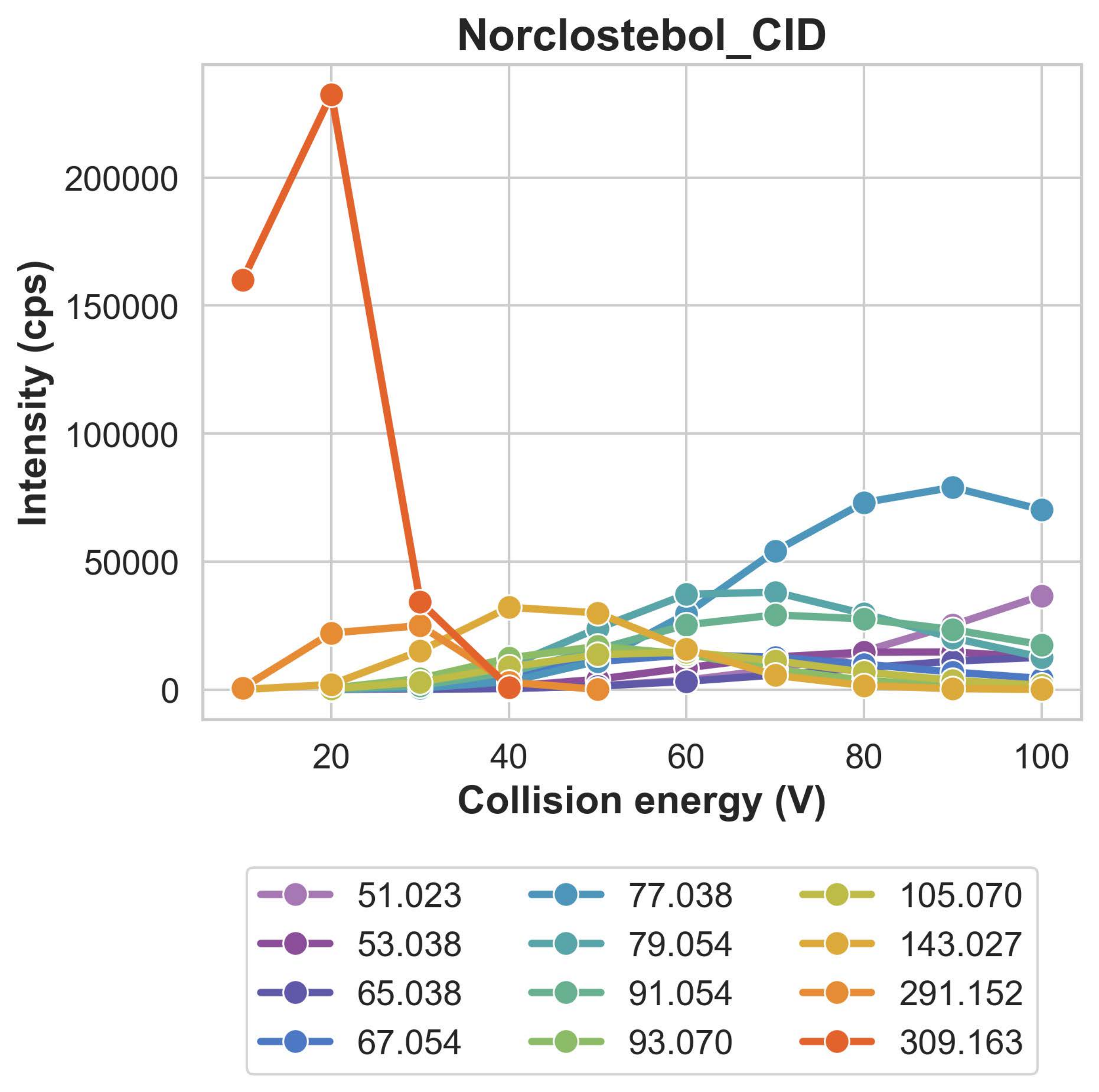

Norclostebol_CID
Intensity (cps)
200000
150000
100000
50000
0
20
40
60
80
100
Collision energy (V)
51.023
53.038
65.038
67.054
77.038
79.054
91.054
93.070
105.070
143.027
291.152
309.163

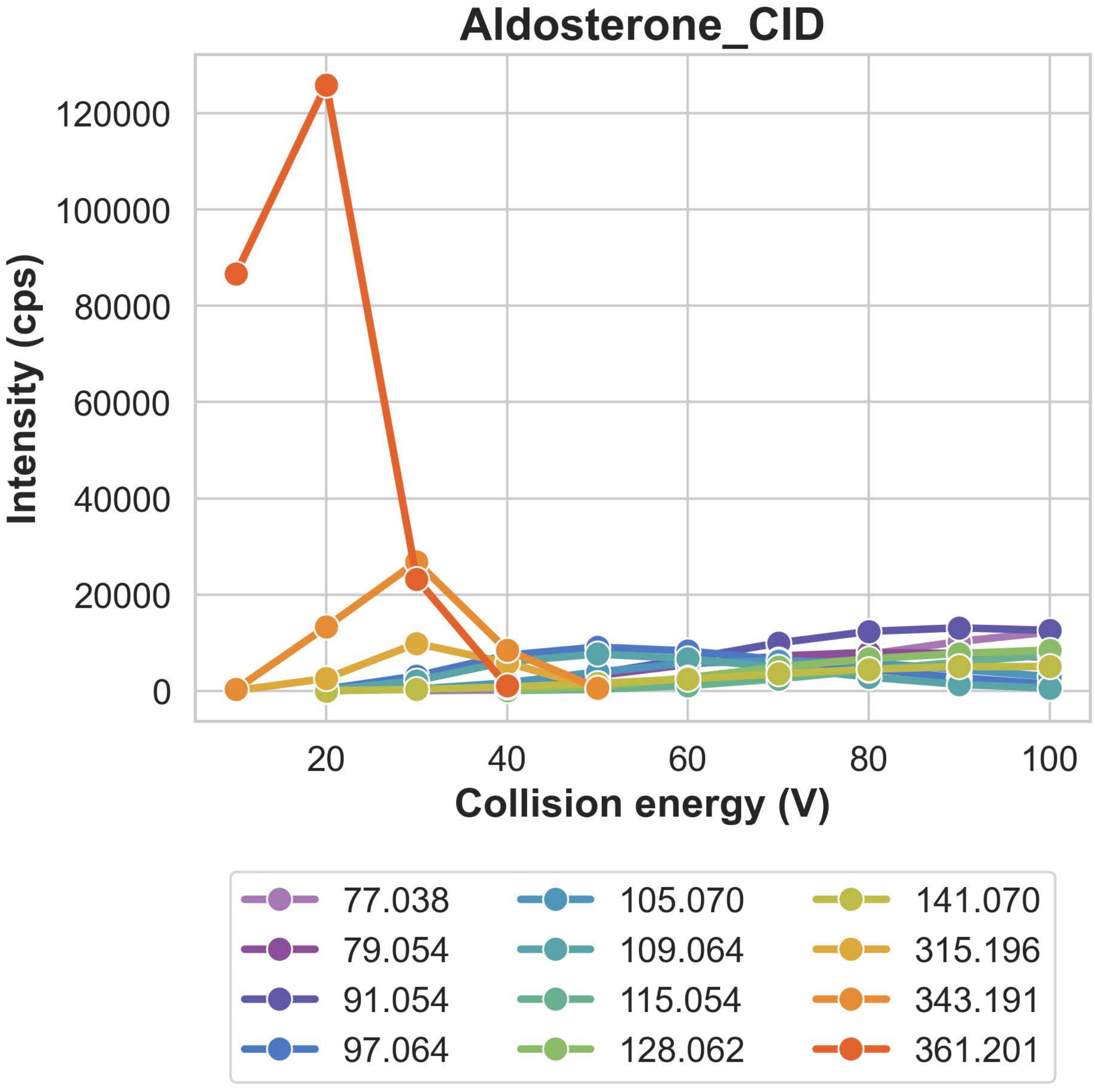
Aldosterone_CID
Intensity (cps)
120000
100000
80000
60000
40000
20000
0
20
40
60
80
100
Collision energy (V)
77.038
79.054
91.054
97.064
105.070
109.064
115.054
128.062
141.070
315.196
343.191
361.201

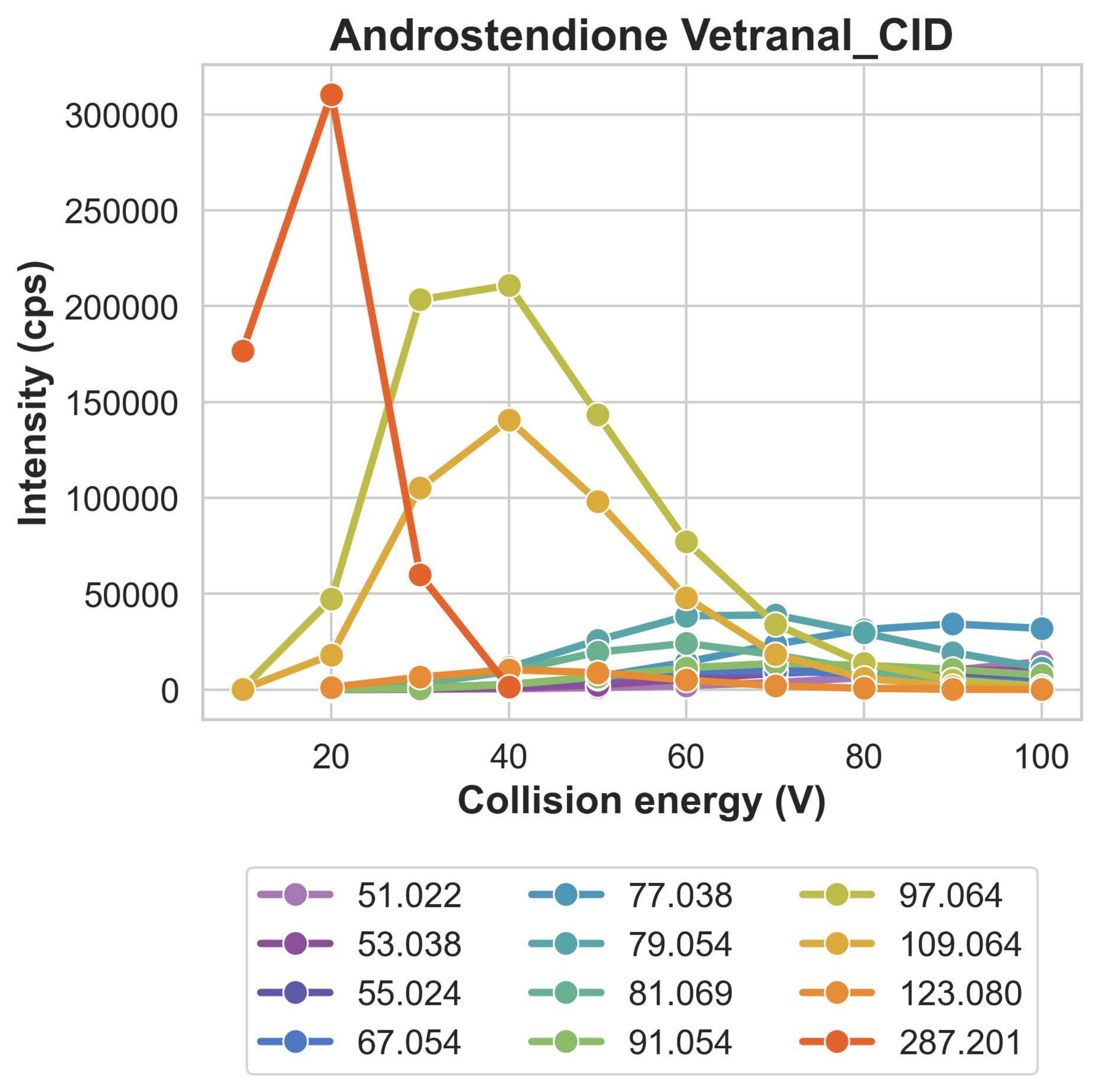
Androstendione Vetranal_CID
Intensity (cps)
300000
250000
200000
150000
100000
50000
0
20
40
60
80
100
Collision energy (V)
51.022
53.038
55.024
67.054
77.038
79.054
81.069
91.054
97.064
109.064
123.080
287.201

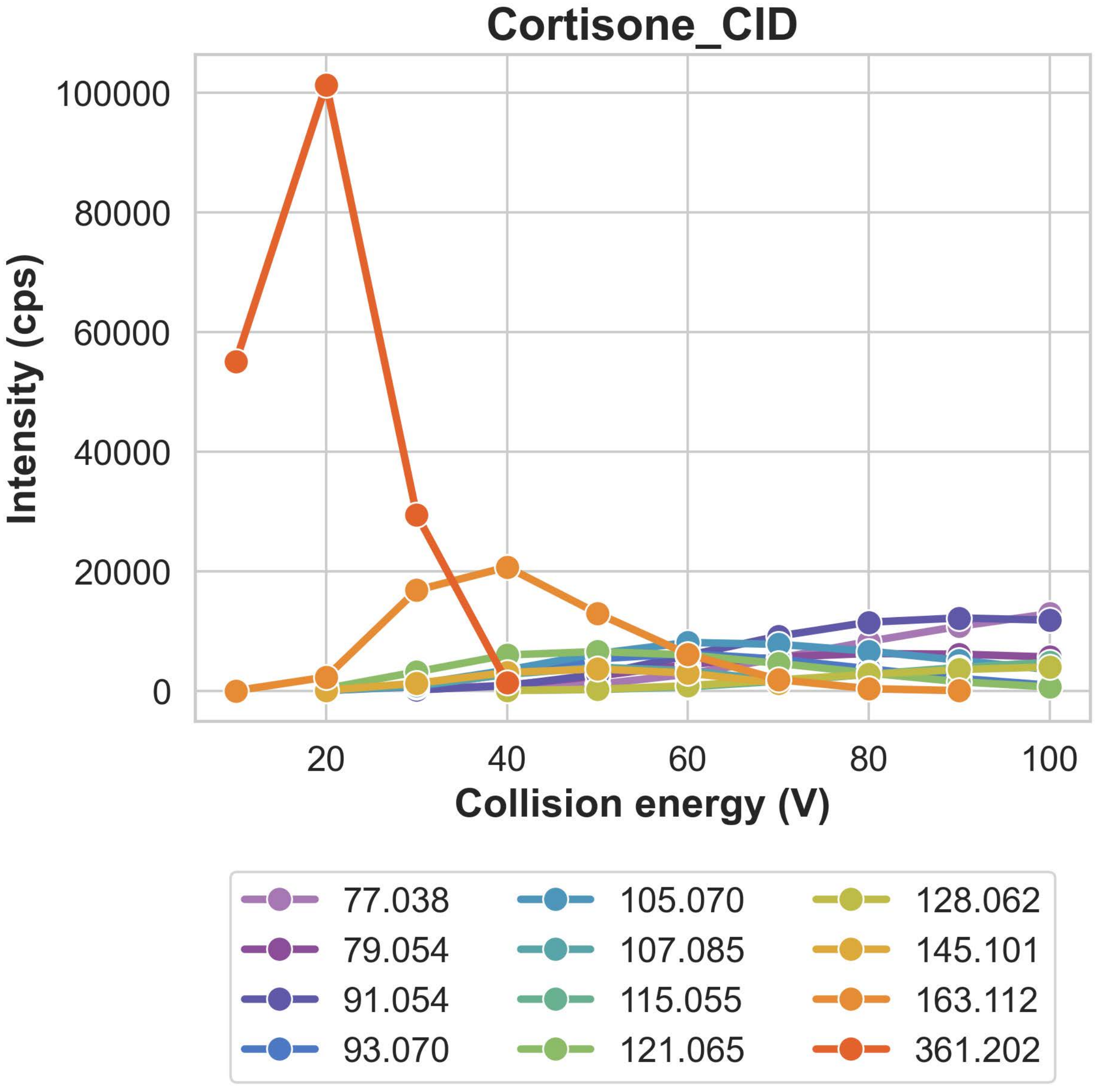
Cortisone_CID
Intensity (cps)
100000
80000
60000
40000
20000
0
20
40
60
80
100
Collision energy (V)
77.038
79.054
91.054
93.070
105.070
107.085
115.055
121.065
128.062
145.101
163.112
361.202

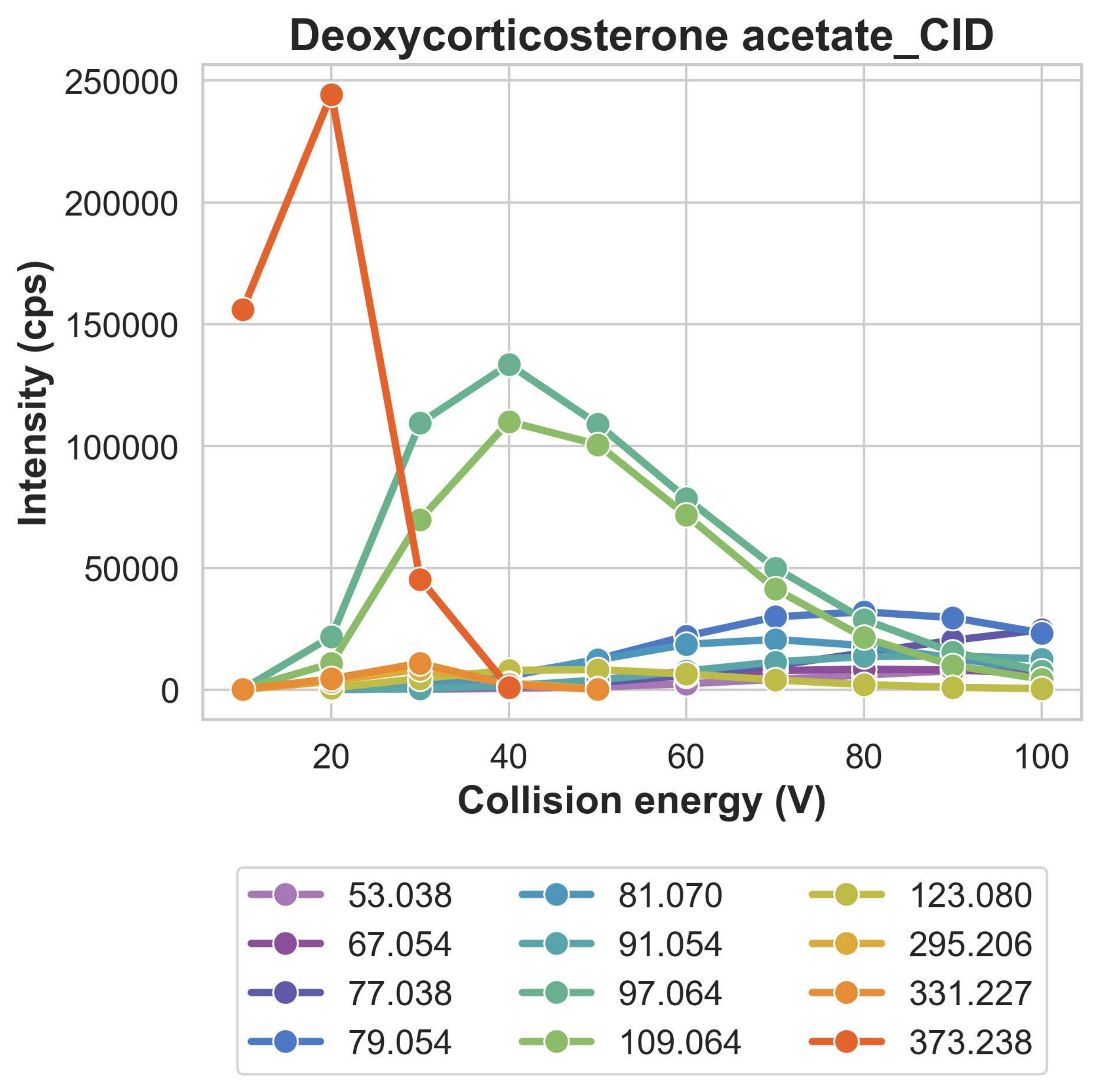
Deoxycorticosterone acetate_CID
Intensity (cps)
250000
200000
150000
100000
50000
0
20
40
60
80
100
Collision energy (V)
53.038
67.054
77.038
79.054
81.070
91.054
97.064
109.064
123.080
295.206
331.227
373.238

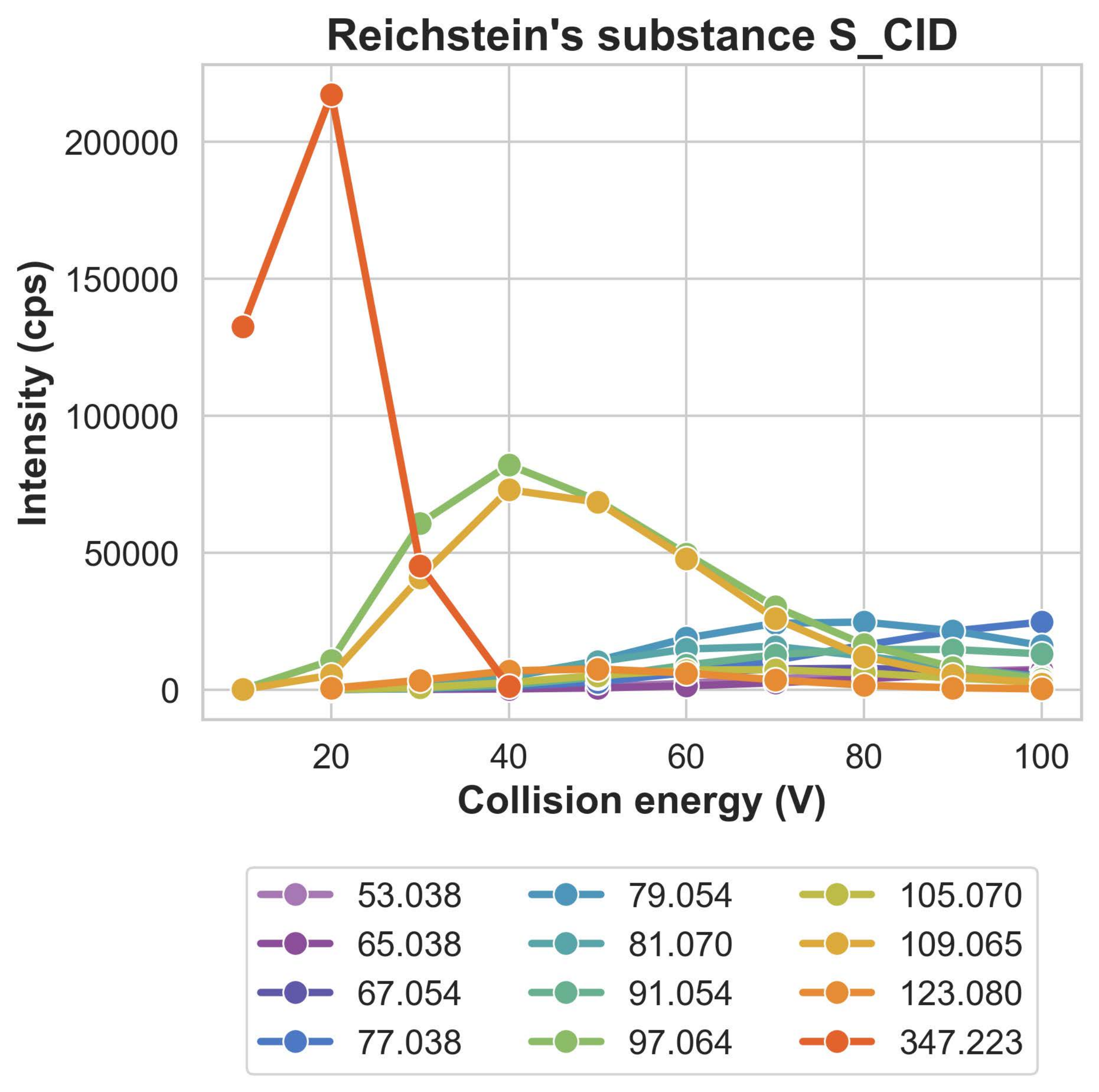
Reichstein's substance S_CID
Intensity (cps)
200000
150000
100000
50000
0
20
40
60
80
100
Collision energy (V)
53.038
65.038
67.054
77.038
79.054
81.070
91.054
97.064
105.070
109.065
123.080
347.223

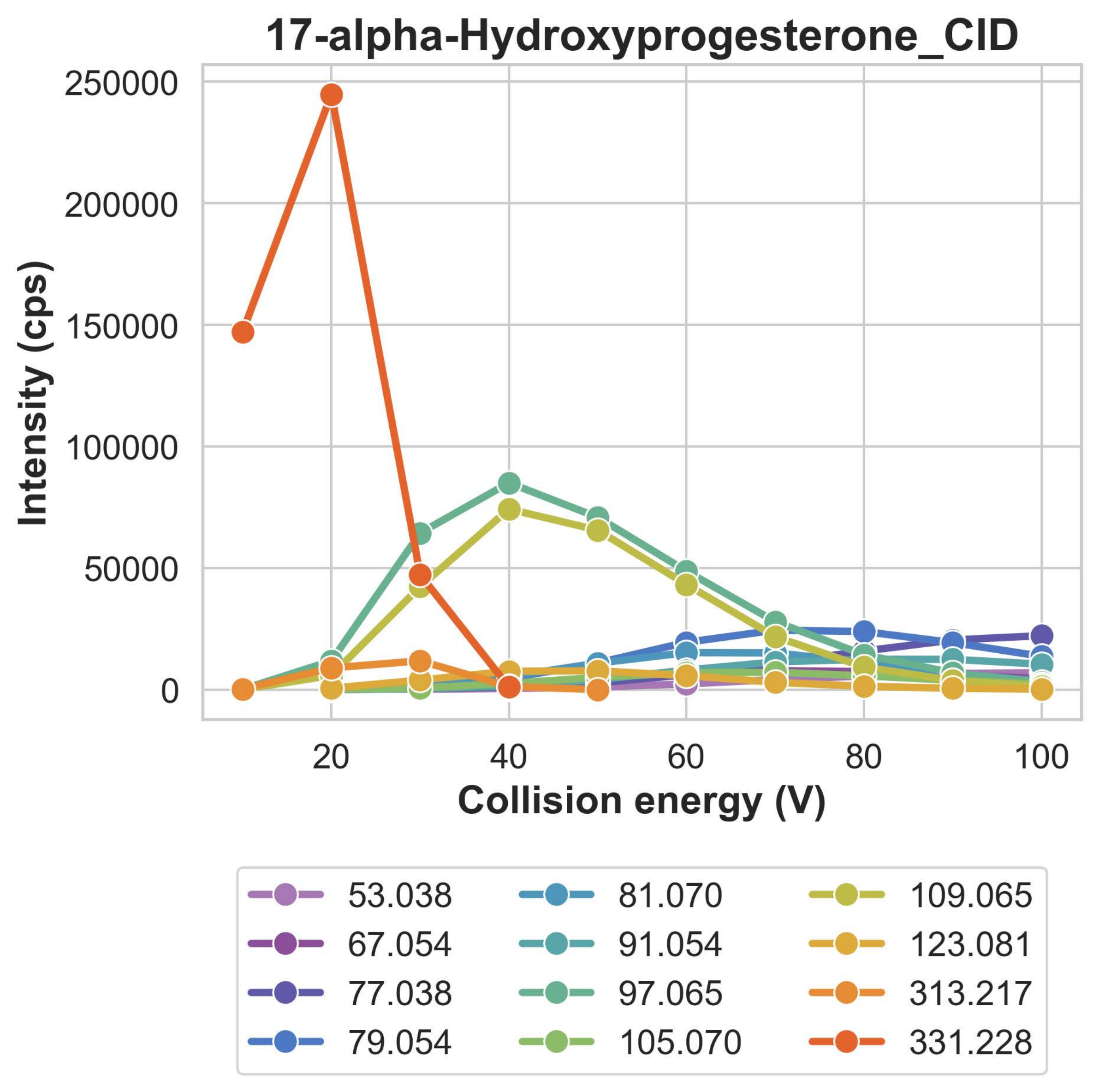

17-alpha-Hydroxyprogesterone_CID
Intensity (cps)
250000
200000
150000
100000
50000
0
20
40
60
80
100
Collision energy (V)
53.038
67.054
77.038
79.054
81.070
91.054
97.065
105.070
109.065
123.081
313.217
331.228

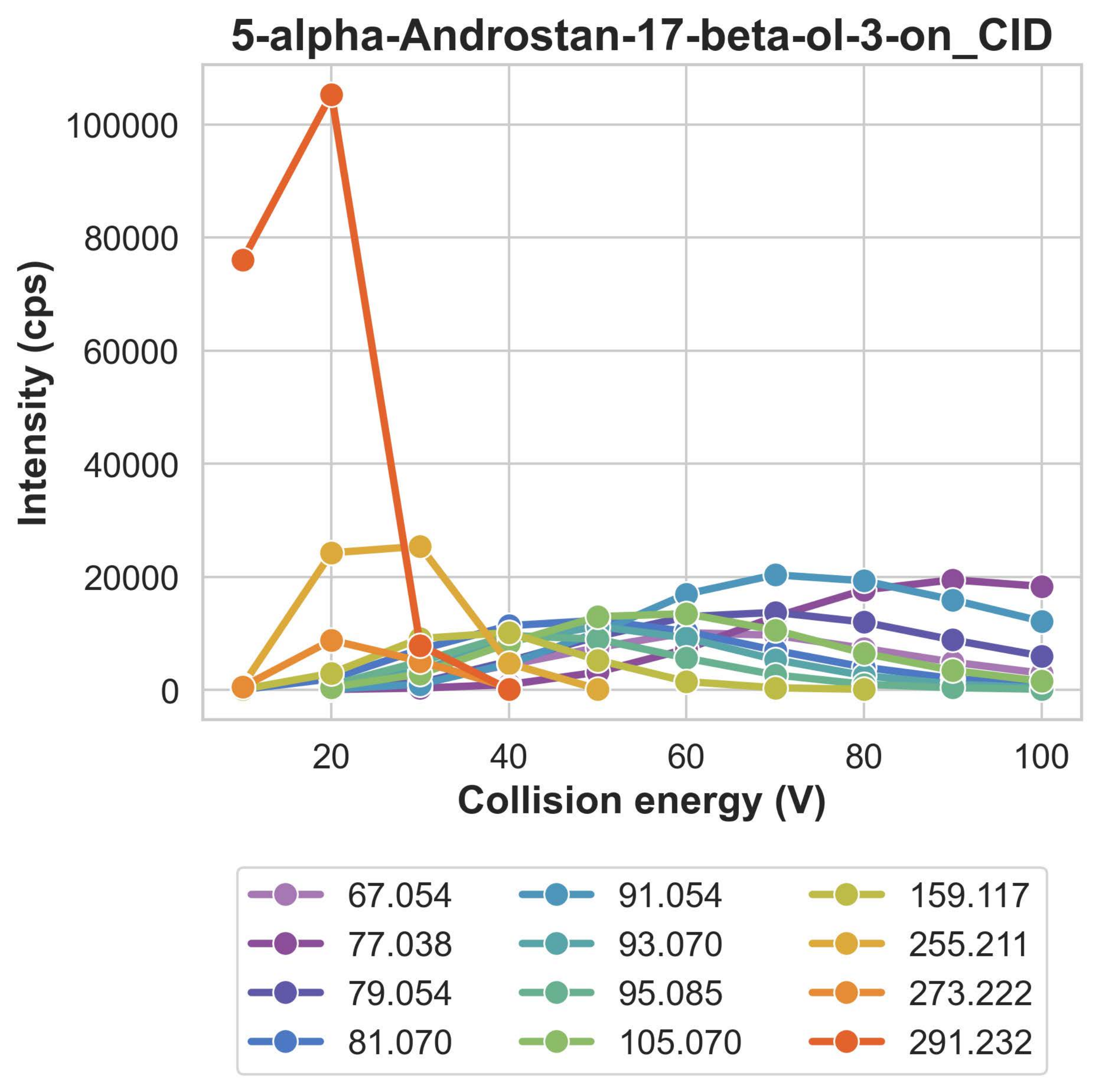
5-alpha-Androstan-17-beta-ol-3-on_CID
Intensity (cps)
100000
80000
60000
40000
20000
0
20
40
60
80
100
Collision energy (V)
67.054
77.038
79.054
81.070
91.054
93.070
95.085
105.070
159.117
255.211
273.222
291.232

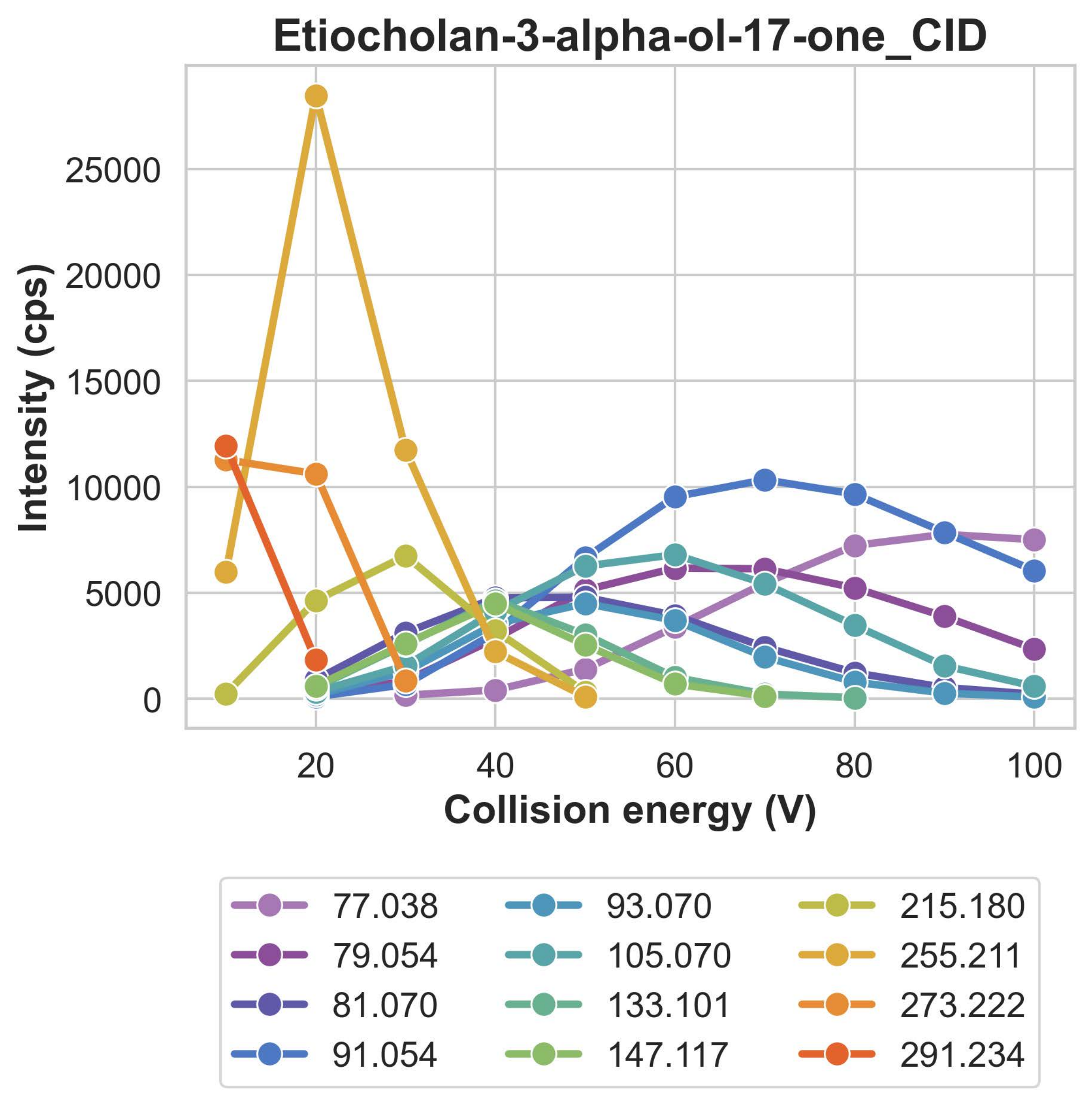

Etiocholan-3-alpha-ol-17-one_CID
Intensity (cps)
25000
20000
15000
10000
5000
0
20
40
60
80
100
Collision energy (V)
77.038
79.054
81.070
91.054
93.070
105.070
133.101
147.117
215.180
255.211
273.222
291.234

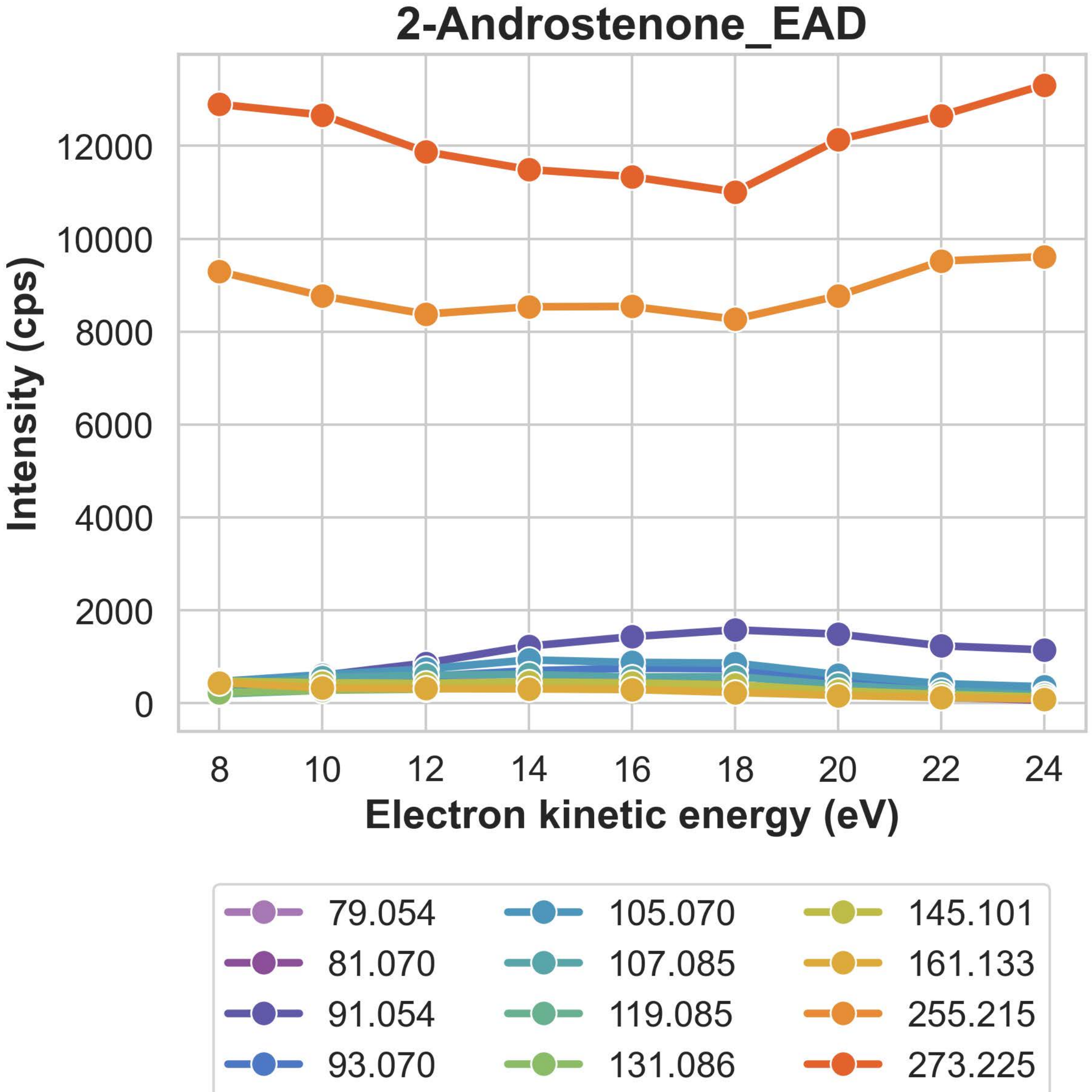
2-Androstenone_EAD
Intensity (cps)
12000
10000
8000
6000
4000
2000
0
8
10
12
14
16
18
20
22
24
Electron kinetic energy (eV)
79.054
81.070
91.054
93.070
105.070
107.085
119.085
131.086
145.101
161.133
255.215
273.225

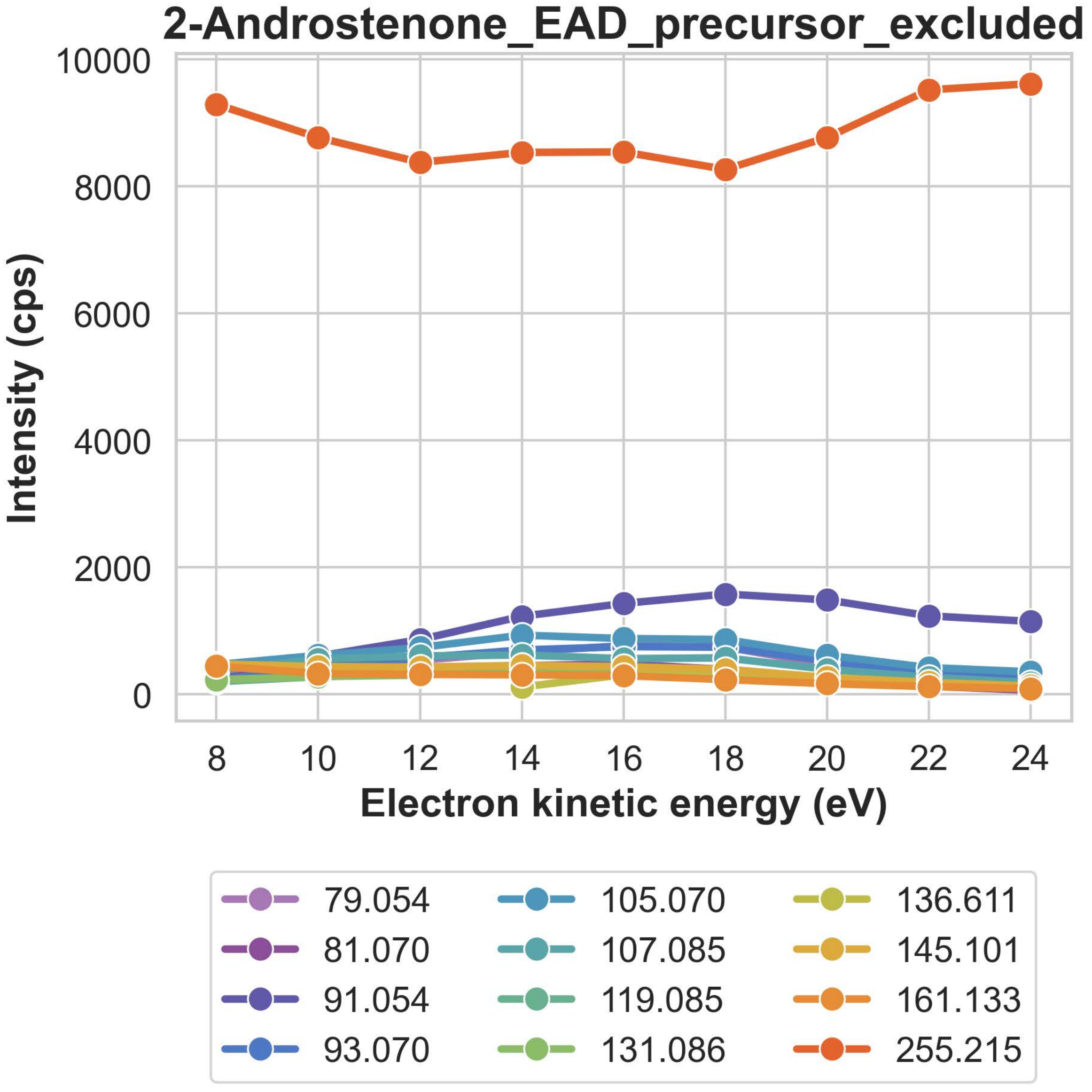
2-Androstenone_EAD_precursor_excluded
10000
8000
6000
4000
2000
0
Intensity (cps)
8
10
12
14
16
18
20
22
24
Electron kinetic energy (eV)
79.054
81.070
91.054
93.070
105.070
107.085
119.085
131.086
136.611
145.101
161.133
255.215

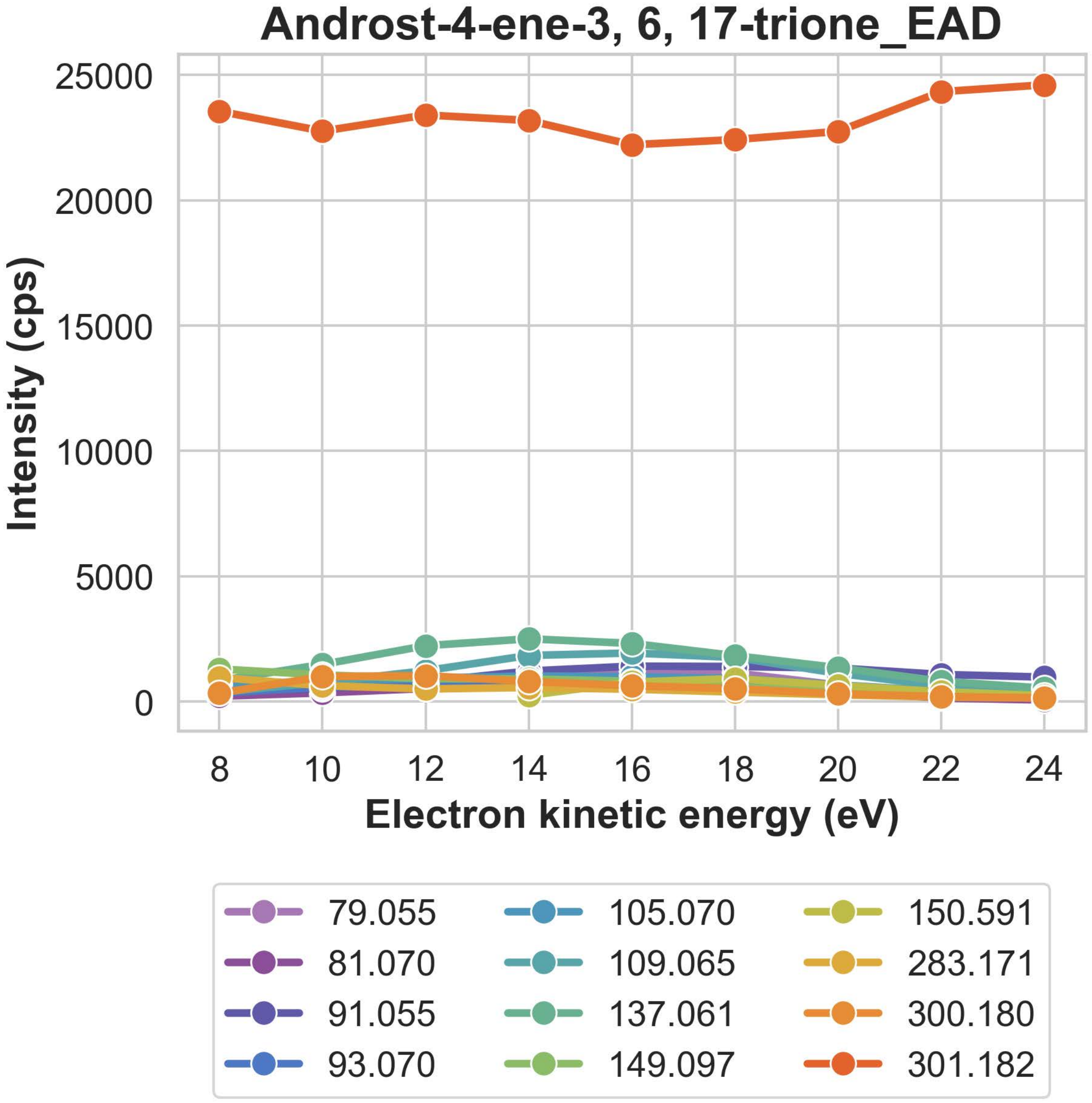
Androst-4-ene-3, 6, 17-trione_EAD
Intensity (cps)
25000
20000
15000
10000
5000
0
8
10
12
14
16
18
20
22
24
Electron kinetic energy (eV)
79.055
81.070
91.055
93.070
105.070
109.065
137.061
149.097
150.591
283.171
300.180
301.182

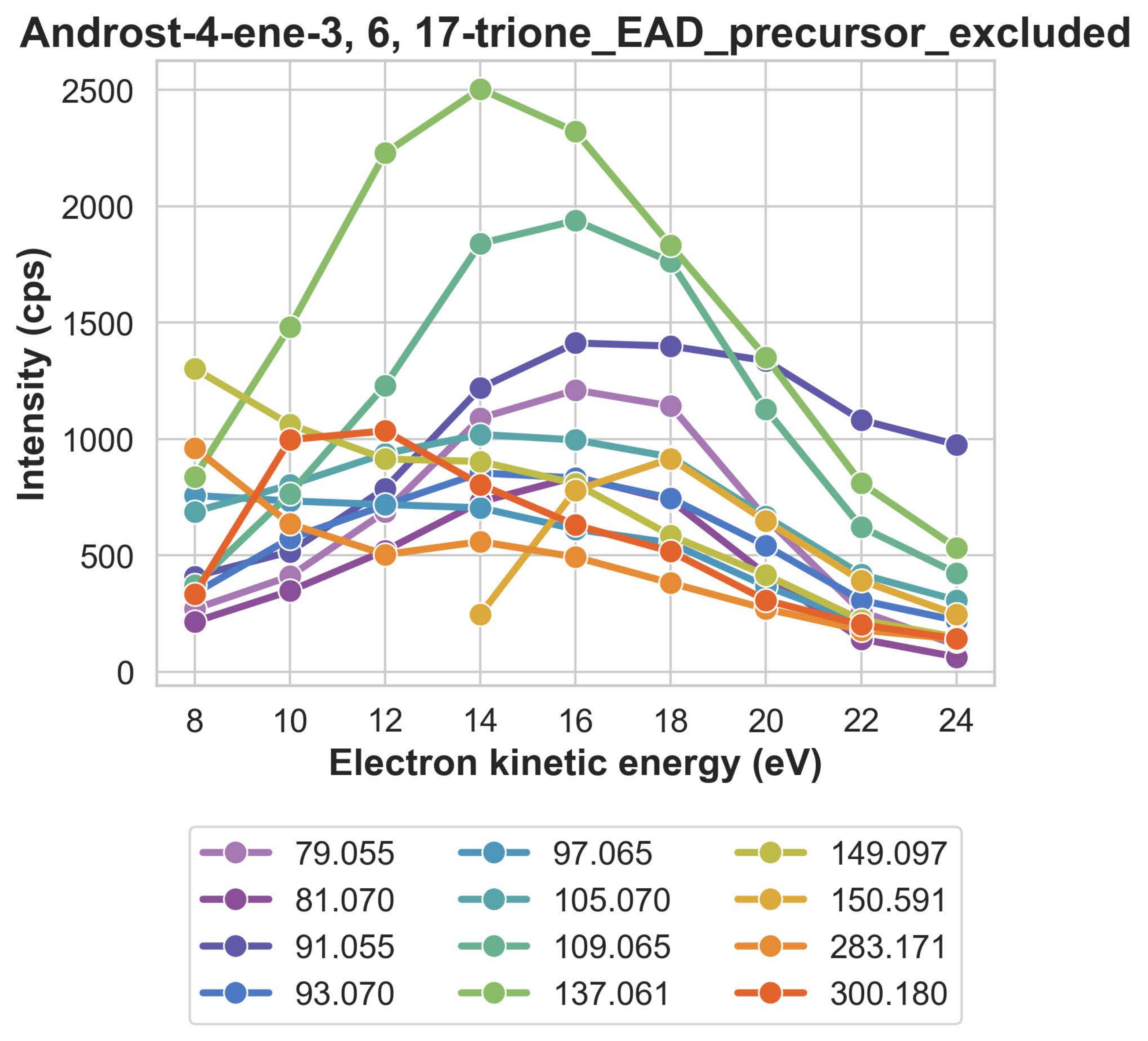
Androst-4-ene-3, 6, 17-trione_EAD_precursor_excluded
Intensity (cps)
2500
2000
1500
1000
500
0
8
10
12
14
16
18
20
22
24
Electron kinetic energy (eV)
79.055
81.070
91.055
93.070
97.065
105.070
109.065
137.061
149.097
150.591
283.171
300.180

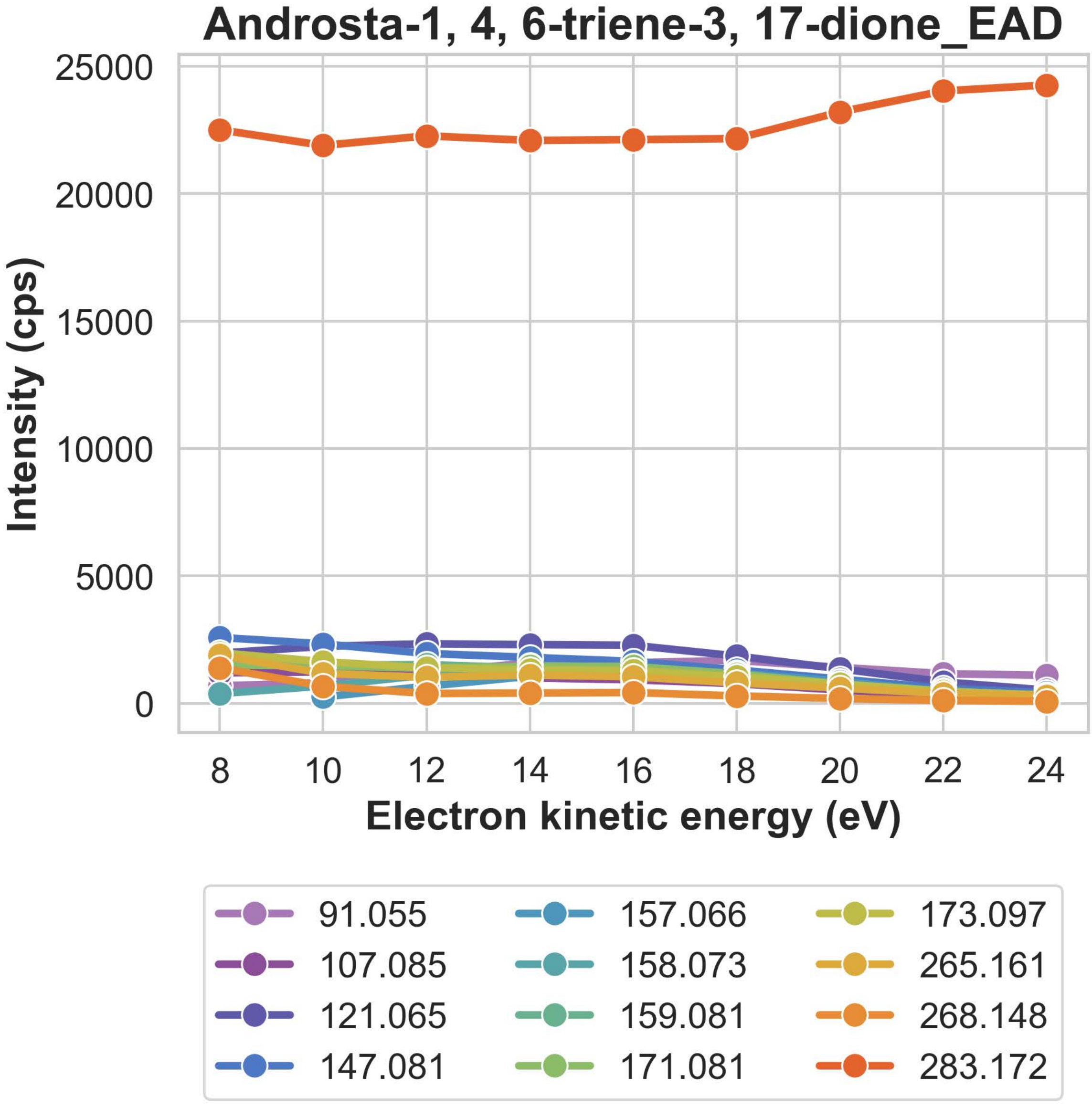
Androsta-1, 4, 6-triene-3, 17-dione_EAD
Intensity (cps)
25000
20000
15000
10000
5000
0
8
10
12
14
16
18
20
22
24
Electron kinetic energy (eV)
91.055
107.085
121.065
147.081
157.066
158.073
159.081
171.081
173.097
265.161
268.148
283.172

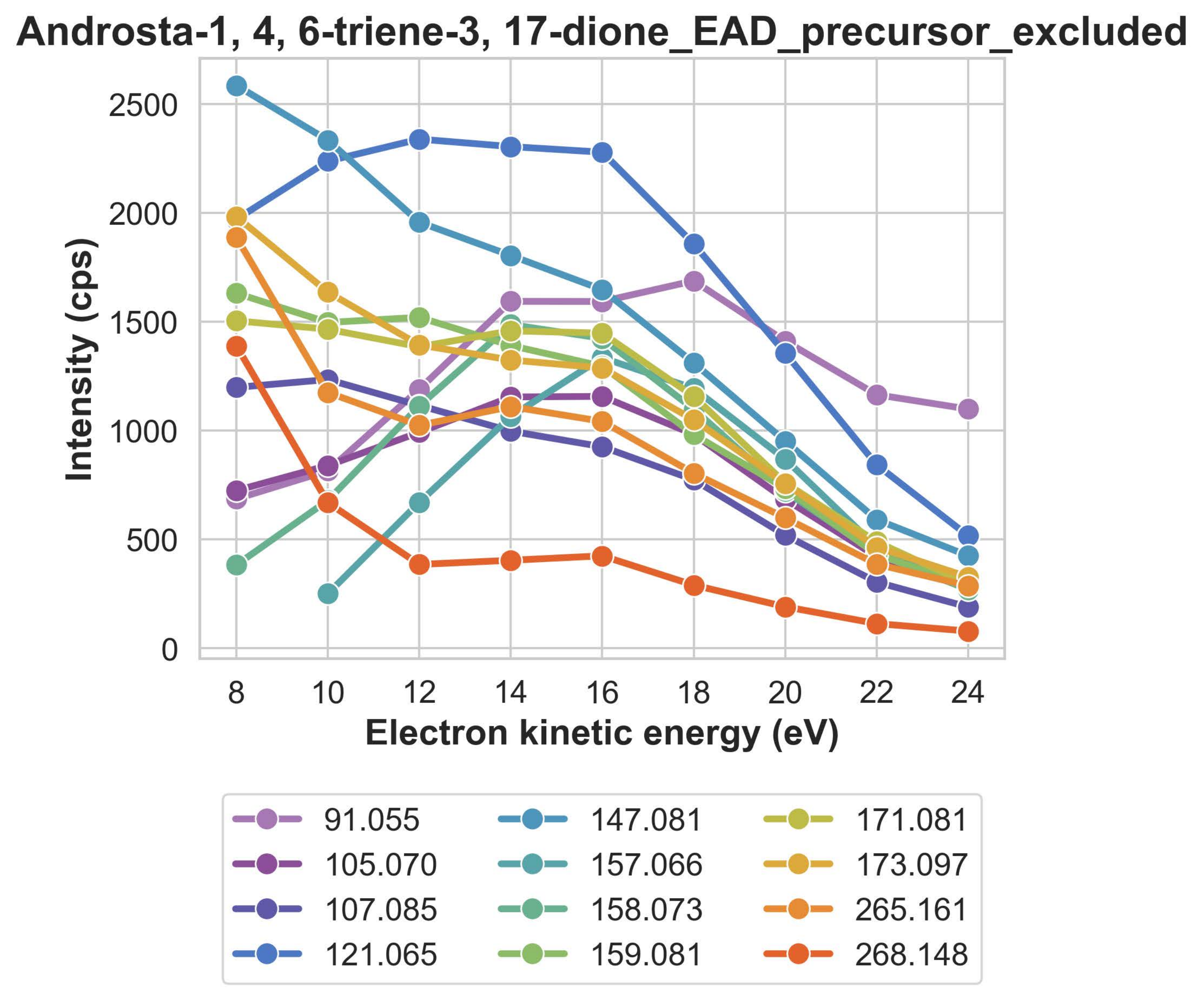
Androsta-1, 4, 6-triene-3, 17-dione_EAD_precursor_excluded
Intensity (cps)
Electron kinetic energy (eV)
0
500
1000
1500
2000
2500
8
10
12
14
16
18
20
22
24
91.055
105.070
107.085
121.065
147.081
157.066
158.073
159.081
171.081
173.097
265.161
268.148

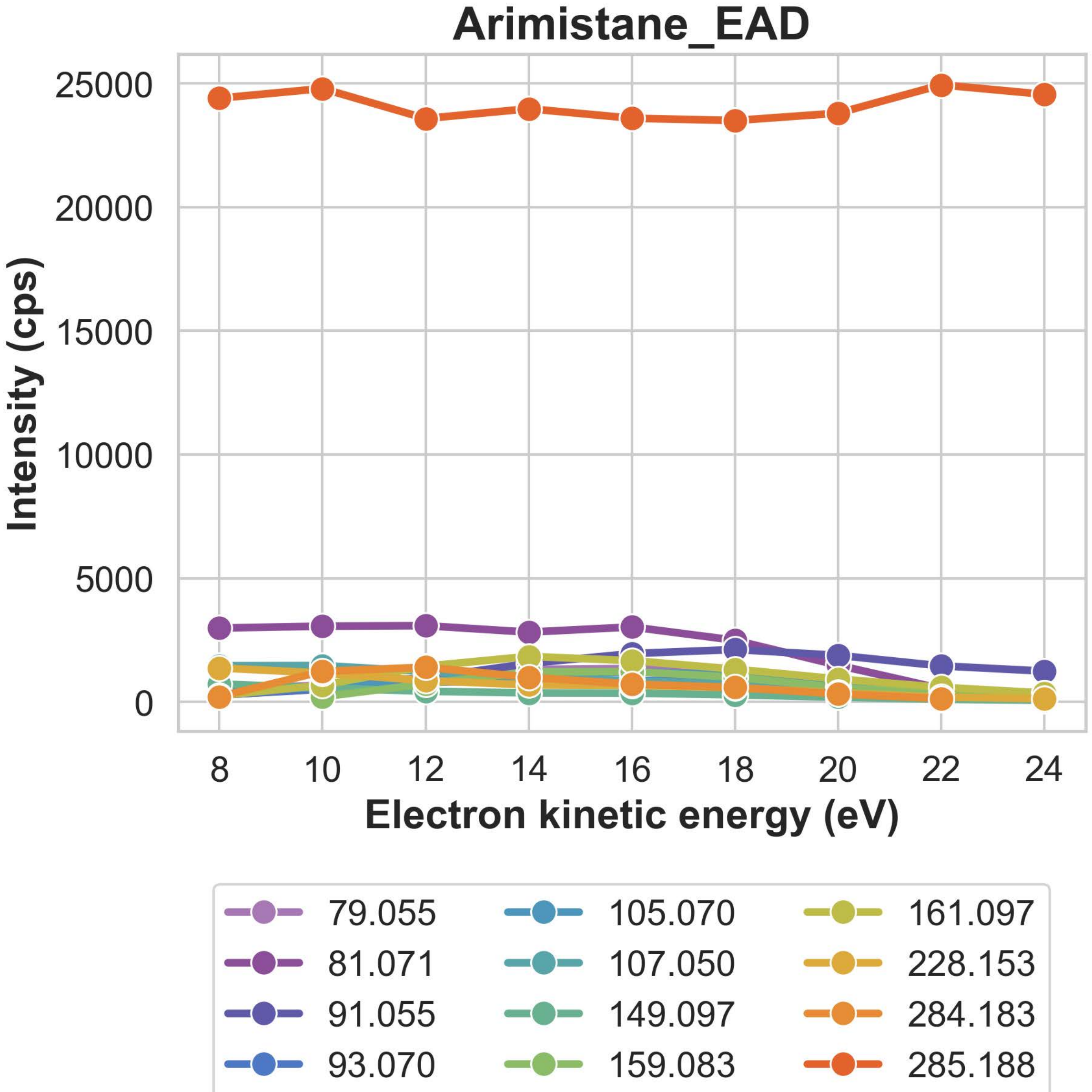
Arimistane_EAD
Intensity (cps)
25000
20000
15000
10000
5000
0
8
10
12
14
16
18
20
22
24
Electron kinetic energy (eV)
79.055
81.071
91.055
93.070
105.070
107.050
149.097
159.083
161.097
228.153
284.183
285.188

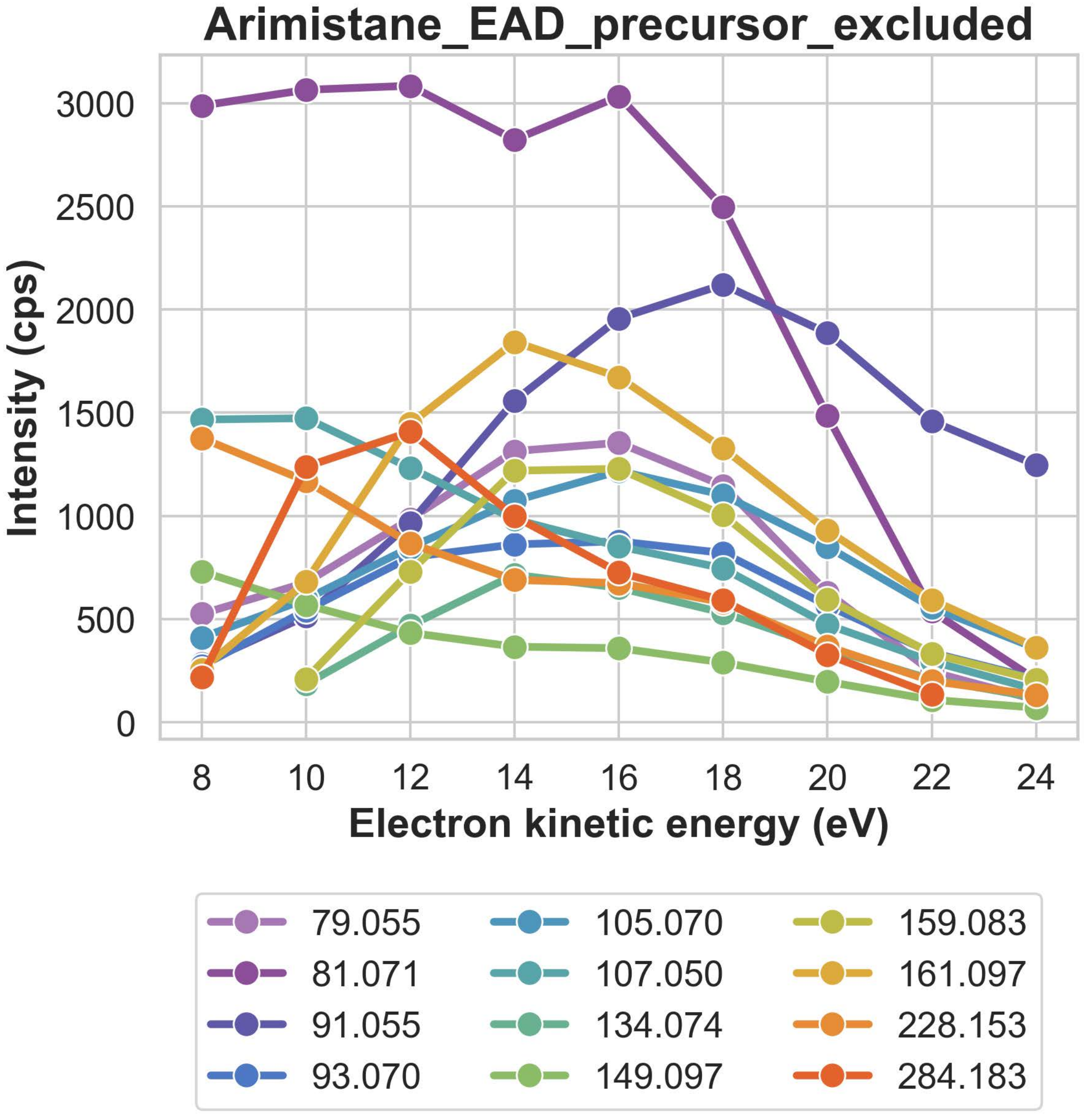
Arimistane_EAD_precursor_excluded
Intensity (cps)
3000
2500
2000
1500
1000
500
0
8
10
12
14
16
18
20
22
24
Electron kinetic energy (eV)
79.055
81.071
91.055
93.070
105.070
107.050
134.074
149.097
159.083
161.097
228.153
284.183

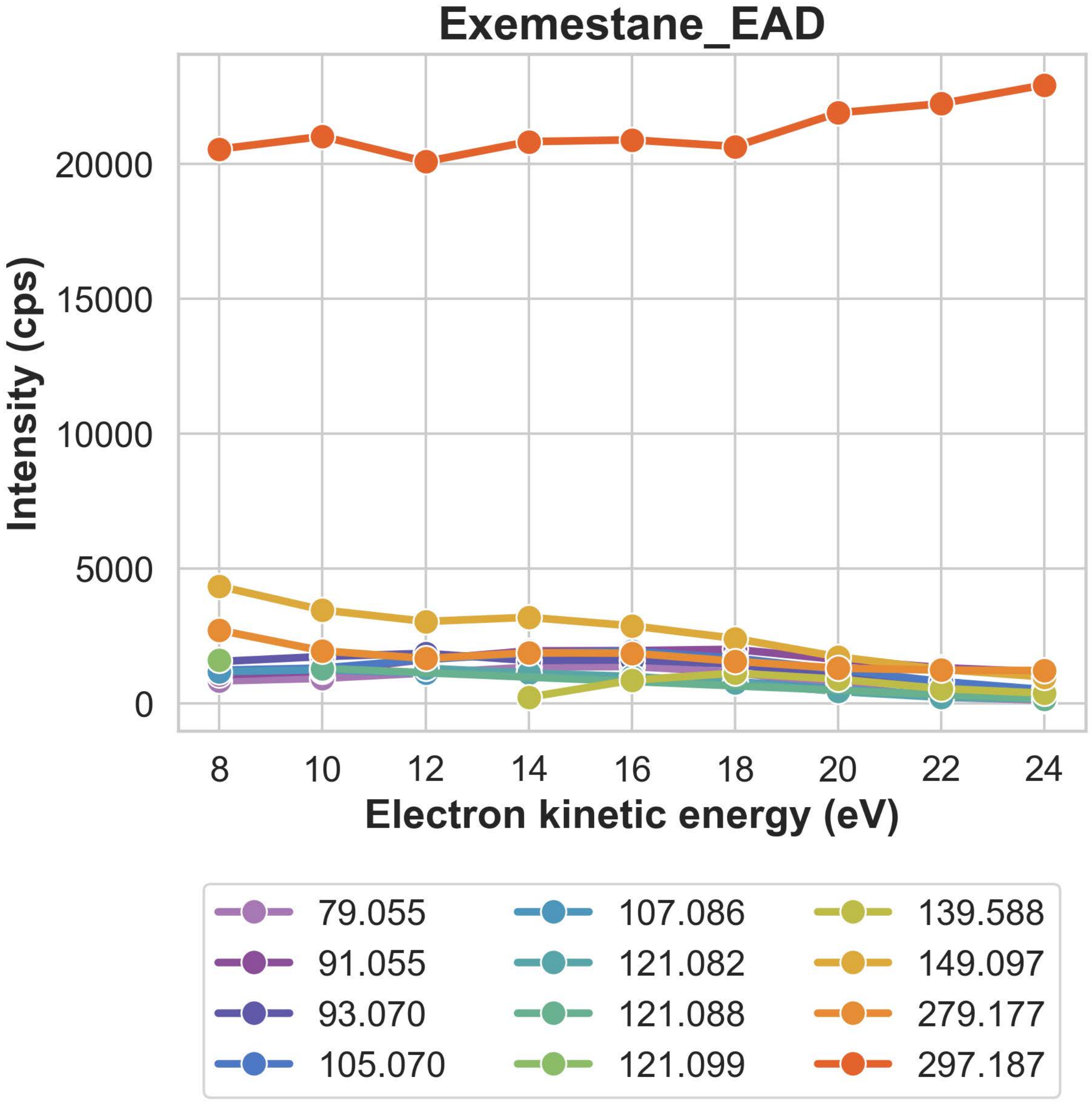
Exemestane_EAD
Intensity (cps)
20000
15000
10000
5000
0
8
10
12
14
16
18
20
22
24
Electron kinetic energy (eV)
79.055
91.055
93.070
105.070
107.086
121.082
121.088
121.099
139.588
149.097
279.177
297.187

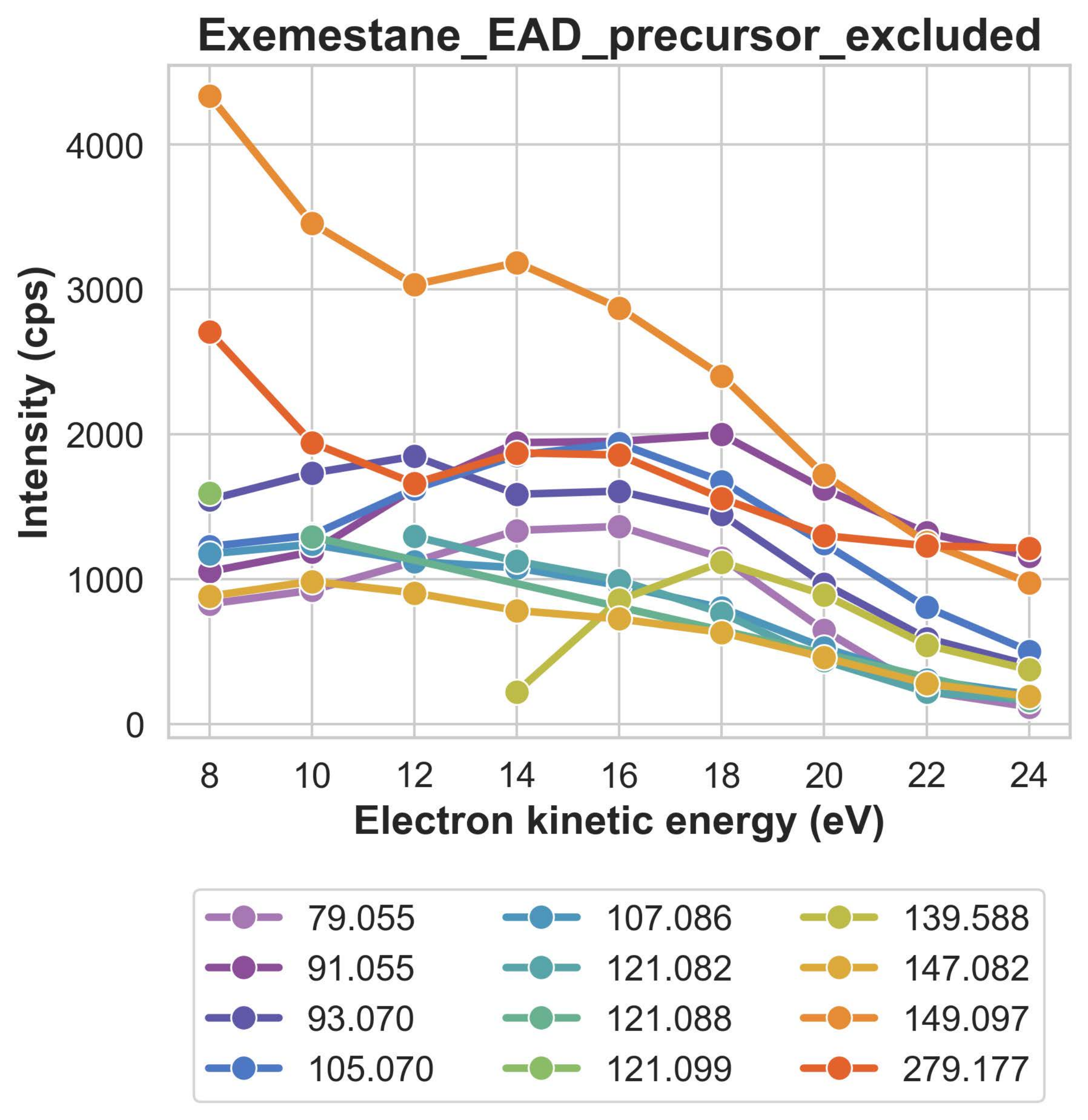

Exemestane_EAD_precursor_excluded
Intensity (cps)
4000
3000
2000
1000
0
8
10
12
14
16
18
20
22
24
Electron kinetic energy (eV)
79.055
91.055
93.070
105.070
107.086
121.082
121.088
121.099
139.588
147.082
149.097
279.177

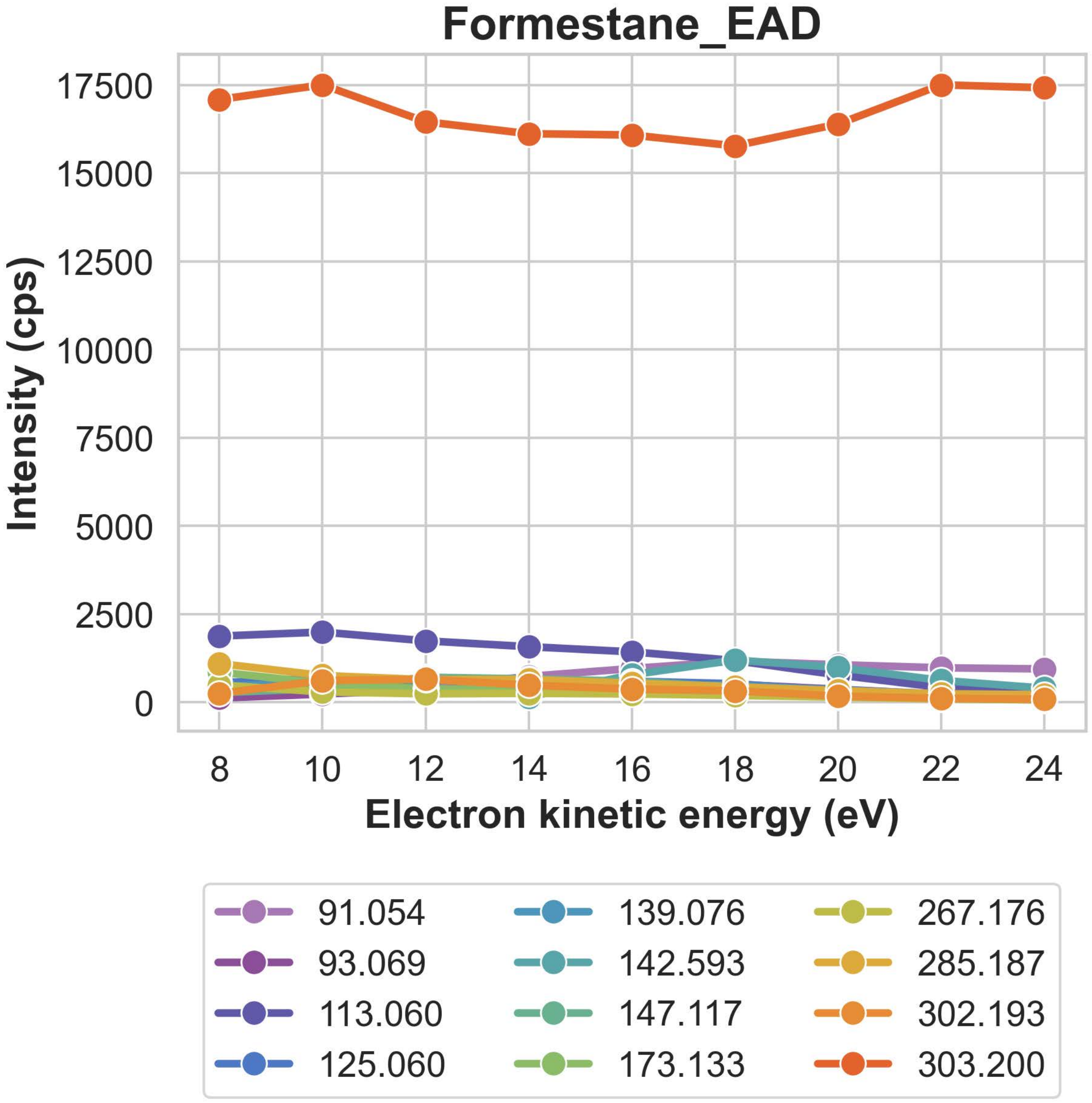

Formestane_EAD
Intensity (cps)
17500
15000
12500
10000
7500
5000
2500
0
8
10
12
14
16
18
20
22
24
Electron kinetic energy (eV)
91.054
93.069
113.060
125.060
139.076
142.593
147.117
173.133
267.176
285.187
302.193
303.200

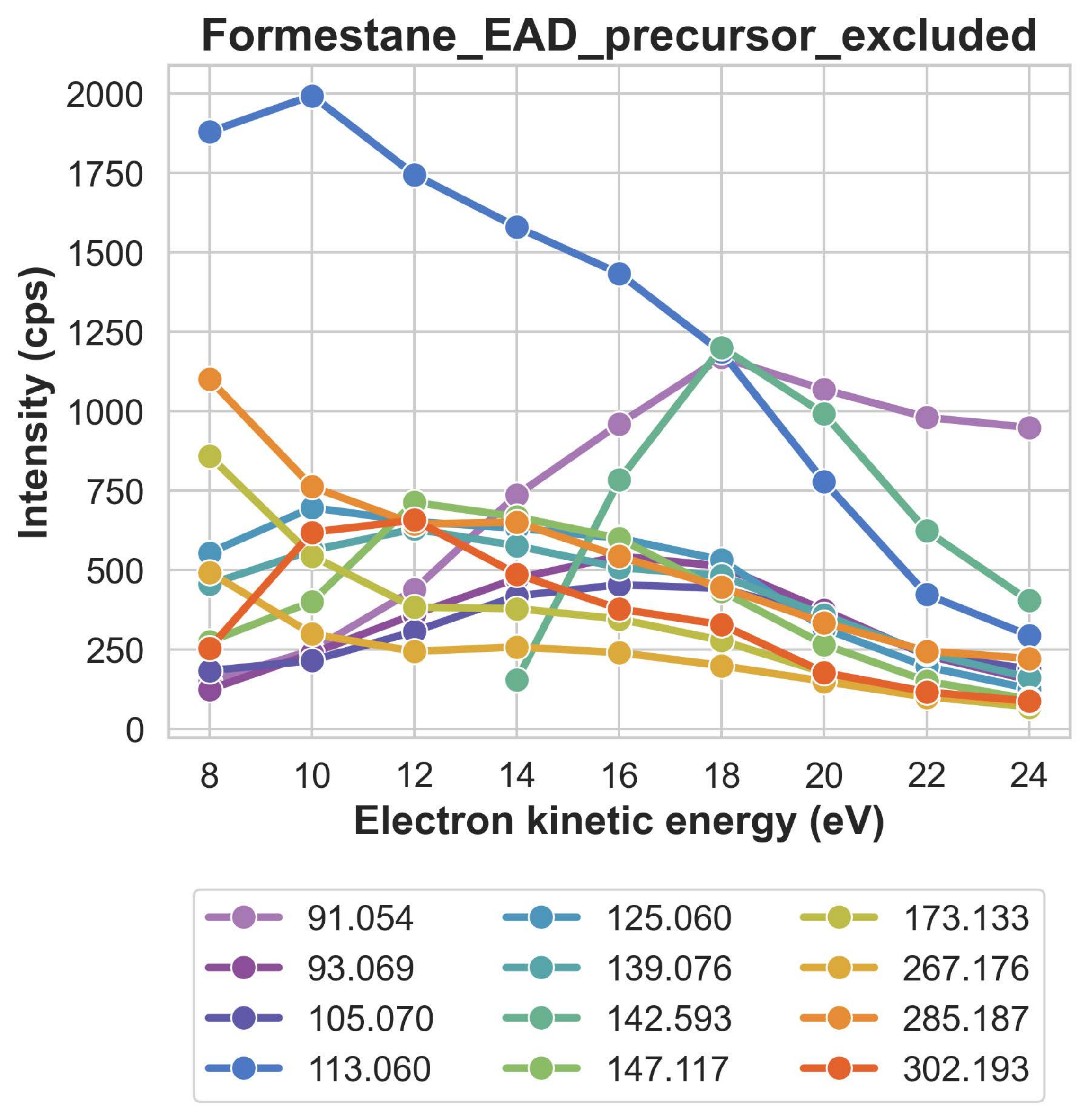

Formestane_EAD_precursor_excluded
Intensity (cps)
2000
1750
1500
1250
1000
750
500
250
0
8
10
12
14
16
18
20
22
24
Electron kinetic energy (eV)
91.054
93.069
105.070
113.060
125.060
139.076
142.593
147.117
173.133
267.176
285.187
302.193

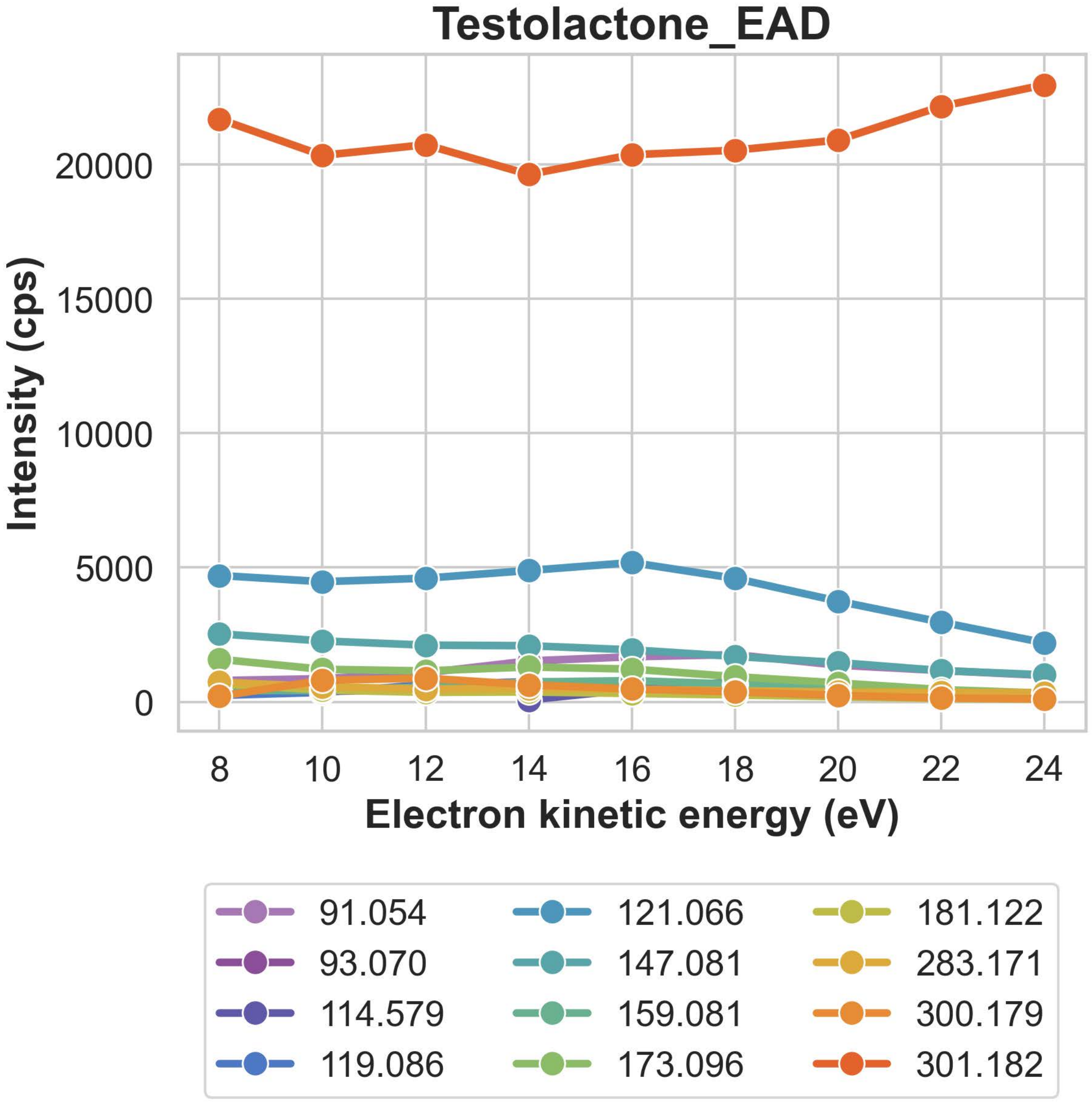

Testolactone_EAD
Intensity (cps)
20000
15000
10000
5000
0
8
10
12
14
16
18
20
22
24
Electron kinetic energy (eV)
91.054
93.070
114.579
119.086
121.066
147.081
159.081
173.096
181.122
283.171
300.179
301.182

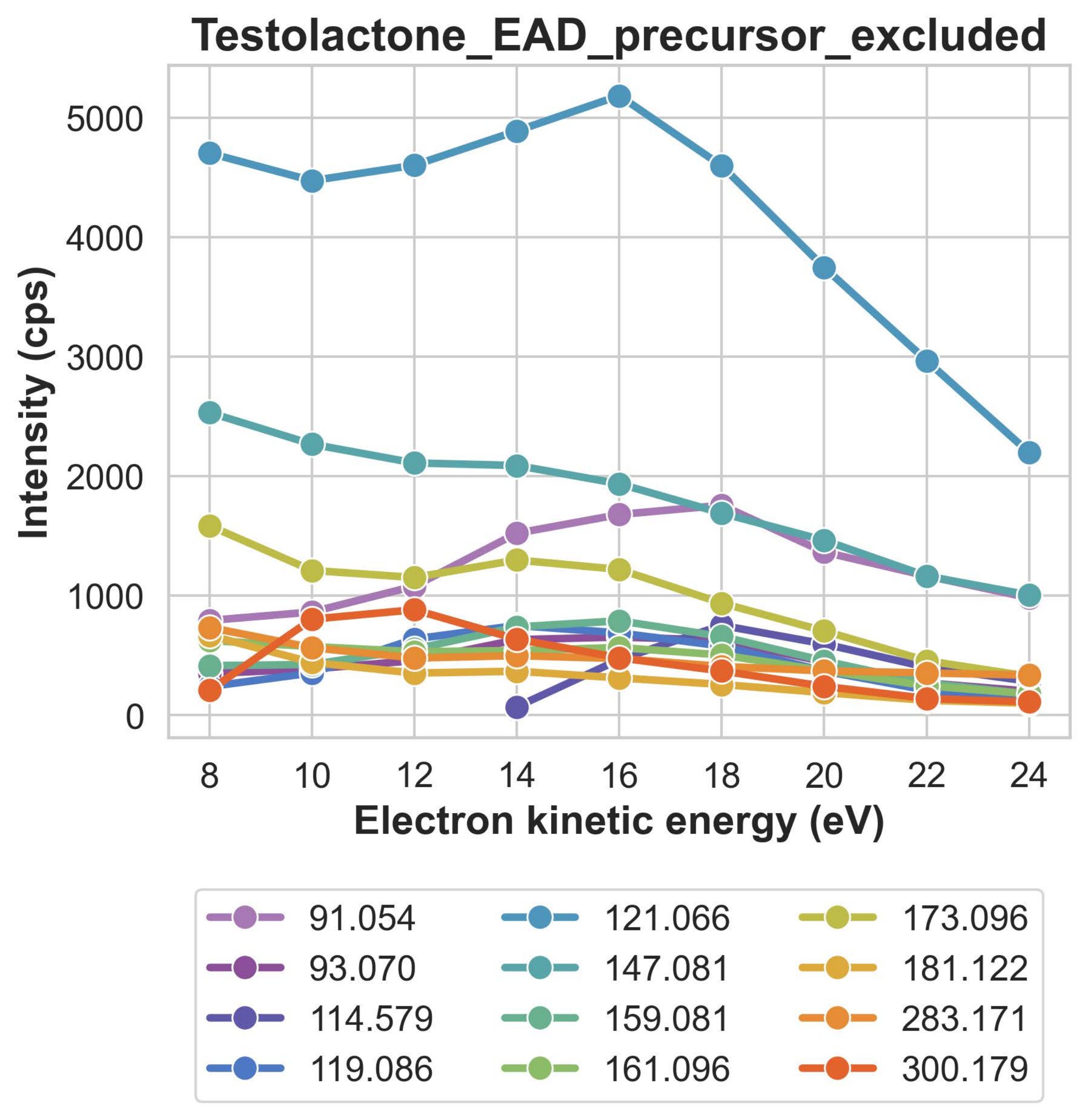

Testolactone_EAD_precursor_excluded
Intensity (cps)
5000
4000
3000
2000
1000
0
8
10
12
14
16
18
20
22
24
Electron kinetic energy (eV)
91.054
93.070
114.579
119.086
121.066
147.081
159.081
161.096
173.096
181.122
283.171
300.179

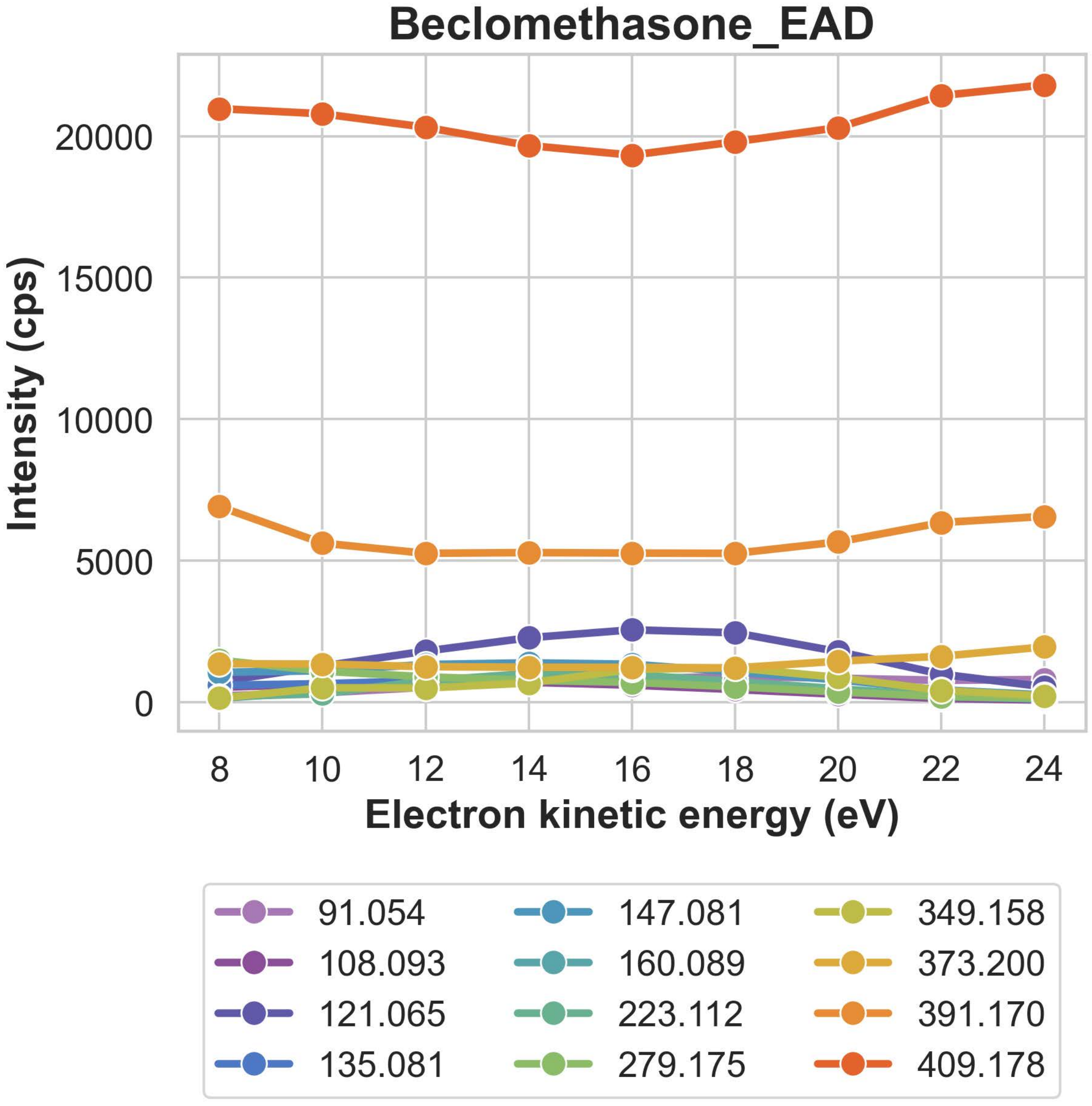

Beclomethasone_EAD
Intensity (cps)
20000
15000
10000
5000
0
8
10
12
14
16
18
20
22
24
Electron kinetic energy (eV)
91.054
108.093
121.065
135.081
147.081
160.089
223.112
279.175
349.158
373.200
391.170
409.178

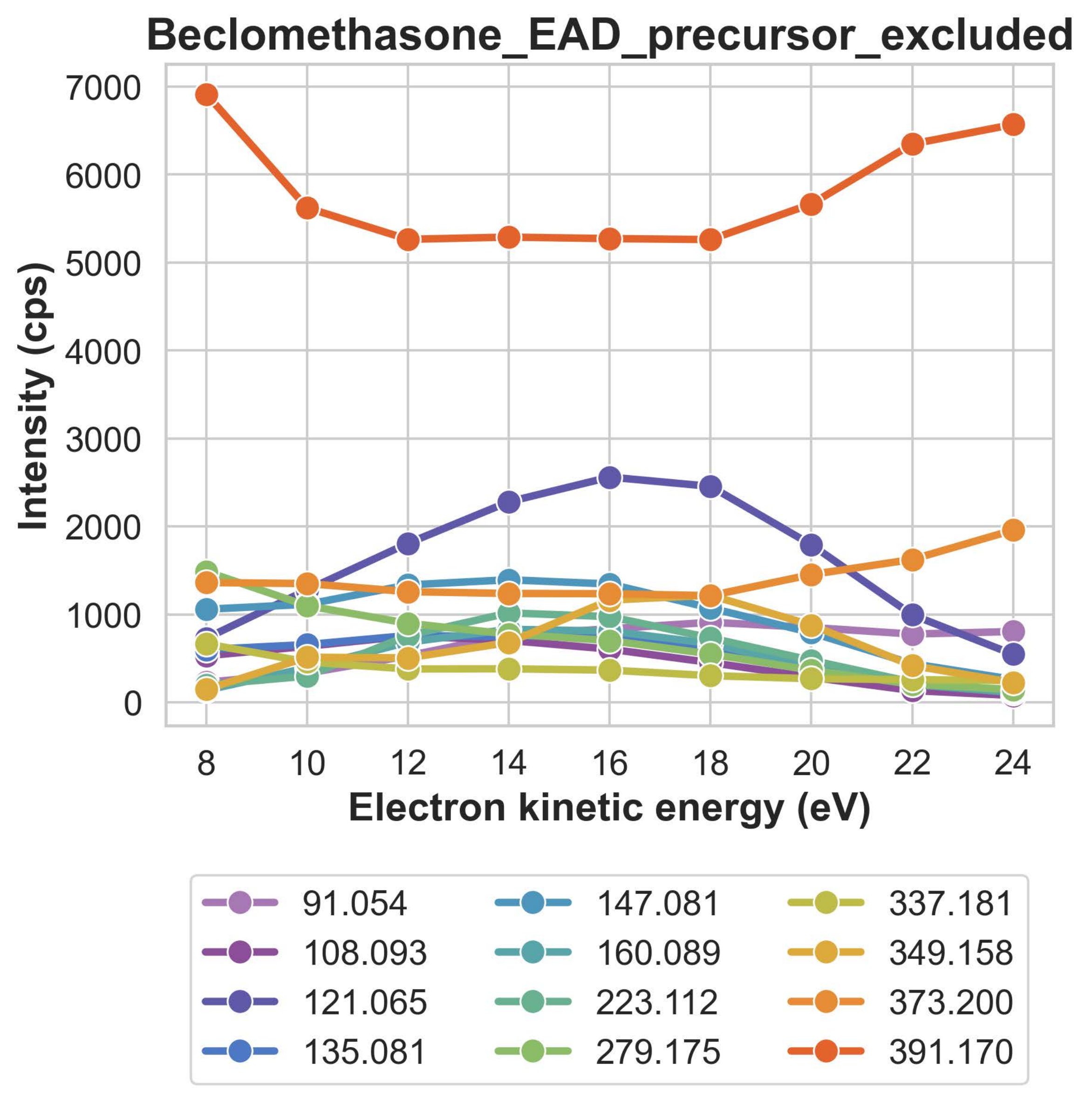
Beclomethasone_EAD_precursor_excluded
Intensity (cps)
7000
6000
5000
4000
3000
2000
1000
0
8
10
12
14
16
18
20
22
24
Electron kinetic energy (eV)
91.054
108.093
121.065
135.081
147.081
160.089
223.112
279.175
337.181
349.158
373.200
391.170

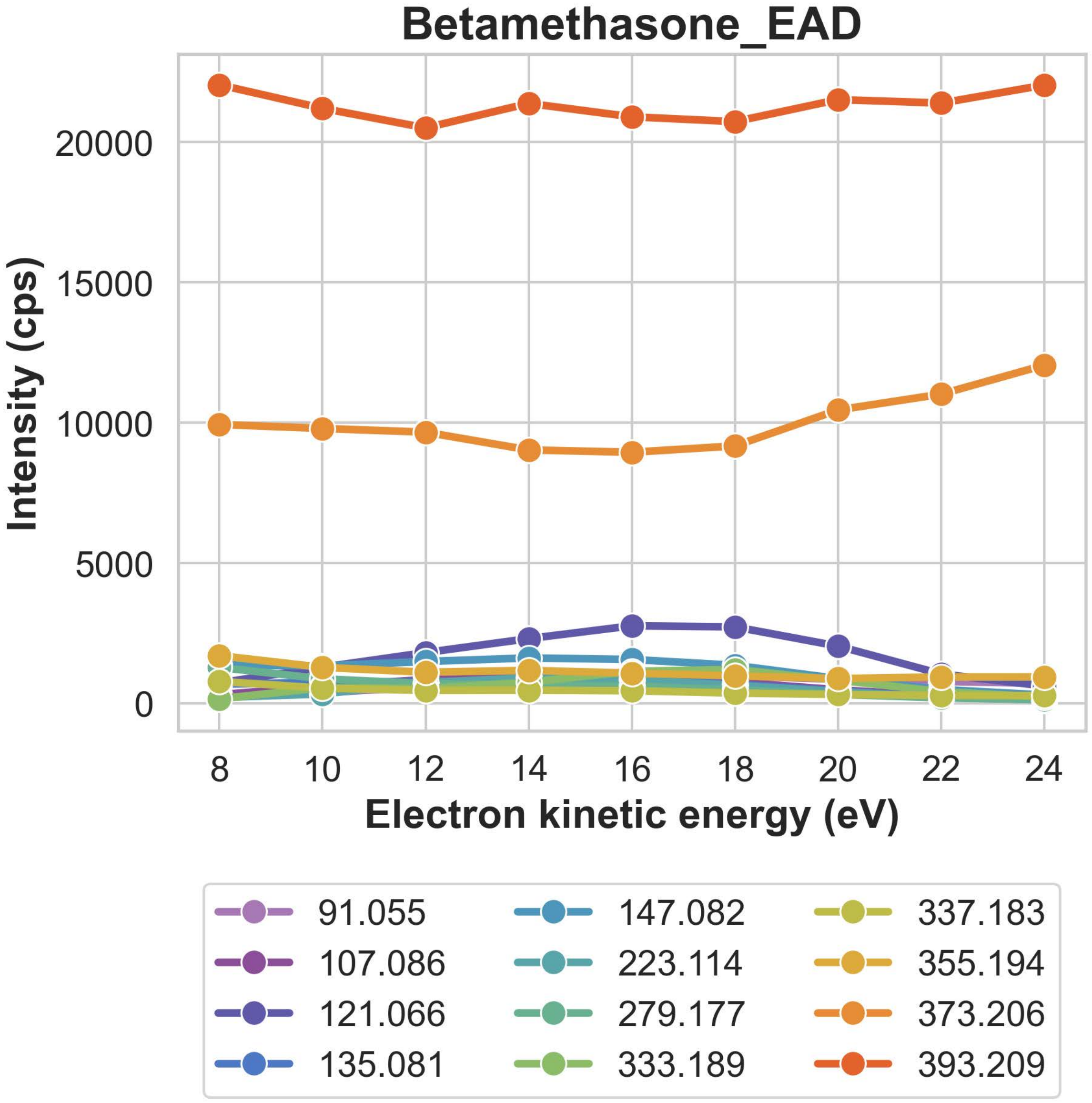
Betamethasone_EAD
Intensity (cps)
20000
15000
10000
5000
0
8
10
12
14
16
18
20
22
24
Electron kinetic energy (eV)
91.055
107.086
121.066
135.081
147.082
223.114
279.177
333.189
337.183
355.194
373.206
393.209

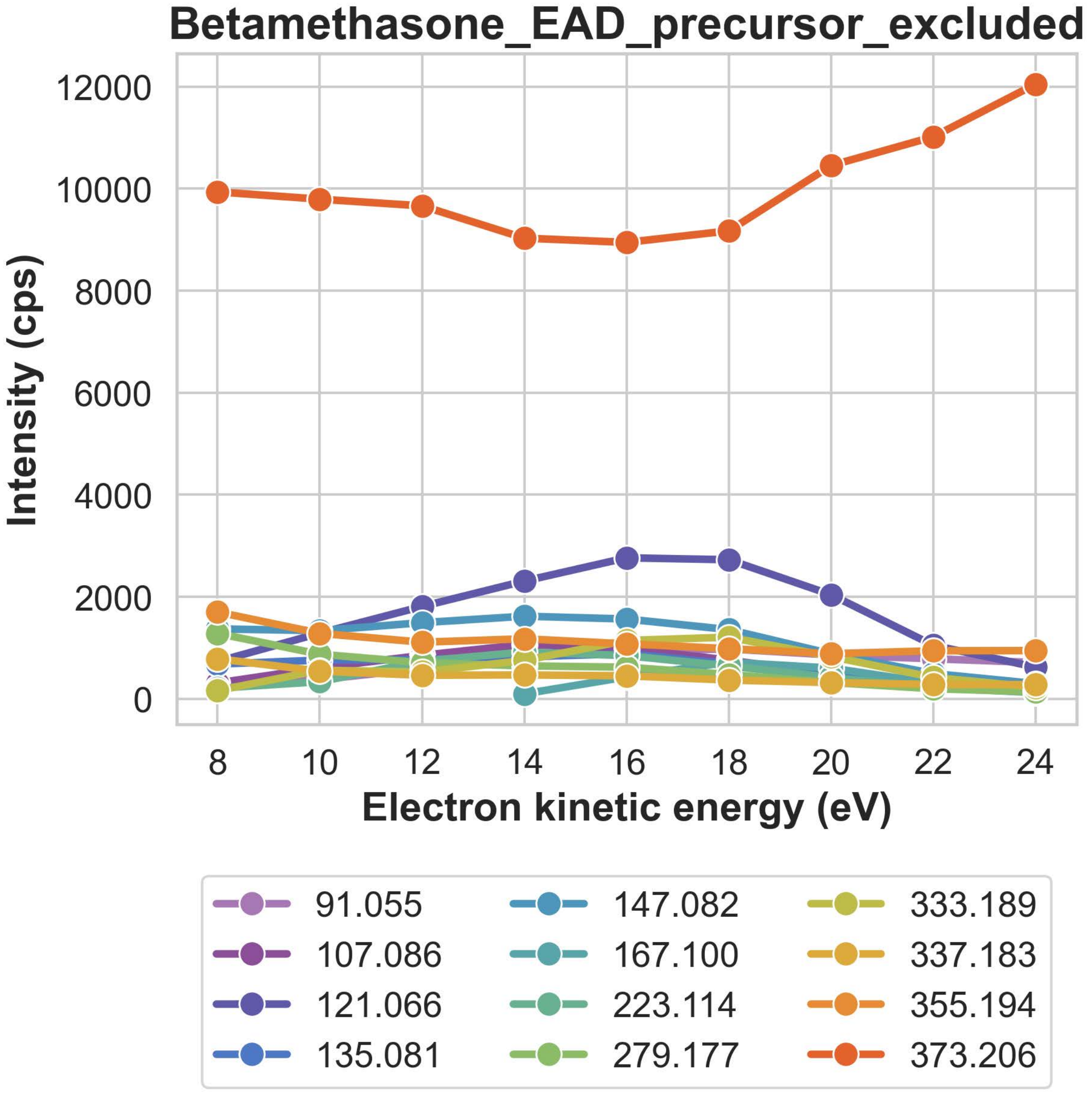
Betamethasone_EAD_precursor_excluded
Intensity (cps)
12000
10000
8000
6000
4000
2000
0
8
10
12
14
16
18
20
22
24
Electron kinetic energy (eV)
91.055
107.086
121.066
135.081
147.082
167.100
223.114
279.177
333.189
337.183
355.194
373.206

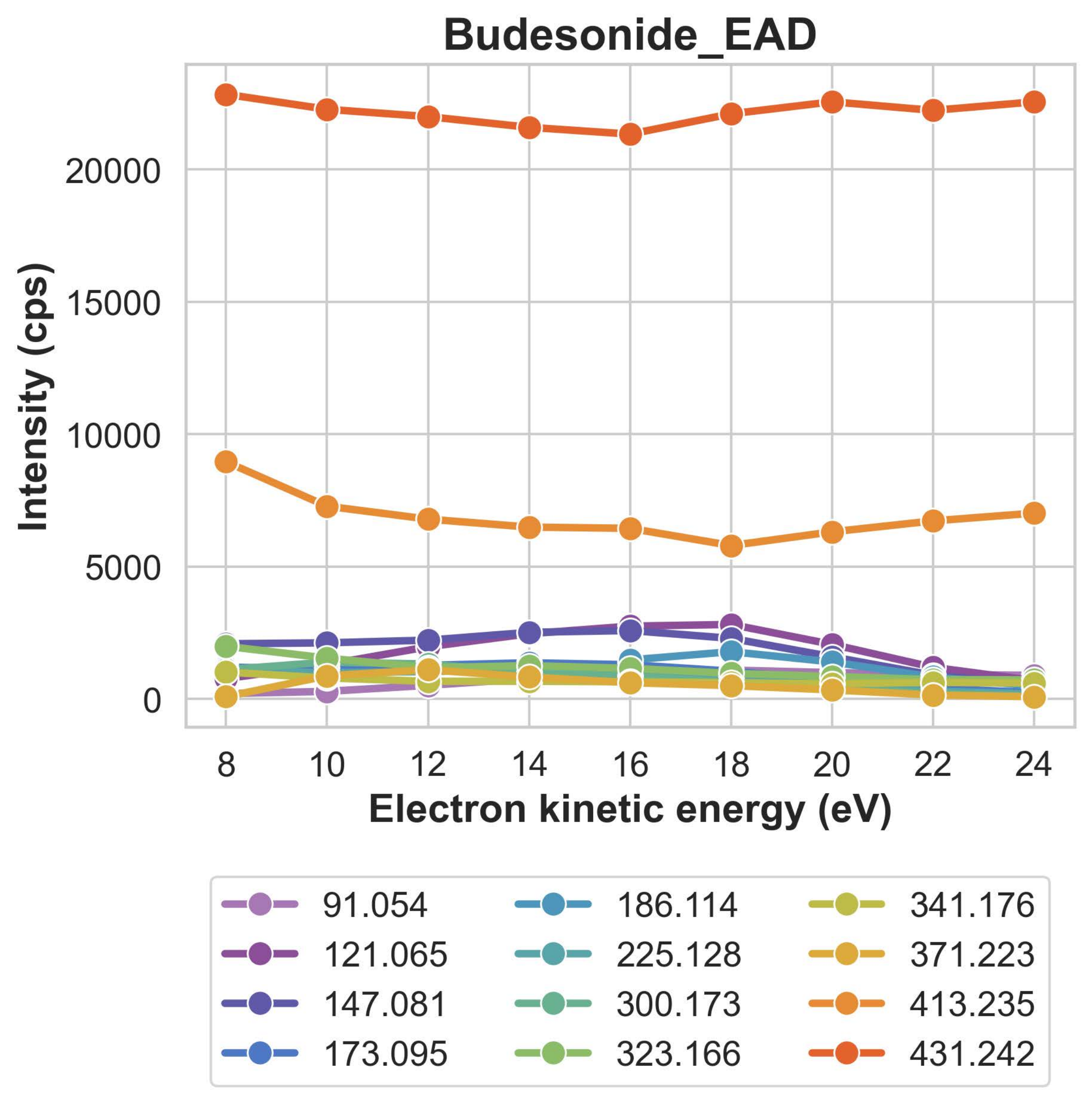
Budesonide_EAD
Intensity (cps)
20000
15000
10000
5000
0
8
10
12
14
16
18
20
22
24
Electron kinetic energy (eV)
91.054
121.065
147.081
173.095
186.114
225.128
300.173
323.166
341.176
371.223
413.235
431.242

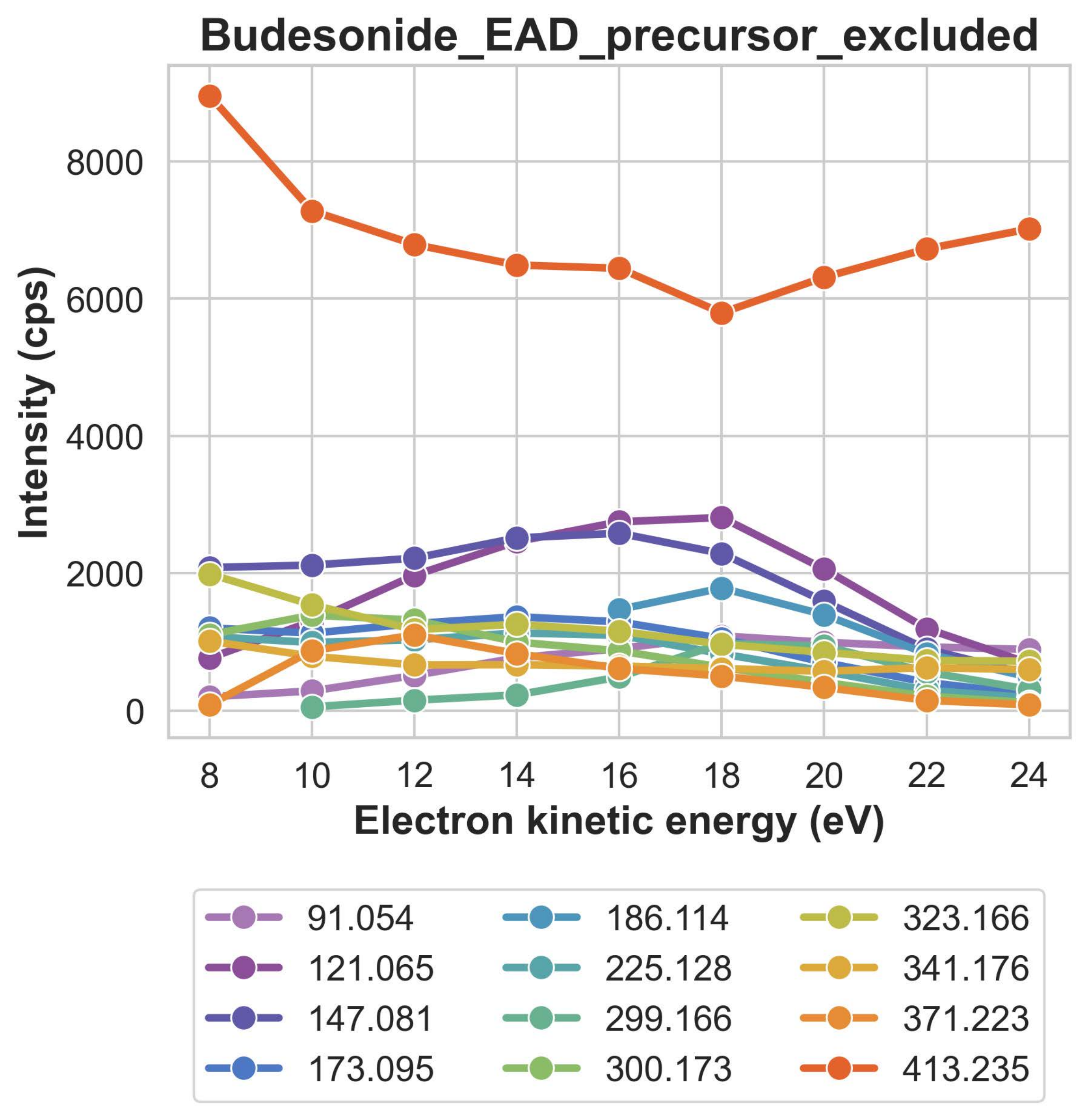
Budesonide_EAD_precursor_excluded
Intensity (cps)
8000
6000
4000
2000
0
8
10
12
14
16
18
20
22
24
Electron kinetic energy (eV)
91.054
121.065
147.081
173.095
186.114
225.128
299.166
300.173
323.166
341.176
371.223
413.235

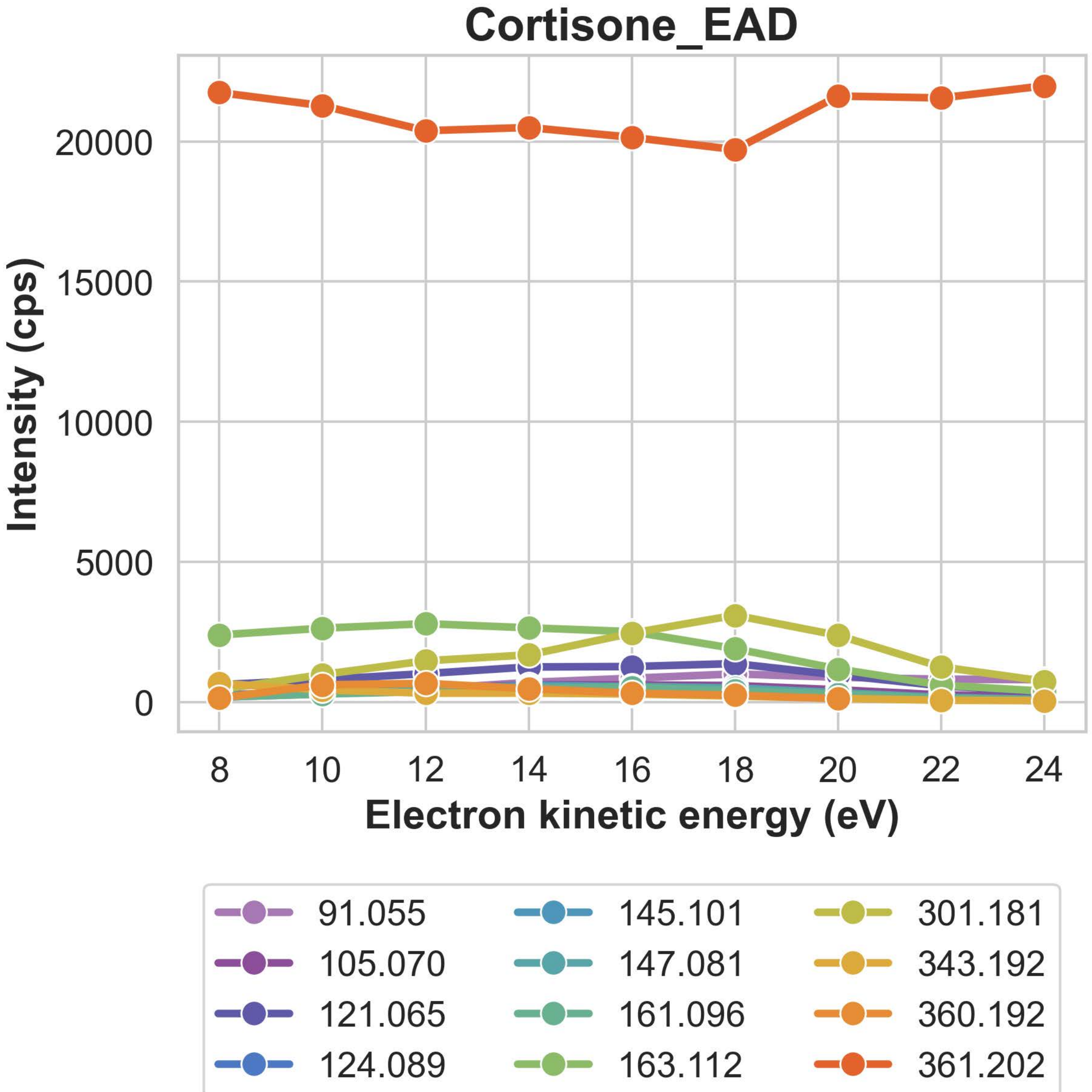
Cortisone_EAD
Intensity (cps)
20000
15000
10000
5000
0
8
10
12
14
16
18
20
22
24
Electron kinetic energy (eV)
91.055
105.070
121.065
124.089
145.101
147.081
161.096
163.112
301.181
343.192
360.192
361.202

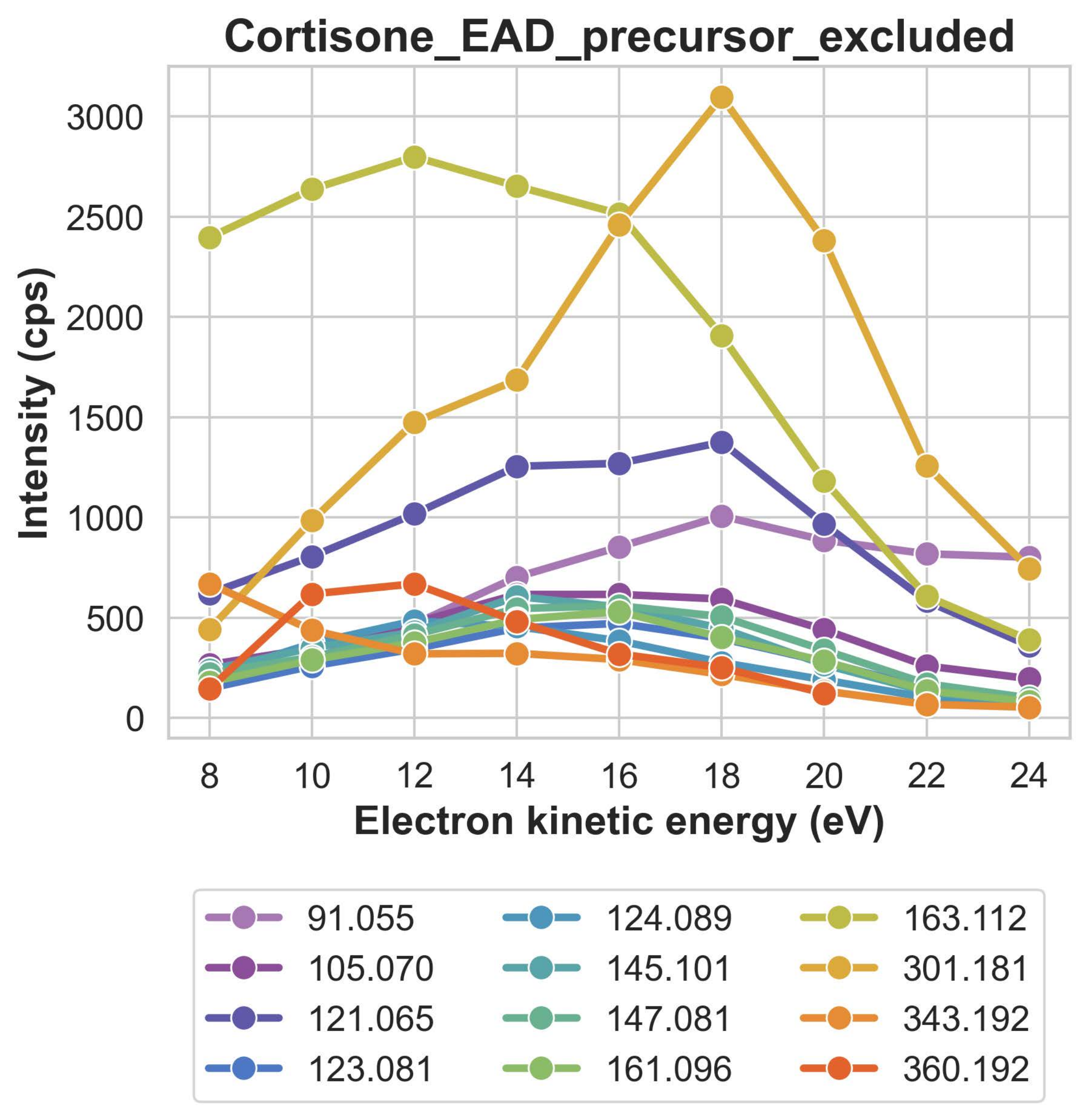
Cortisone_EAD_precursor_excluded
Intensity (cps)
0
500
1000
1500
2000
2500
3000
8
10
12
14
16
18
20
22
24
Electron kinetic energy (eV)
91.055
105.070
121.065
123.081
124.089
145.101
147.081
161.096
163.112
301.181
343.192
360.192

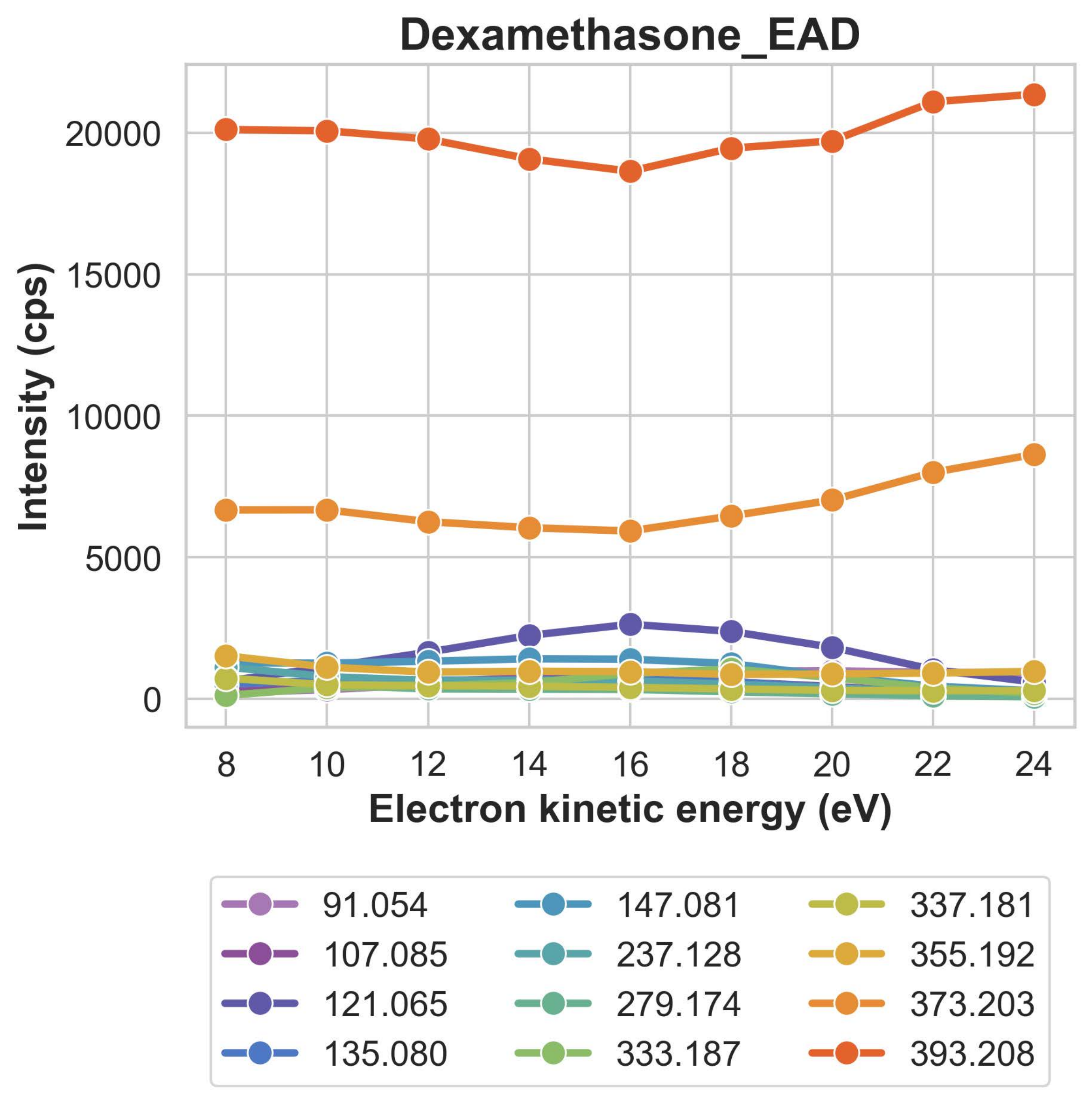
Dexamethasone_EAD
Intensity (cps)
20000
15000
10000
5000
0
8
10
12
14
16
18
20
22
24
Electron kinetic energy (eV)
91.054
107.085
121.065
135.080
147.081
237.128
279.174
333.187
337.181
355.192
373.203
393.208

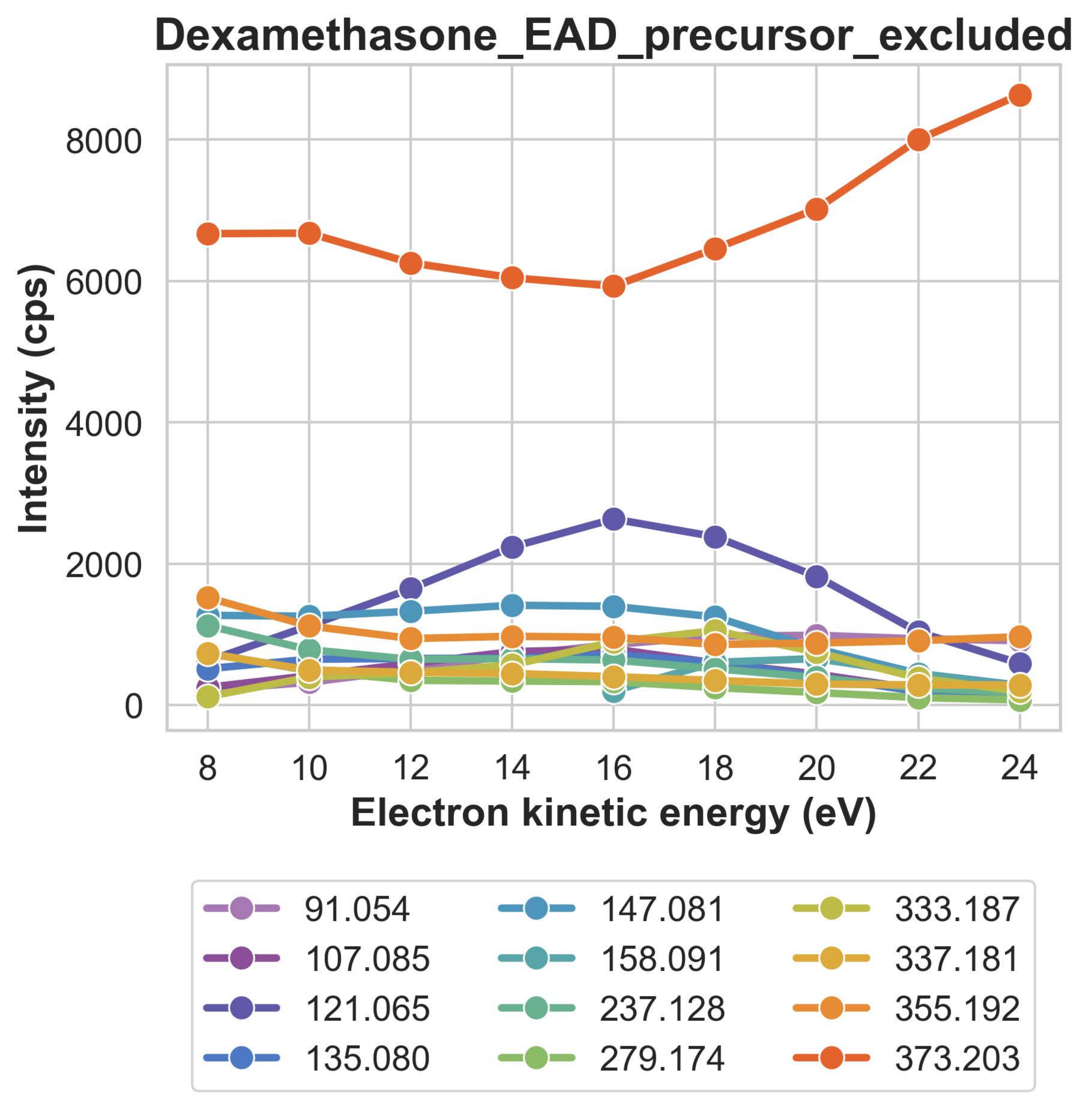

Dexamethasone_EAD_precursor_excluded
Intensity (cps)
8000
6000
4000
2000
0
8
10
12
14
16
18
20
22
24
Electron kinetic energy (eV)
91.054
107.085
121.065
135.080
147.081
158.091
237.128
279.174
333.187
337.181
355.192
373.203

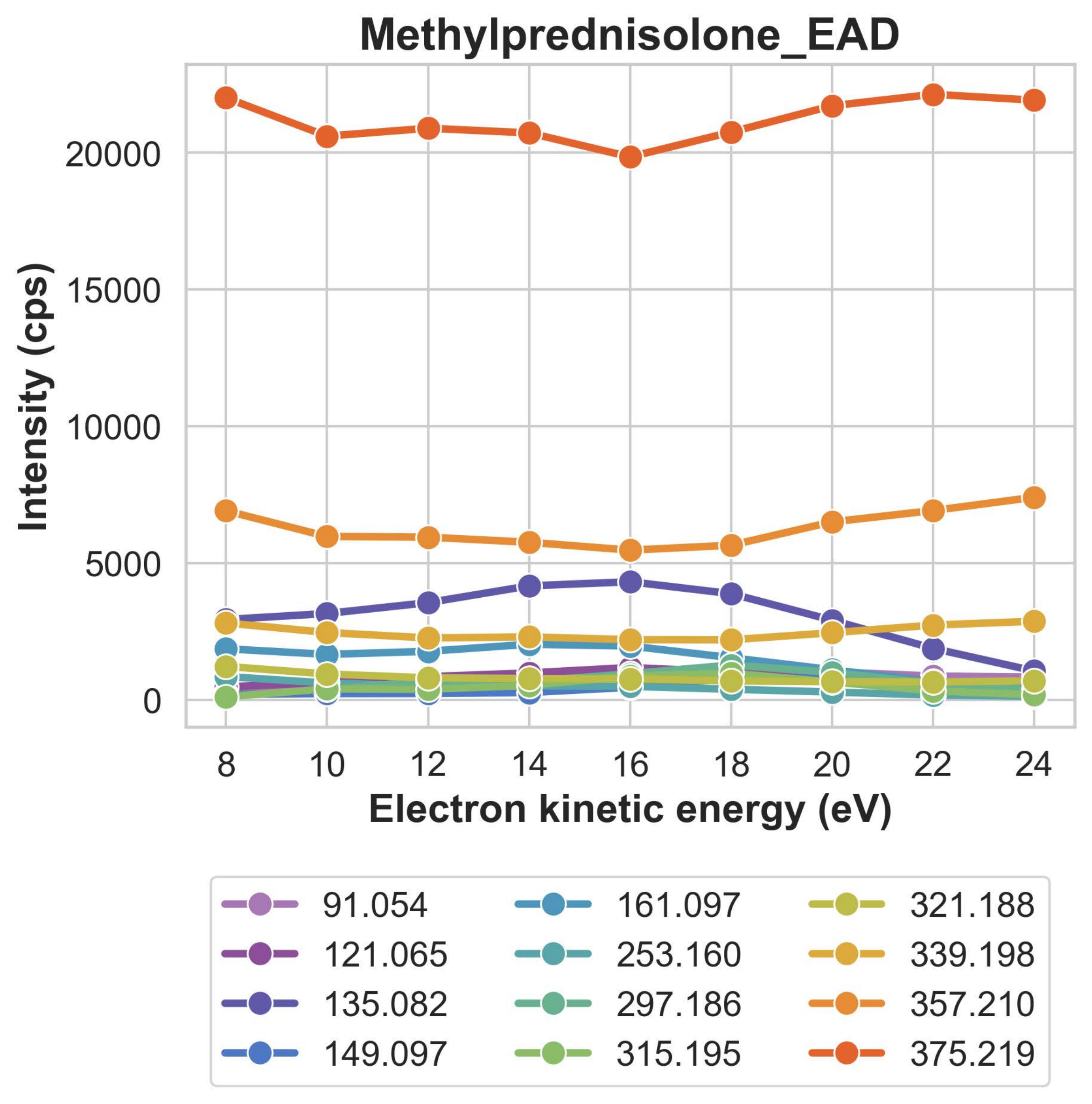

Methylprednisolone_EAD
Intensity (cps)
20000
15000
10000
5000
0
8
10
12
14
16
18
20
22
24
Electron kinetic energy (eV)
91.054
121.065
135.082
149.097
161.097
253.160
297.186
315.195
321.188
339.198
357.210
375.219

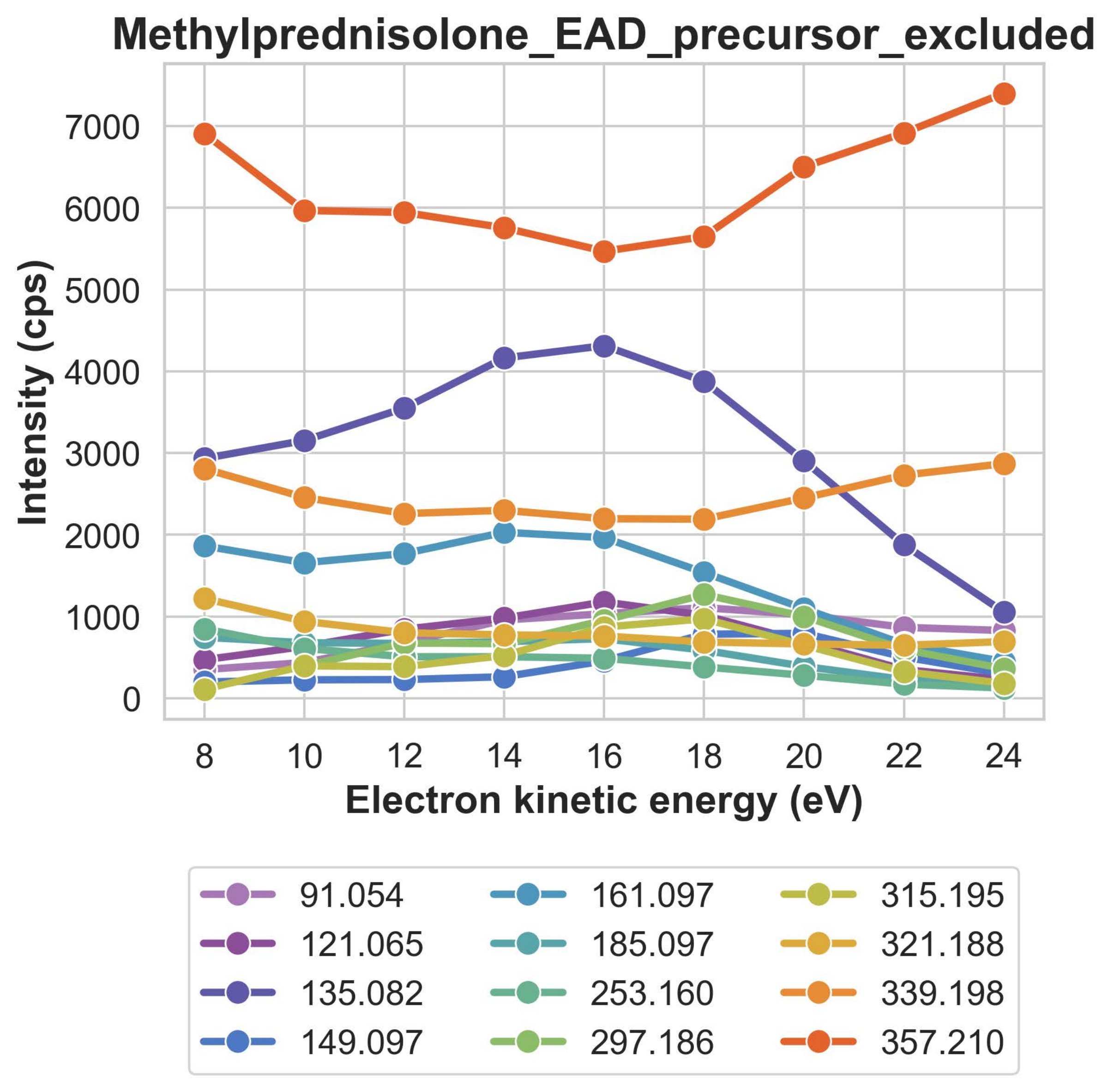
Methylprednisolone_EAD_precursor_excluded
Intensity (cps)
0
1000
2000
3000
4000
5000
6000
7000
8
10
12
14
16
18
20
22
24
Electron kinetic energy (eV)
91.054
121.065
135.082
149.097
161.097
185.097
253.160
297.186
315.195
321.188
339.198
357.210

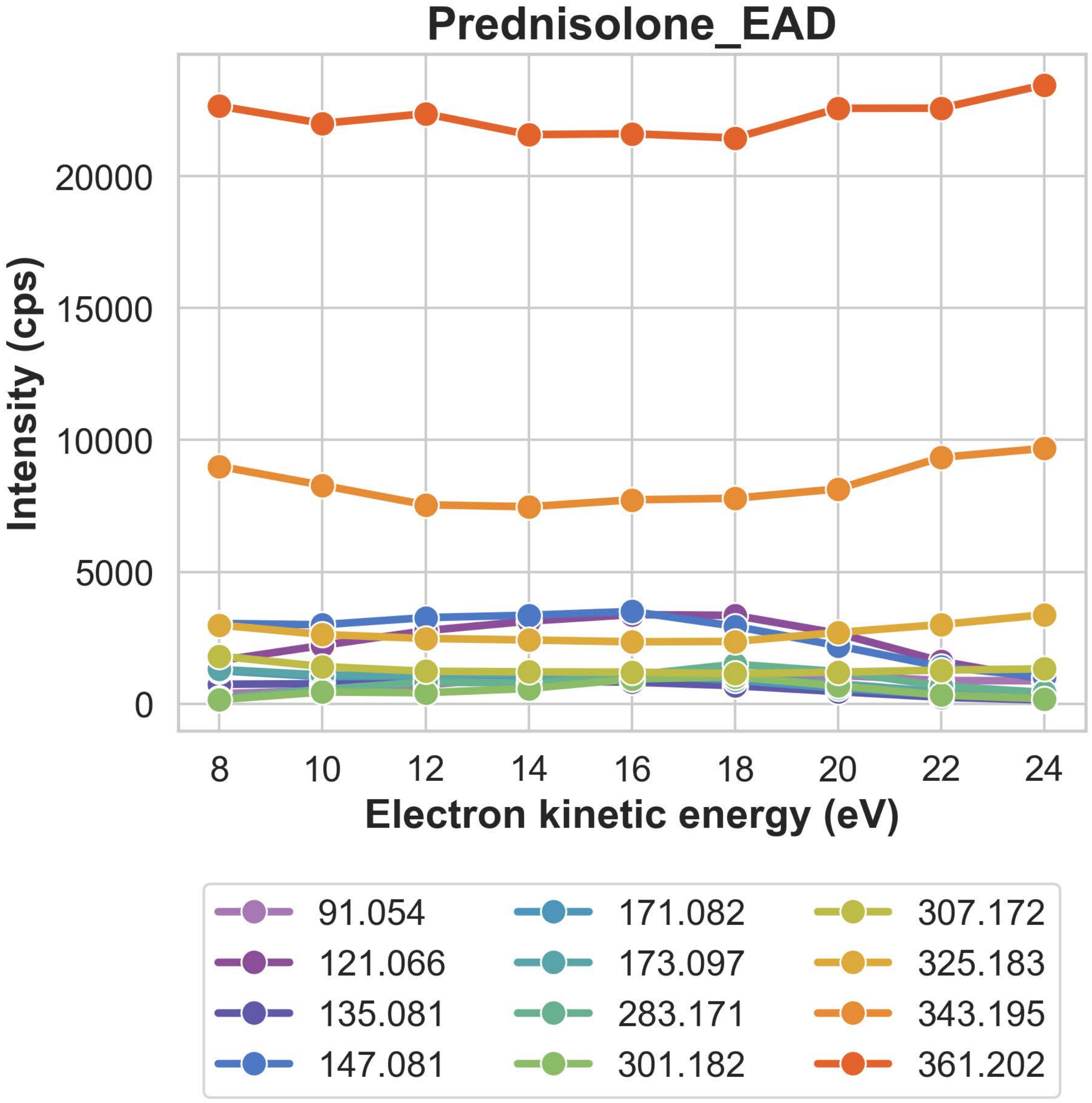

Prednisolone_EAD
Intensity (cps)
20000
15000
10000
5000
0
8
10
12
14
16
18
20
22
24
Electron kinetic energy (eV)
91.054
121.066
135.081
147.081
171.082
173.097
283.171
301.182
307.172
325.183
343.195
361.202

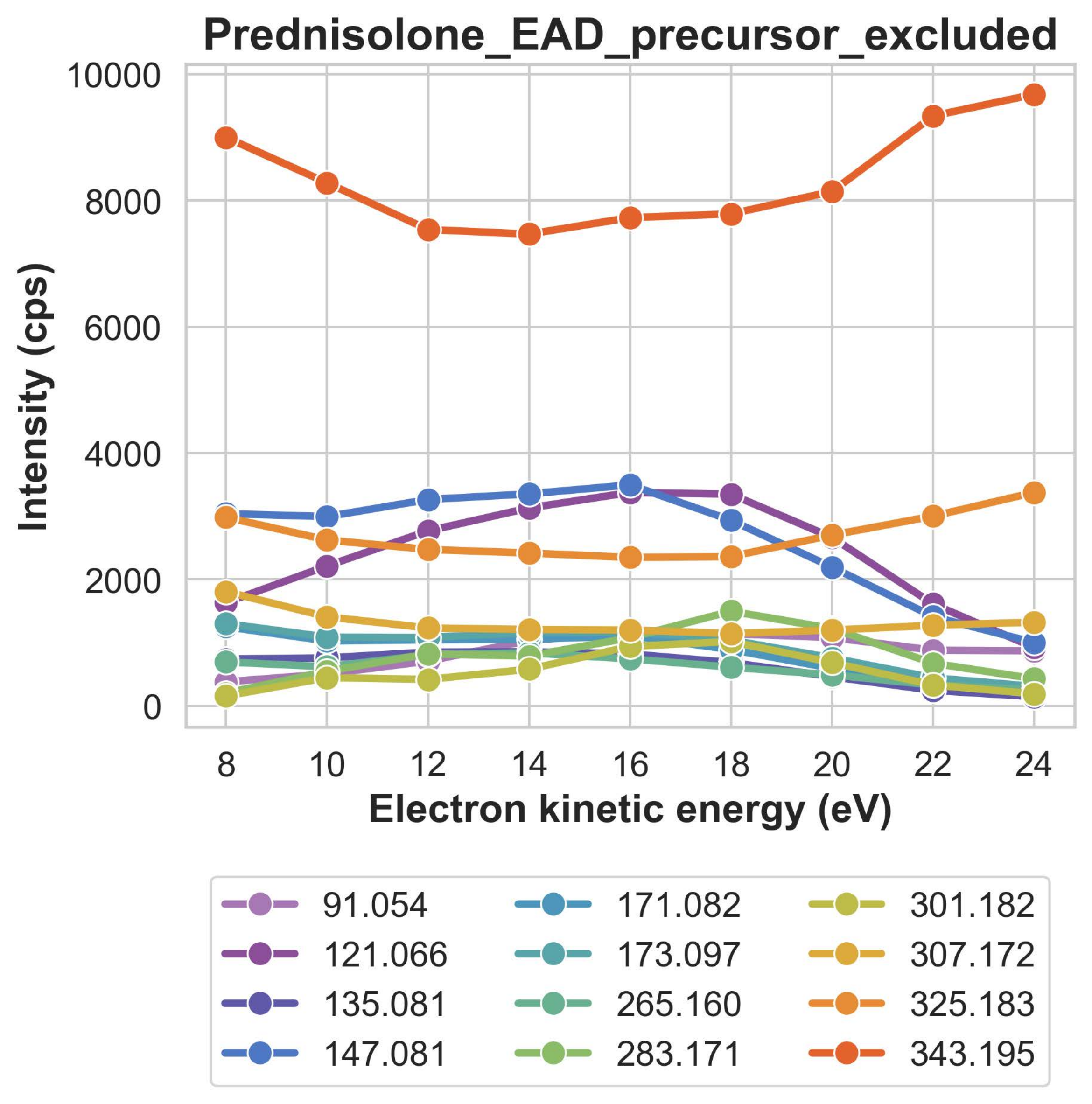

Prednisolone_EAD_precursor_excluded
Intensity (cps)
10000
8000
6000
4000
2000
0
8
10
12
14
16
18
20
22
24
Electron kinetic energy (eV)
91.054
121.066
135.081
147.081
171.082
173.097
265.160
283.171
301.182
307.172
325.183
343.195

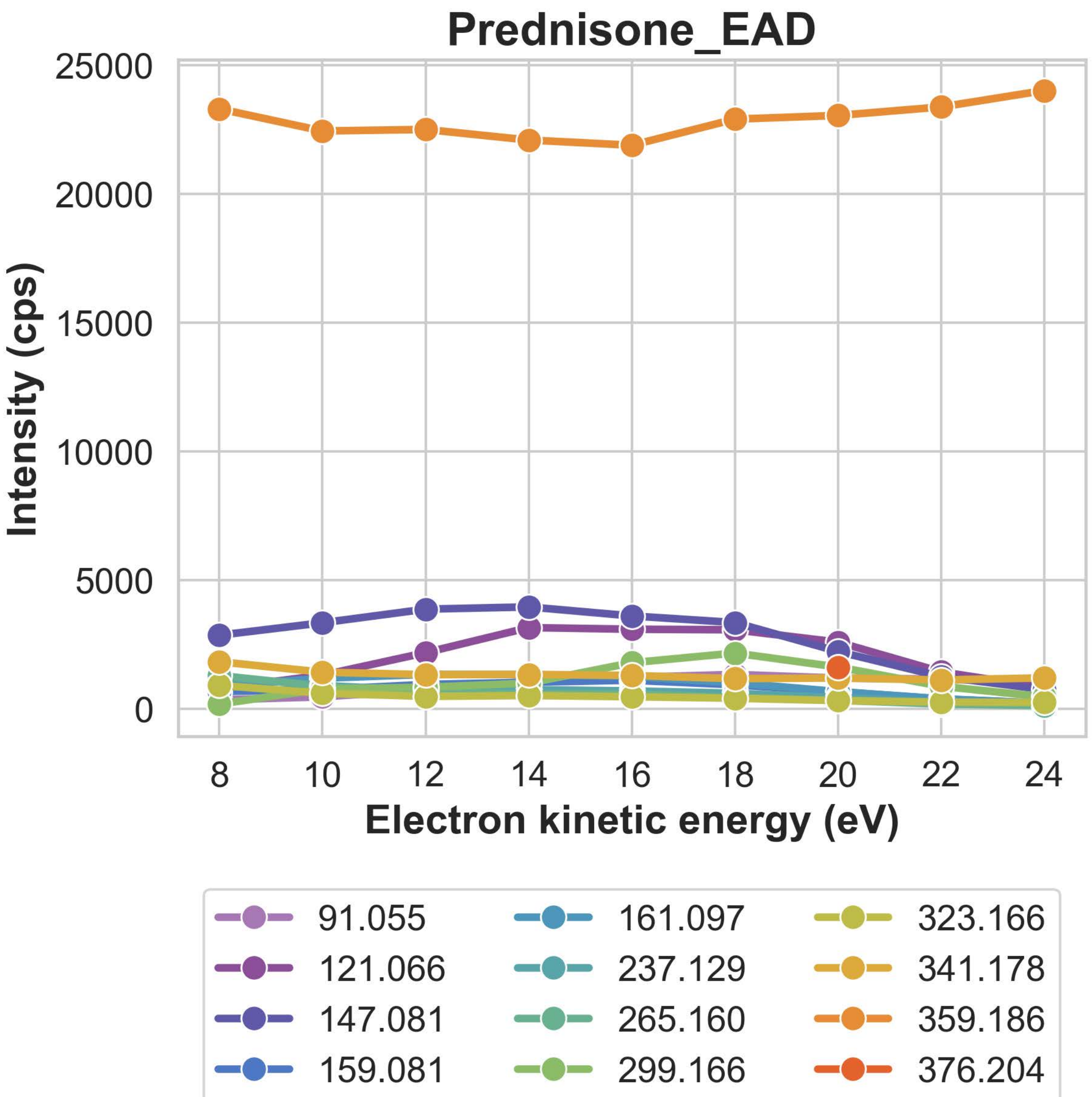
Prednisone_EAD
Intensity (cps)
25000
20000
15000
10000
5000
0
8
10
12
14
16
18
20
22
24
Electron kinetic energy (eV)
91.055
121.066
147.081
159.081
161.097
237.129
265.160
299.166
323.166
341.178
359.186
376.204

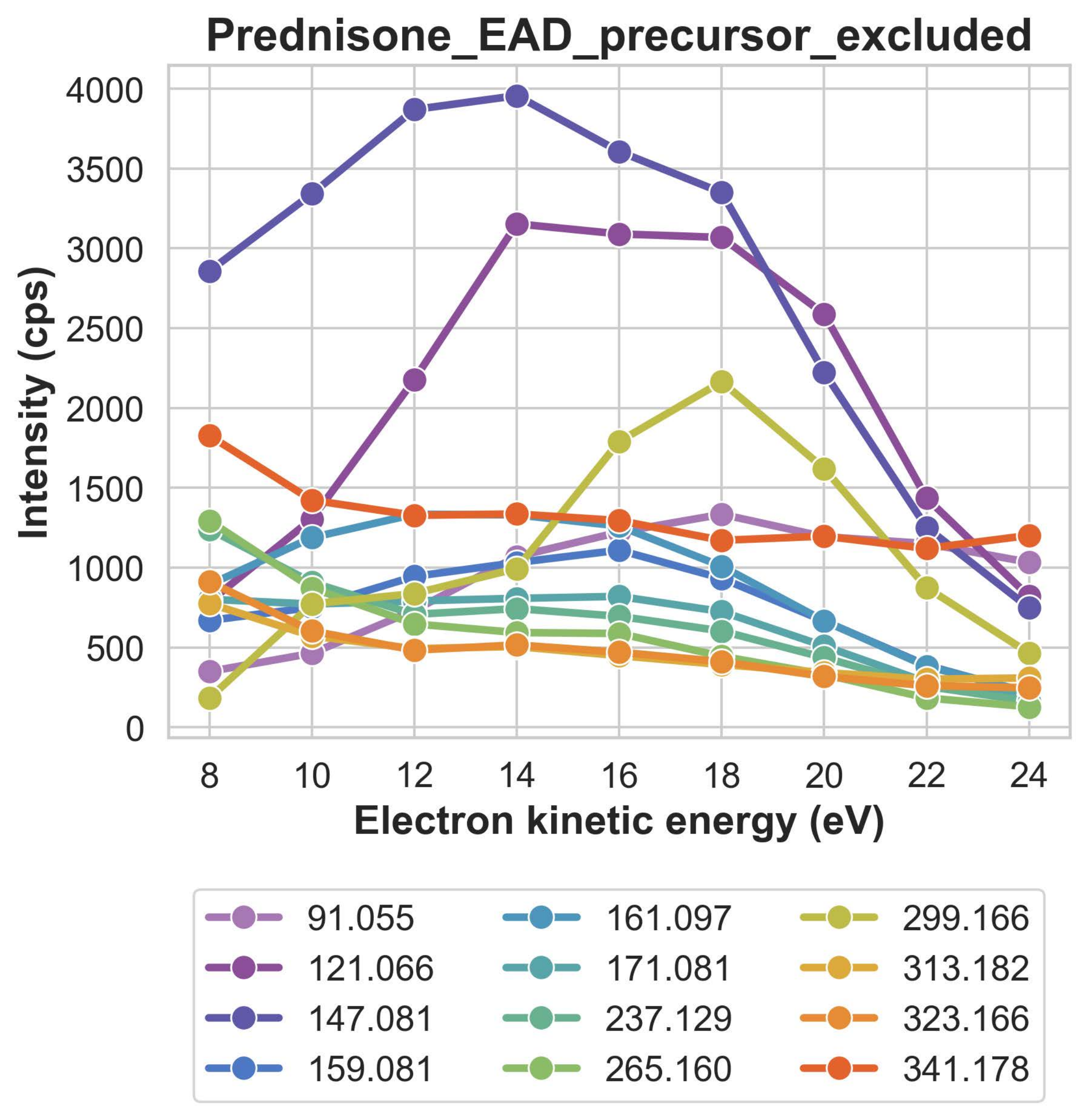

Prednisone_EAD_precursor_excluded
Intensity (cps)
0
500
1000
1500
2000
2500
3000
3500
4000
8
10
12
14
16
18
20
22
24
Electron kinetic energy (eV)
91.055
121.066
147.081
159.081
161.097
171.081
237.129
265.160
299.166
313.182
323.166
341.178

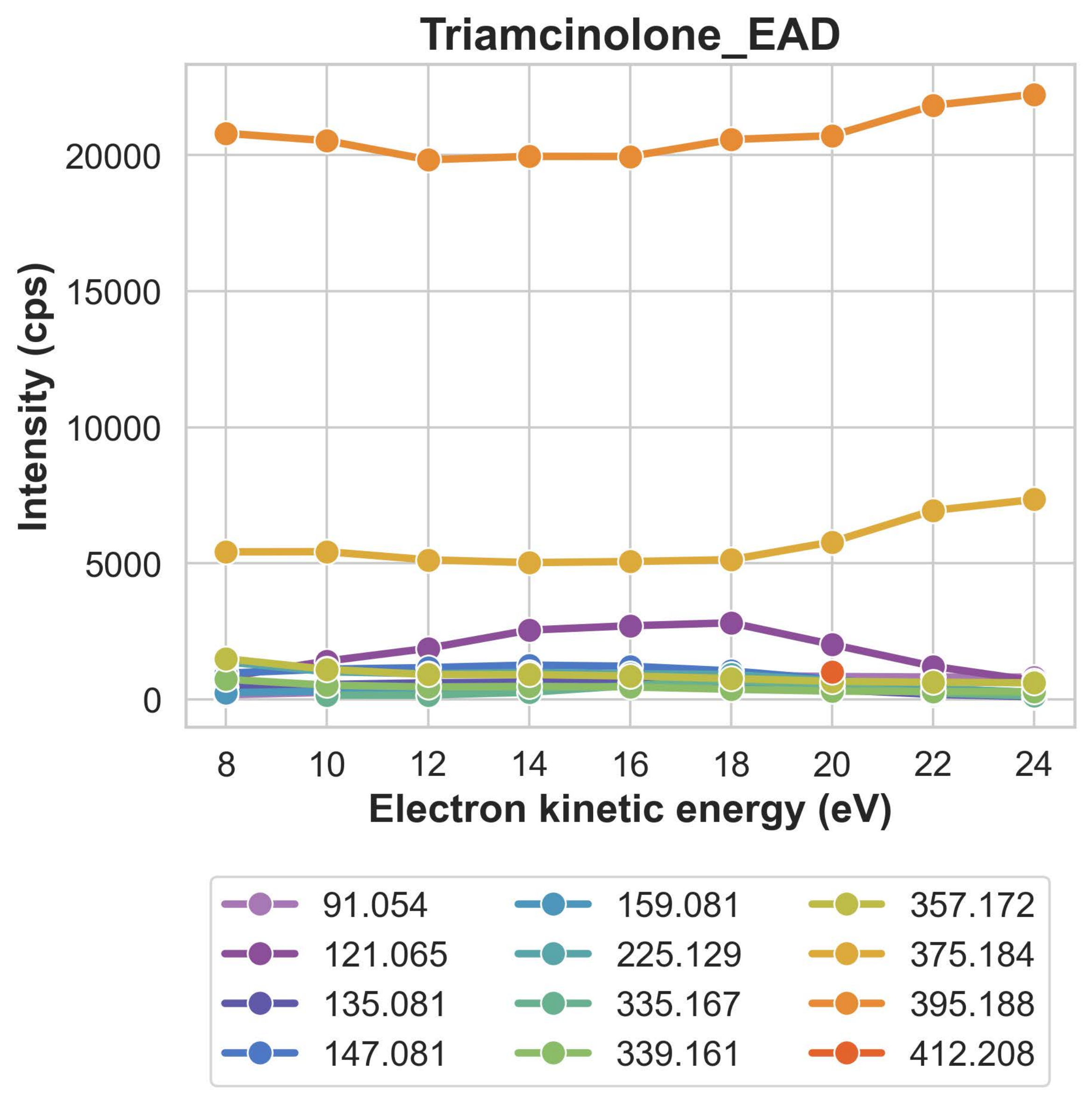
Triamcinolone_EAD
Intensity (cps)
20000
15000
10000
5000
0
8
10
12
14
16
18
20
22
24
Electron kinetic energy (eV)
91.054
121.065
135.081
147.081
159.081
225.129
335.167
339.161
357.172
375.184
395.188
412.208

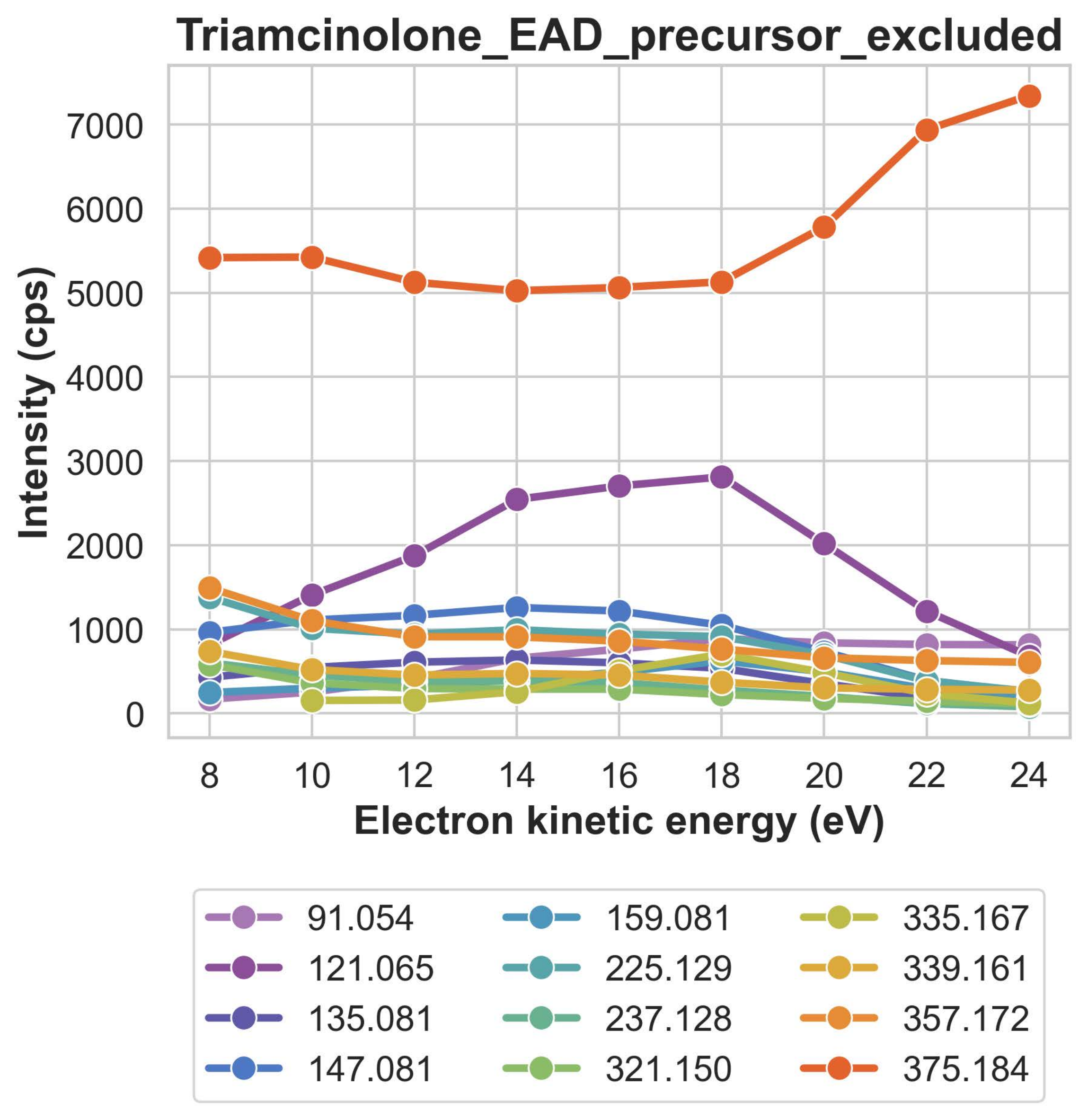

Triamcinolone_EAD_precursor_excluded
Intensity (cps)
0
1000
2000
3000
4000
5000
6000
7000
8
10
12
14
16
18
20
22
24
Electron kinetic energy (eV)
91.054
121.065
135.081
147.081
159.081
225.129
237.128
321.150
335.167
339.161
357.172
375.184

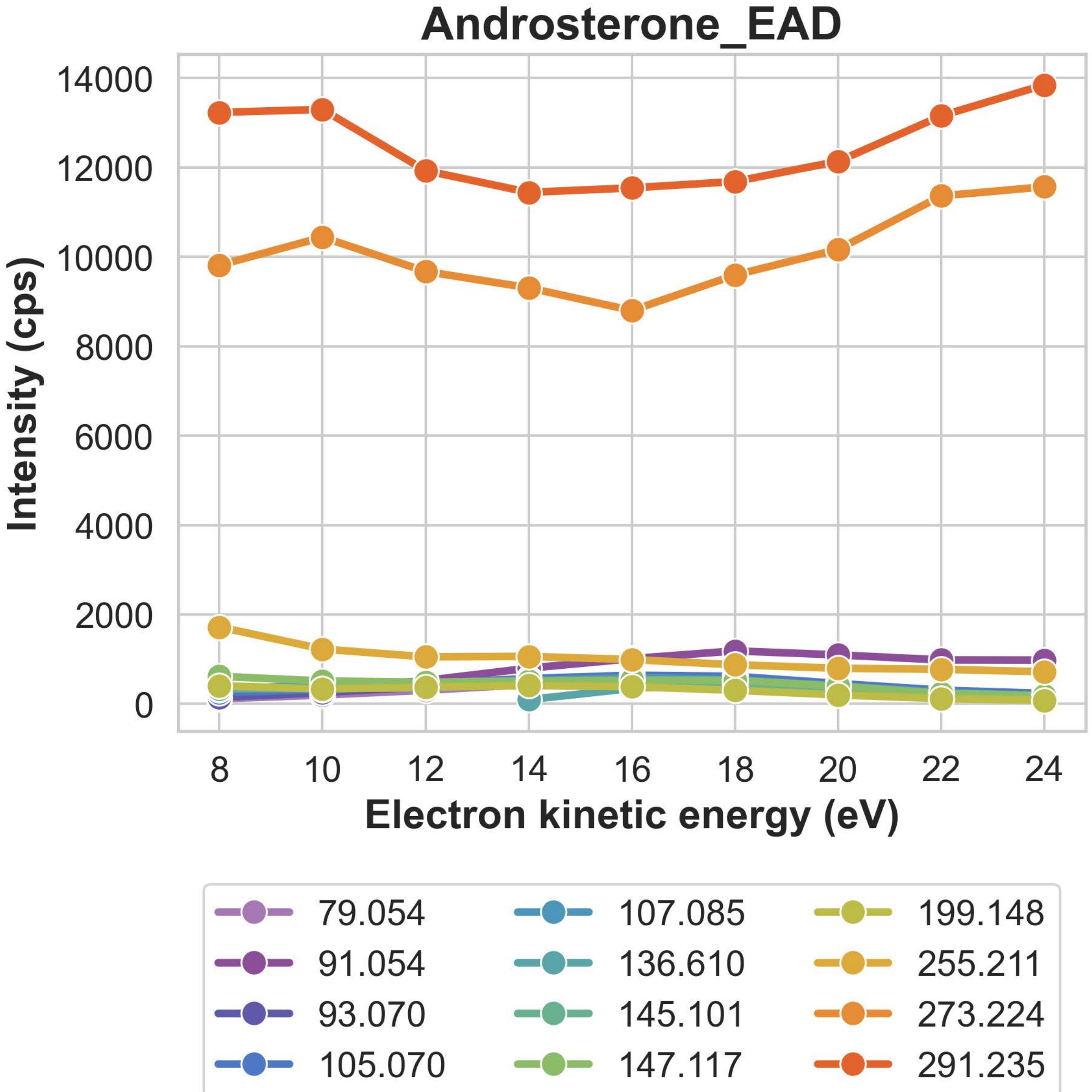
Androsterone_EAD
Intensity (cps)
14000
12000
10000
8000
6000
4000
2000
0
8
10
12
14
16
18
20
22
24
Electron kinetic energy (eV)
79.054
91.054
93.070
105.070
107.085
136.610
145.101
147.117
199.148
255.211
273.224
291.235

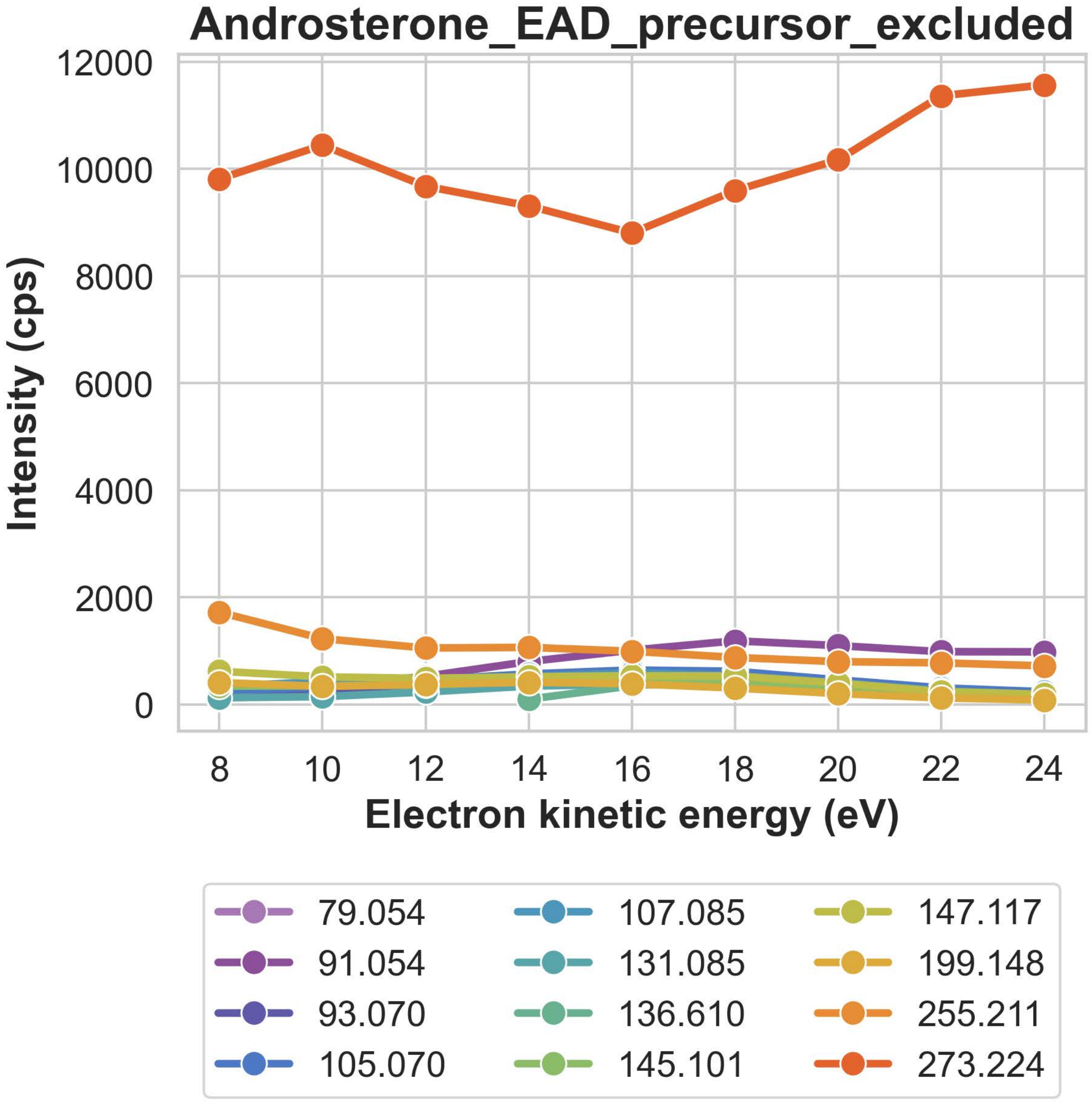
Androsterone_EAD_precursor_excluded
Intensity (cps)
12000
10000
8000
6000
4000
2000
0
8
10
12
14
16
18
20
22
24
Electron kinetic energy (eV)
79.054
91.054
93.070
105.070
107.085
131.085
136.610
145.101
147.117
199.148
255.211
273.224

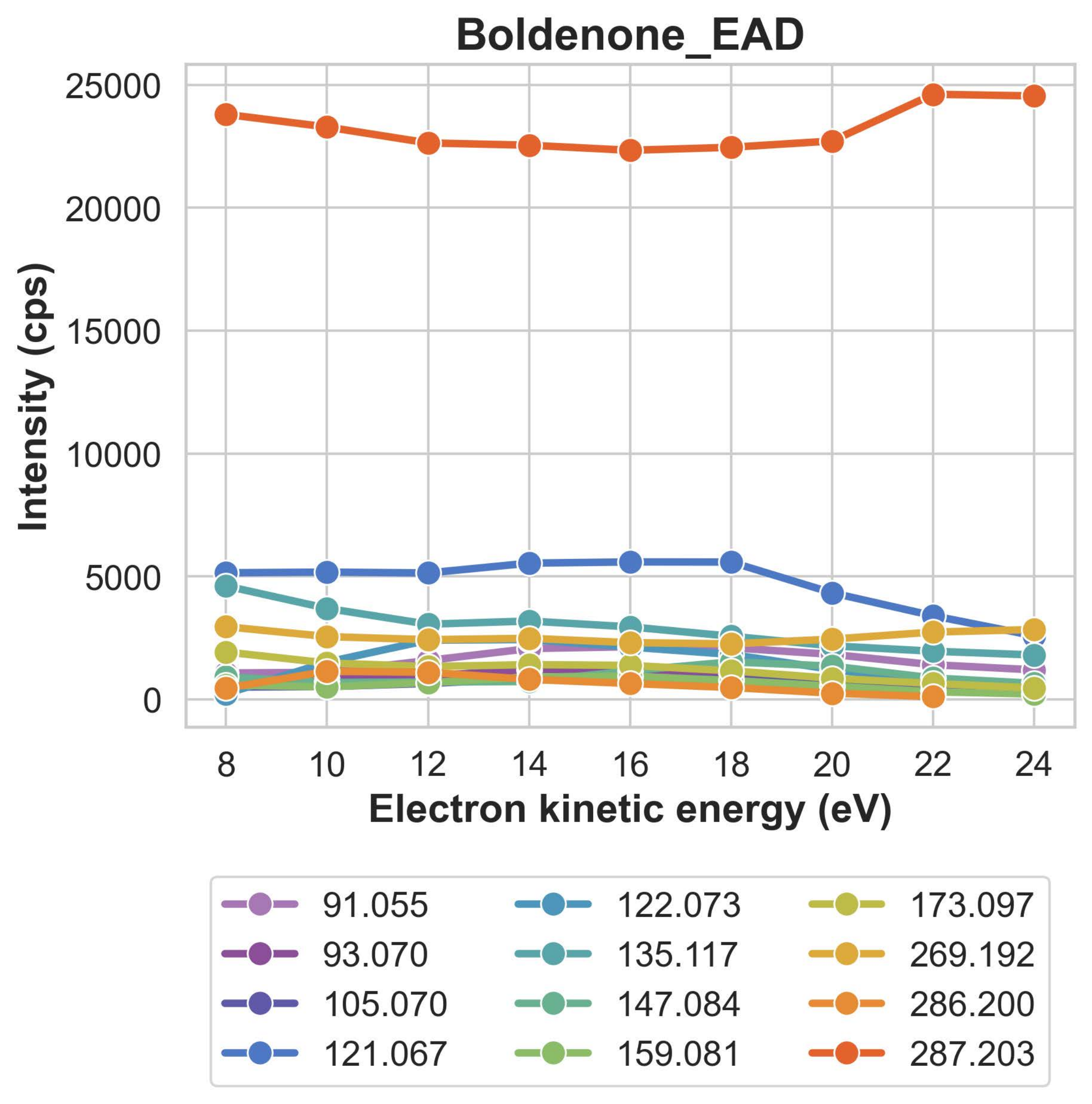
Boldenone_EAD
Intensity (cps)
25000
20000
15000
10000
5000
0
8
10
12
14
16
18
20
22
24
Electron kinetic energy (eV)
91.055
93.070
105.070
121.067
122.073
135.117
147.084
159.081
173.097
269.192
286.200
287.203

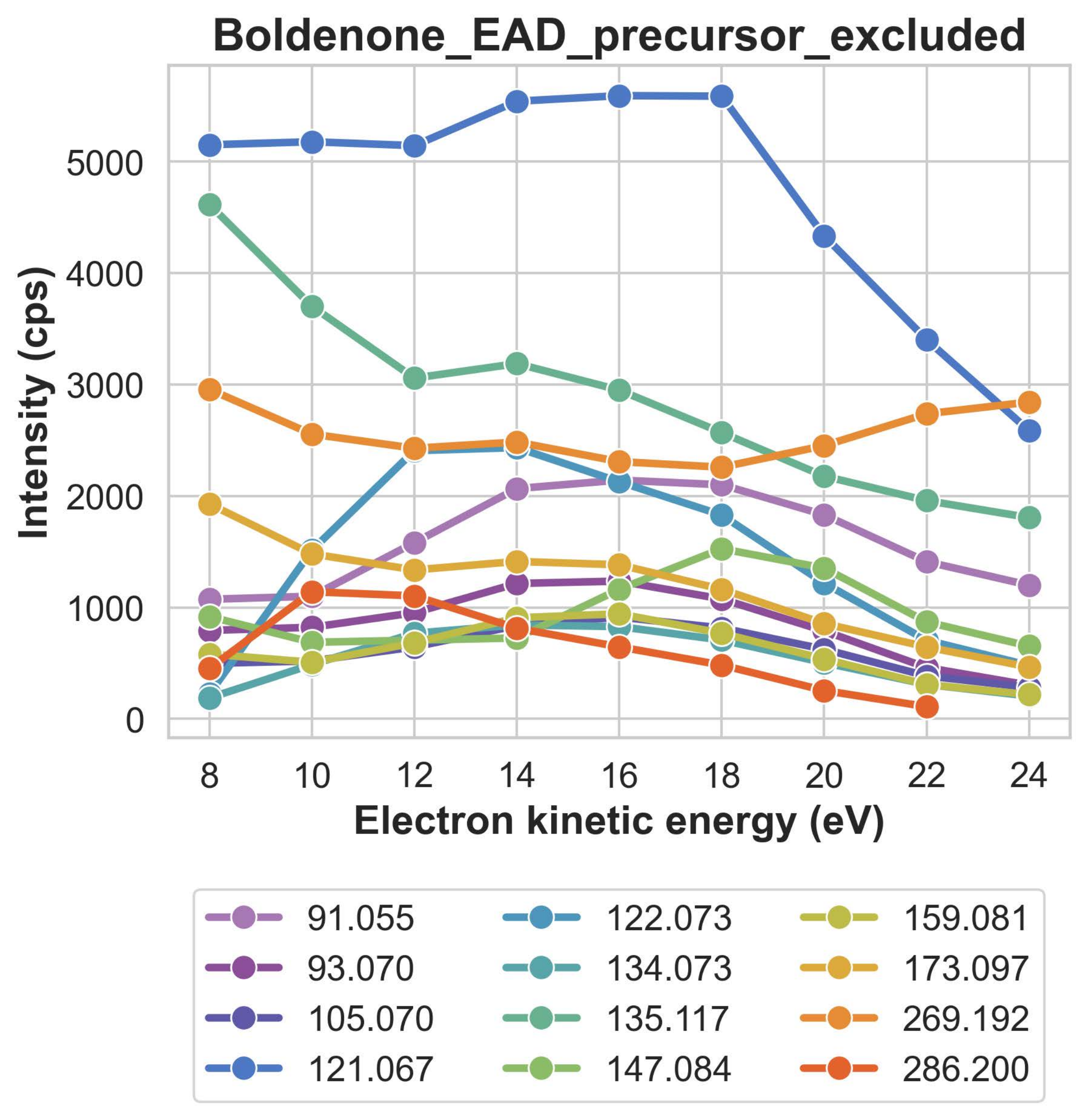
Boldenone_EAD_precursor_excluded
Intensity (cps)
5000
4000
3000
2000
1000
0
8
10
12
14
16
18
20
22
24
Electron kinetic energy (eV)
91.055
93.070
105.070
121.067
122.073
134.073
135.117
147.084
159.081
173.097
269.192
286.200

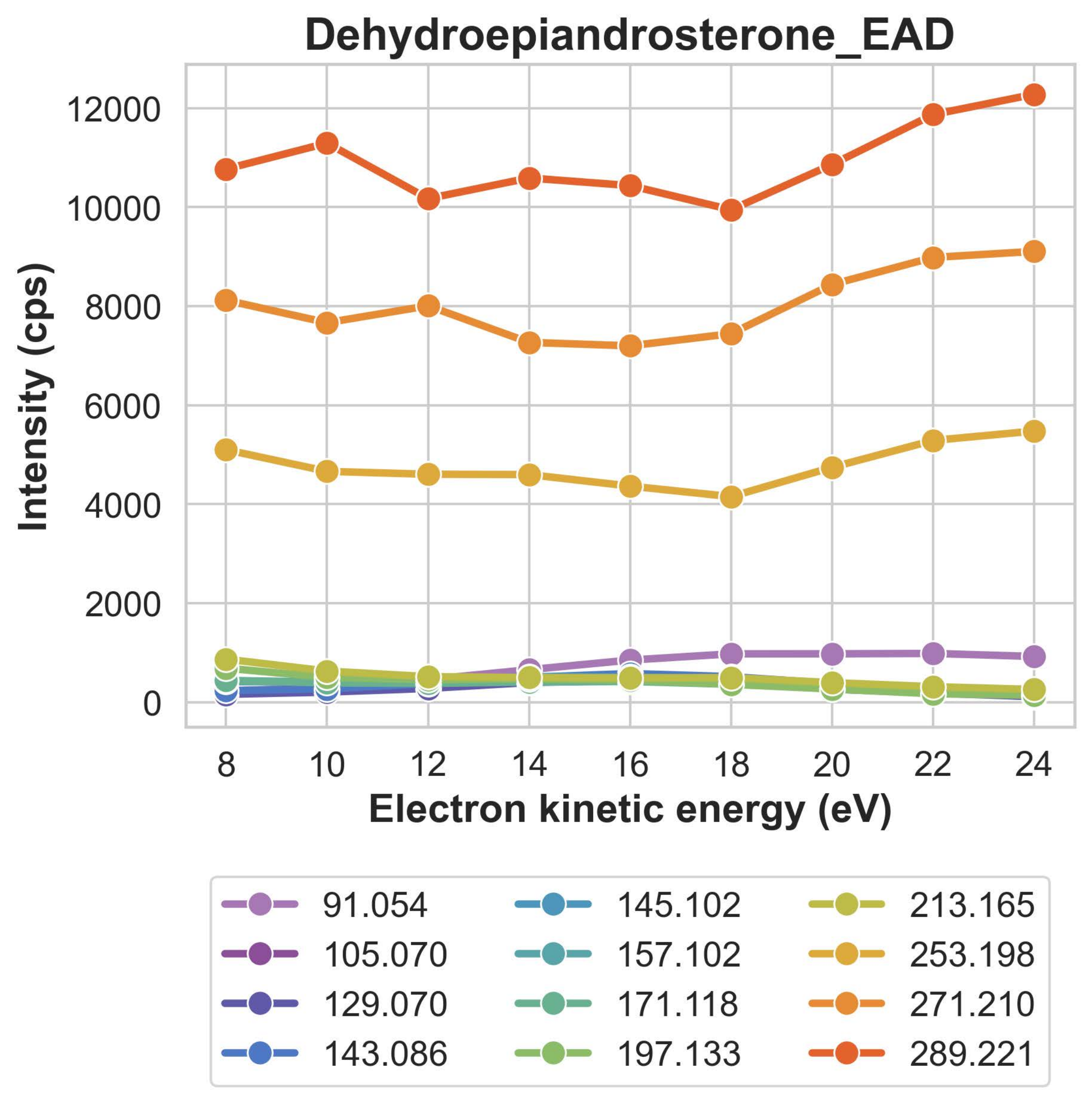

Dehydroepiandrosterone_EAD
Intensity (cps)
0
2000
4000
6000
8000
10000
12000
8
10
12
14
16
18
20
22
24
Electron kinetic energy (eV)
91.054
105.070
129.070
143.086
145.102
157.102
171.118
197.133
213.165
253.198
271.210
289.221

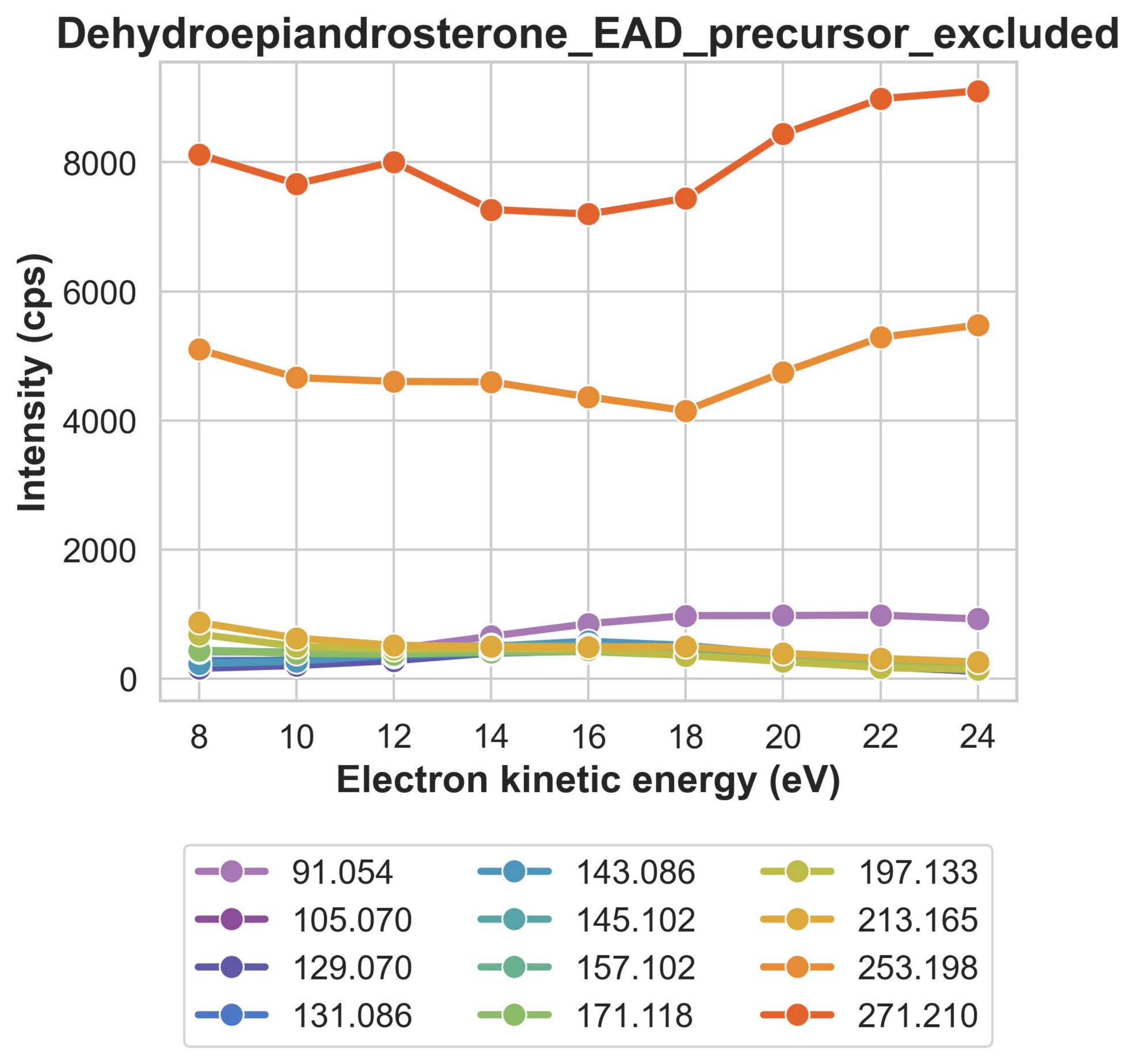
Dehydroepiandrosterone_EAD_precursor_excluded
Intensity (cps)
0
2000
4000
6000
8000
8
10
12
14
16
18
20
22
24
Electron kinetic energy (eV)
91.054
105.070
129.070
131.086
143.086
145.102
157.102
171.118
197.133
213.165
253.198
271.210

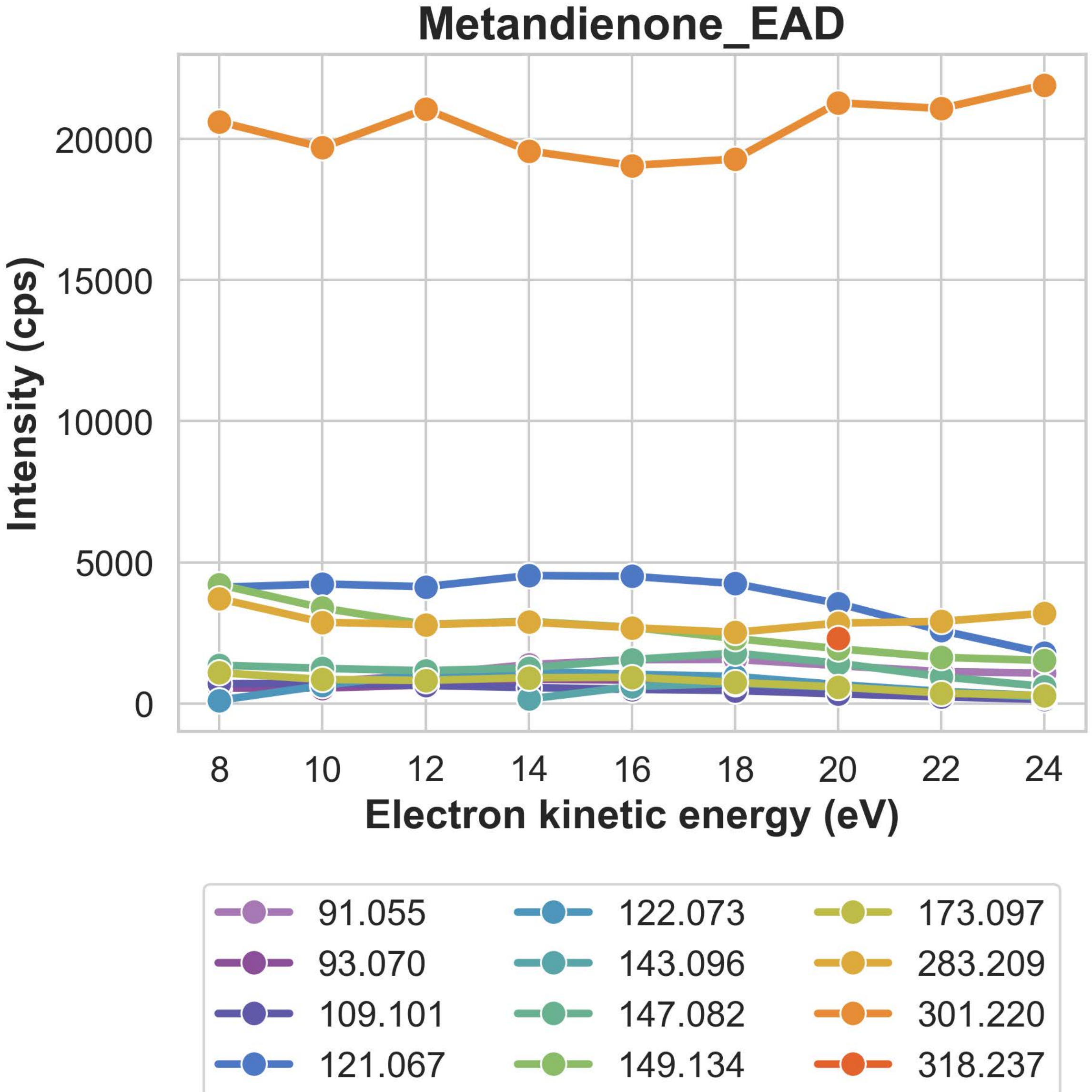

Metandienone_EAD
Intensity (cps)
20000
15000
10000
5000
0
8
10
12
14
16
18
20
22
24
Electron kinetic energy (eV)
91.055
93.070
109.101
121.067
122.073
143.096
147.082
149.134
173.097
283.209
301.220
318.237

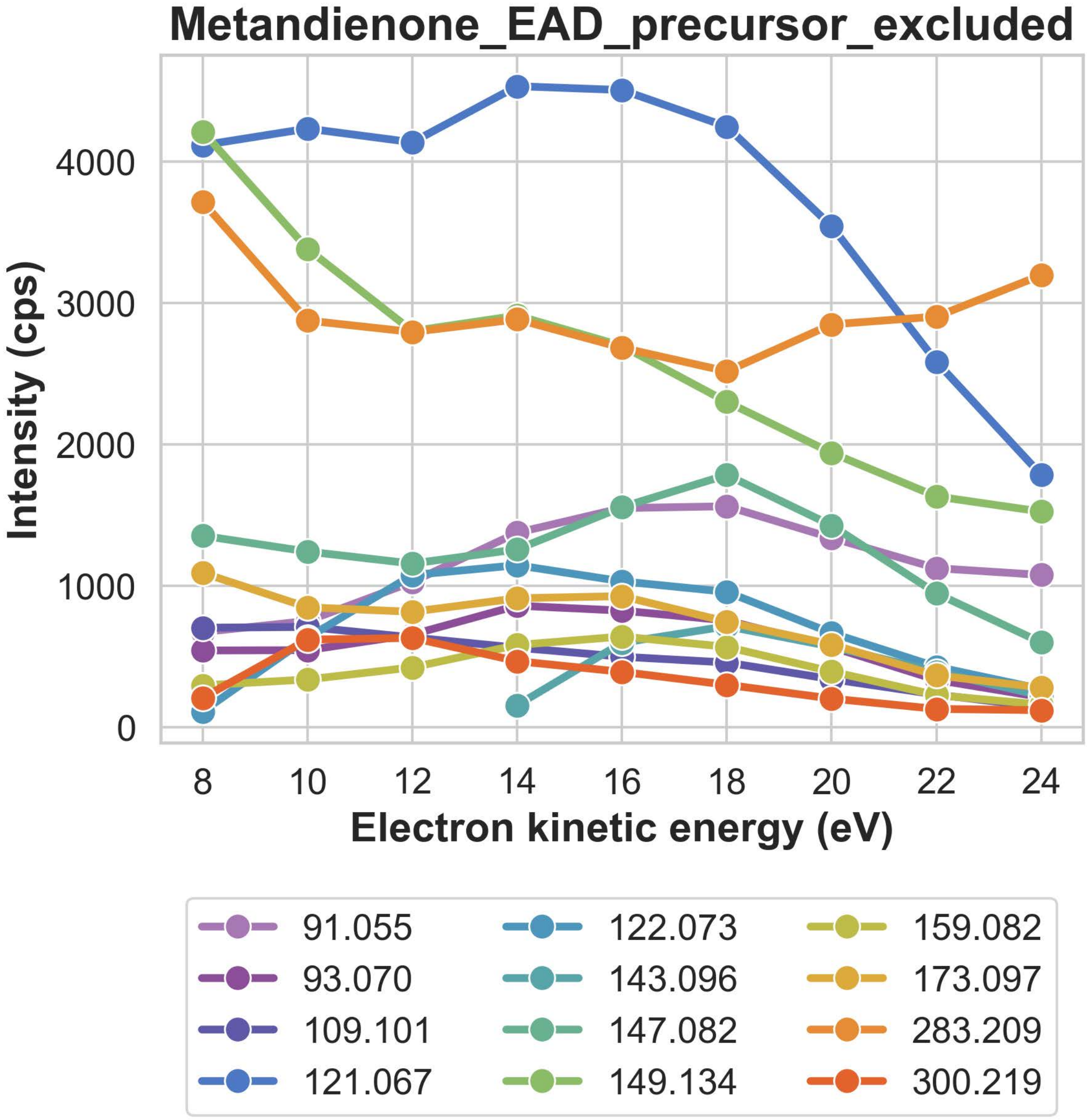
Metandienone_EAD_precursor_excluded
Intensity (cps)
0
1000
2000
3000
4000
8
10
12
14
16
18
20
22
24
Electron kinetic energy (eV)
91.055
93.070
109.101
121.067
122.073
143.096
147.082
149.134
159.082
173.097
283.209
300.219

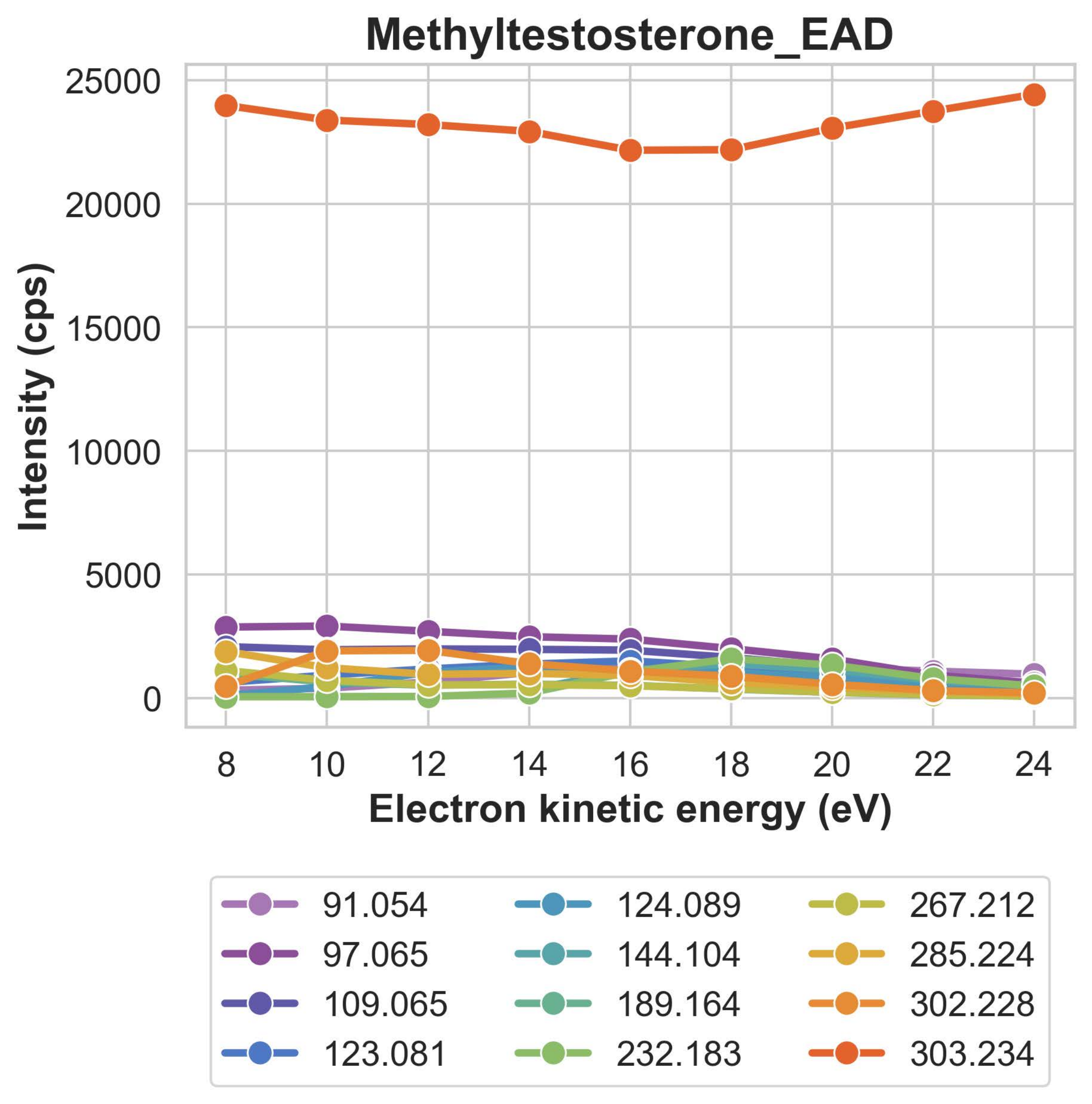
Methyltestosterone_EAD
Intensity (cps)
25000
20000
15000
10000
5000
0
8
10
12
14
16
18
20
22
24
Electron kinetic energy (eV)
91.054
97.065
109.065
123.081
124.089
144.104
189.164
232.183
267.212
285.224
302.228
303.234

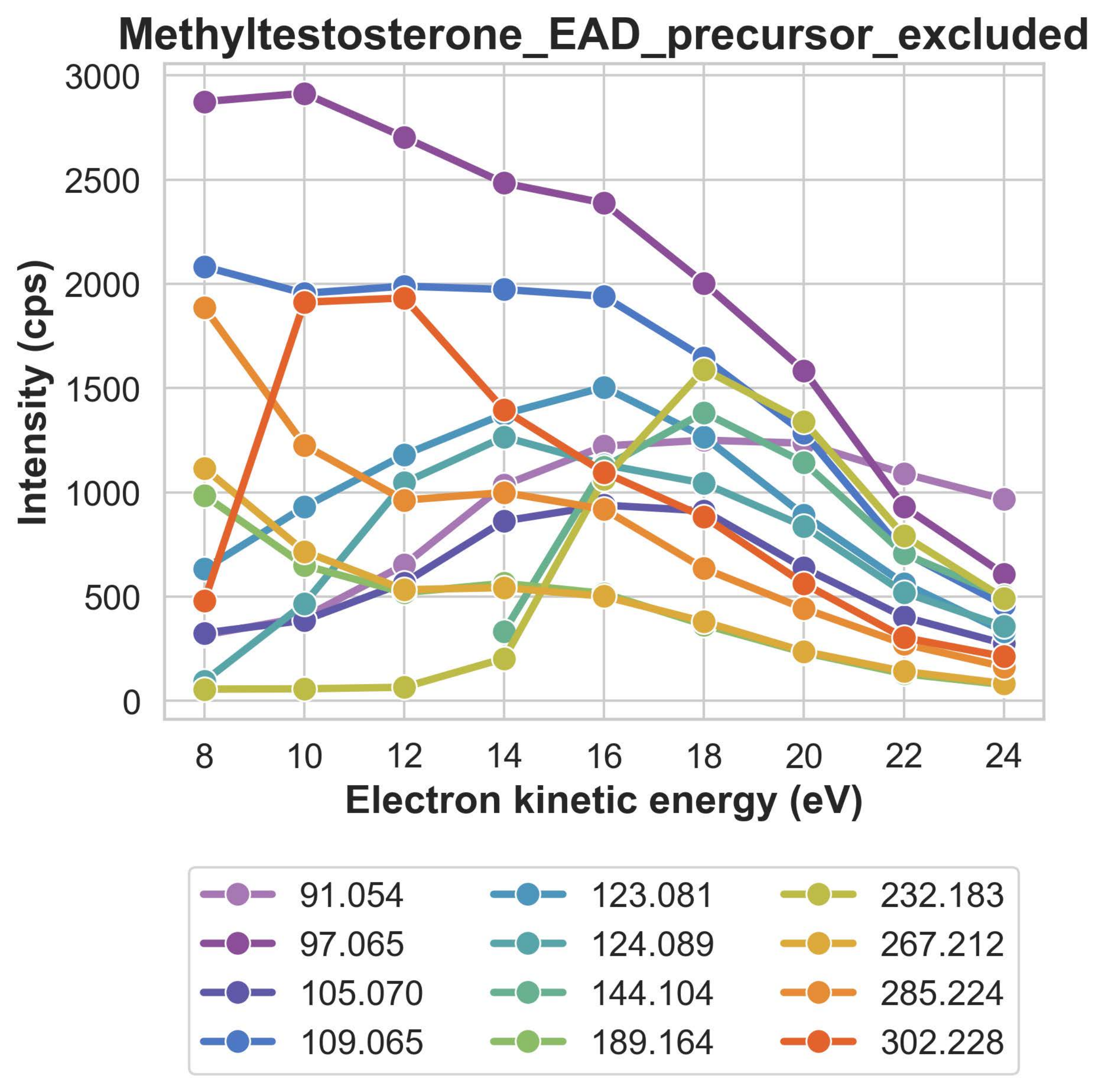

Methyltestosterone_EAD_precursor_excluded
Intensity (cps)
3000
2500
2000
1500
1000
500
0
8
10
12
14
16
18
20
22
24
Electron kinetic energy (eV)
91.054
97.065
105.070
109.065
123.081
124.089
144.104
189.164
232.183
267.212
285.224
302.228

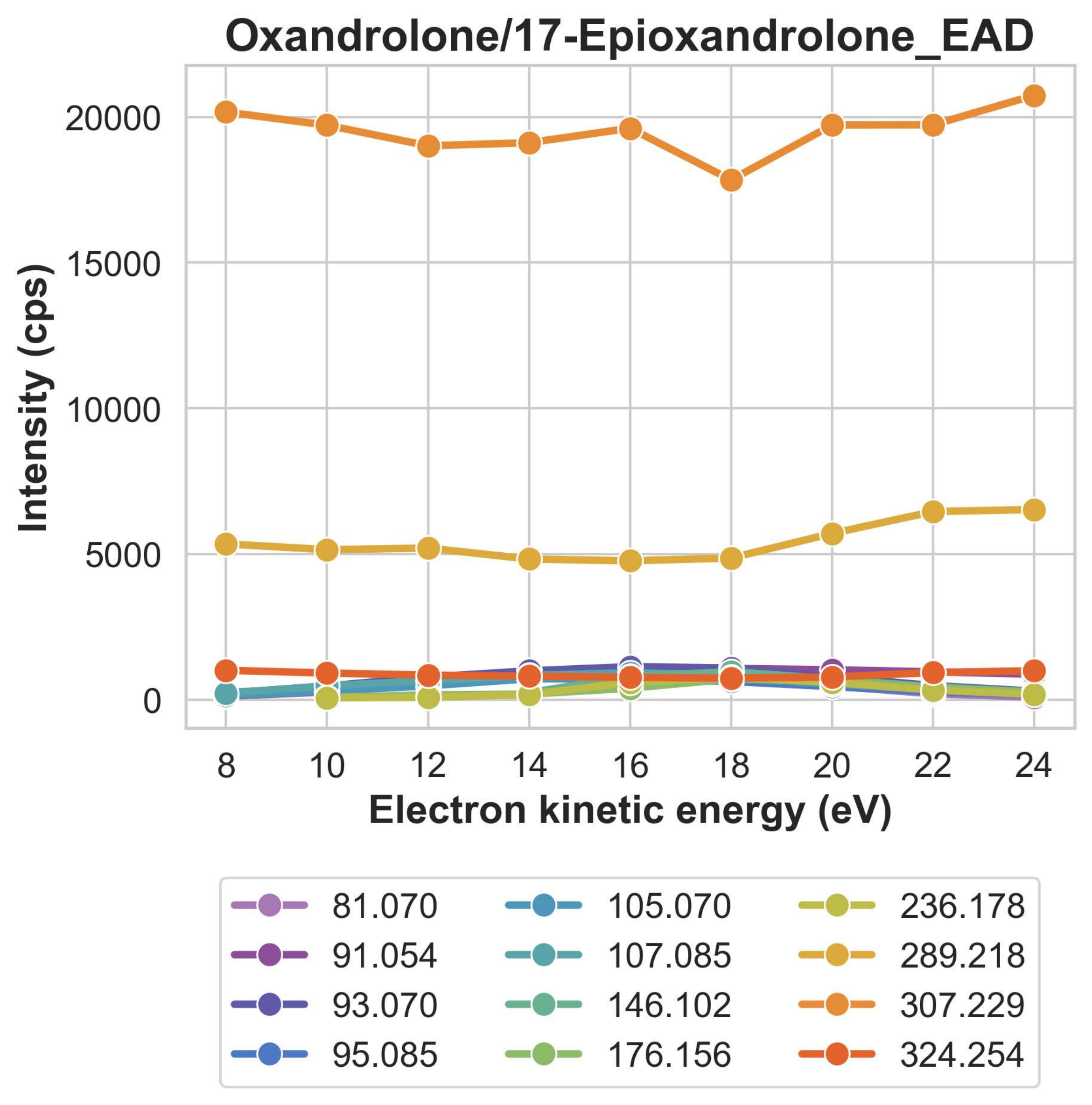

Oxandrolone/17-Epioxandrolone_EAD
Intensity (cps)
20000
15000
10000
5000
0
8
10
12
14
16
18
20
22
24
Electron kinetic energy (eV)
81.070
91.054
93.070
95.085
105.070
107.085
146.102
176.156
236.178
289.218
307.229
324.254

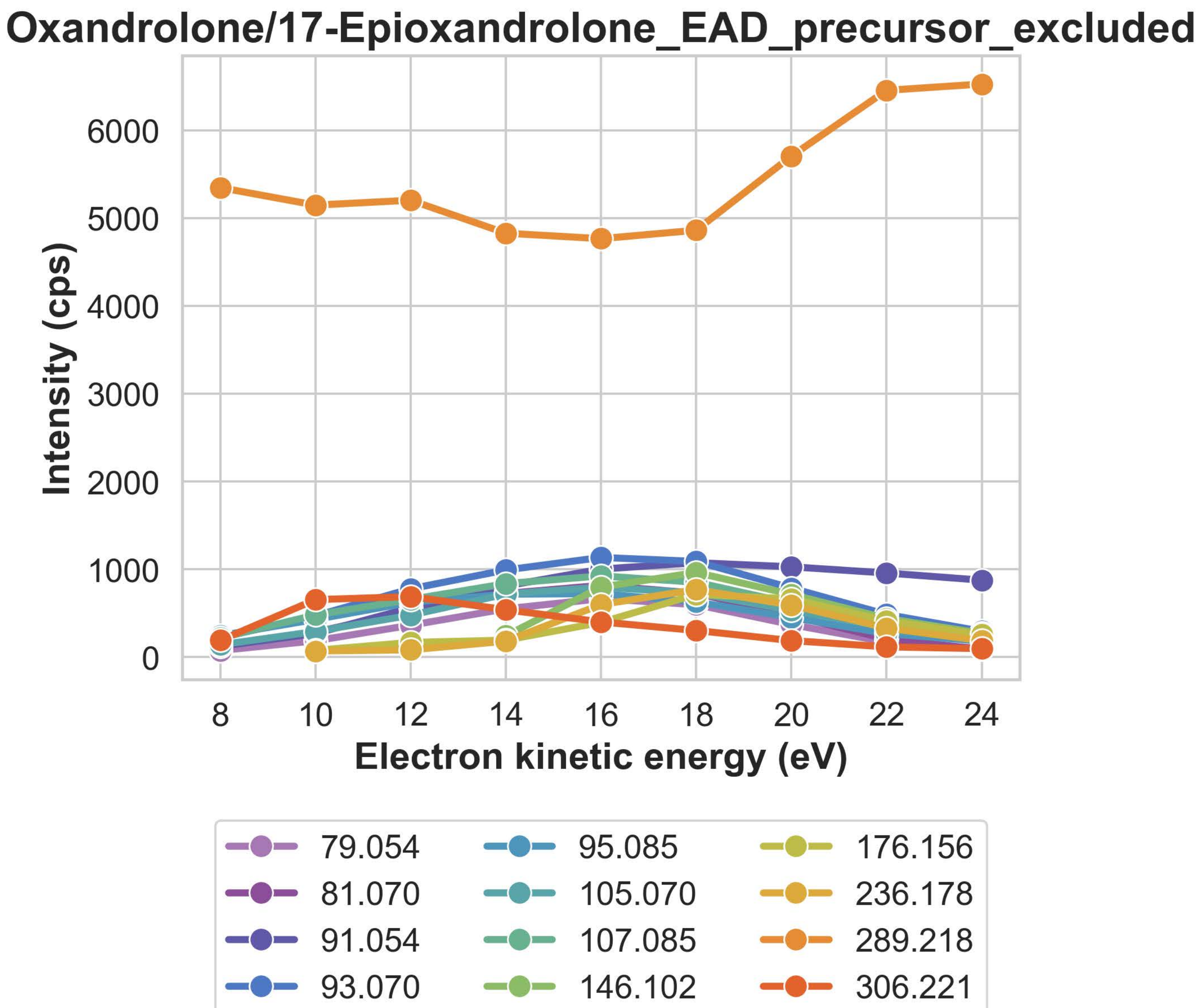
Oxandrolone/17-Epioxandrolone_EAD_precursor_excluded
Intensity (cps)
0
1000
2000
3000
4000
5000
6000
Electron kinetic energy (eV)
8
10
12
14
16
18
20
22
24
79.054
81.070
91.054
93.070
95.085
105.070
107.085
146.102
176.156
236.178
289.218
306.221

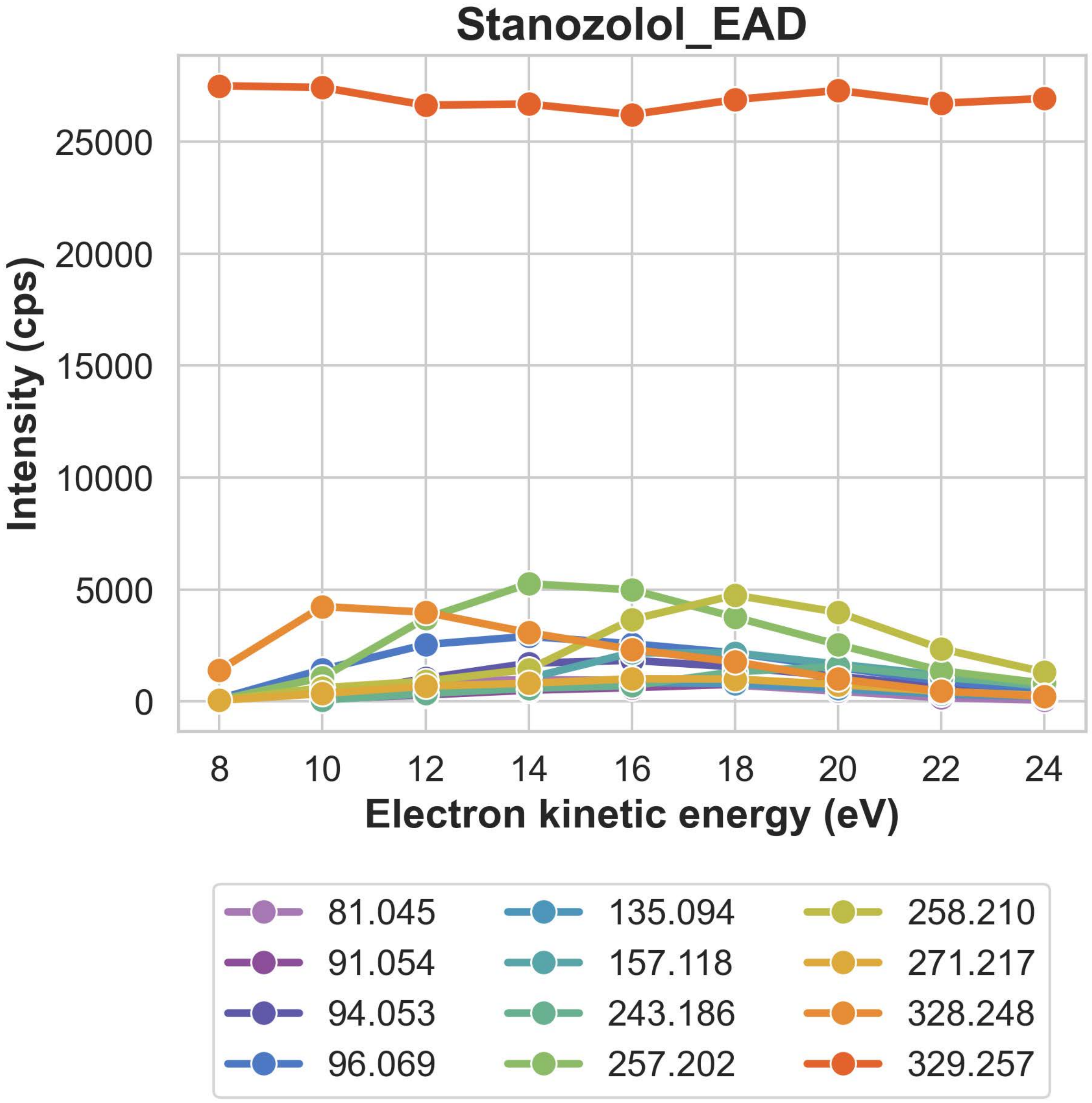
Stanozolol_EAD
Intensity (cps)
25000
20000
15000
10000
5000
0
8
10
12
14
16
18
20
22
24
Electron kinetic energy (eV)
81.045
91.054
94.053
96.069
135.094
157.118
243.186
257.202
258.210
271.217
328.248
329.257

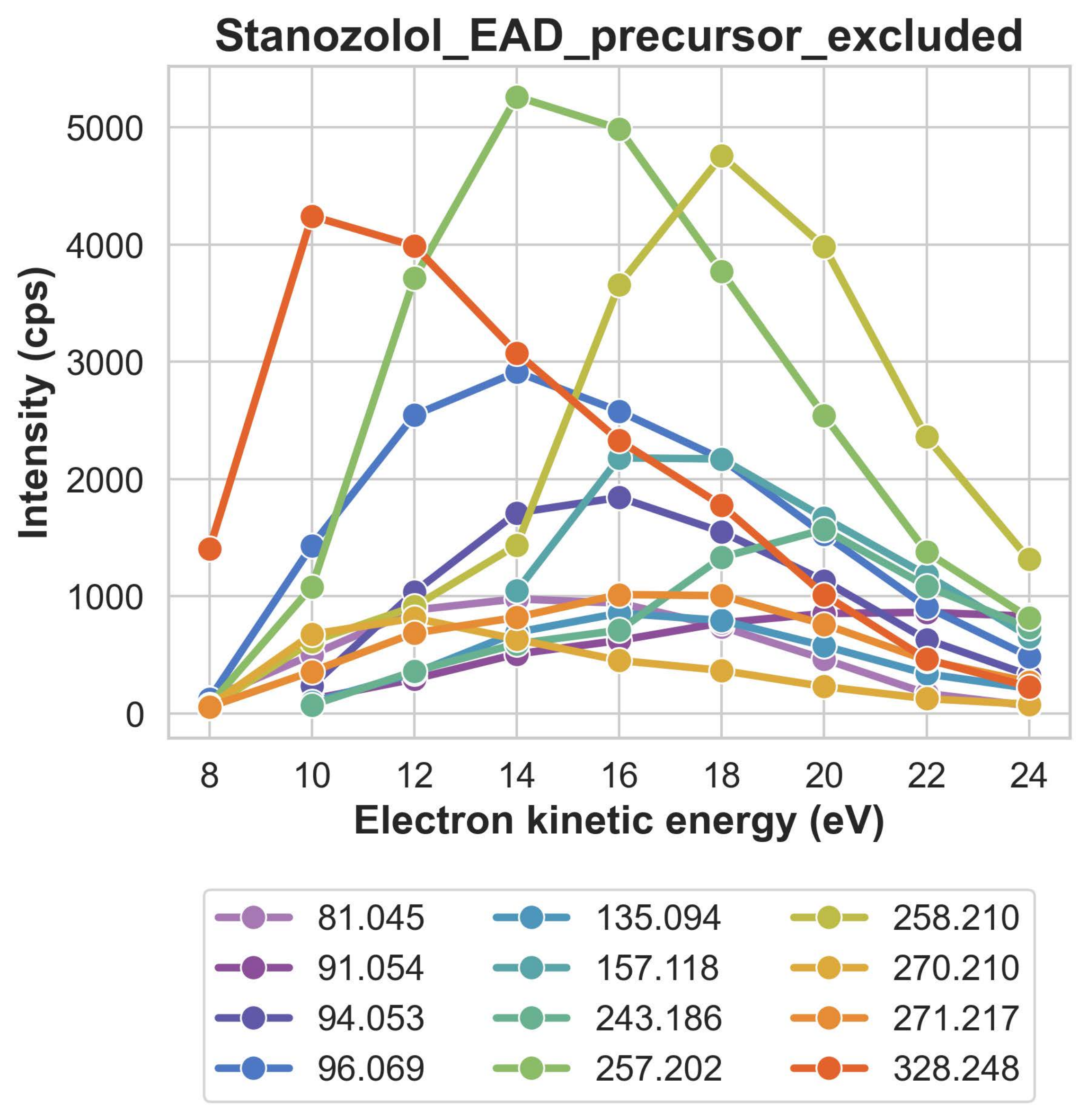

Stanozolol_EAD_precursor_excluded
Intensity (cps)
0
1000
2000
3000
4000
5000
8
10
12
14
16
18
20
22
24
Electron kinetic energy (eV)
81.045
91.054
94.053
96.069
135.094
157.118
243.186
257.202
258.210
270.210
271.217
328.248

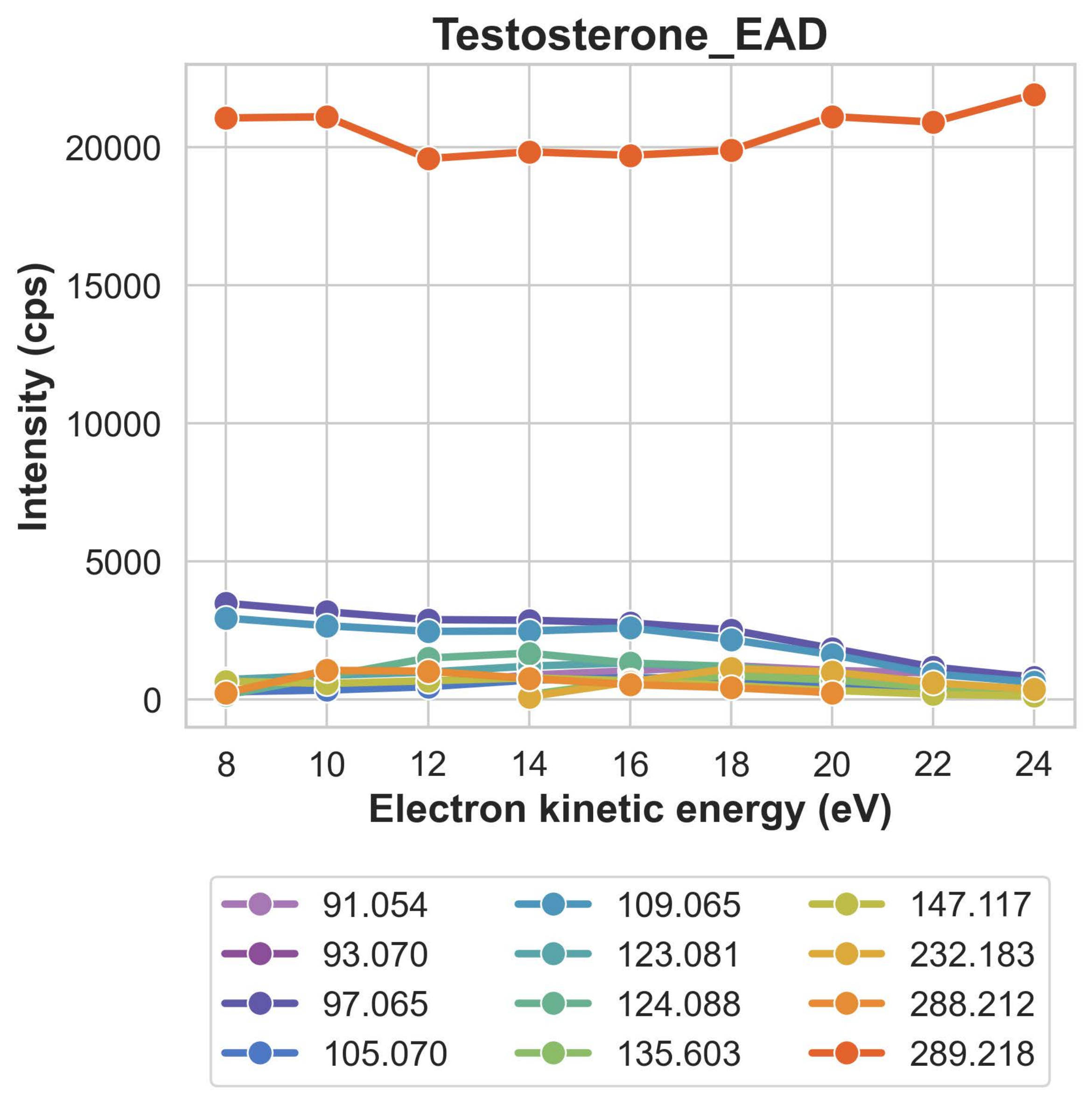

Testosterone_EAD
Intensity (cps)
20000
15000
10000
5000
0
8
10
12
14
16
18
20
22
24
Electron kinetic energy (eV)
91.054
93.070
97.065
105.070
109.065
123.081
124.088
135.603
147.117
232.183
288.212
289.218

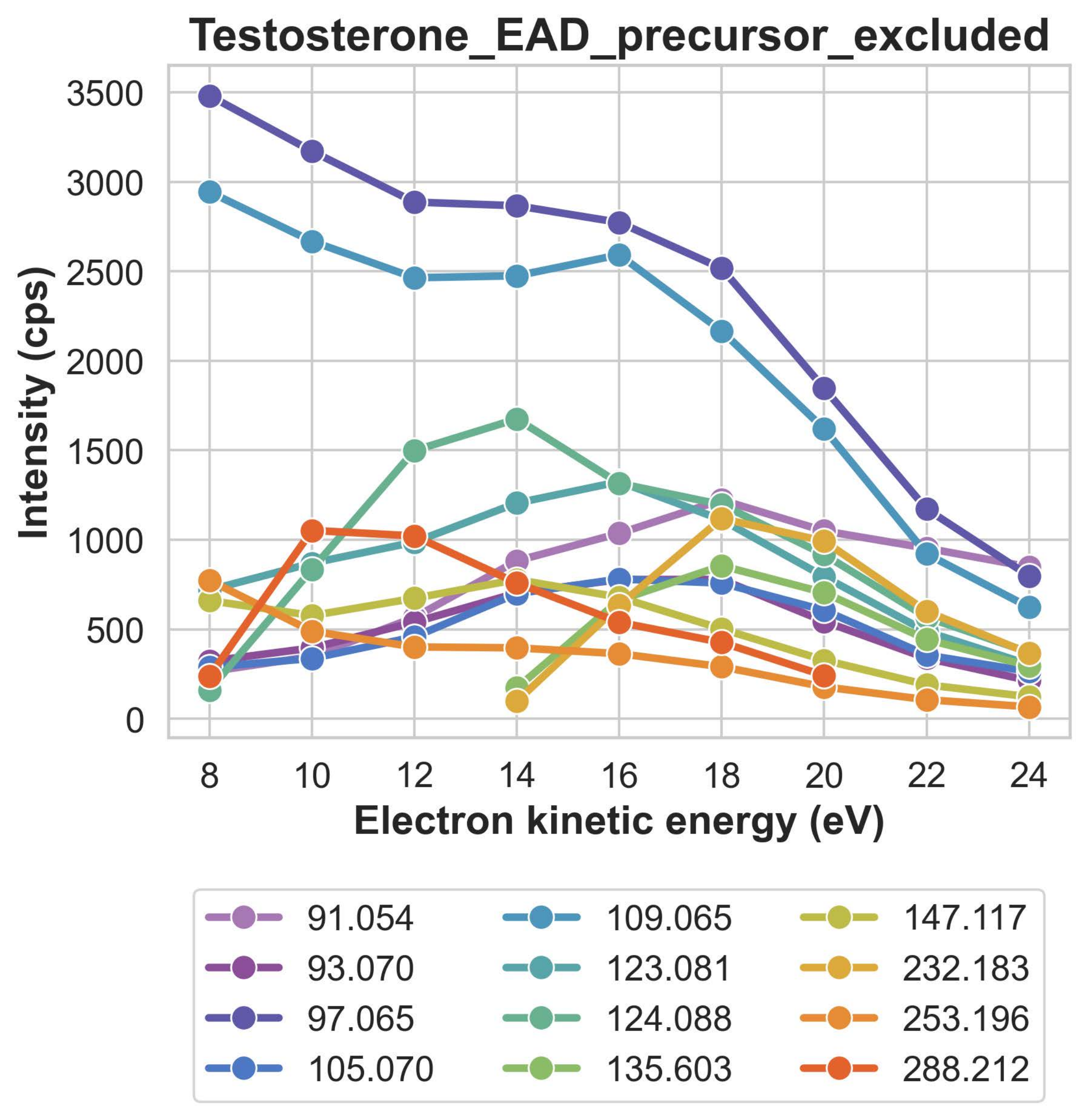

Testosterone_EAD_precursor_excluded
Intensity (cps)
3500
3000
2500
2000
1500
1000
500
0
8
10
12
14
16
18
20
22
24
Electron kinetic energy (eV)
91.054
93.070
97.065
105.070
109.065
123.081
124.088
135.603
147.117
232.183
253.196
288.212

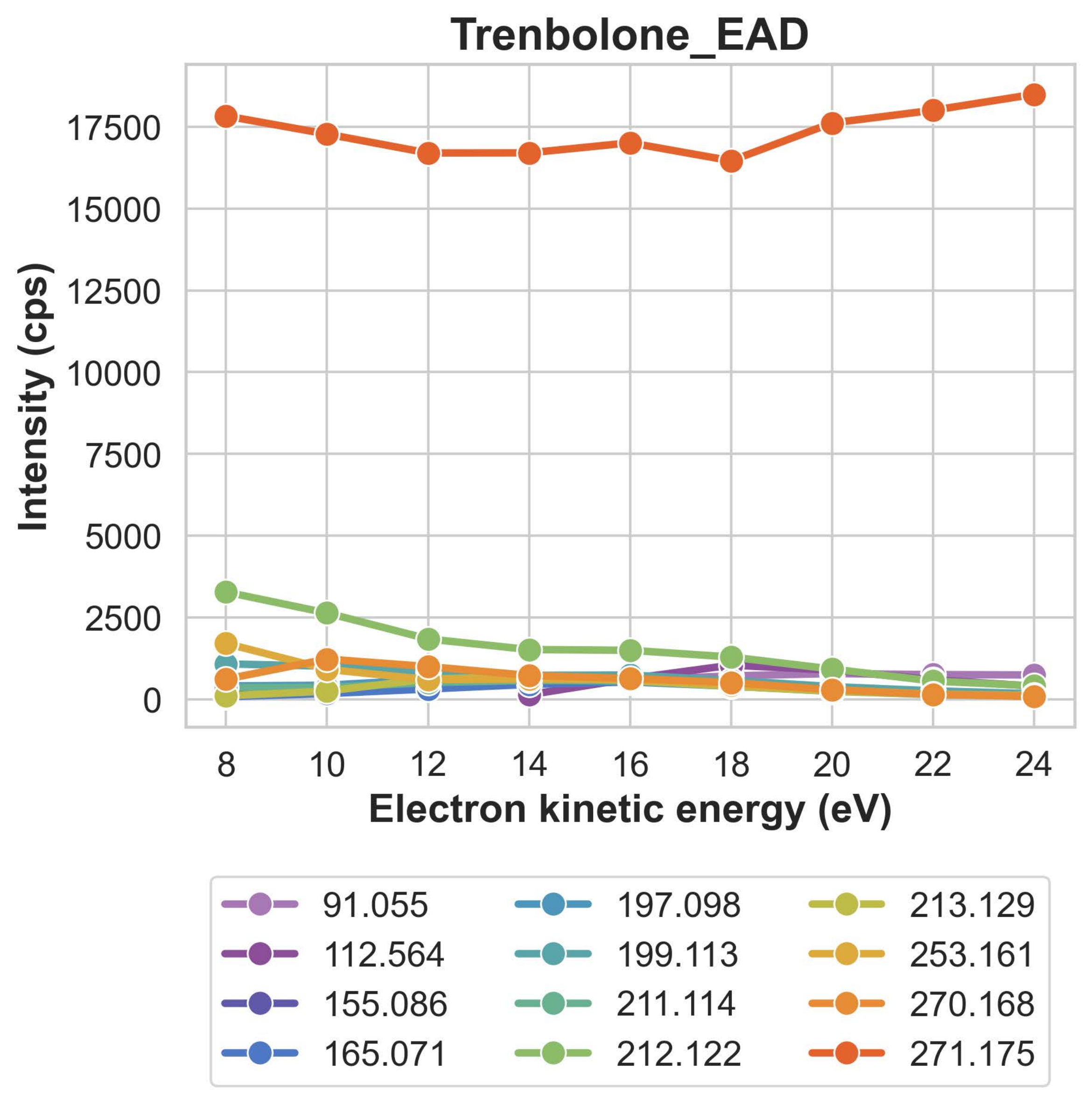

Trenbolone_EAD
Intensity (cps)
17500
15000
12500
10000
7500
5000
2500
0
8
10
12
14
16
18
20
22
24
Electron kinetic energy (eV)
91.055
112.564
155.086
165.071
197.098
199.113
211.114
212.122
213.129
253.161
270.168
271.175

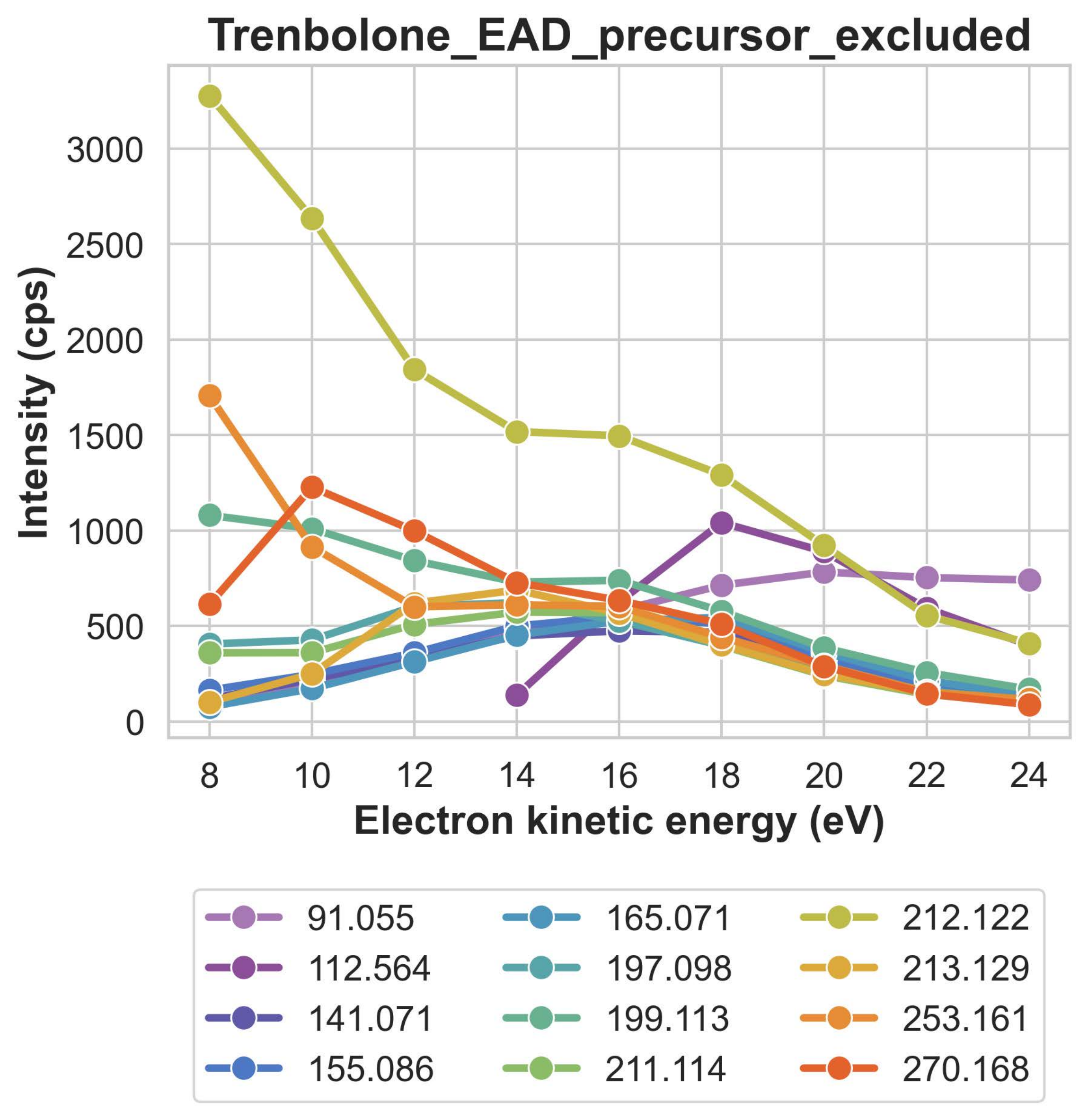
Trenbolone_EAD_precursor_excluded
Intensity (cps)
3000
2500
2000
1500
1000
500
0
8
10
12
14
16
18
20
22
24
Electron kinetic energy (eV)
91.055
112.564
141.071
155.086
165.071
197.098
199.113
211.114
212.122
213.129
253.161
270.168

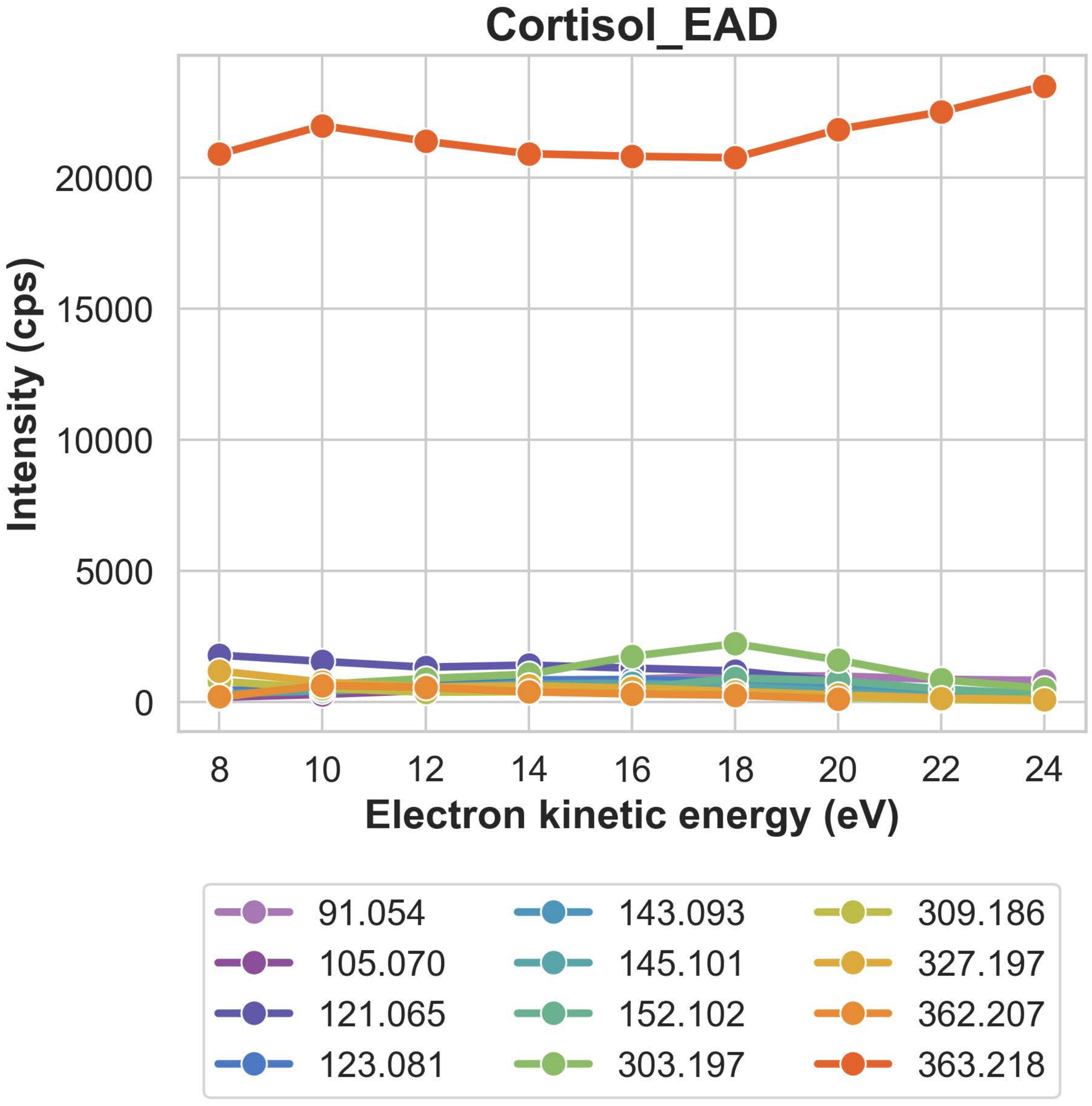
Cortisol_EAD
Intensity (cps)
20000
15000
10000
5000
0
8
10
12
14
16
18
20
22
24
Electron kinetic energy (eV)
91.054
105.070
121.065
123.081
143.093
145.101
152.102
303.197
309.186
327.197
362.207
363.218

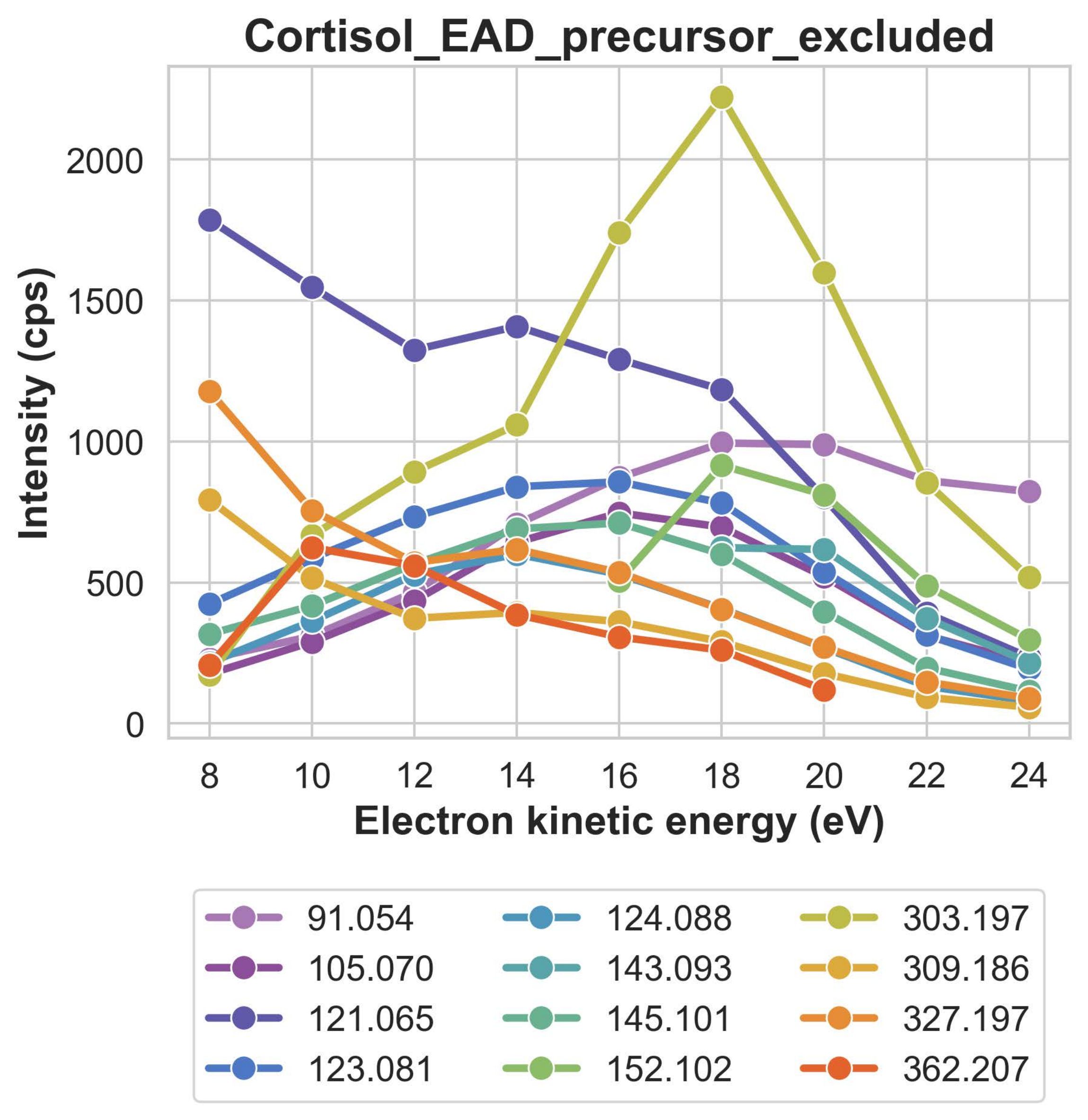
Cortisol_EAD_precursor_excluded
Intensity (cps)
0
500
1000
1500
2000
8
10
12
14
16
18
20
22
24
Electron kinetic energy (eV)
91.054
105.070
121.065
123.081
124.088
143.093
145.101
152.102
303.197
309.186
327.197
362.207

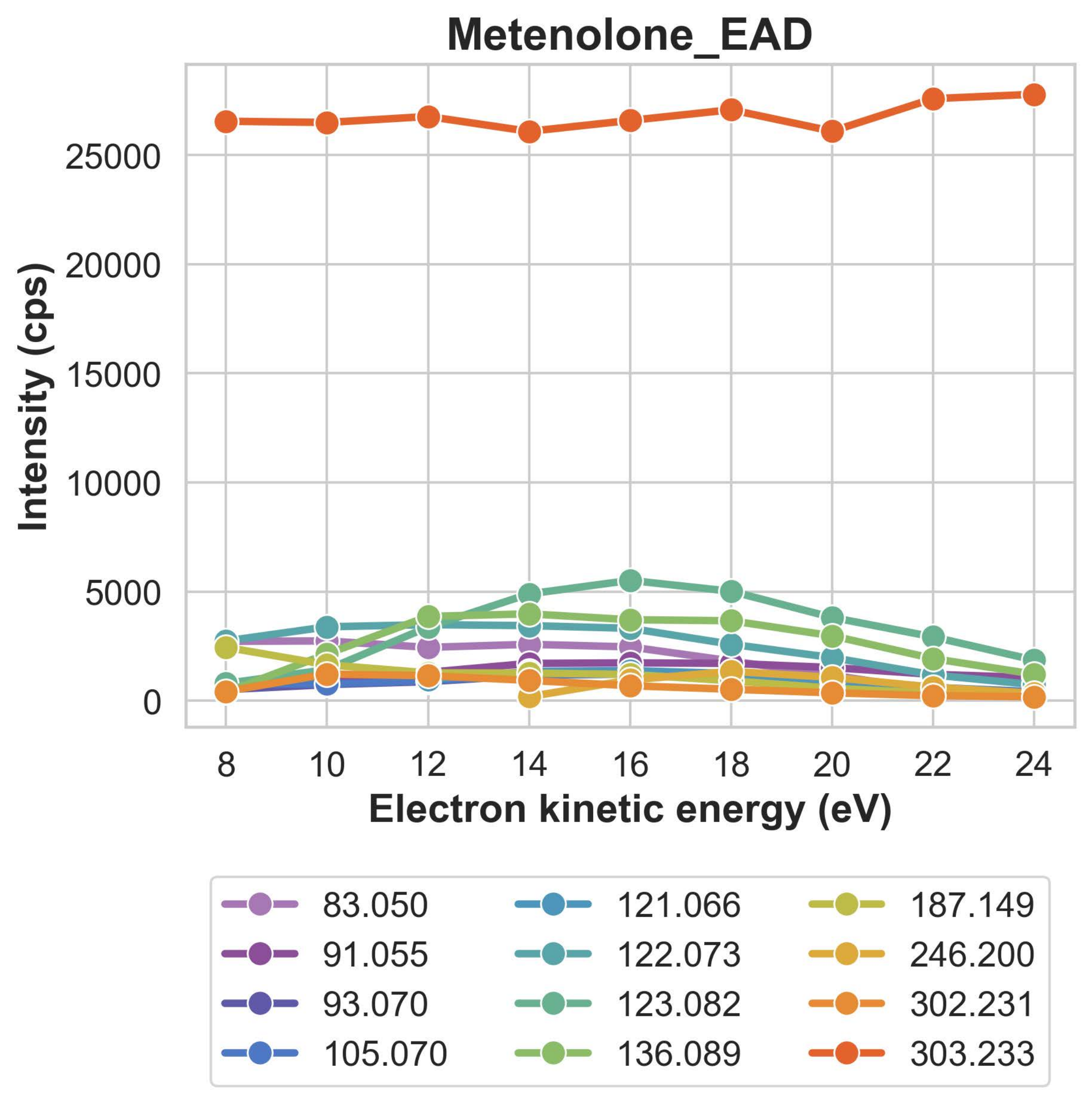
Metenolone_EAD
Intensity (cps)
25000
20000
15000
10000
5000
0
8
10
12
14
16
18
20
22
24
Electron kinetic energy (eV)
83.050
91.055
93.070
105.070
121.066
122.073
123.082
136.089
187.149
246.200
302.231
303.233

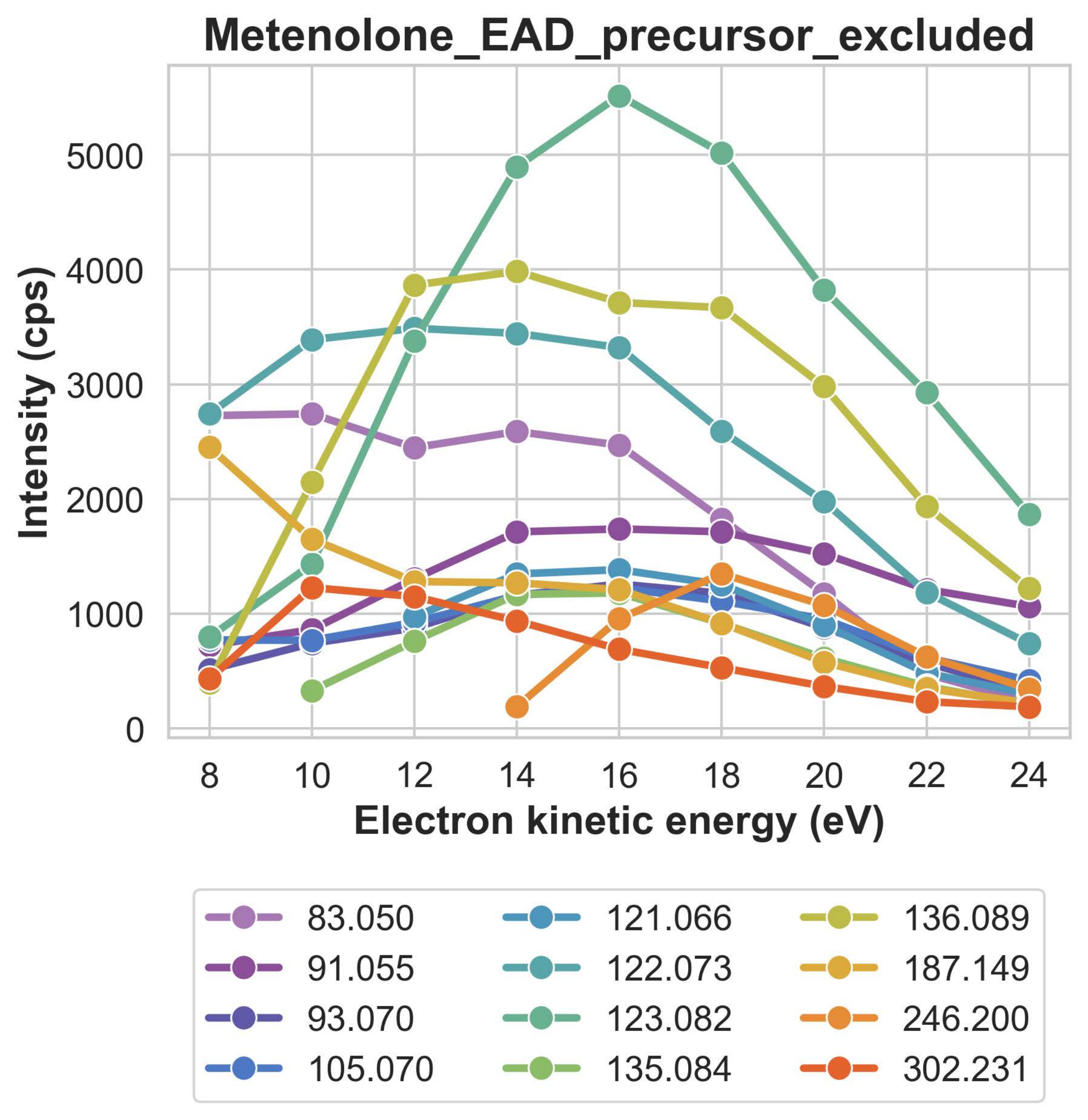
Metenolone_EAD_precursor_excluded
Intensity (cps)
0
1000
2000
3000
4000
5000
8
10
12
14
16
18
20
22
24
Electron kinetic energy (eV)
83.050
91.055
93.070
105.070
121.066
122.073
123.082
135.084
136.089
187.149
246.200
302.231

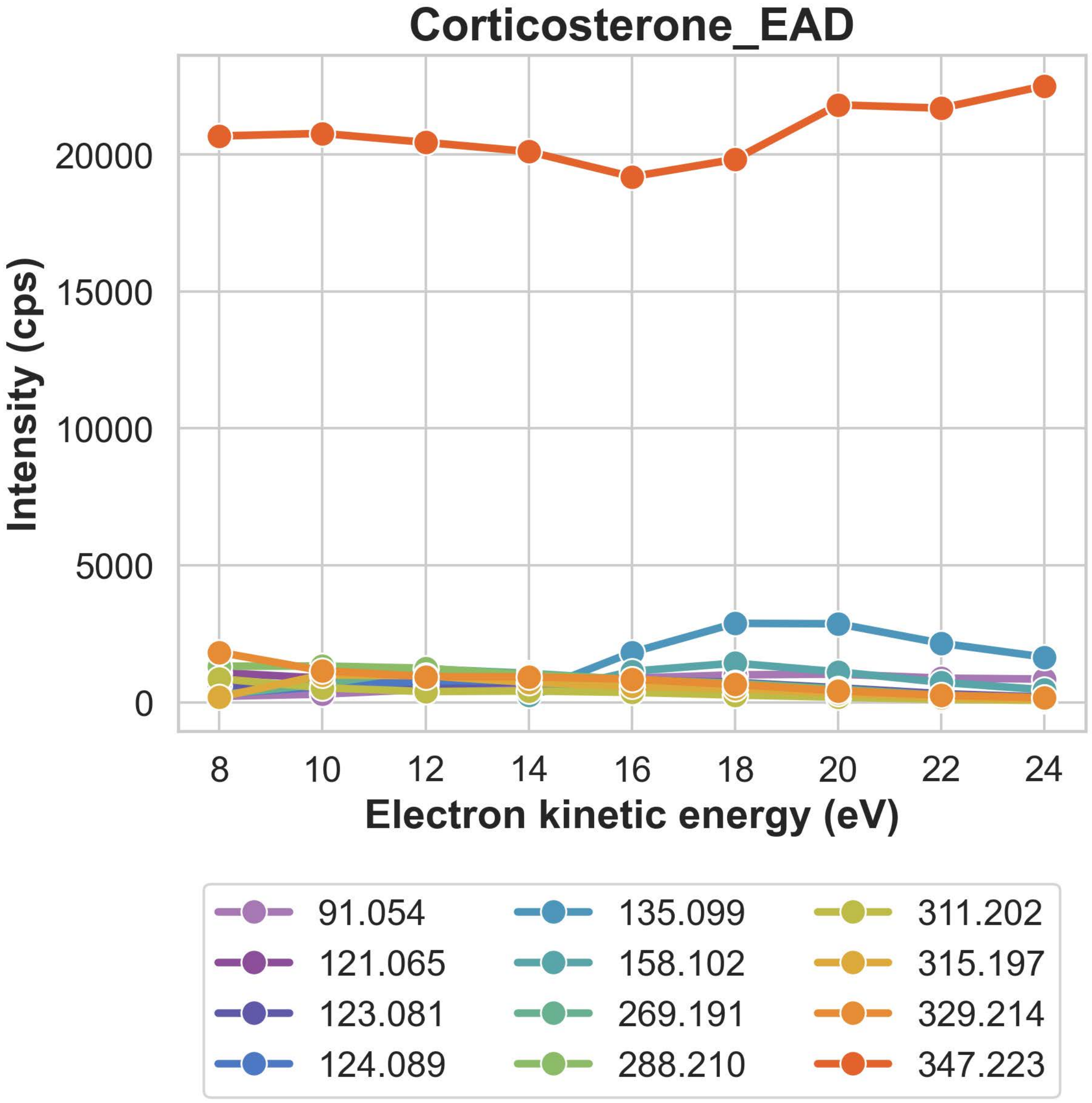
Corticosterone_EAD
Intensity (cps)
20000
15000
10000
5000
0
8
10
12
14
16
18
20
22
24
Electron kinetic energy (eV)
91.054
121.065
123.081
124.089
135.099
158.102
269.191
288.210
311.202
315.197
329.214
347.223

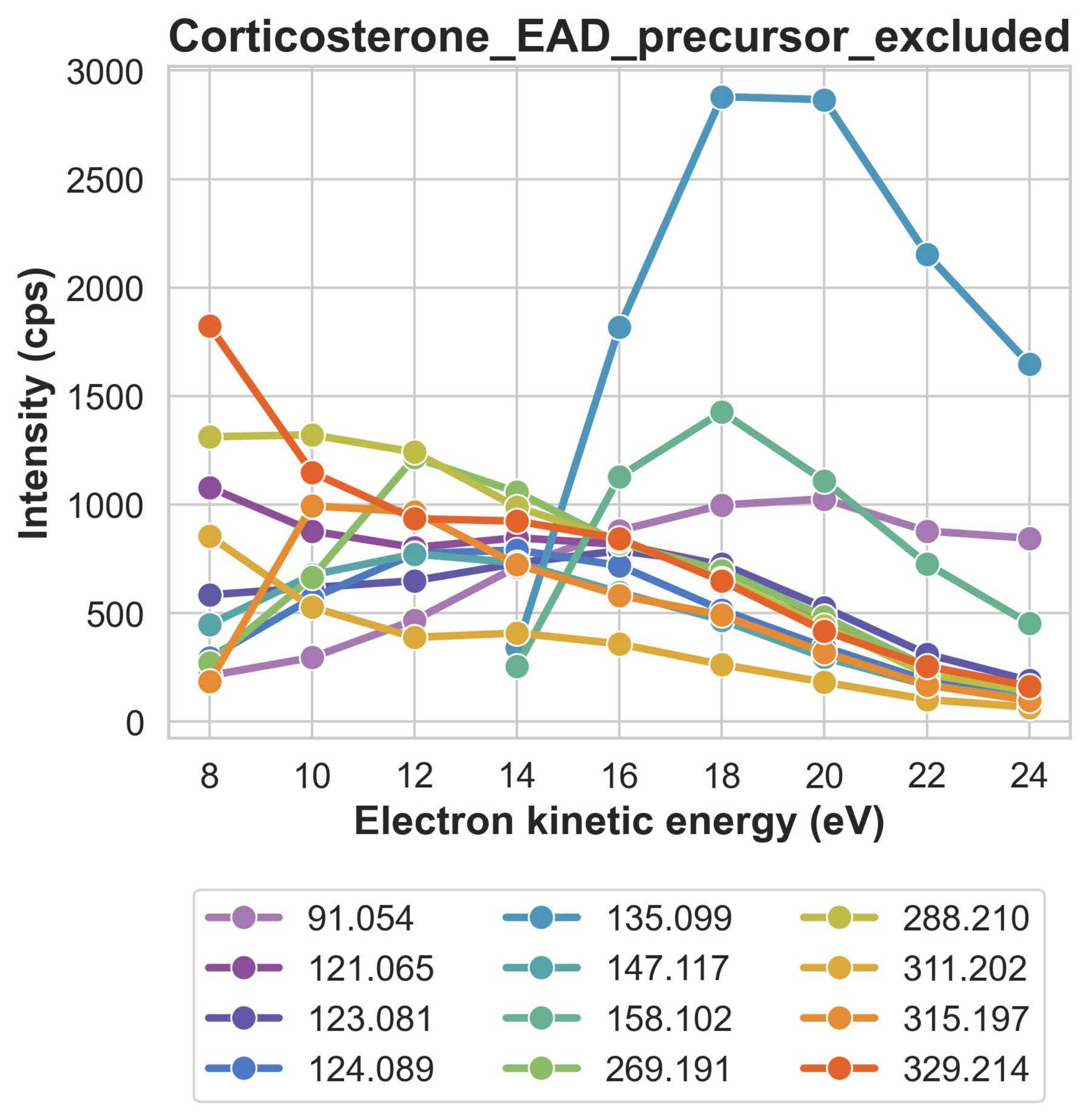
Corticosterone_EAD_precursor_excluded
Intensity (cps)
3000
2500
2000
1500
1000
500
0
8
10
12
14
16
18
20
22
24
Electron kinetic energy (eV)
91.054
121.065
123.081
124.089
135.099
147.117
158.102
269.191
288.210
311.202
315.197
329.214

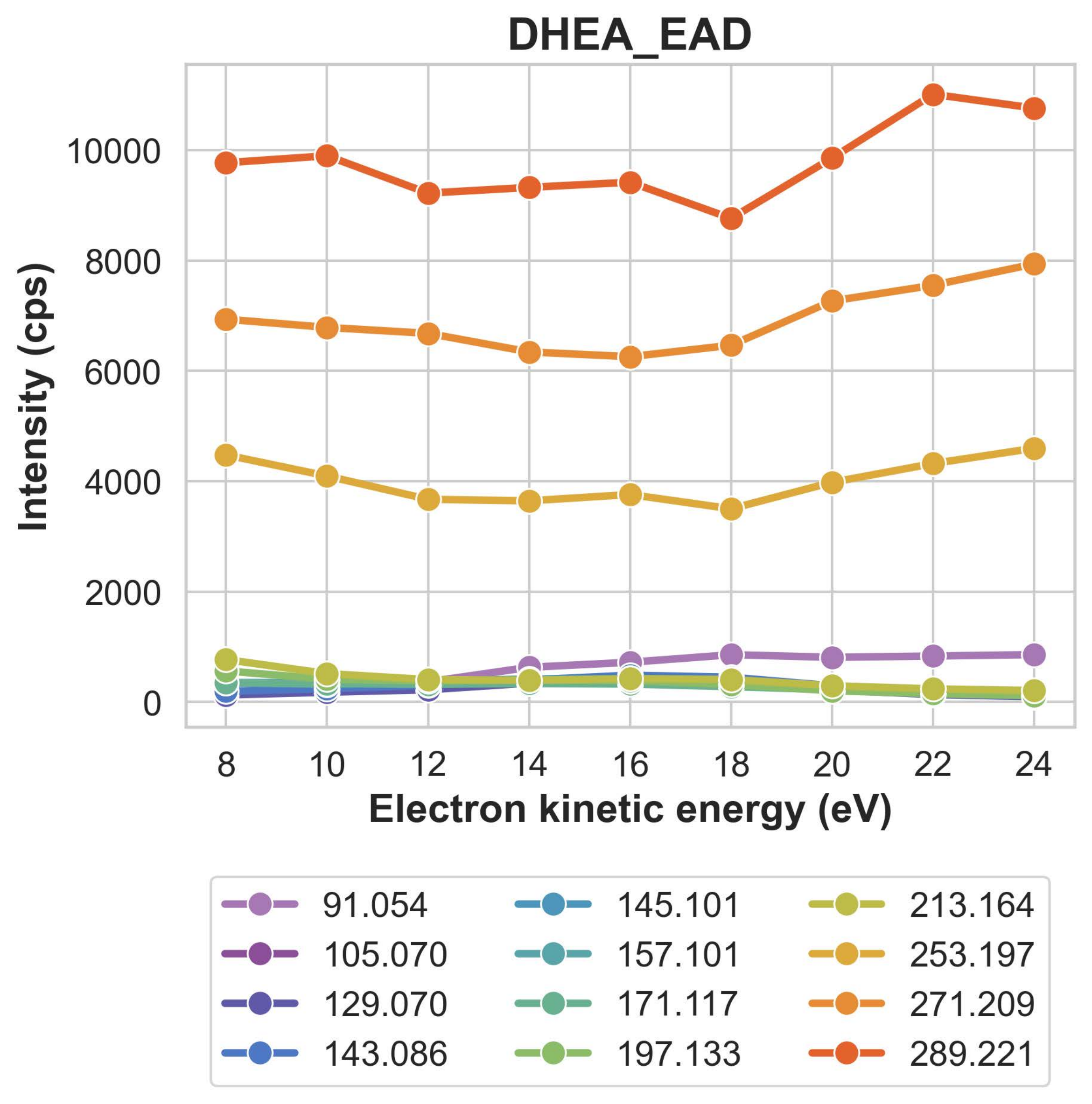
DHEA_EAD
Intensity (cps)
10000
8000
6000
4000
2000
0
8
10
12
14
16
18
20
22
24
Electron kinetic energy (eV)
91.054
105.070
129.070
143.086
145.101
157.101
171.117
197.133
213.164
253.197
271.209
289.221

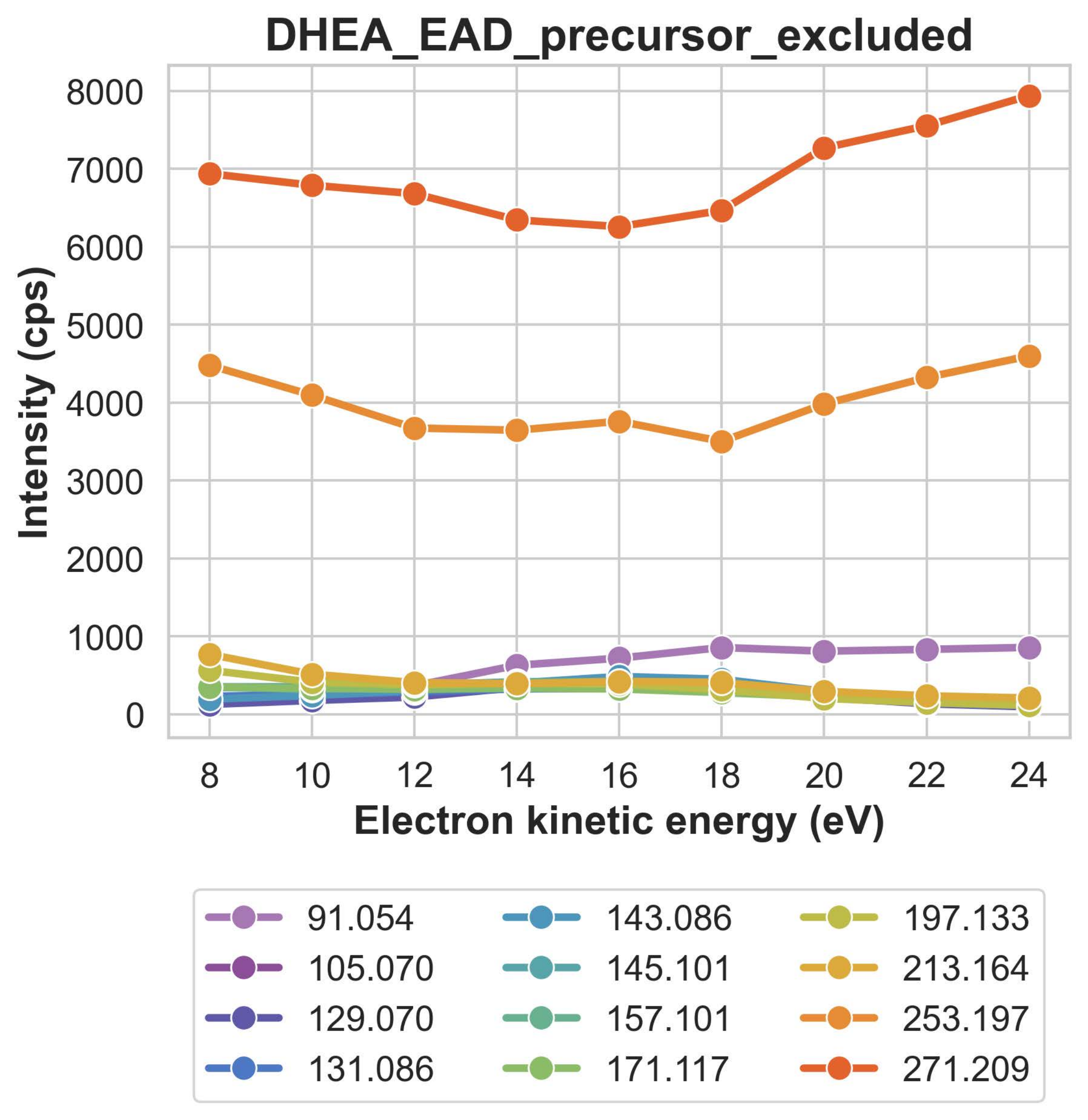

DHEA_EAD_precursor_excluded
Intensity (cps)
8000
7000
6000
5000
4000
3000
2000
1000
0
8
10
12
14
16
18
20
22
24
Electron kinetic energy (eV)
91.054
105.070
129.070
131.086
143.086
145.101
157.101
171.117
197.133
213.164
253.197
271.209

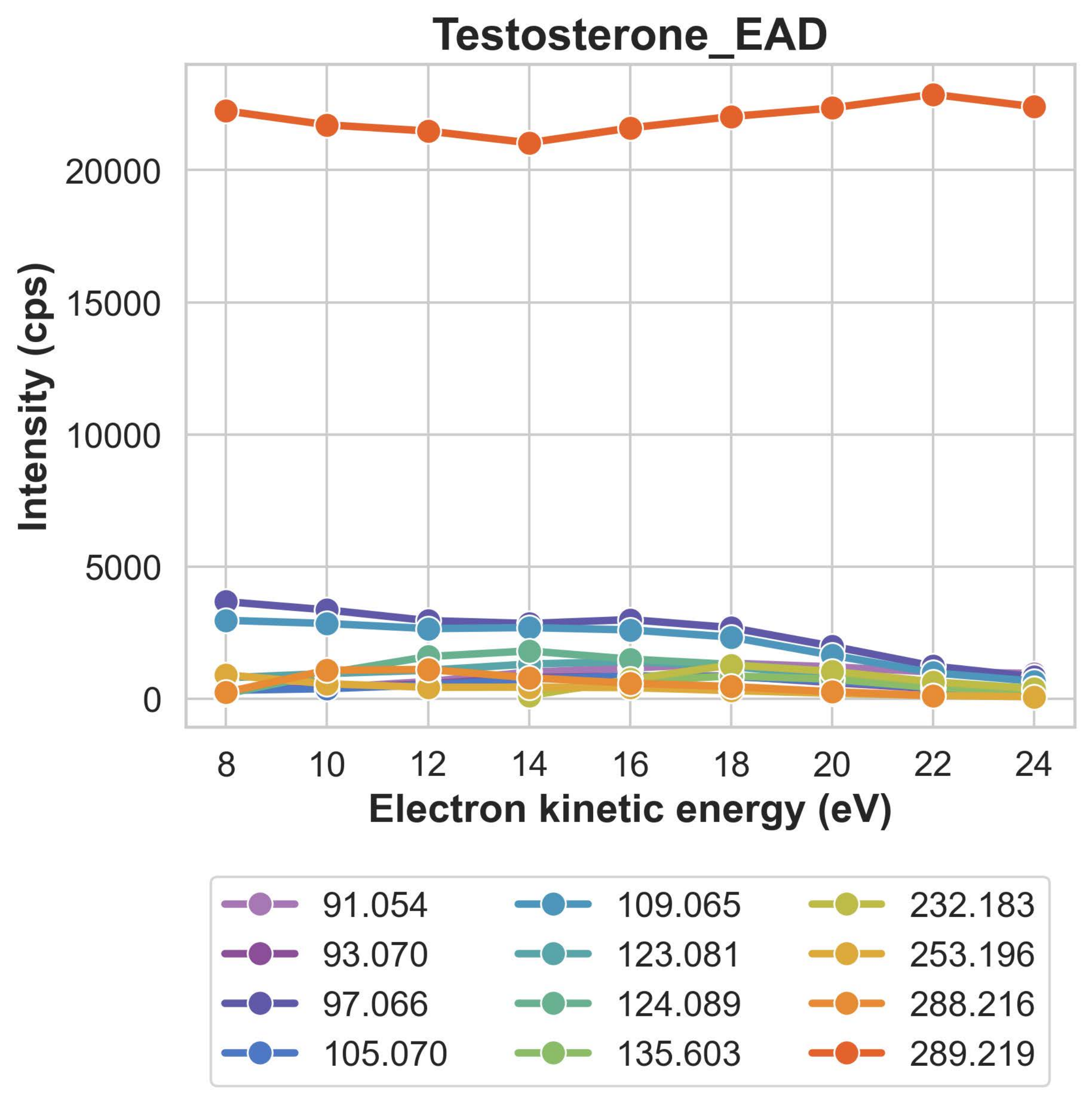
Testosterone_EAD
Intensity (cps)
20000
15000
10000
5000
0
8
10
12
14
16
18
20
22
24
Electron kinetic energy (eV)
91.054
93.070
97.066
105.070
109.065
123.081
124.089
135.603
232.183
253.196
288.216
289.219

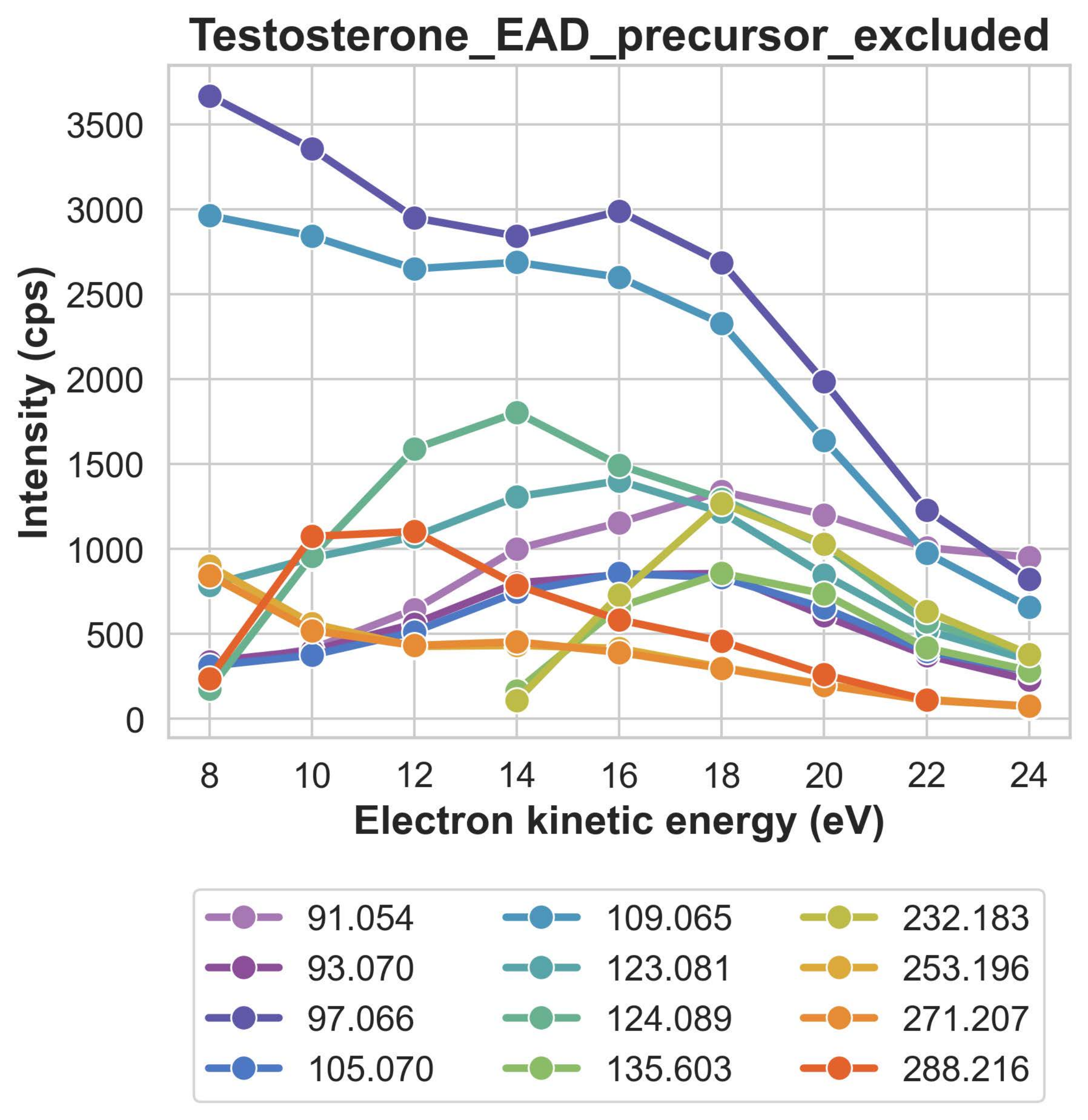

Testosterone_EAD_precursor_excluded
Intensity (cps)
0
500
1000
1500
2000
2500
3000
3500
Electron kinetic energy (eV)
8
10
12
14
16
18
20
22
24
91.054
93.070
97.066
105.070
109.065
123.081
124.089
135.603
232.183
253.196
271.207
288.216

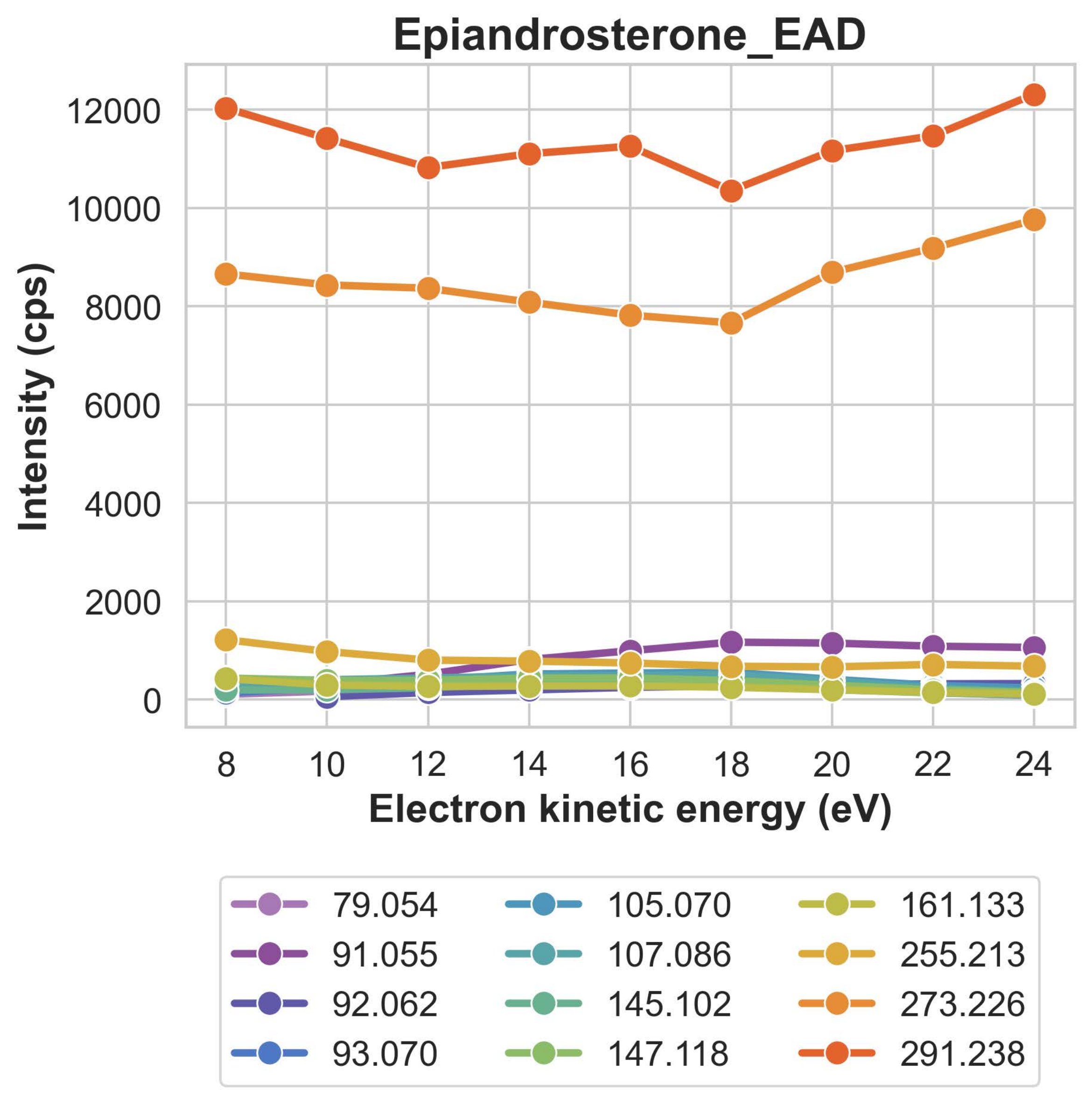
Epiandrosterone_EAD
Intensity (cps)
12000
10000
8000
6000
4000
2000
0
8
10
12
14
16
18
20
22
24
Electron kinetic energy (eV)
79.054
91.055
92.062
93.070
105.070
107.086
145.102
147.118
161.133
255.213
273.226
291.238

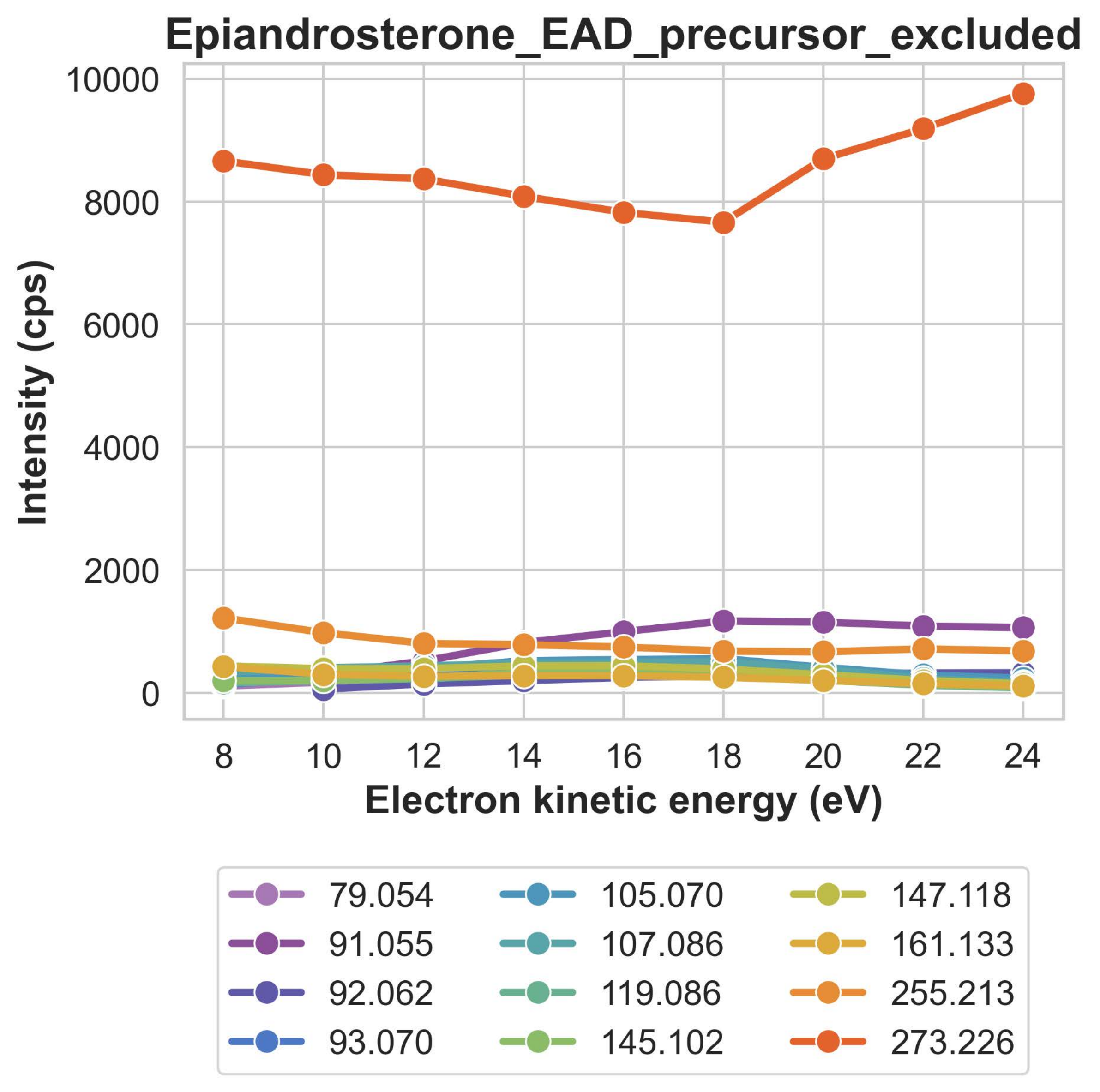

Epiandrosterone_EAD_precursor_excluded
Intensity (cps)
10000
8000
6000
4000
2000
0
8
10
12
14
16
18
20
22
24
Electron kinetic energy (eV)
79.054
91.055
92.062
93.070
105.070
107.086
119.086
145.102
147.118
161.133
255.213
273.226

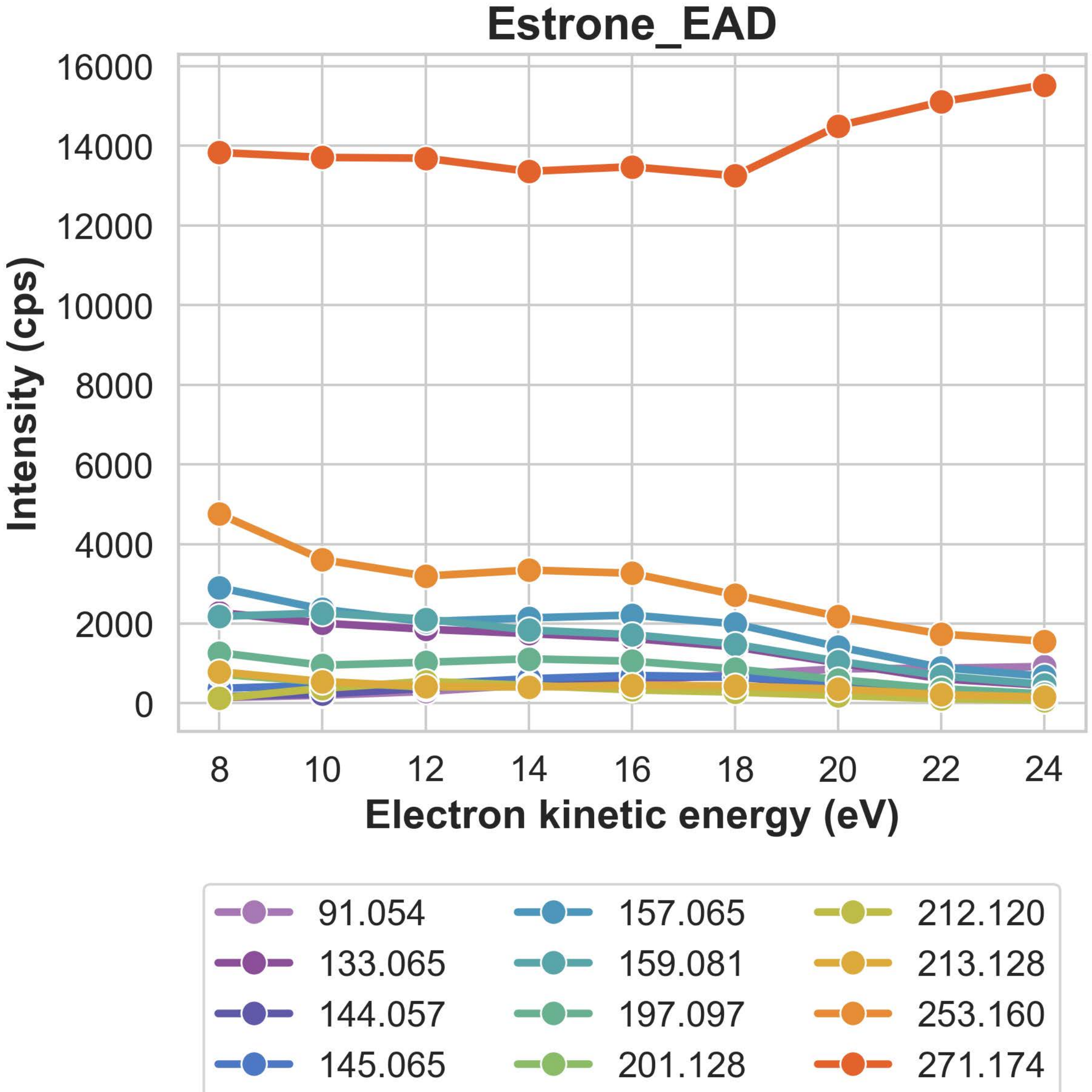
Estrone_EAD
Intensity (cps)
16000
14000
12000
10000
8000
6000
4000
2000
0
8
10
12
14
16
18
20
22
24
Electron kinetic energy (eV)
91.054
133.065
144.057
145.065
157.065
159.081
197.097
201.128
212.120
213.128
253.160
271.174

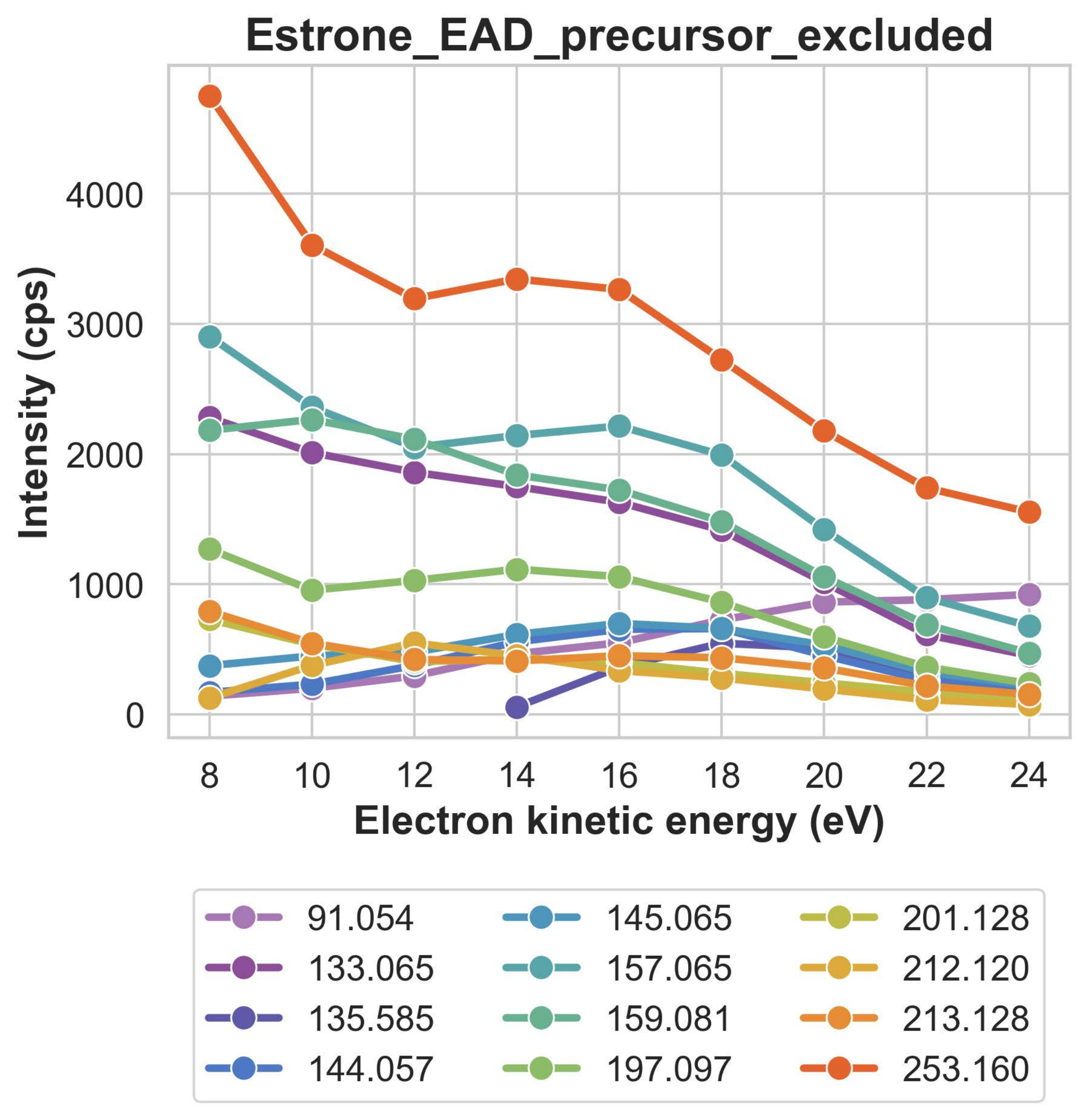

Estrone_EAD_precursor_excluded
Intensity (cps)
0
1000
2000
3000
4000
8
10
12
14
16
18
20
22
24
Electron kinetic energy (eV)
91.054
133.065
135.585
144.057
145.065
157.065
159.081
197.097
201.128
212.120
213.128
253.160

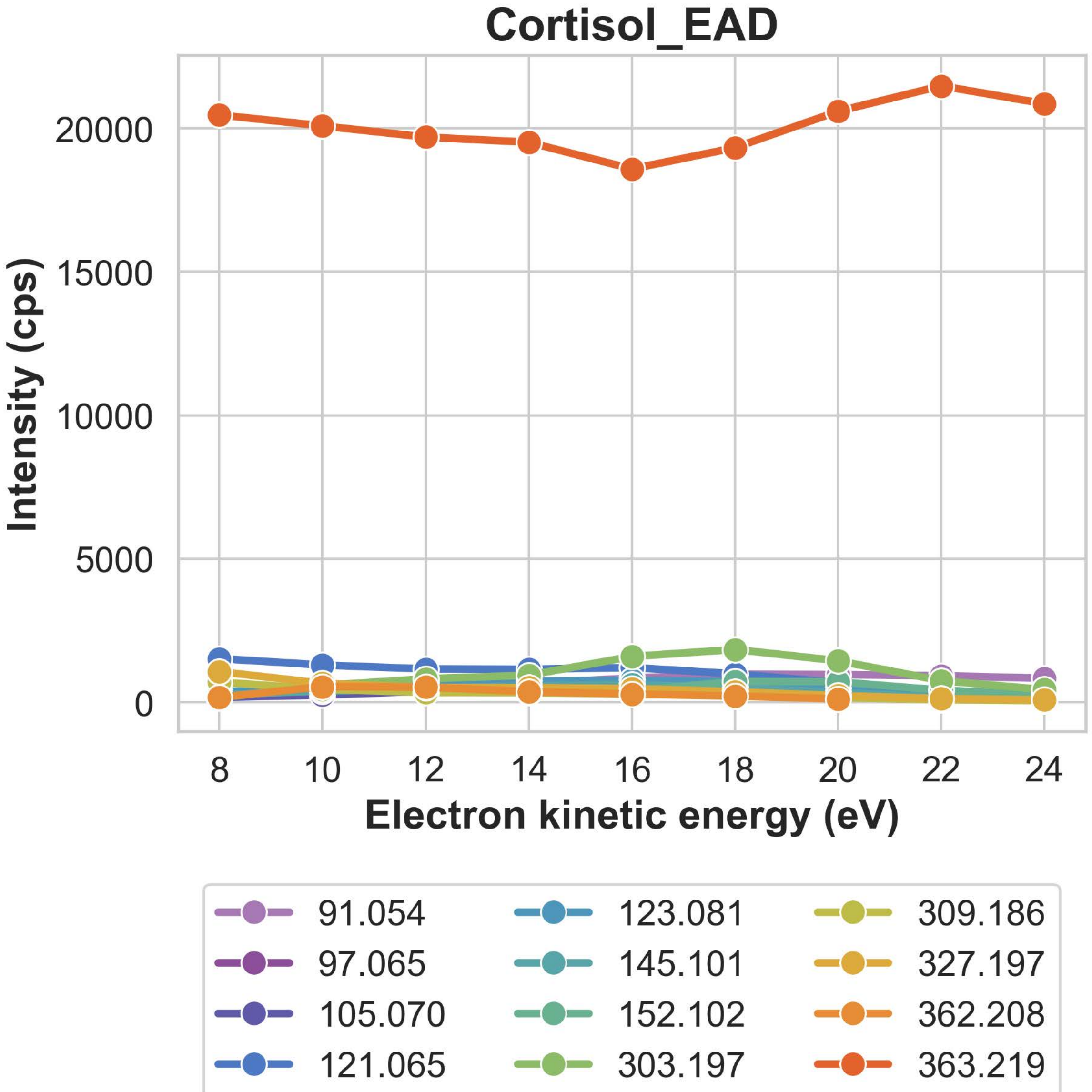
Cortisol_EAD
Intensity (cps)
20000
15000
10000
5000
0
8
10
12
14
16
18
20
22
24
Electron kinetic energy (eV)
91.054
97.065
105.070
121.065
123.081
145.101
152.102
303.197
309.186
327.197
362.208
363.219

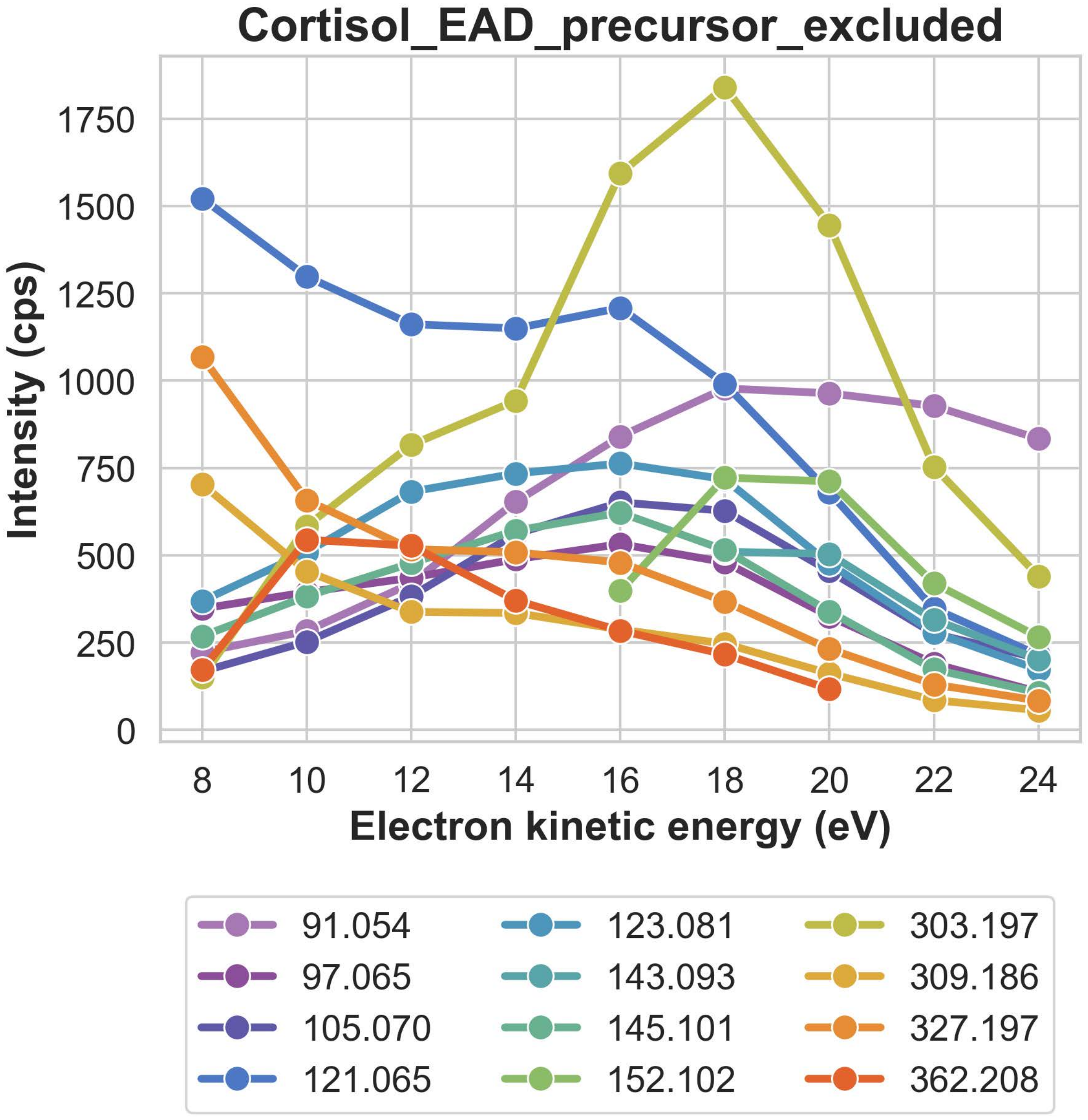
Cortisol_EAD_precursor_excluded
Intensity (cps)
0
250
500
750
1000
1250
1500
1750
8
10
12
14
16
18
20
22
24
Electron kinetic energy (eV)
91.054
97.065
105.070
121.065
123.081
143.093
145.101
152.102
303.197
309.186
327.197
362.208

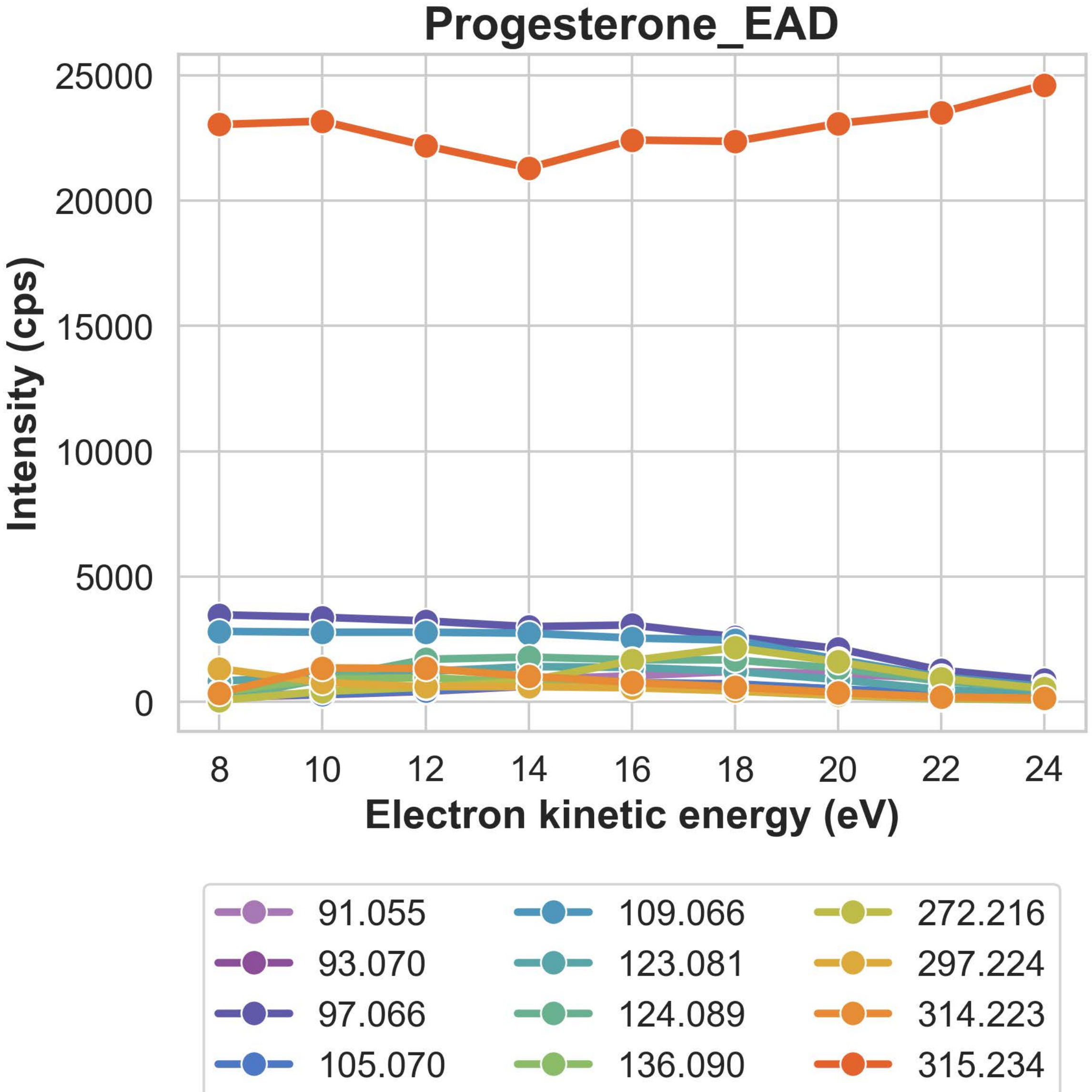

Progesterone_EAD
Intensity (cps)
25000
20000
15000
10000
5000
0
8
10
12
14
16
18
20
22
24
Electron kinetic energy (eV)
91.055
93.070
97.066
105.070
109.066
123.081
124.089
136.090
272.216
297.224
314.223
315.234

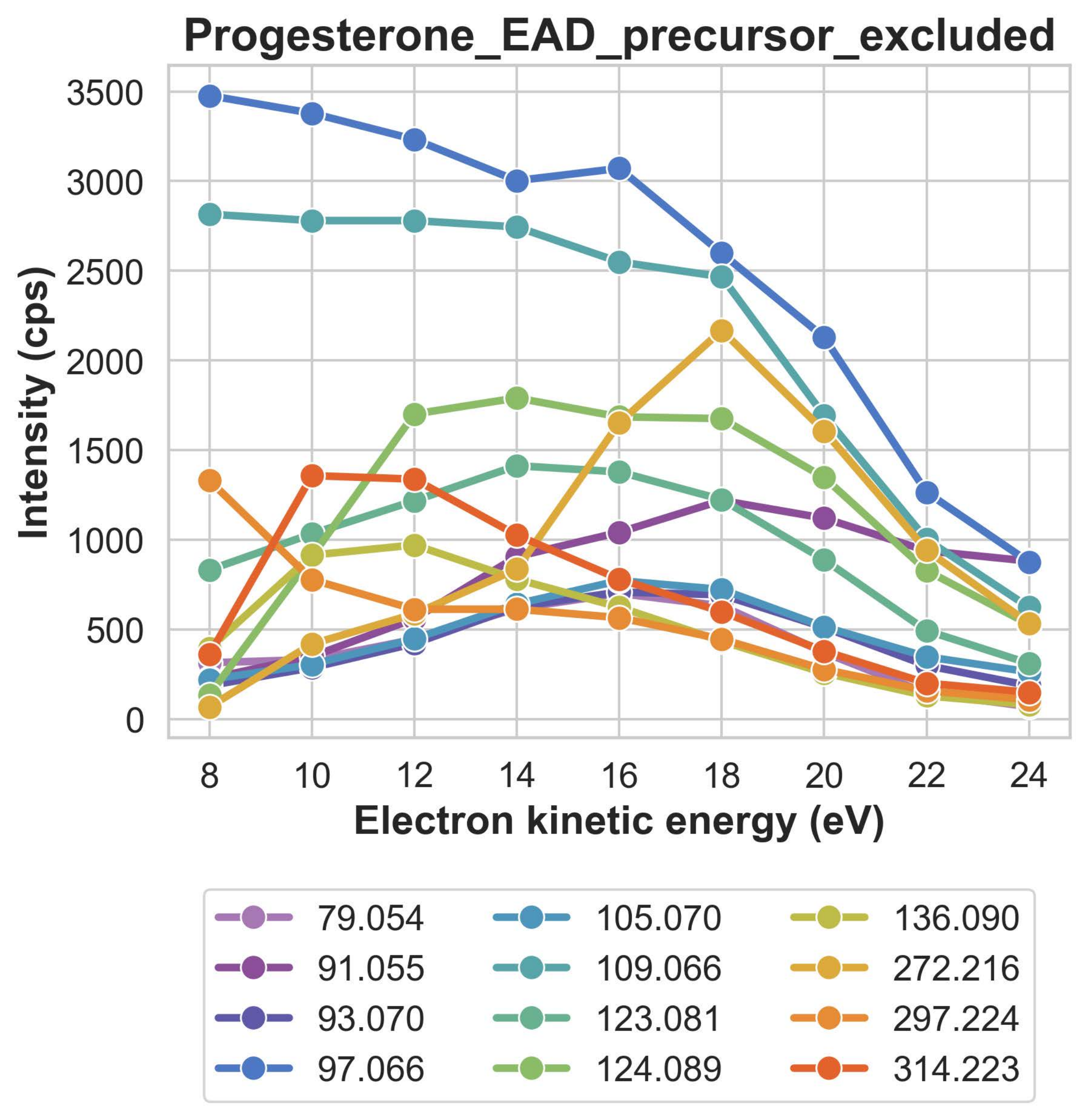

Progesterone_EAD_precursor_excluded
Intensity (cps)
3500
3000
2500
2000
1500
1000
500
0
8
10
12
14
16
18
20
22
24
Electron kinetic energy (eV)
79.054
91.055
93.070
97.066
105.070
109.066
123.081
124.089
136.090
272.216
297.224
314.223

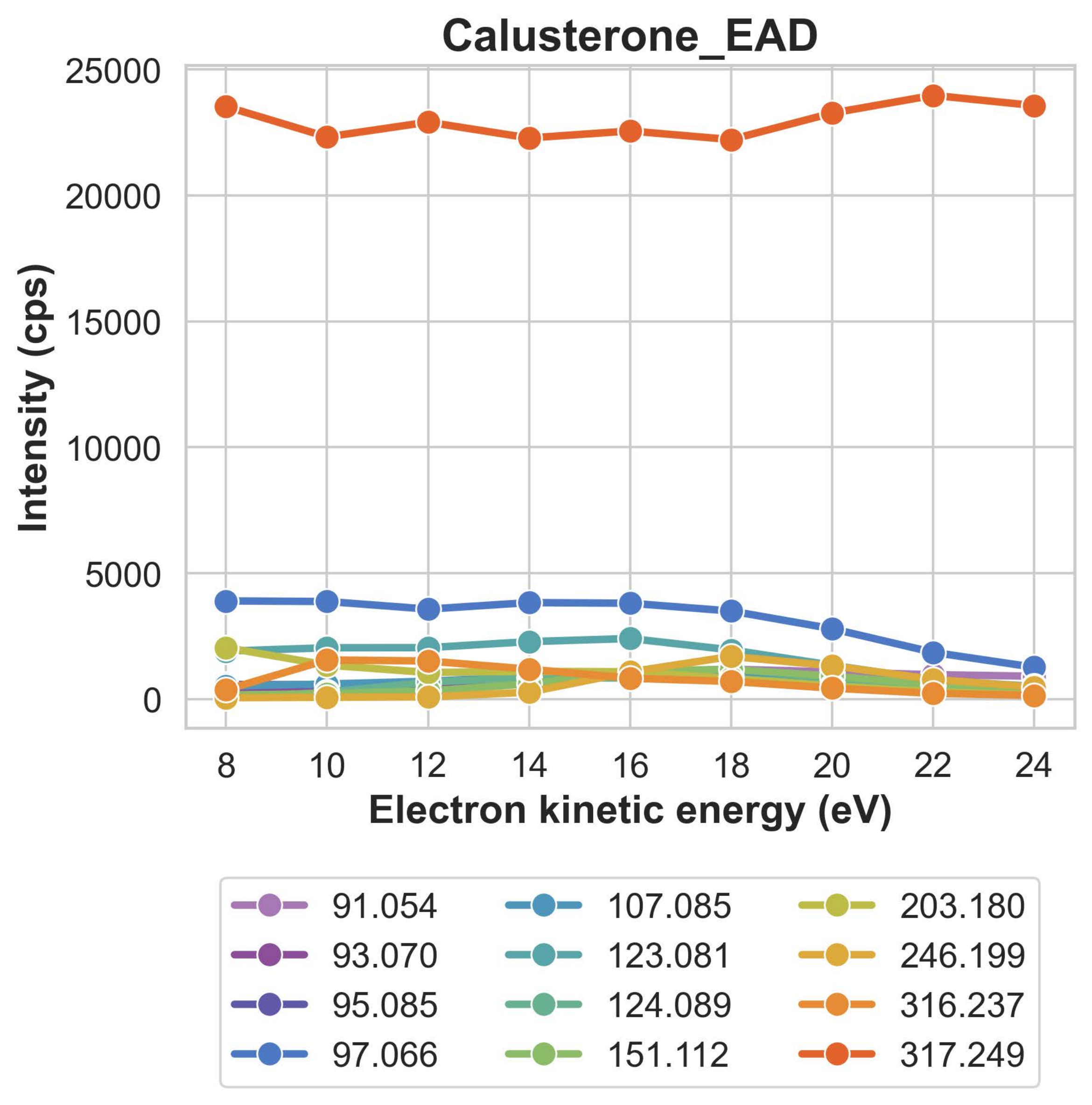
Calusterone_EAD
Intensity (cps)
25000
20000
15000
10000
5000
0
8
10
12
14
16
18
20
22
24
Electron kinetic energy (eV)
91.054
93.070
95.085
97.066
107.085
123.081
124.089
151.112
203.180
246.199
316.237
317.249

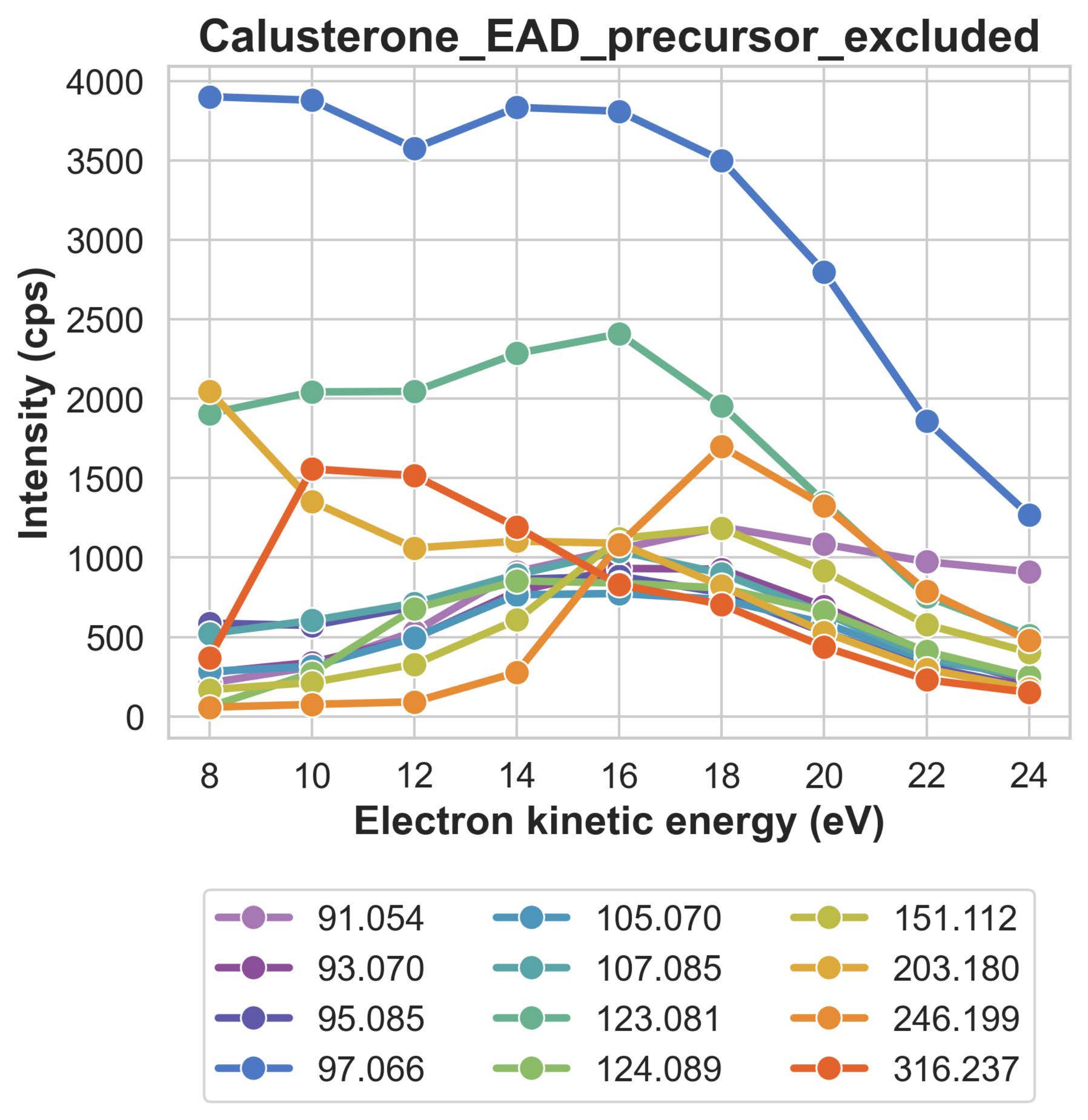

Calusterone_EAD_precursor_excluded
4000
3500
3000
2500
2000
1500
1000
500
0
Intensity (cps)
8
10
12
14
16
18
20
22
24
Electron kinetic energy (eV)
91.054
93.070
95.085
97.066
105.070
107.085
123.081
124.089
151.112
203.180
246.199
316.237

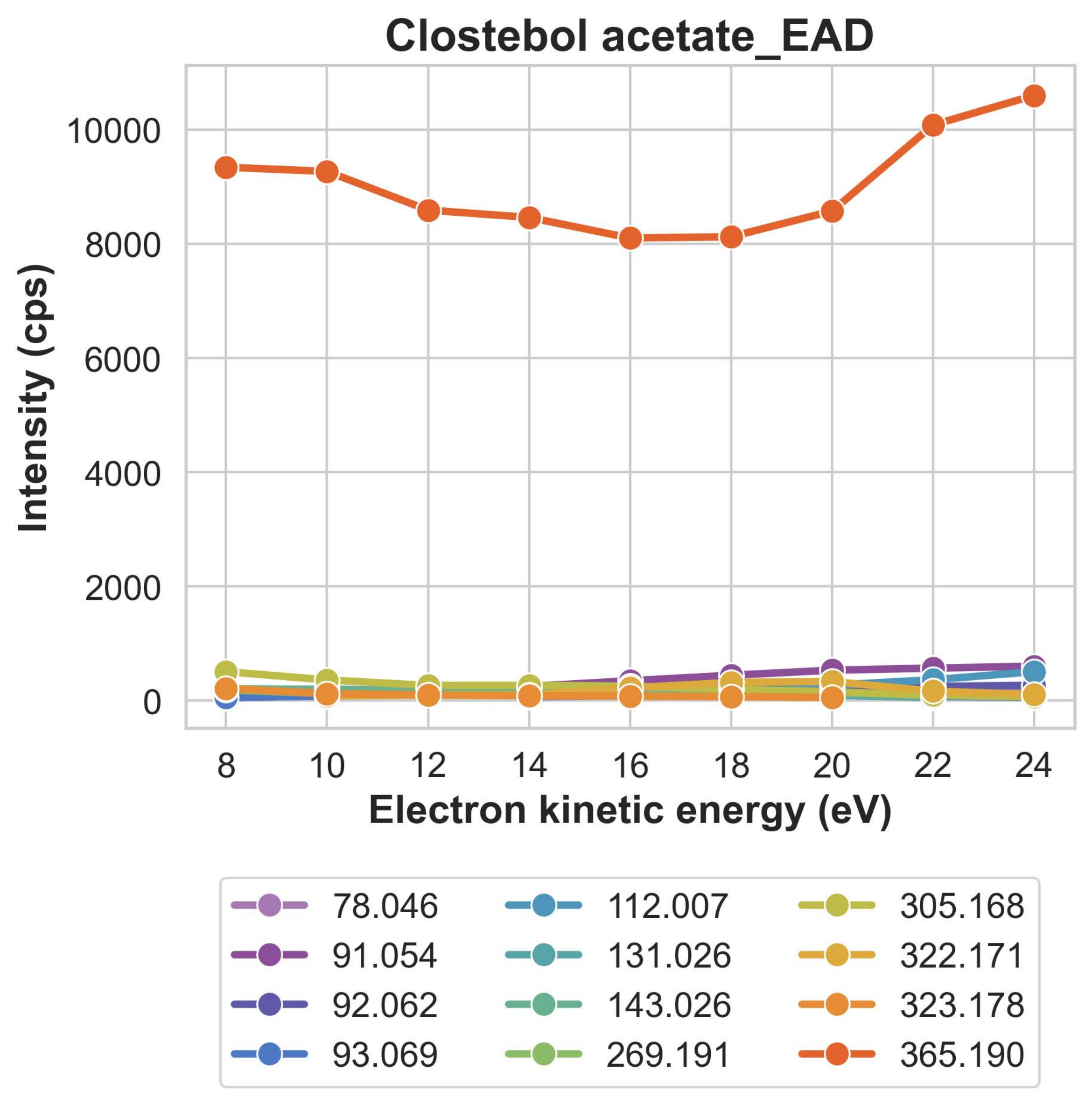

Clostebol acetate_EAD
Intensity (cps)
10000
8000
6000
4000
2000
0
8
10
12
14
16
18
20
22
24
Electron kinetic energy (eV)
78.046
91.054
92.062
93.069
112.007
131.026
143.026
269.191
305.168
322.171
323.178
365.190

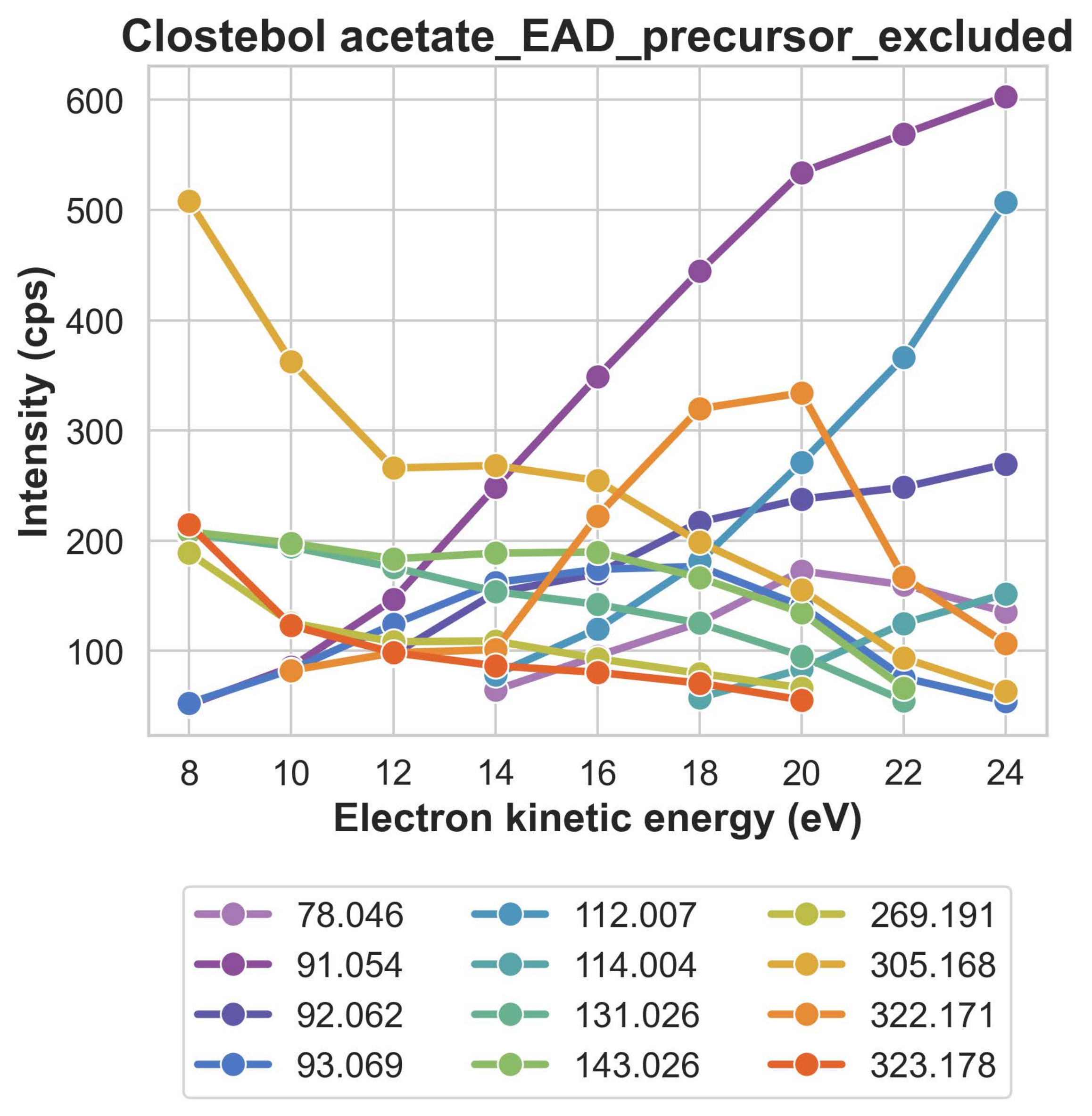

Clostebol acetate_EAD_precursor_excluded
Intensity (cps)
600
500
400
300
200
100
8
10
12
14
16
18
20
22
24
Electron kinetic energy (eV)
78.046
91.054
92.062
93.069
112.007
114.004
131.026
143.026
269.191
305.168
322.171
323.178

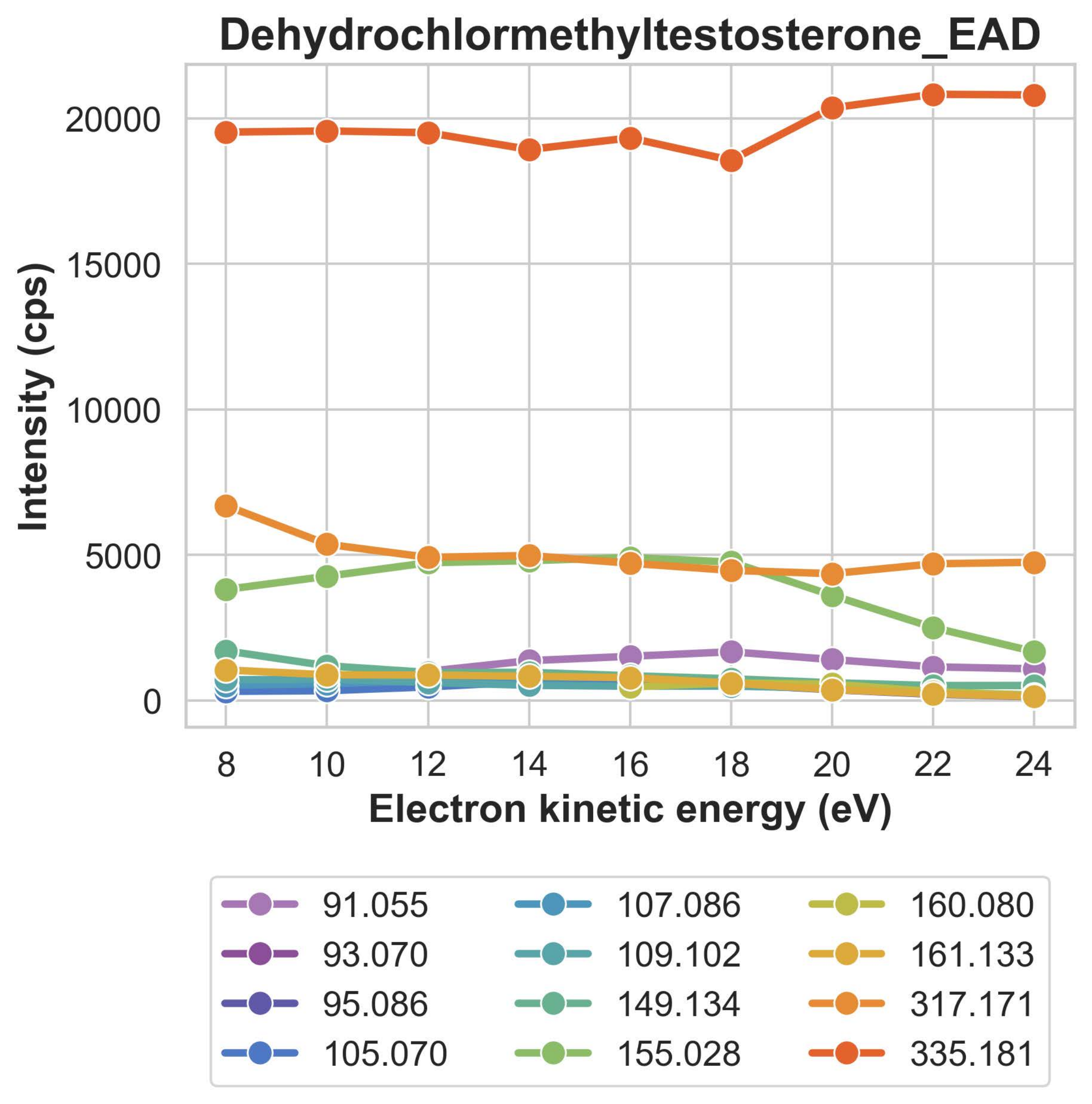
Dehydrochlormethyltestosterone_EAD
Intensity (cps)
20000
15000
10000
5000
0
8
10
12
14
16
18
20
22
24
Electron kinetic energy (eV)
91.055
93.070
95.086
105.070
107.086
109.102
149.134
155.028
160.080
161.133
317.171
335.181

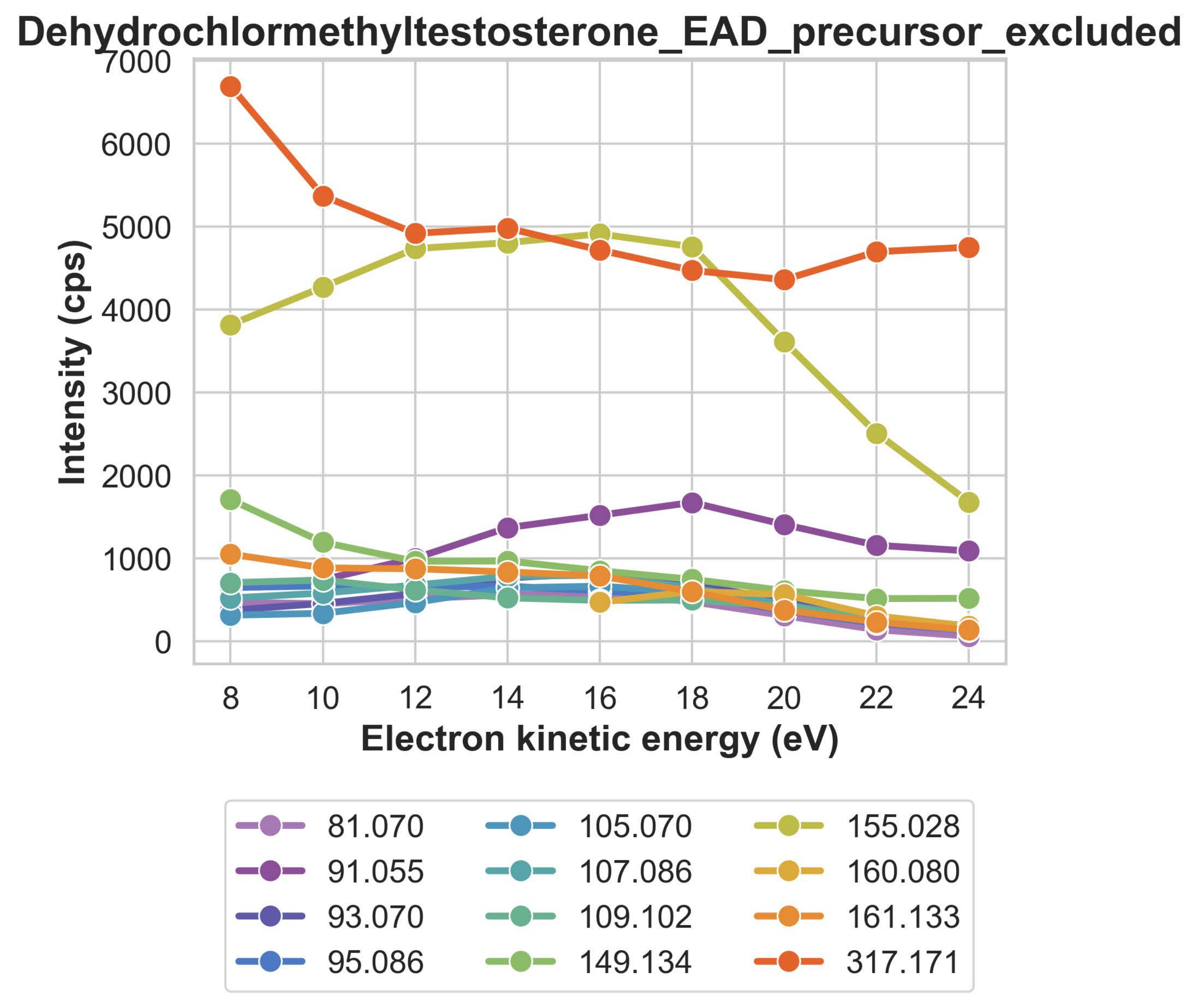

Dehydrochlormethyltestosterone_EAD_precursor_excluded
Intensity (cps)
0
1000
2000
3000
4000
5000
6000
7000
8
10
12
14
16
18
20
22
24
Electron kinetic energy (eV)
81.070
91.055
93.070
95.086
105.070
107.086
109.102
149.134
155.028
160.080
161.133
317.171

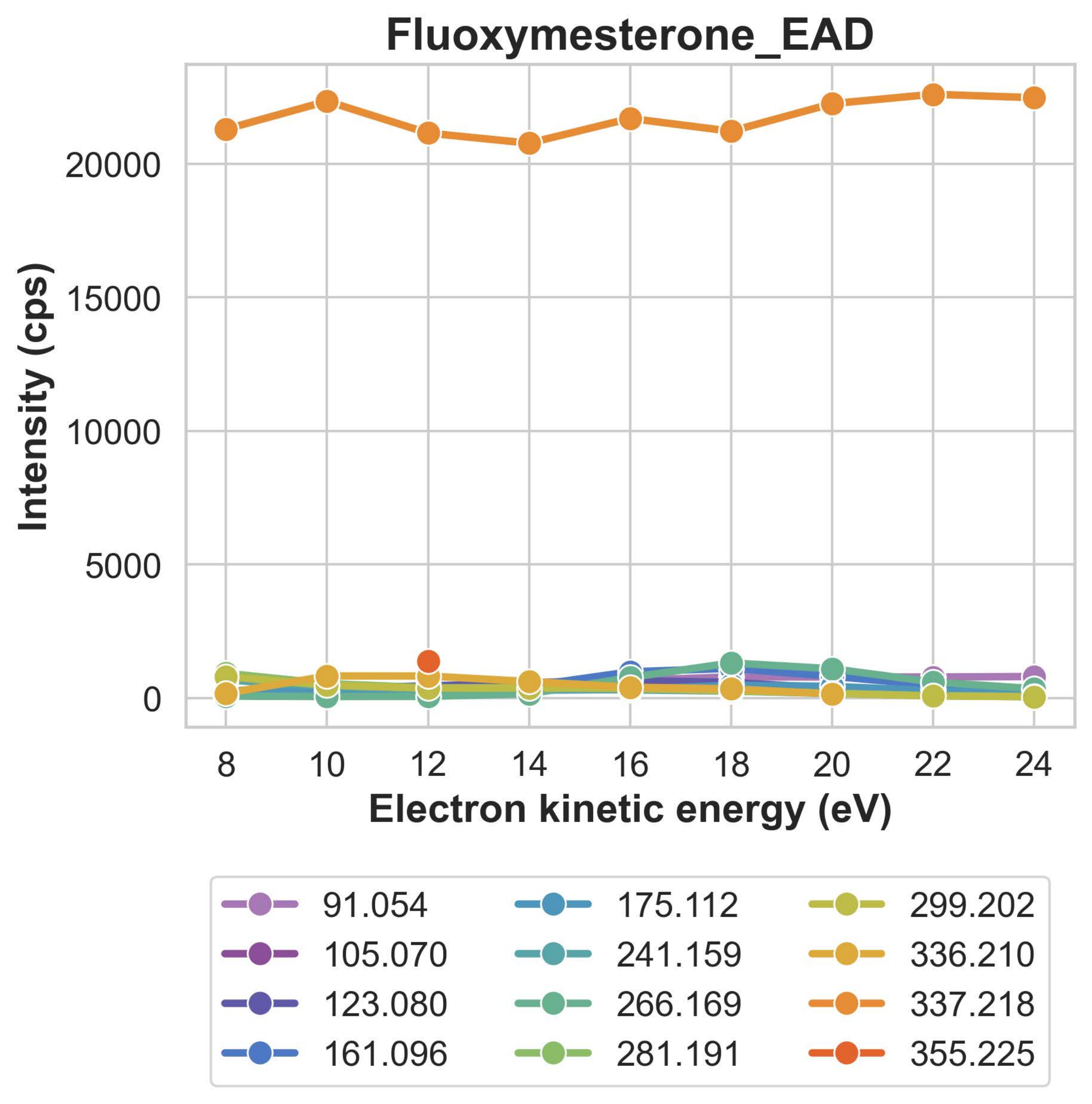
Fluoxymesterone_EAD
Intensity (cps)
20000
15000
10000
5000
0
8
10
12
14
16
18
20
22
24
Electron kinetic energy (eV)
91.054
105.070
123.080
161.096
175.112
241.159
266.169
281.191
299.202
336.210
337.218
355.225

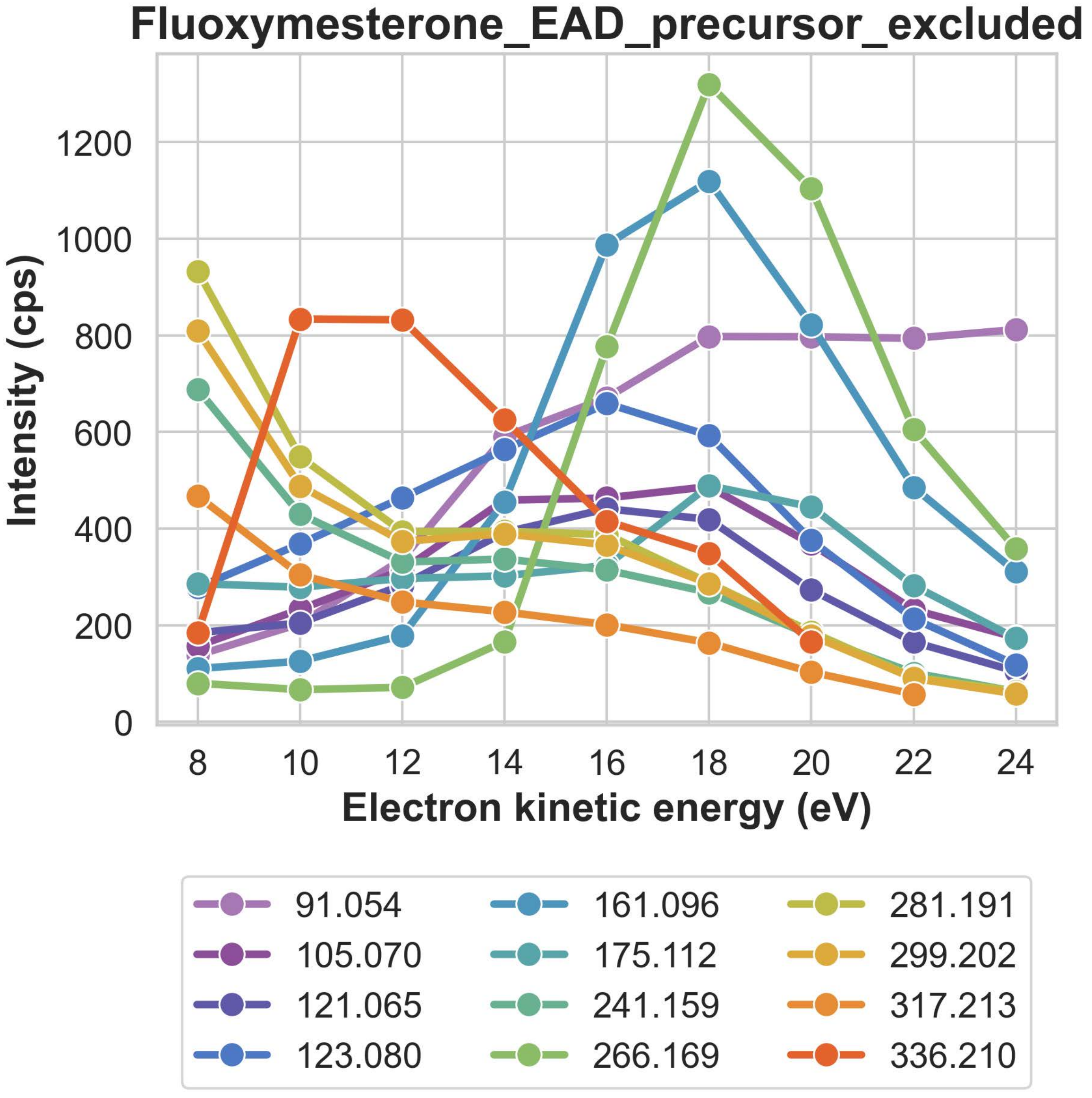
Fluoxymesterone_EAD_precursor_excluded
Intensity (cps)
0
200
400
600
800
1000
1200
8
10
12
14
16
18
20
22
24
Electron kinetic energy (eV)
91.054
105.070
121.065
123.080
161.096
175.112
241.159
266.169
281.191
299.202
317.213
336.210

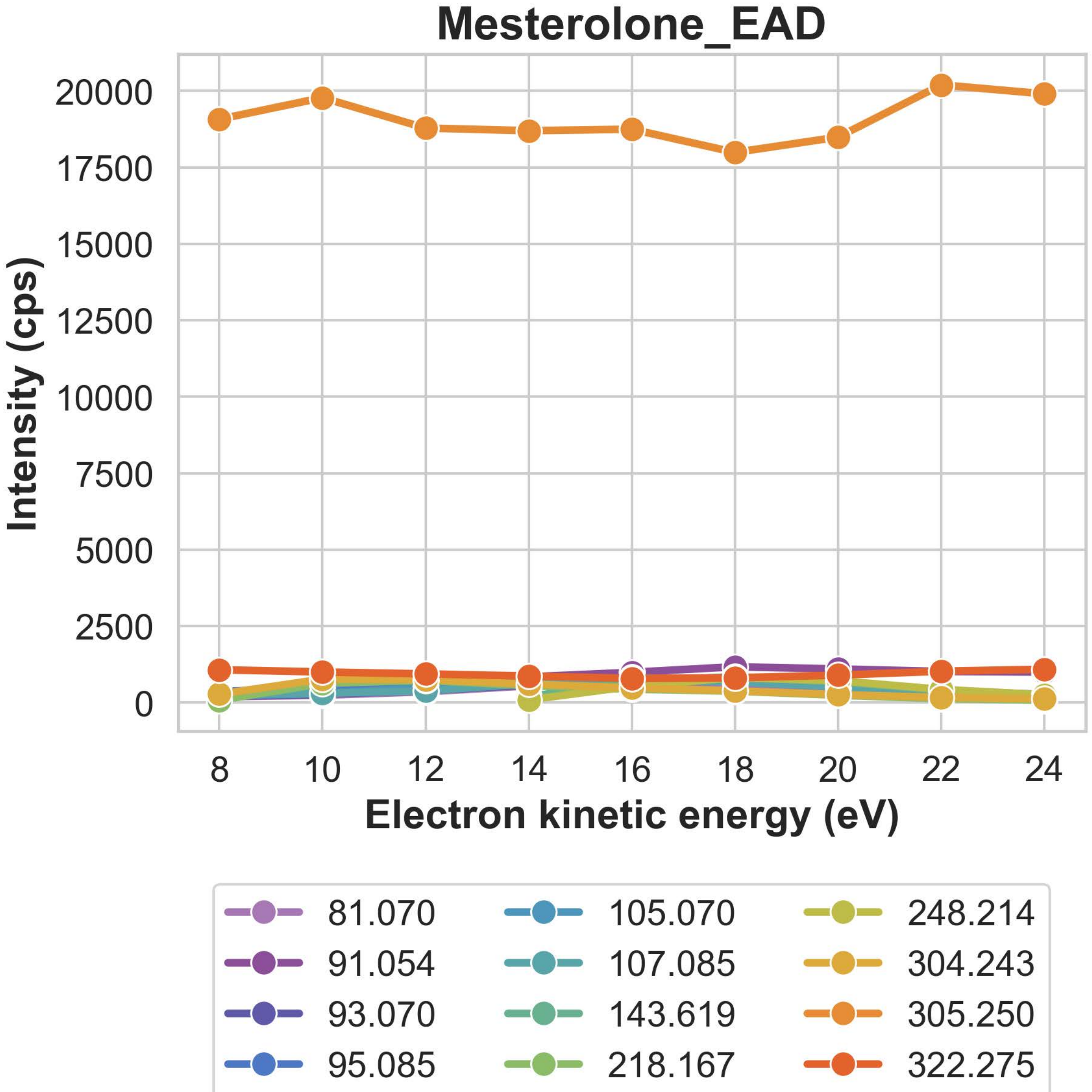

Mesterolone_EAD
Intensity (cps)
20000
17500
15000
12500
10000
7500
5000
2500
0
8
10
12
14
16
18
20
22
24
Electron kinetic energy (eV)
81.070
91.054
93.070
95.085
105.070
107.085
143.619
218.167
248.214
304.243
305.250
322.275

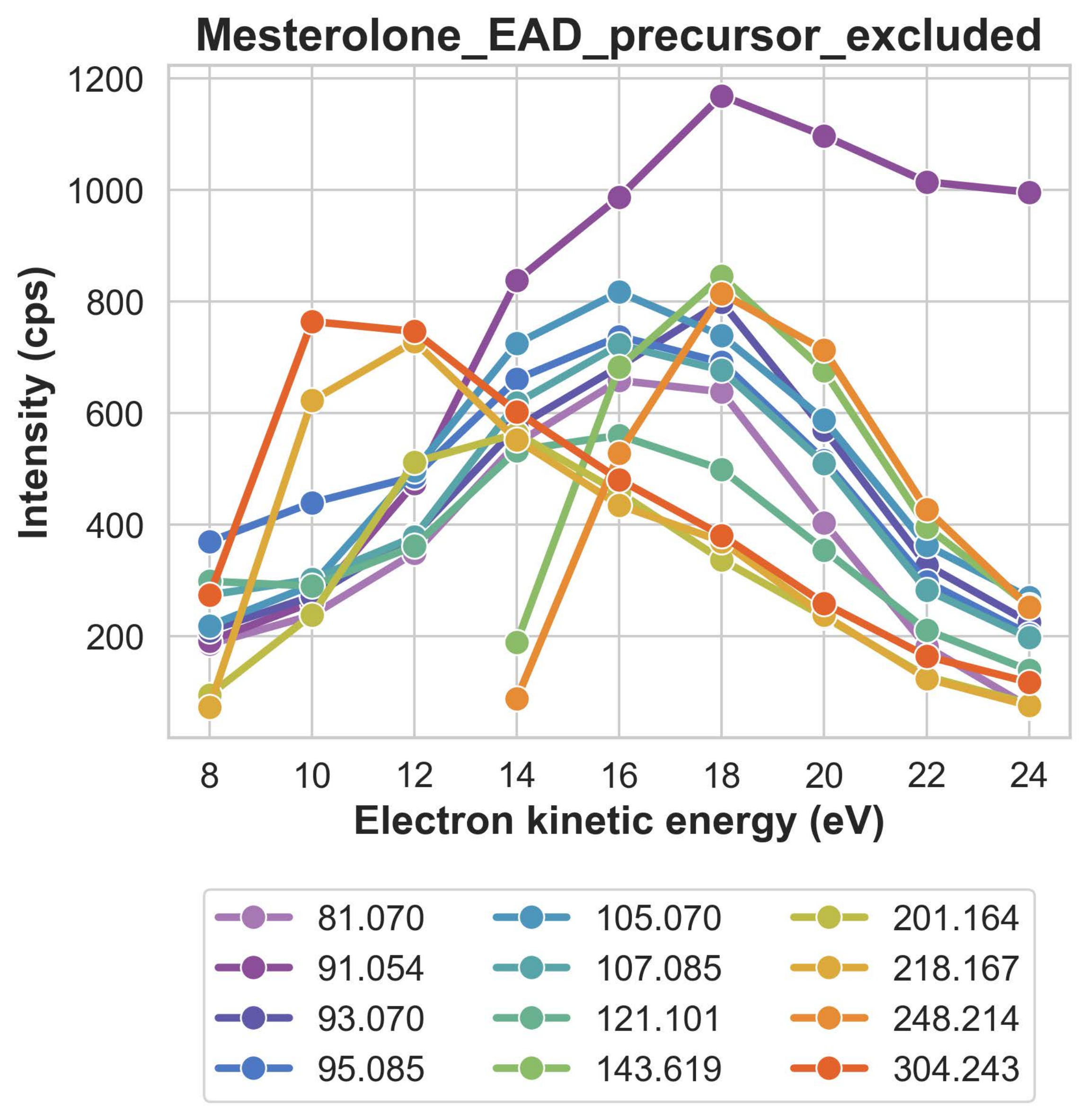

Mesterolone_EAD_precursor_excluded
Intensity (cps)
1200
1000
800
600
400
200
8
10
12
14
16
18
20
22
24
Electron kinetic energy (eV)
81.070
91.054
93.070
95.085
105.070
107.085
121.101
143.619
201.164
218.167
248.214
304.243

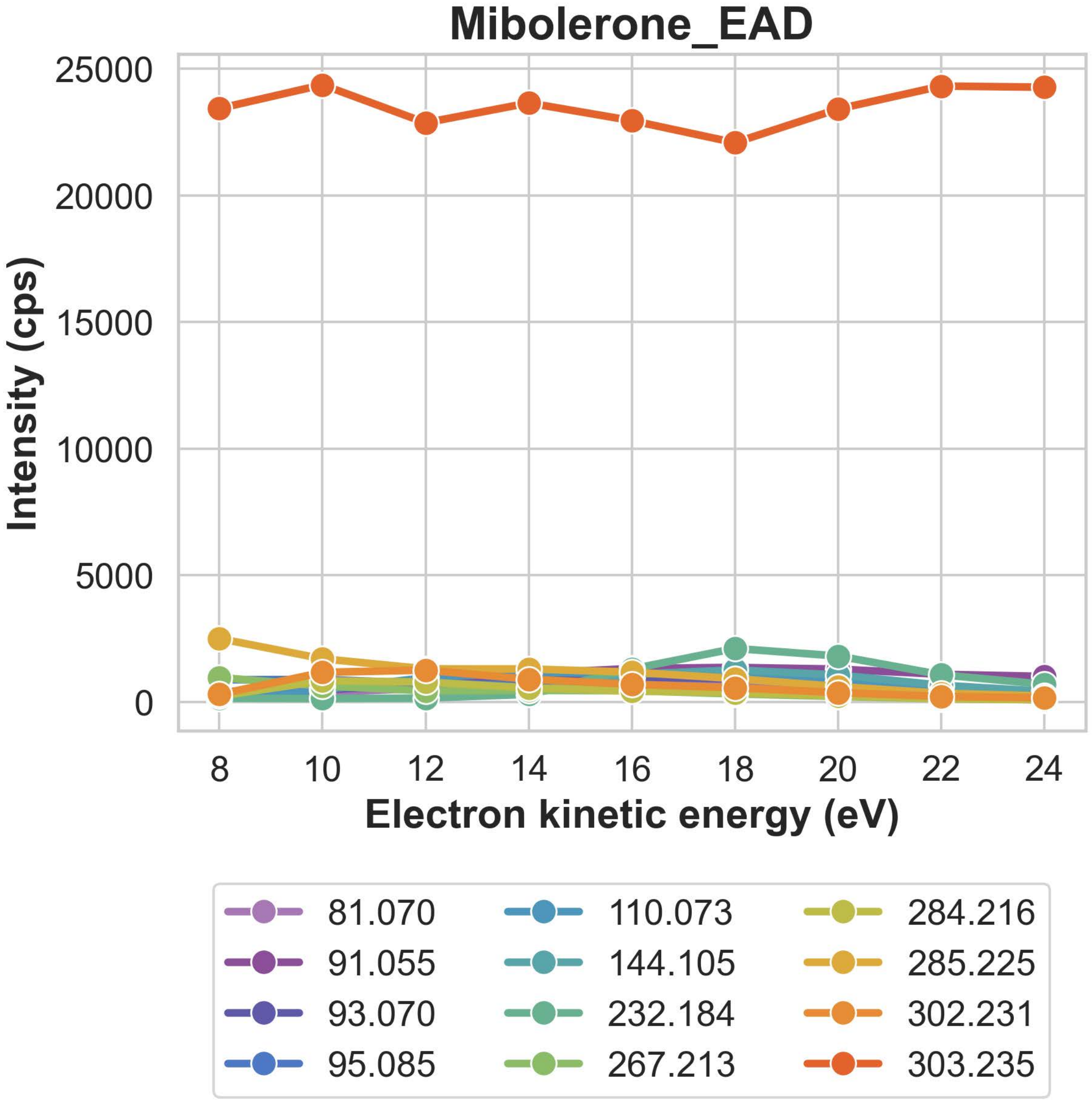

Mibolerone_EAD
Intensity (cps)
25000
20000
15000
10000
5000
0
8
10
12
14
16
18
20
22
24
Electron kinetic energy (eV)
81.070
91.055
93.070
95.085
110.073
144.105
232.184
267.213
284.216
285.225
302.231
303.235

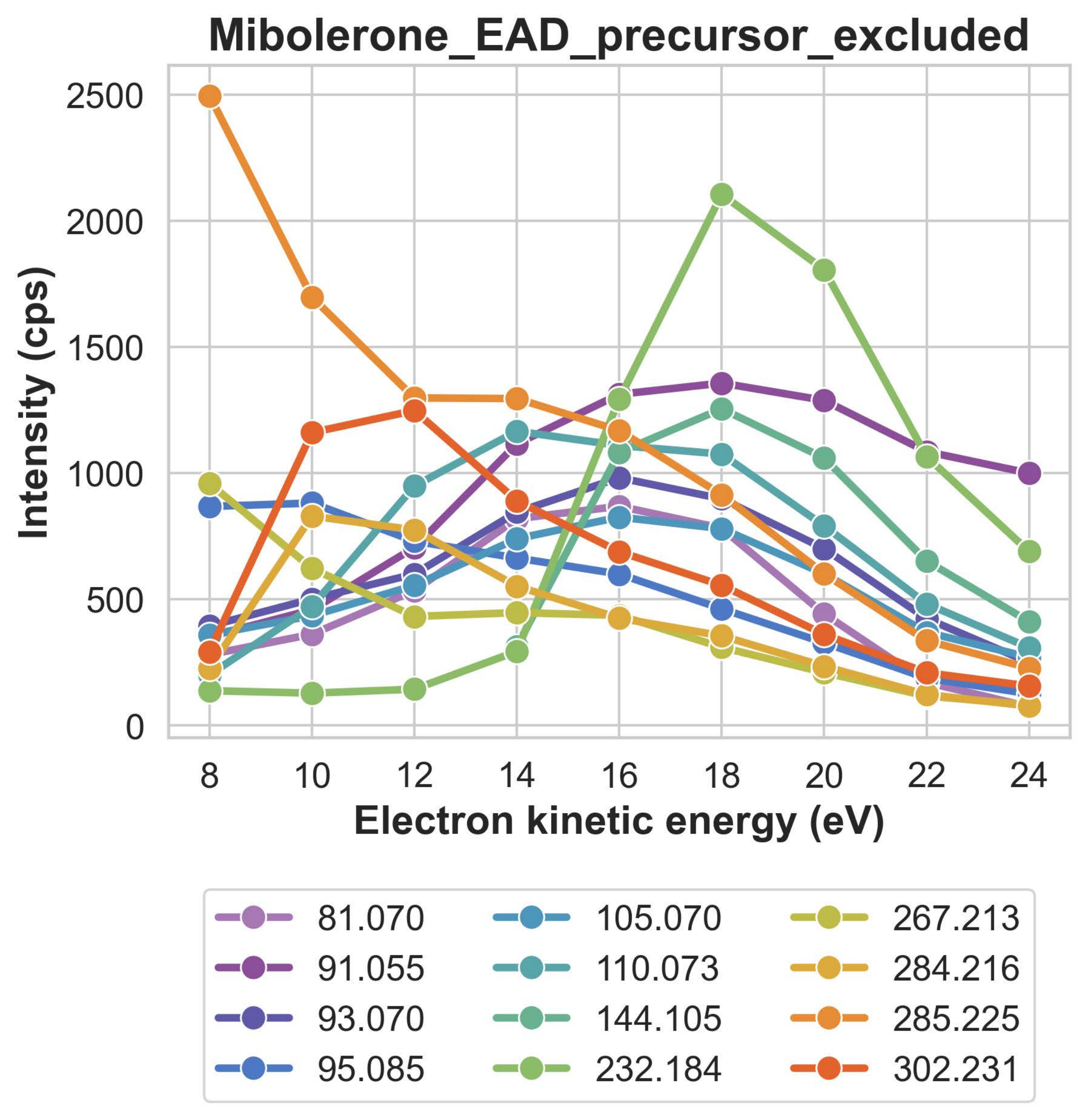
Mibolerone_EAD_precursor_excluded
Intensity (cps)
2500
2000
1500
1000
500
0
8
10
12
14
16
18
20
22
24
Electron kinetic energy (eV)
81.070
91.055
93.070
95.085
105.070
110.073
144.105
232.184
267.213
284.216
285.225
302.231

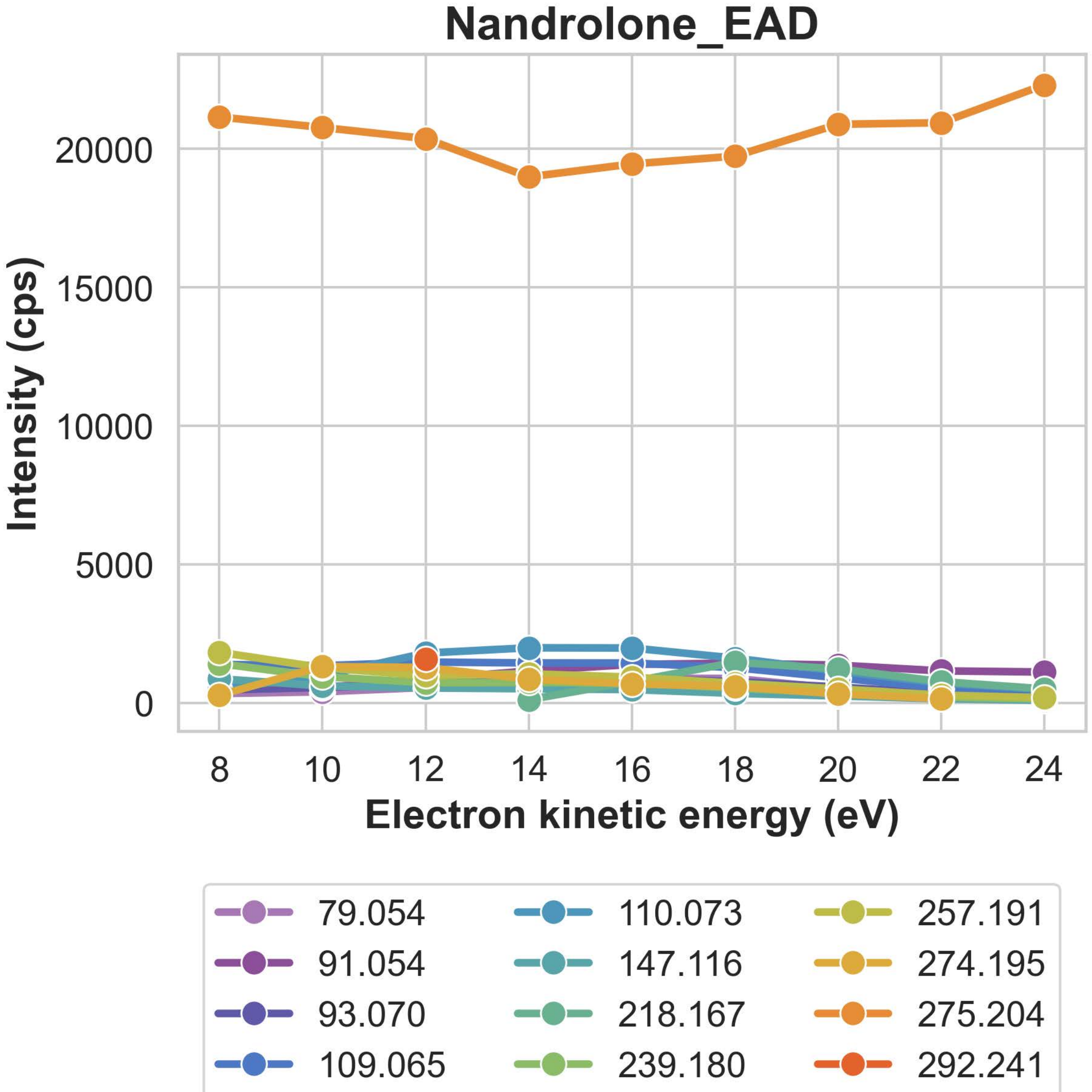

Nandrolone_EAD
Intensity (cps)
20000
15000
10000
5000
0
8
10
12
14
16
18
20
22
24
Electron kinetic energy (eV)
79.054
91.054
93.070
109.065
110.073
147.116
218.167
239.180
257.191
274.195
275.204
292.241

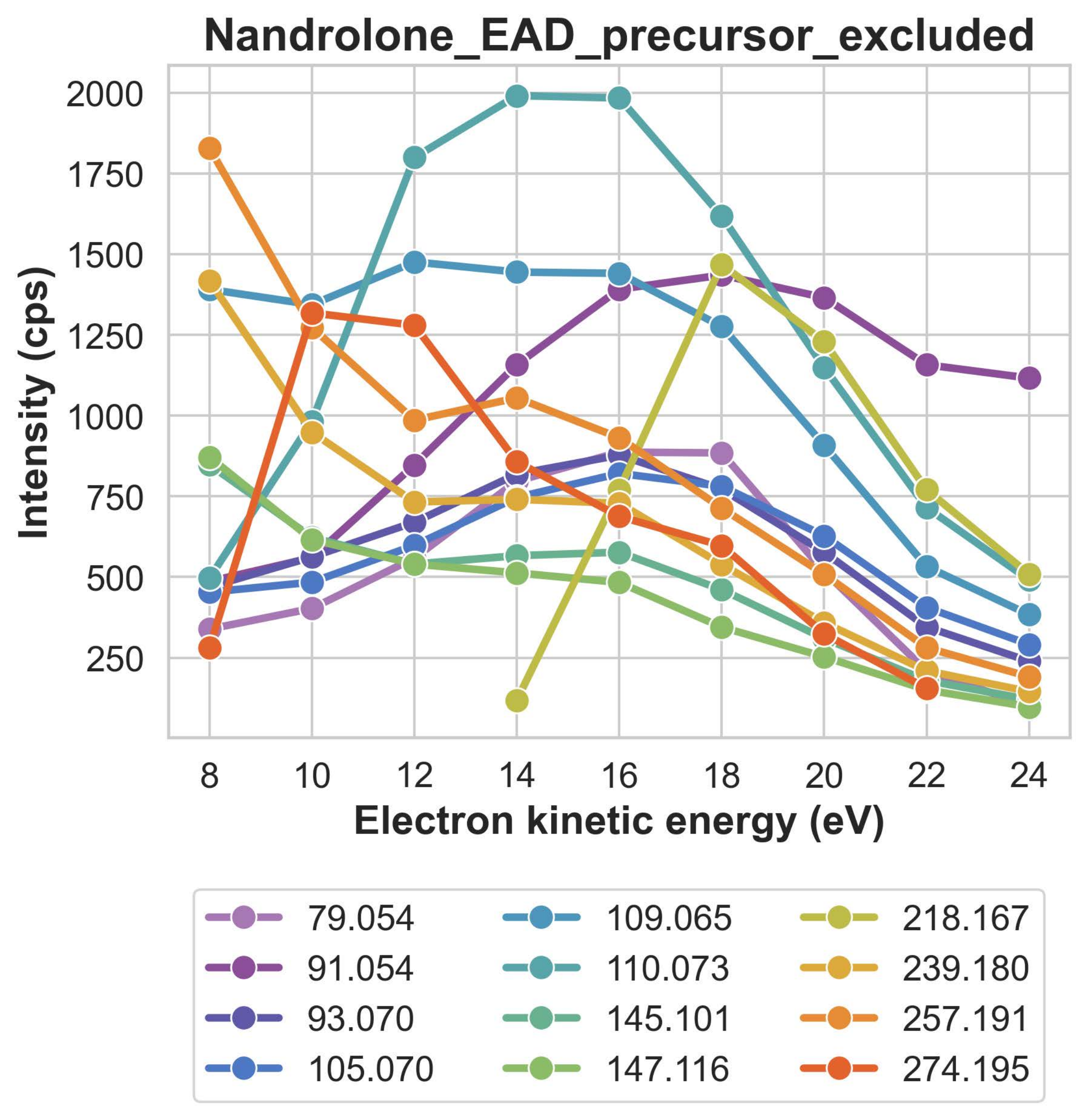
Nandrolone_EAD_precursor_excluded
Intensity (cps)
2000
1750
1500
1250
1000
750
500
250
8
10
12
14
16
18
20
22
24
Electron kinetic energy (eV)
79.054
91.054
93.070
105.070
109.065
110.073
145.101
147.116
218.167
239.180
257.191
274.195

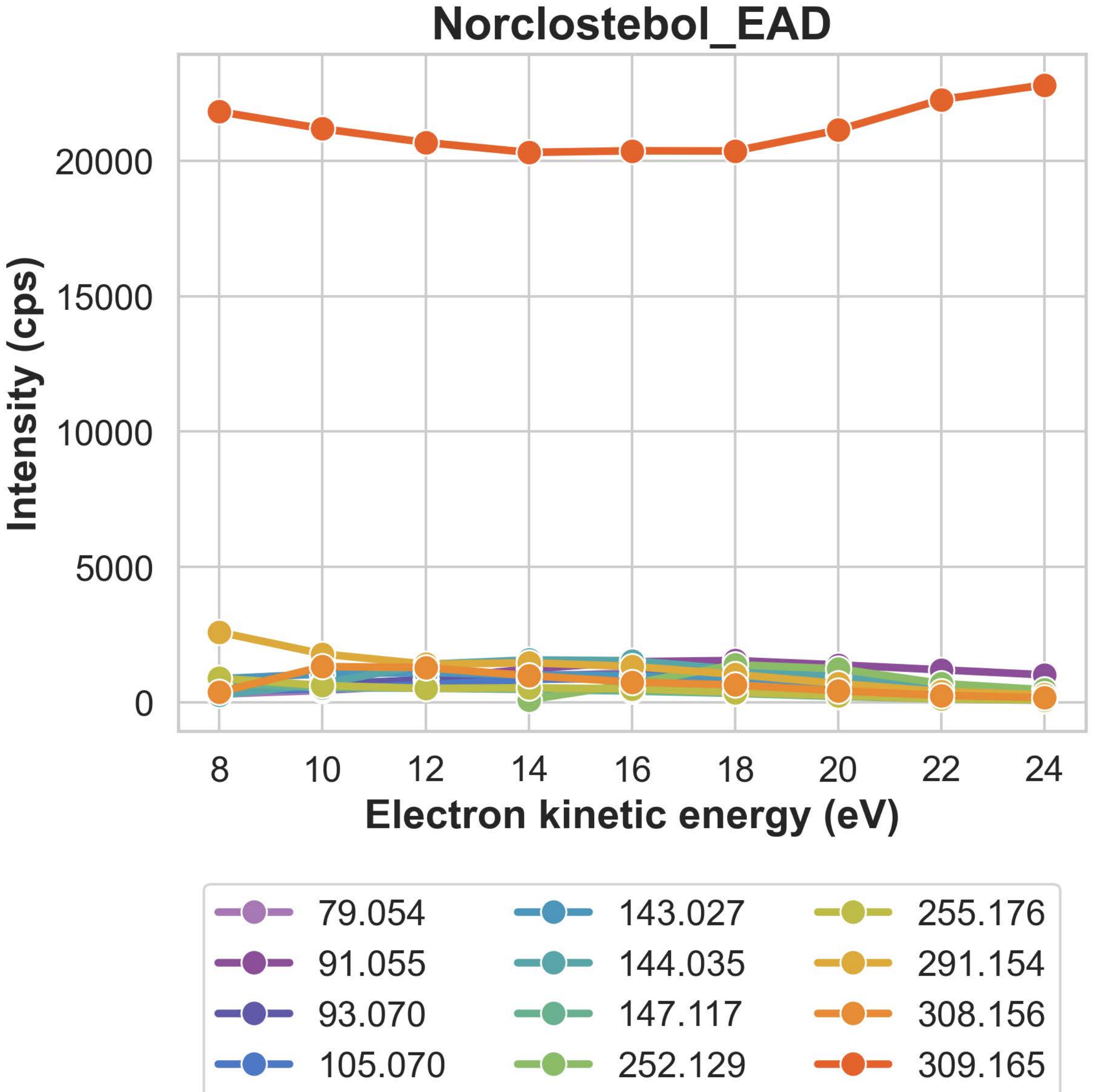
Norclostebol_EAD
Intensity (cps)
20000
15000
10000
5000
0
8
10
12
14
16
18
20
22
24
Electron kinetic energy (eV)
79.054
91.055
93.070
105.070
143.027
144.035
147.117
252.129
255.176
291.154
308.156
309.165

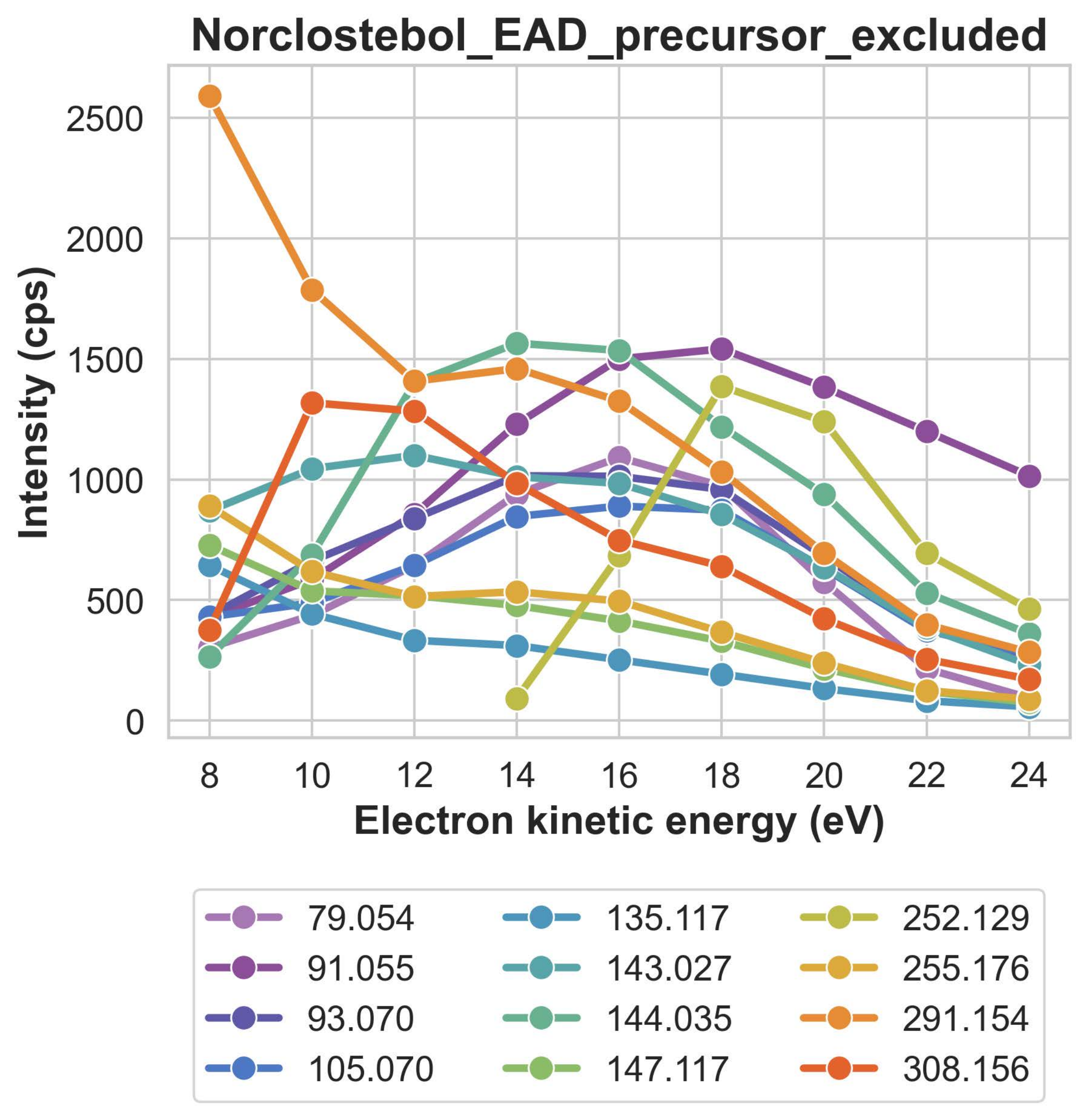

Norclostebol_EAD_precursor_excluded
Intensity (cps)
0
500
1000
1500
2000
2500
8
10
12
14
16
18
20
22
24
Electron kinetic energy (eV)
79.054
91.055
93.070
105.070
135.117
143.027
144.035
147.117
252.129
255.176
291.154
308.156

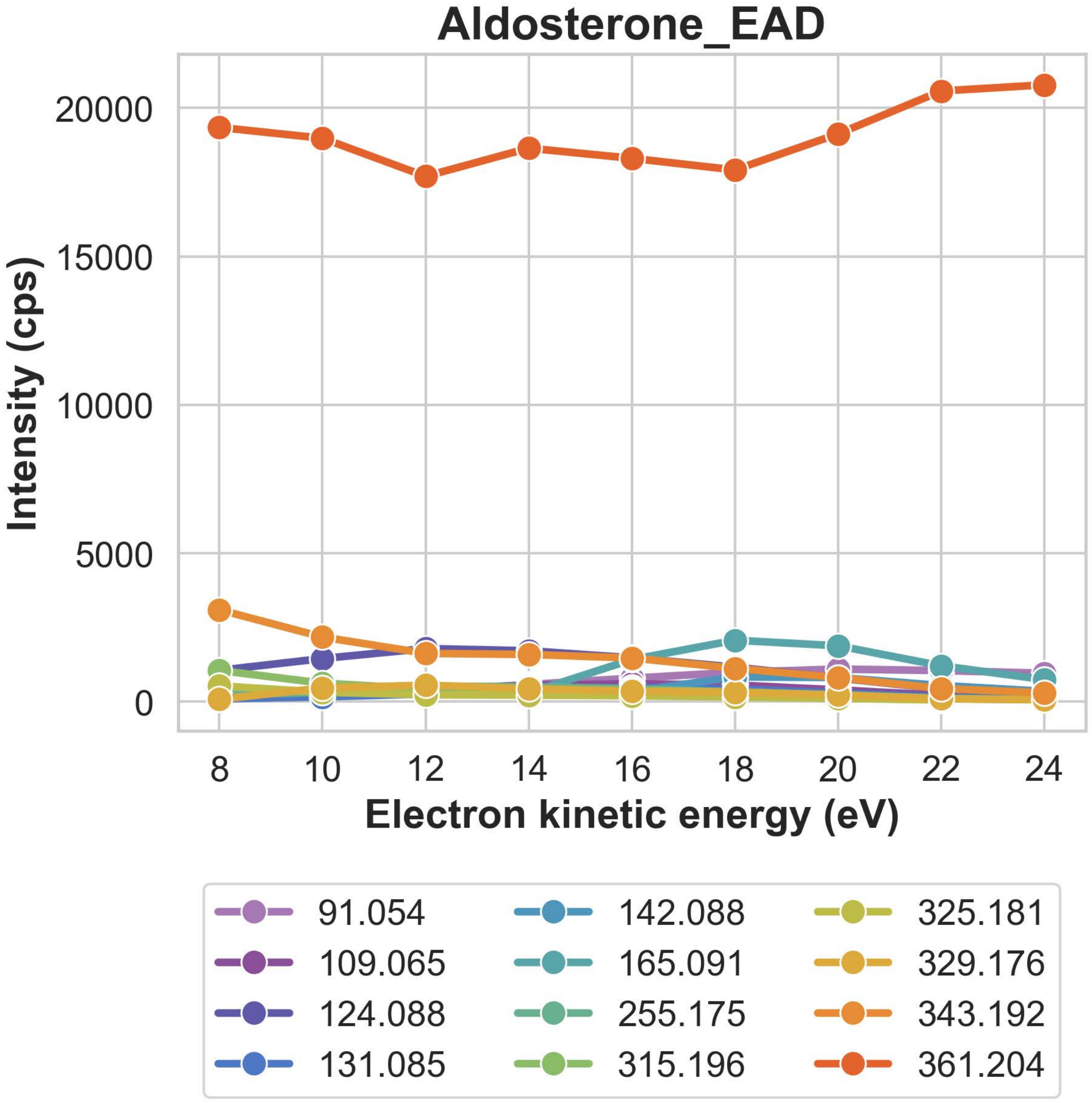
Aldosterone_EAD
Intensity (cps)
20000
15000
10000
5000
0
8
10
12
14
16
18
20
22
24
Electron kinetic energy (eV)
91.054
109.065
124.088
131.085
142.088
165.091
255.175
315.196
325.181
329.176
343.192
361.204

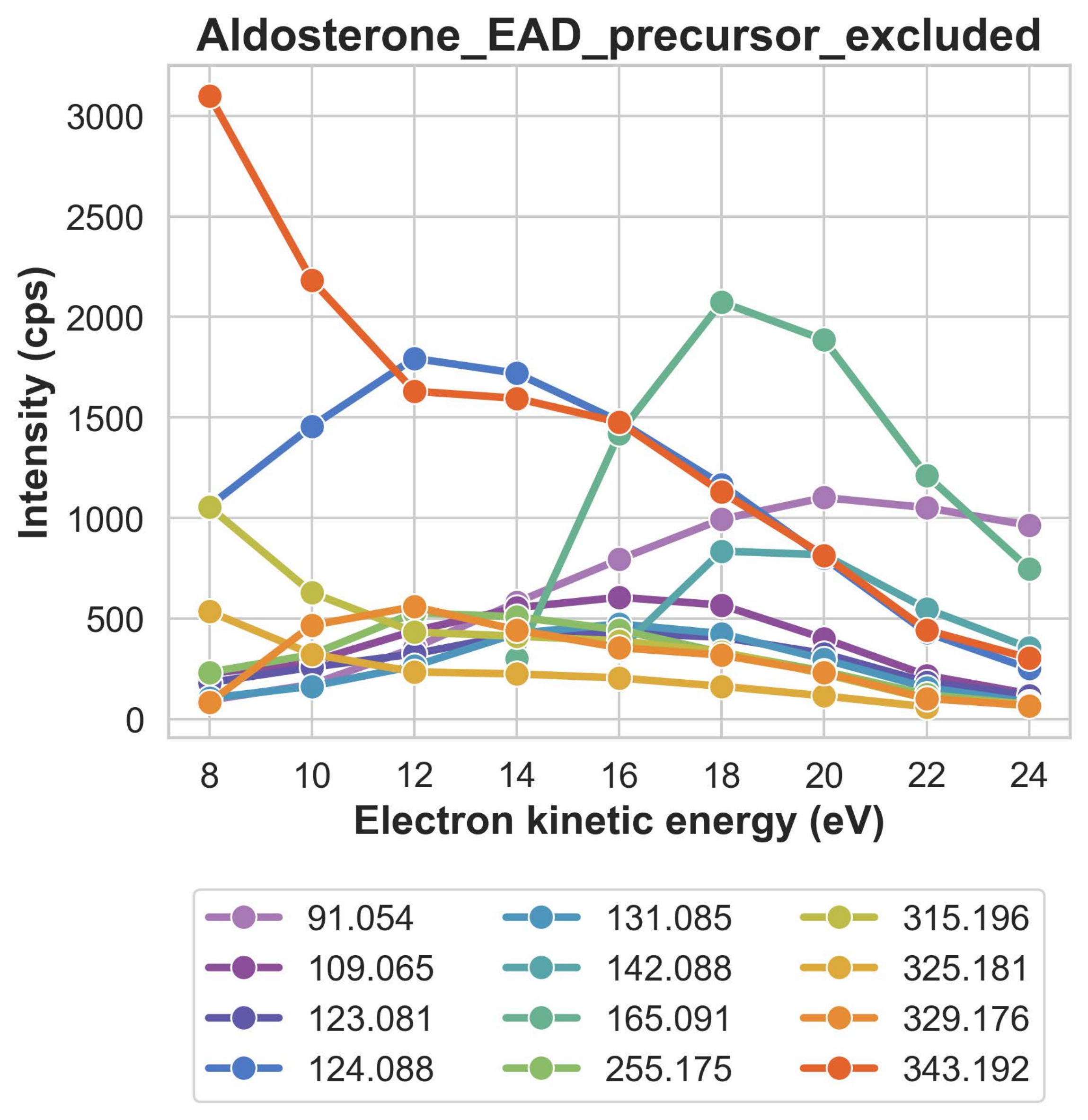
Aldosterone_EAD_precursor_excluded
Intensity (cps)
3000
2500
2000
1500
1000
500
0
8
10
12
14
16
18
20
22
24
Electron kinetic energy (eV)
91.054
109.065
123.081
124.088
131.085
142.088
165.091
255.175
315.196
325.181
329.176
343.192

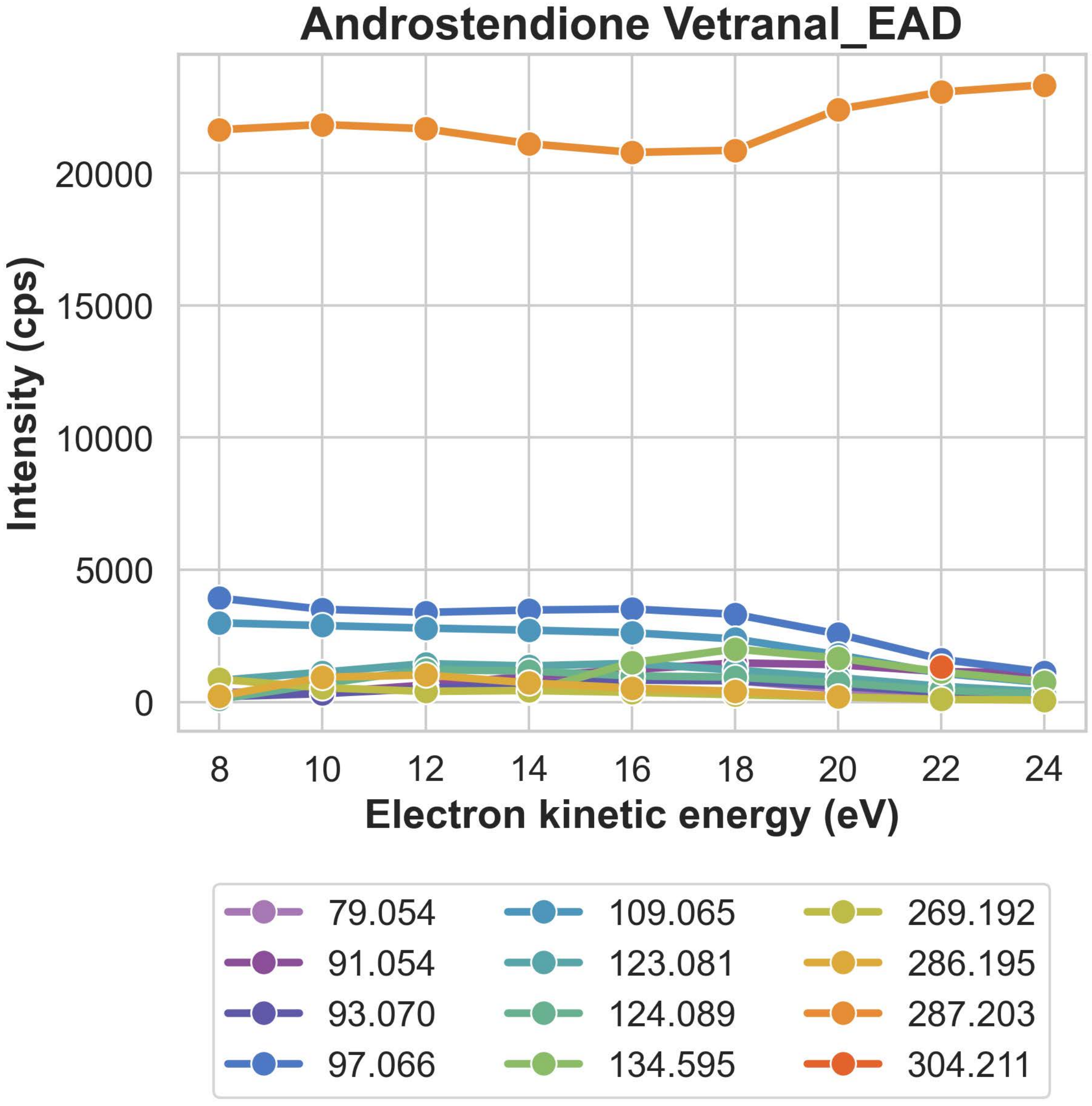
Androstendione Vetranal_EAD
Intensity (cps)
20000
15000
10000
5000
0
8
10
12
14
16
18
20
22
24
Electron kinetic energy (eV)
79.054
91.054
93.070
97.066
109.065
123.081
124.089
134.595
269.192
286.195
287.203
304.211

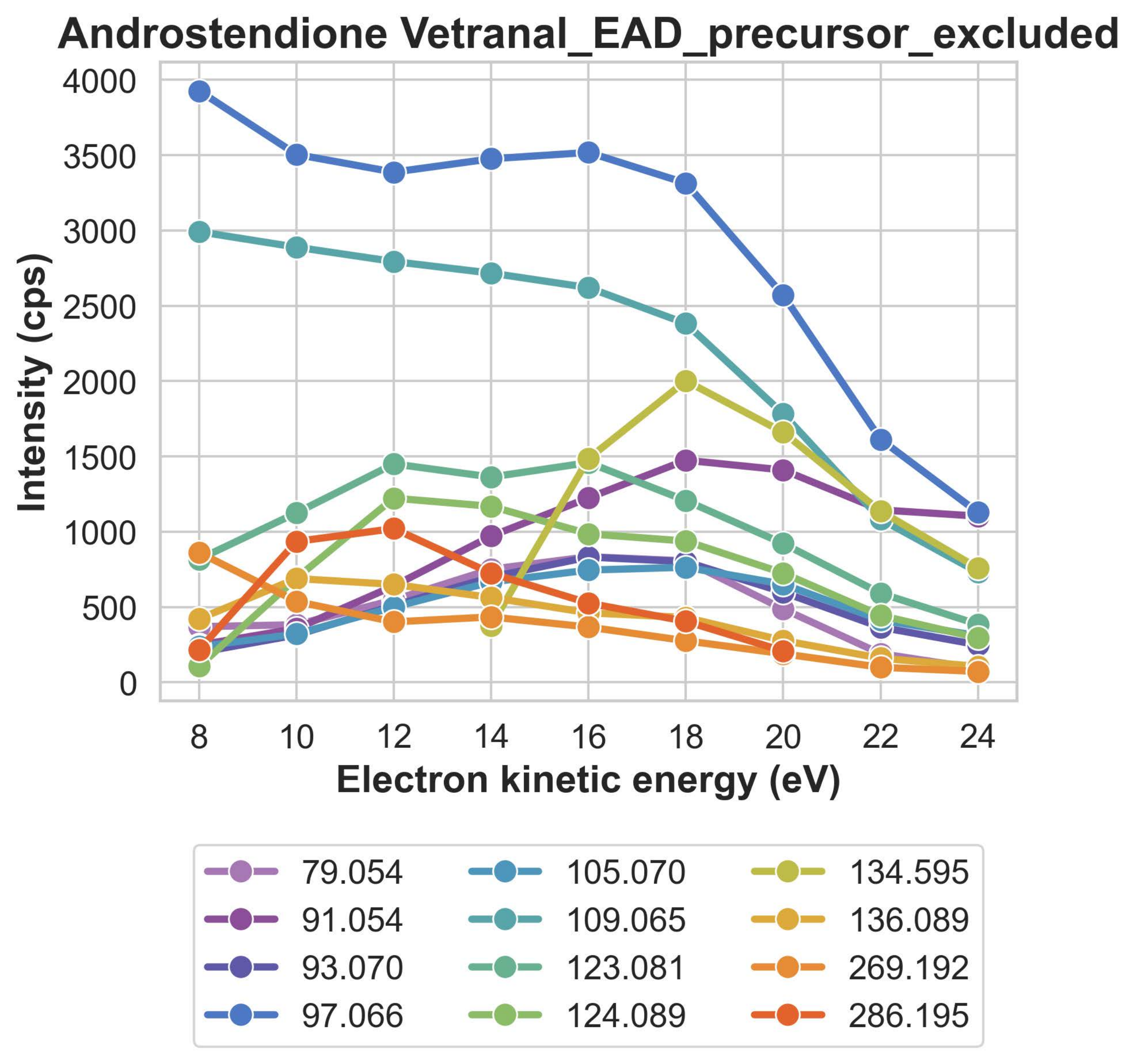
Androstendione Vetranal_EAD_precursor_excluded
Intensity (cps)
4000
3500
3000
2500
2000
1500
1000
500
0
8
10
12
14
16
18
20
22
24
Electron kinetic energy (eV)
79.054
91.054
93.070
97.066
105.070
109.065
123.081
124.089
134.595
136.089
269.192
286.195

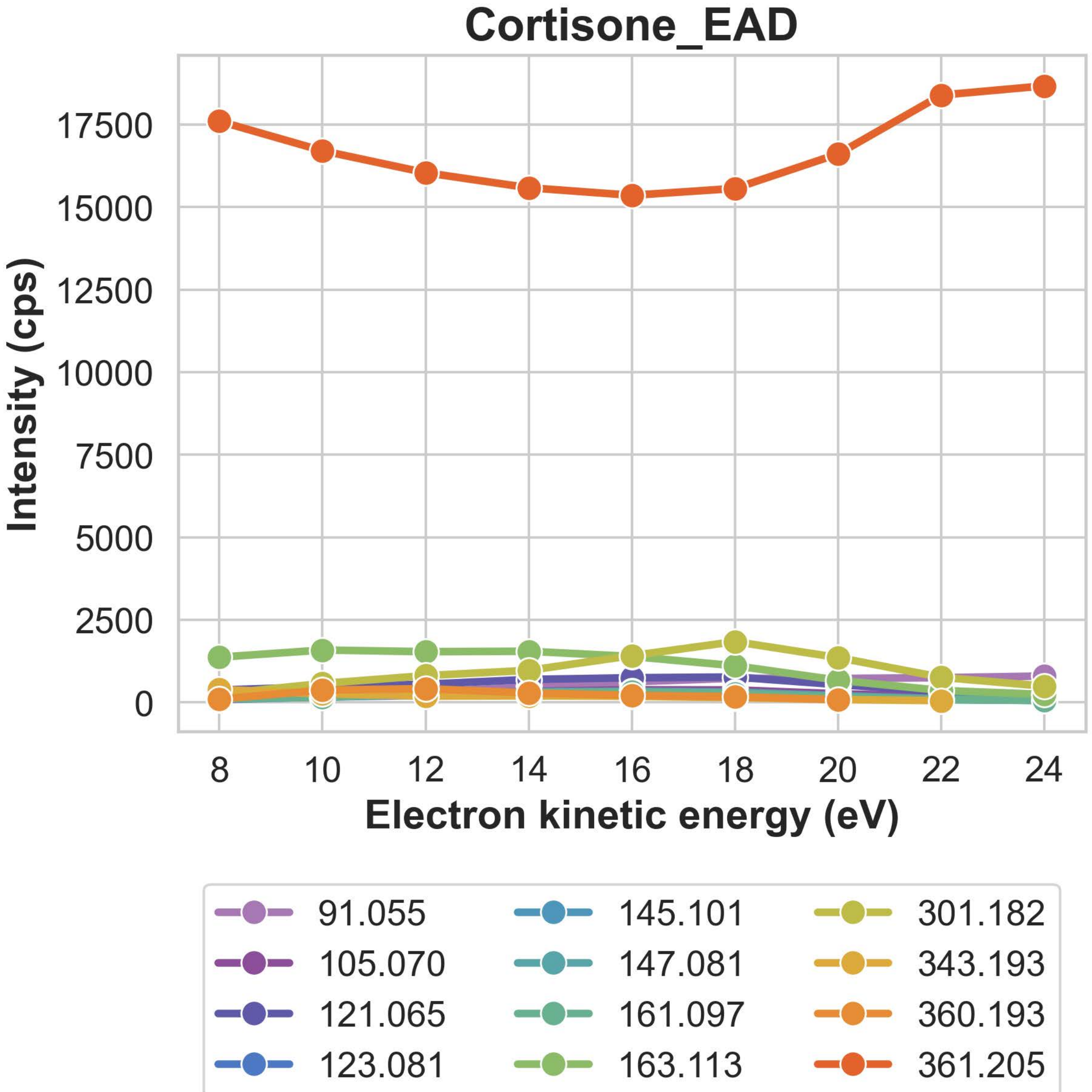
Cortisone_EAD
Intensity (cps)
17500
15000
12500
10000
7500
5000
2500
0
8
10
12
14
16
18
20
22
24
Electron kinetic energy (eV)
91.055
105.070
121.065
123.081
145.101
147.081
161.097
163.113
301.182
343.193
360.193
361.205

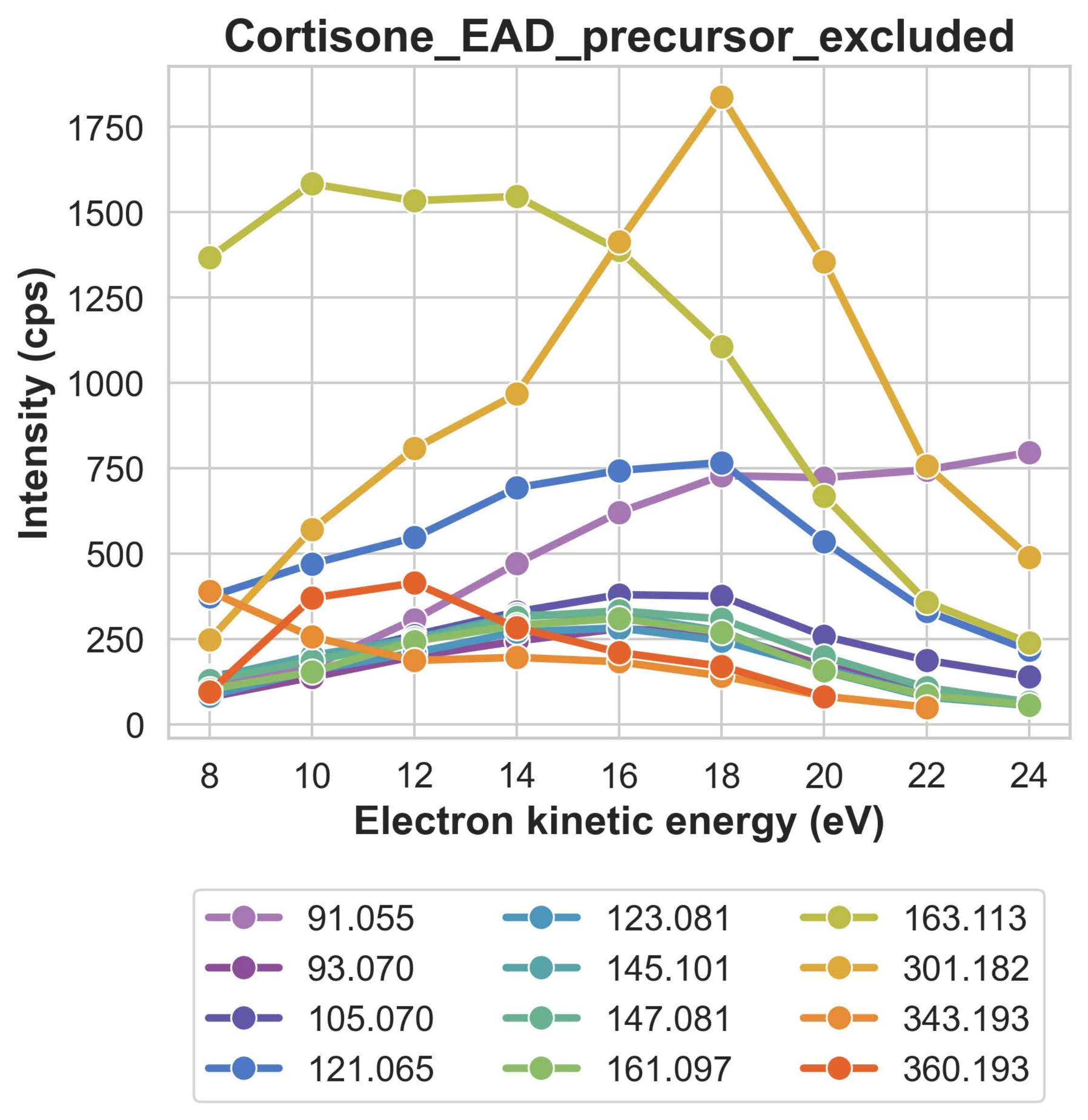

Cortisone_EAD_precursor_excluded
Intensity (cps)
0
250
500
750
1000
1250
1500
1750
8
10
12
14
16
18
20
22
24
Electron kinetic energy (eV)
91.055
93.070
105.070
121.065
123.081
145.101
147.081
161.097
163.113
301.182
343.193
360.193

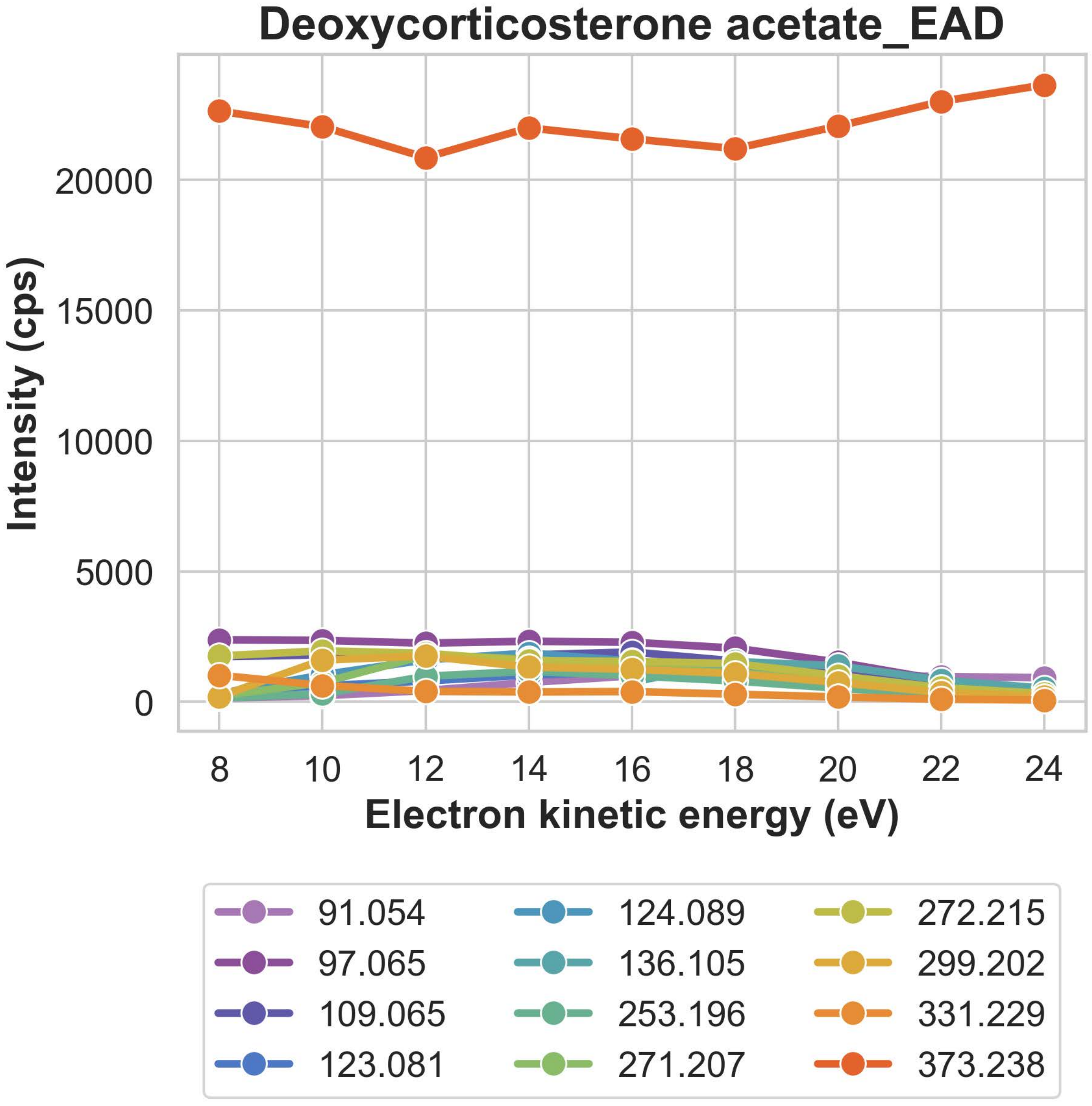
Deoxycorticosterone acetate_EAD
Intensity (cps)
0
5000
10000
15000
20000
8
10
12
14
16
18
20
22
24
Electron kinetic energy (eV)
91.054
97.065
109.065
123.081
124.089
136.105
253.196
271.207
272.215
299.202
331.229
373.238

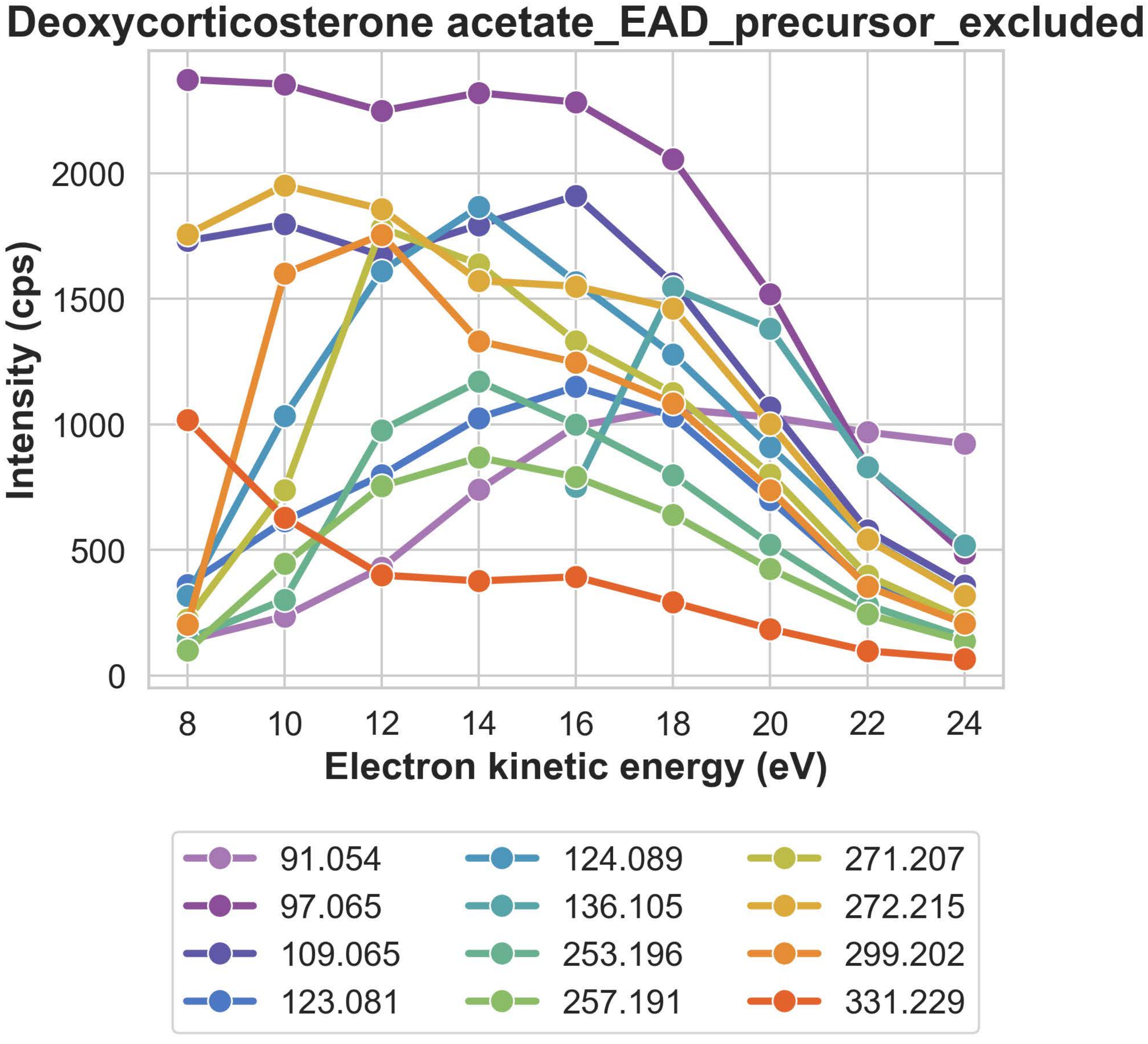
Deoxycorticosterone acetate_EAD_precursor_excluded
Intensity (cps)
0
500
1000
1500
2000
8
10
12
14
16
18
20
22
24
Electron kinetic energy (eV)
91.054
97.065
109.065
123.081
124.089
136.105
253.196
257.191
271.207
272.215
299.202
331.229

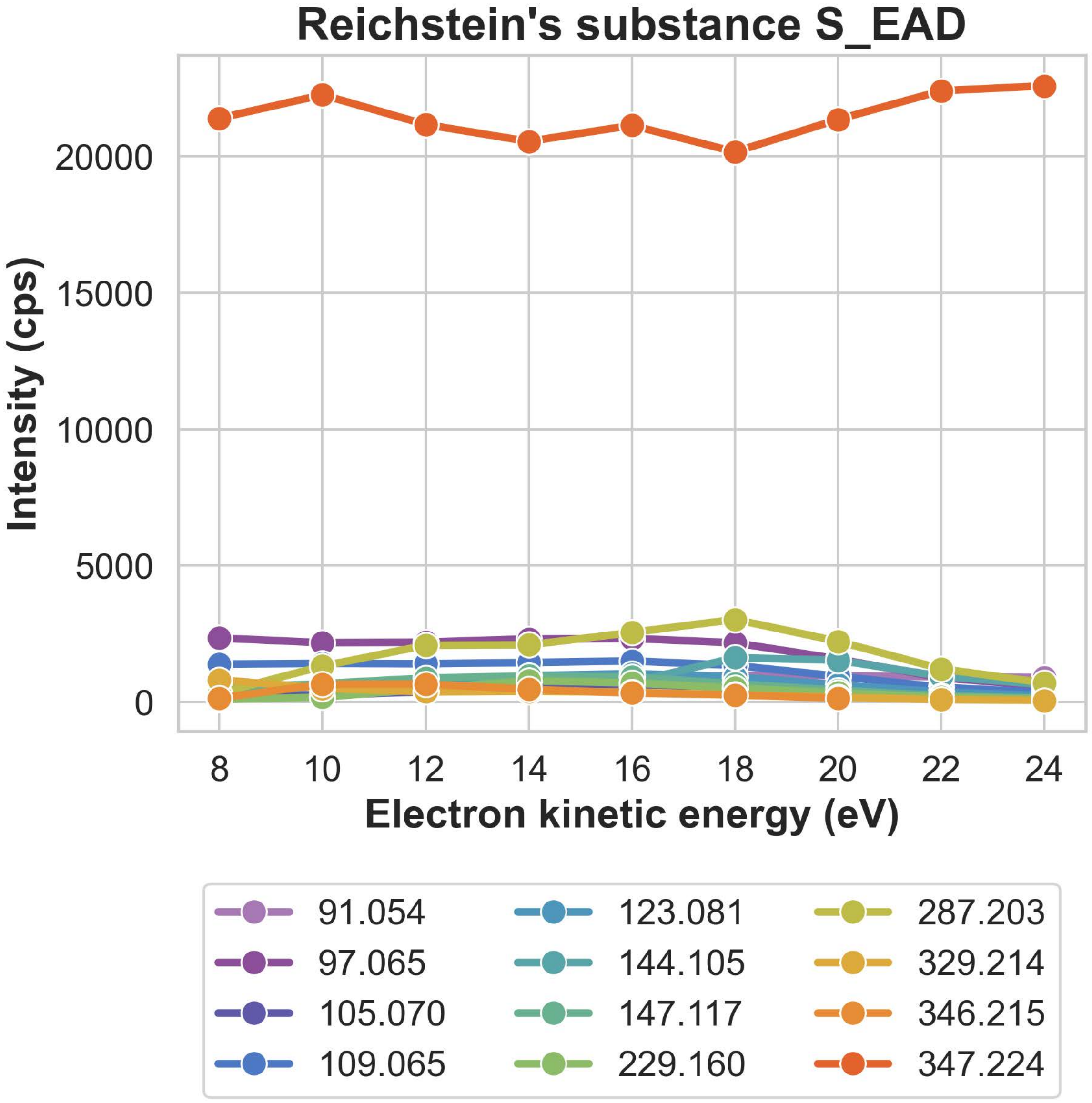
Reichstein's substance S_EAD
Intensity (cps)
20000
15000
10000
5000
0
8
10
12
14
16
18
20
22
24
Electron kinetic energy (eV)
91.054
97.065
105.070
109.065
123.081
144.105
147.117
229.160
287.203
329.214
346.215
347.224

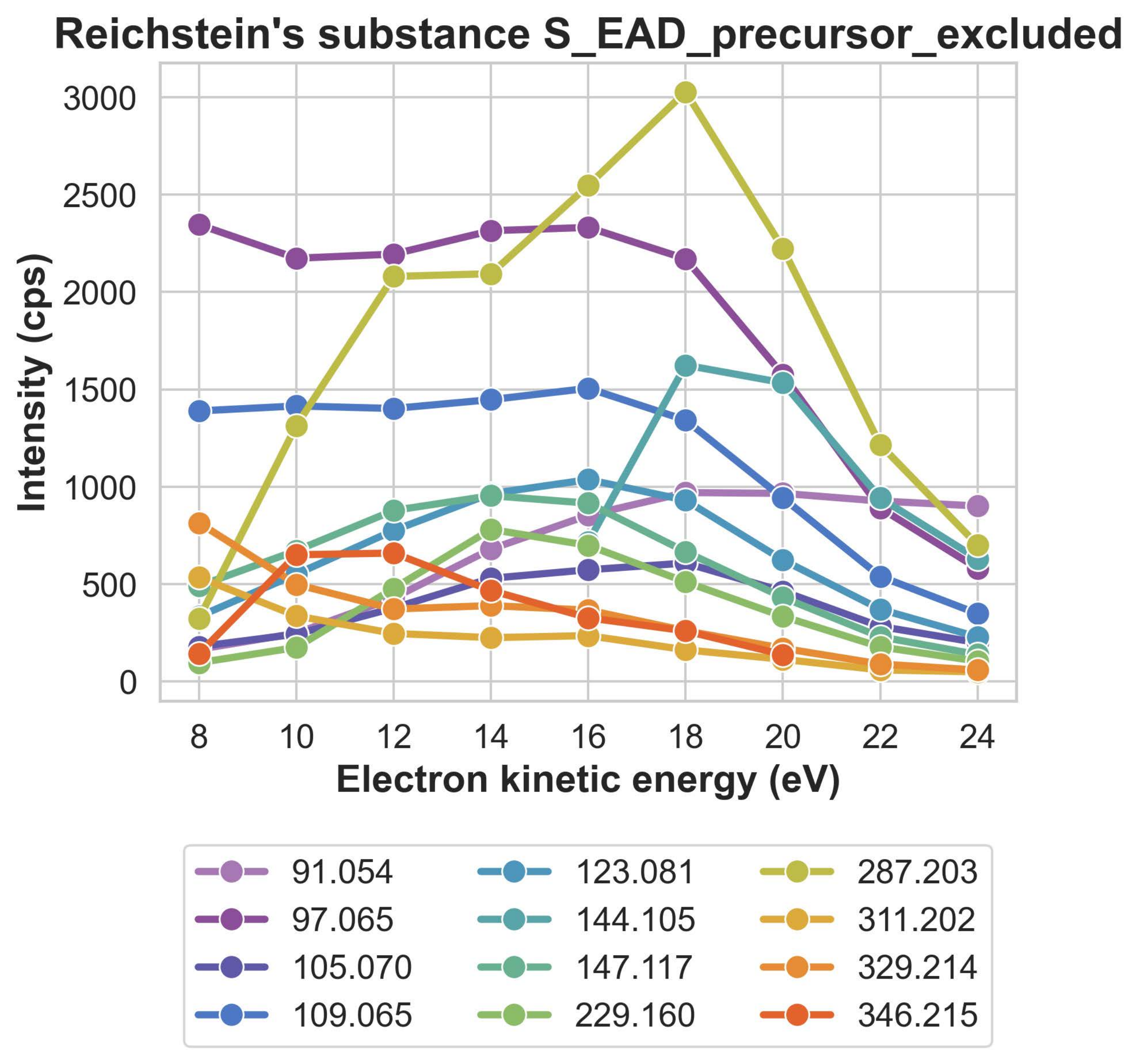
Reichstein's substance S_EAD_precursor_excluded
Intensity (cps)
3000
2500
2000
1500
1000
500
0
8
10
12
14
16
18
20
22
24
Electron kinetic energy (eV)
91.054
97.065
105.070
109.065
123.081
144.105
147.117
229.160
287.203
311.202
329.214
346.215

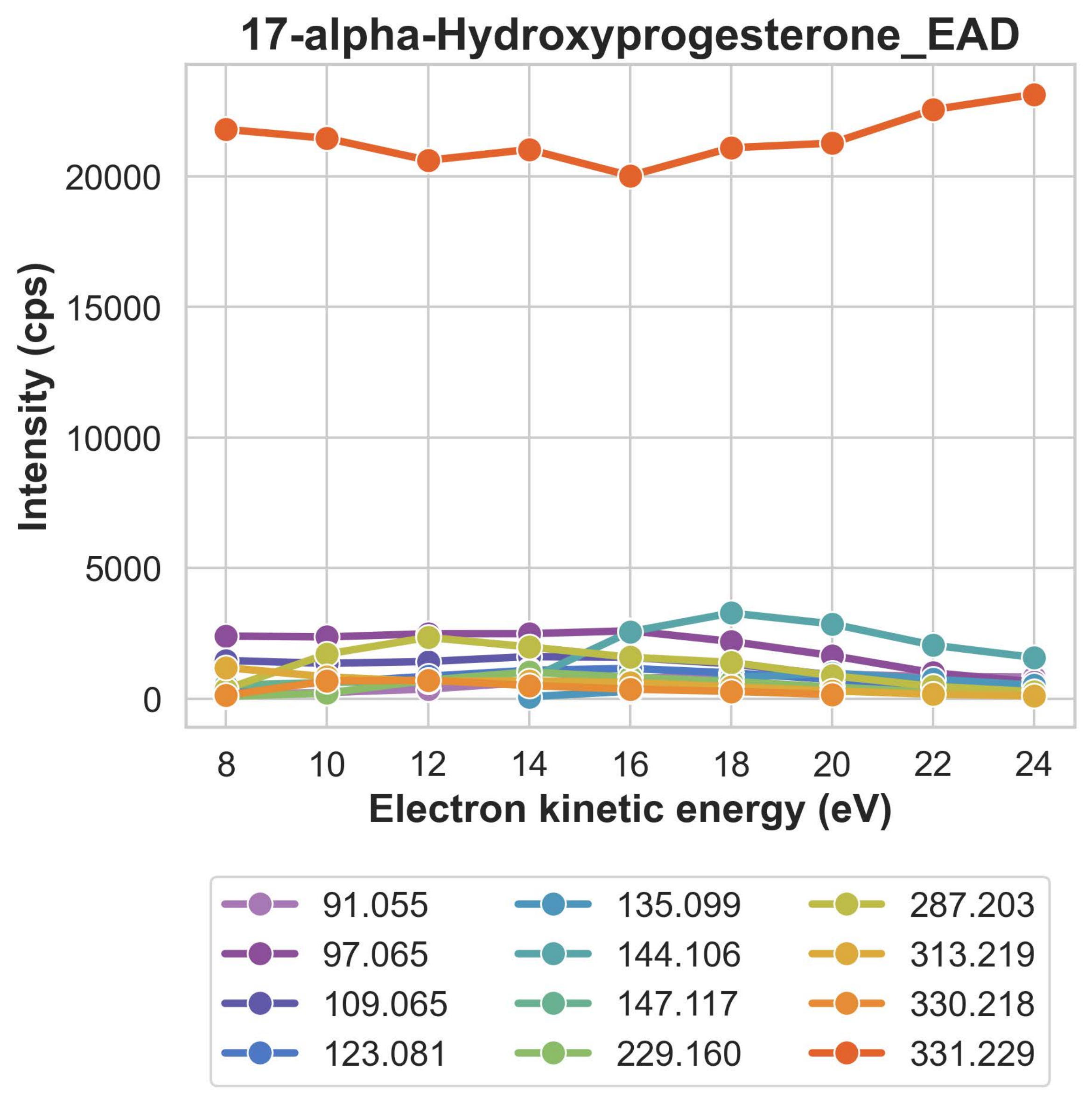
17-alpha-Hydroxyprogesterone_EAD
Intensity (cps)
20000
15000
10000
5000
0
8
10
12
14
16
18
20
22
24
Electron kinetic energy (eV)
91.055
97.065
109.065
123.081
135.099
144.106
147.117
229.160
287.203
313.219
330.218
331.229

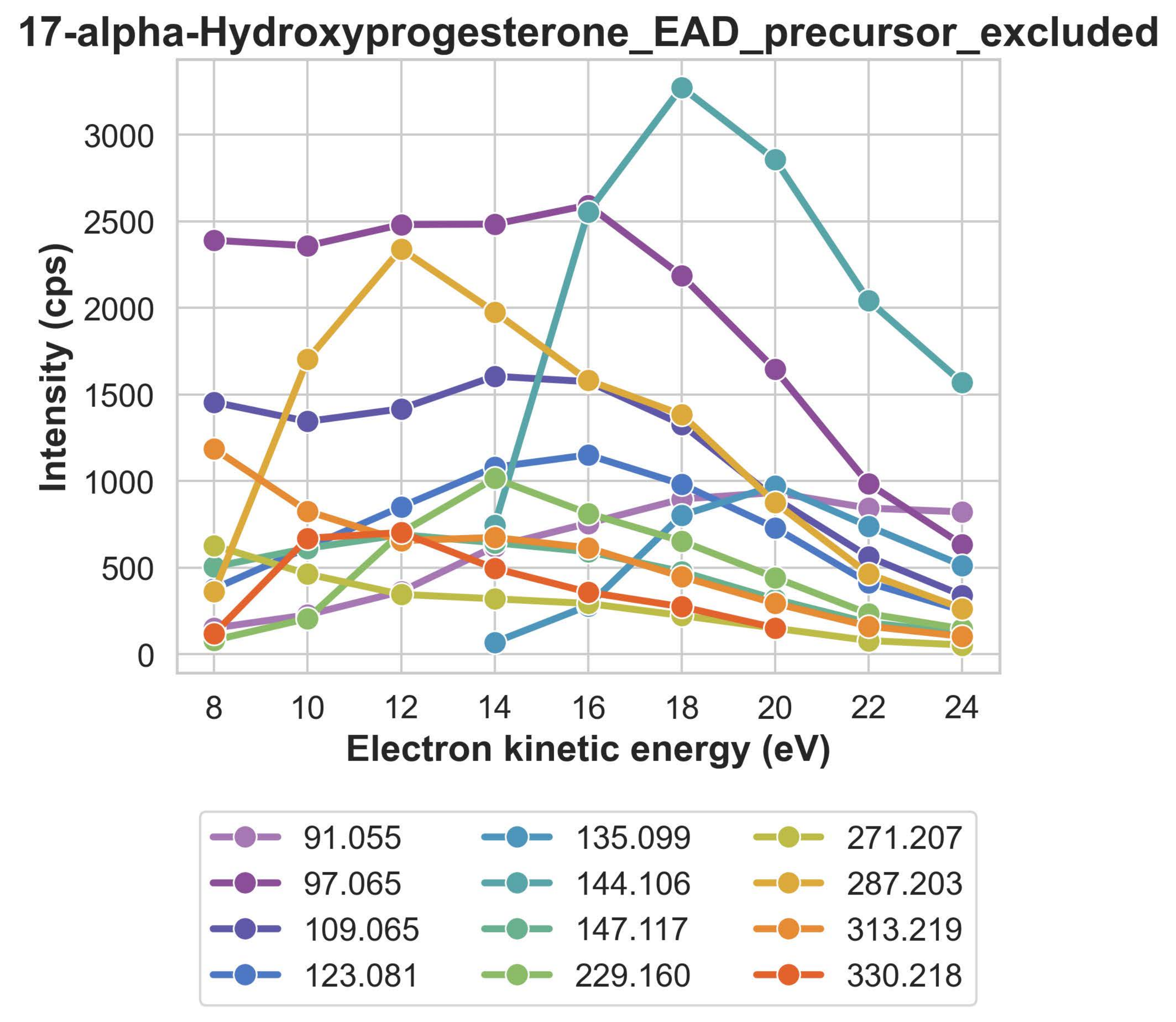
17-alpha-Hydroxyprogesterone_EAD_precursor_excluded
Intensity (cps)
0
500
1000
1500
2000
2500
3000
8
10
12
14
16
18
20
22
24
Electron kinetic energy (eV)
91.055
97.065
109.065
123.081
135.099
144.106
147.117
229.160
271.207
287.203
313.219
330.218

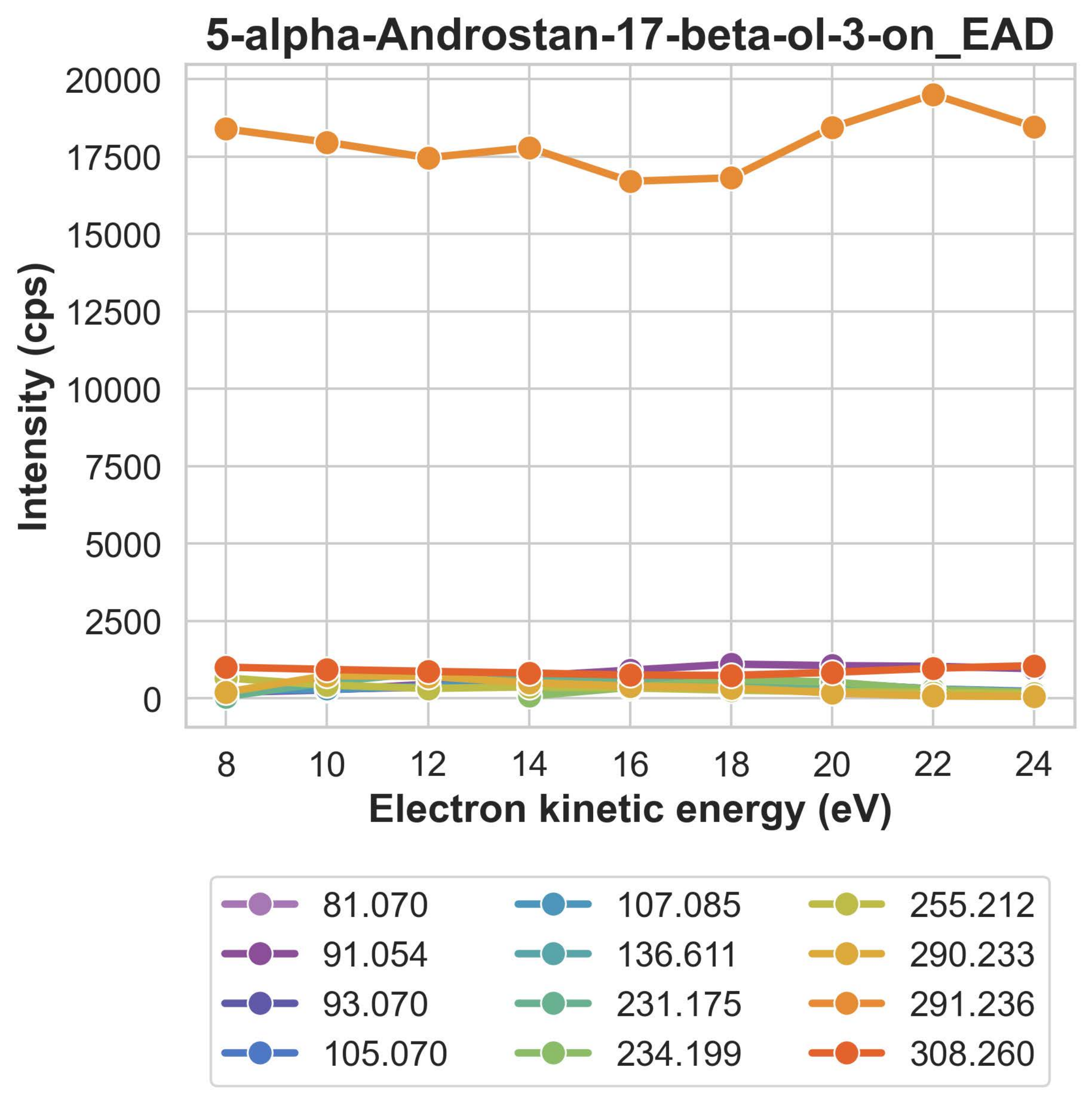
5-alpha-Androstan-17-beta-ol-3-on_EAD
Intensity (cps)
20000
17500
15000
12500
10000
7500
5000
2500
0
8
10
12
14
16
18
20
22
24
Electron kinetic energy (eV)
81.070
91.054
93.070
105.070
107.085
136.611
231.175
234.199
255.212
290.233
291.236
308.260

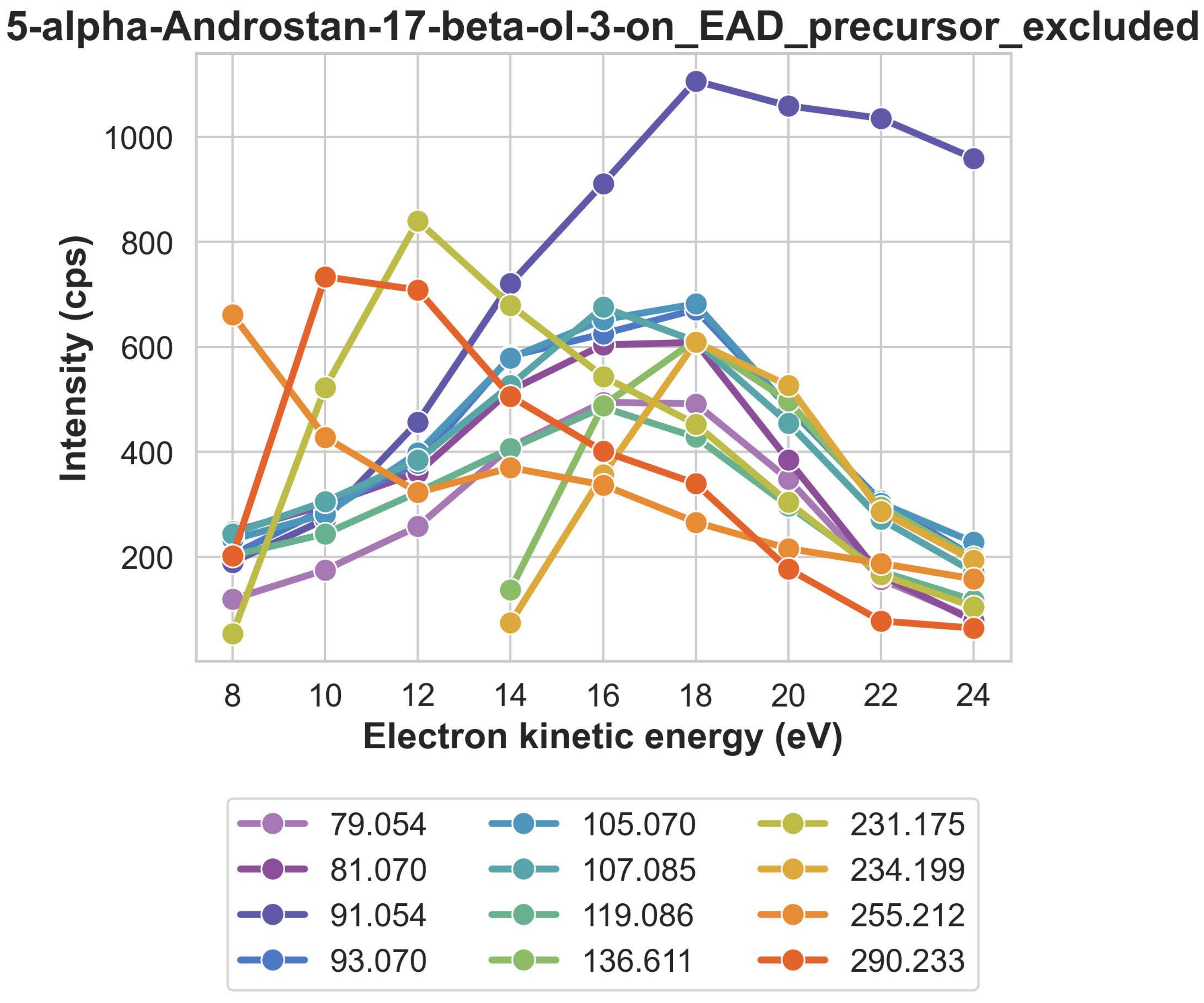
5-alpha-Androstan-17-beta-ol-3-on_EAD_precursor_excluded
Intensity (cps)
1000
800
600
400
200
8
10
12
14
16
18
20
22
24
Electron kinetic energy (eV)
79.054
81.070
91.054
93.070
105.070
107.085
119.086
136.611
231.175
234.199
255.212
290.233

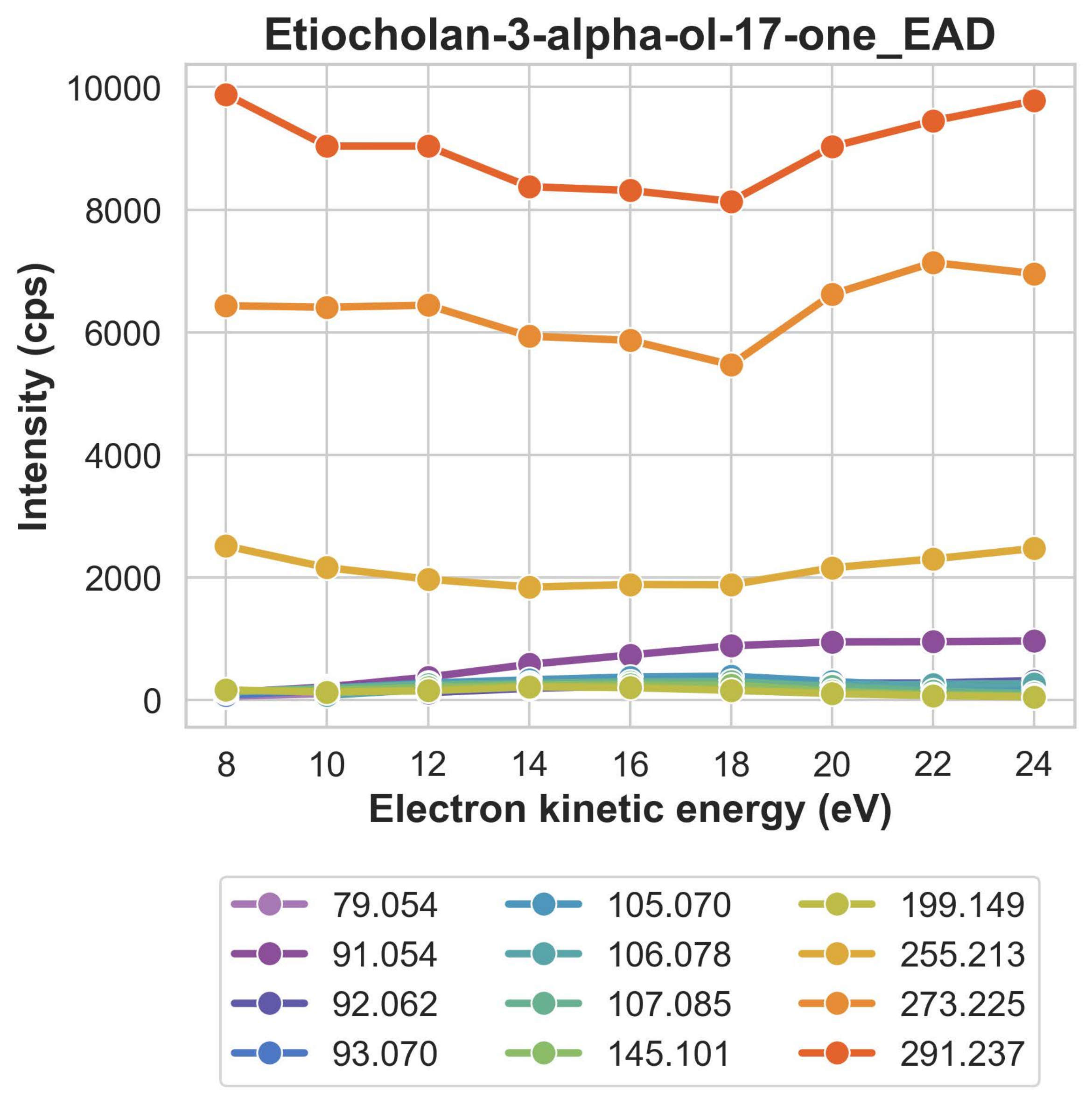
Etiocholan-3-alpha-ol-17-one_EAD
10000
8000
6000
4000
2000
0
Intensity (cps)
8
10
12
14
16
18
20
22
24
Electron kinetic energy (eV)
79.054
91.054
92.062
93.070
105.070
106.078
107.085
145.101
199.149
255.213
273.225
291.237

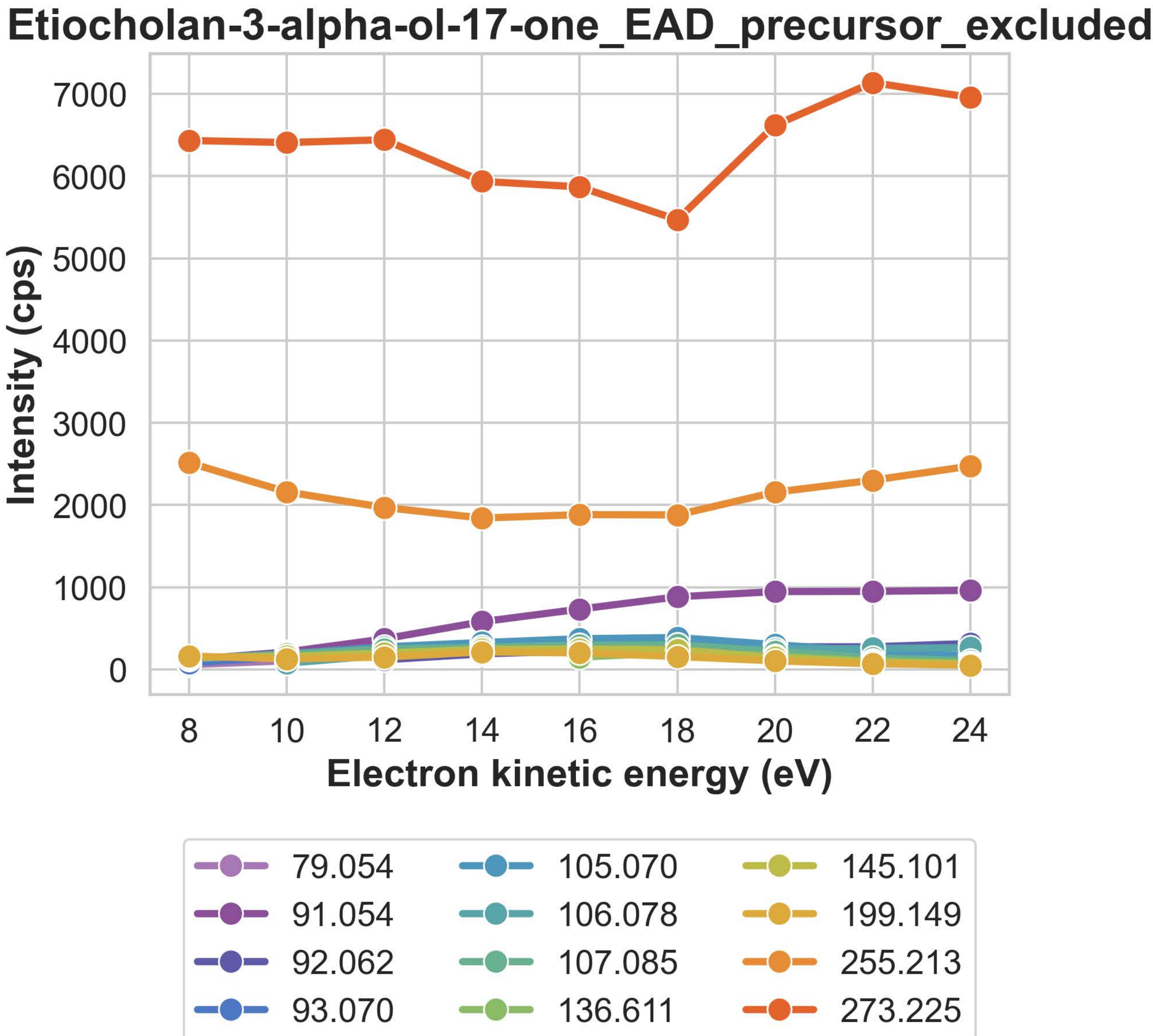

Etiocholan-3-alpha-ol-17-one_EAD_precursor_excluded
Intensity (cps)
0
1000
2000
3000
4000
5000
6000
7000
8
10
12
14
16
18
20
22
24
Electron kinetic energy (eV)
79.054
91.054
92.062
93.070
105.070
106.078
107.085
136.611
145.101
199.149
255.213
273.225
