# Supplementary material for: From Single Injection to Dual Insights: Integrating EAD and CID Acquisition for Spectral Library Generation and EAD–CID Spectral Matching
Source: Anal Chem. 2026 Oct 2;98(41):29433–44. doi: 10.1021/acs.analchem.6c01204 (PMC13689410; doi:10.1021/acs.analchem.6c01204)
Supplement: Supplementary file 2 [file ac6c01204_si_002.pdf]

Supporting Information- Threshold evaluation

# From Single Injection to Dual Insights: Integrating EAD and CID acquisition for Spectral Library Generation and EAD–CID Spectral Matching

Lisa Panzenboeck[1,2], Christina Brenner[1,2], Veronika Fitz[1,2], Mingxun Wang[3], Patrick Mueller[1], Evelyn Rampler[1,2], Yasin El Abiead[4]* and Gunda Koellensperger[1,5]*

1 Department of Analytical Chemistry, Faculty of Chemistry, University of Vienna, 1090 Vienna, Austria

2 Vienna Doctoral School in Chemistry (DoSChem), University of Vienna, 1090 Vienna, Austria

3 Department of Computer Science and Engineering, University of California Riverside, Riverside, California 92521, United States

4 BOKU University, Department of Chemistry, Institute of Analytical Chemistry, Muthgasse 18, 1190, Vienna, Austria

5 Vienna Metabolomics Center (VIME), University of Vienna, 1090, Vienna, Austria

* Corresponding authors:

Gunda Koellensperger: gunda.koellensperger@univie.ac.at, +43-1-0277-52303

Yasin El Abiead: yasin.el-abiead@boku.ac.at, +43-1-47654-77109

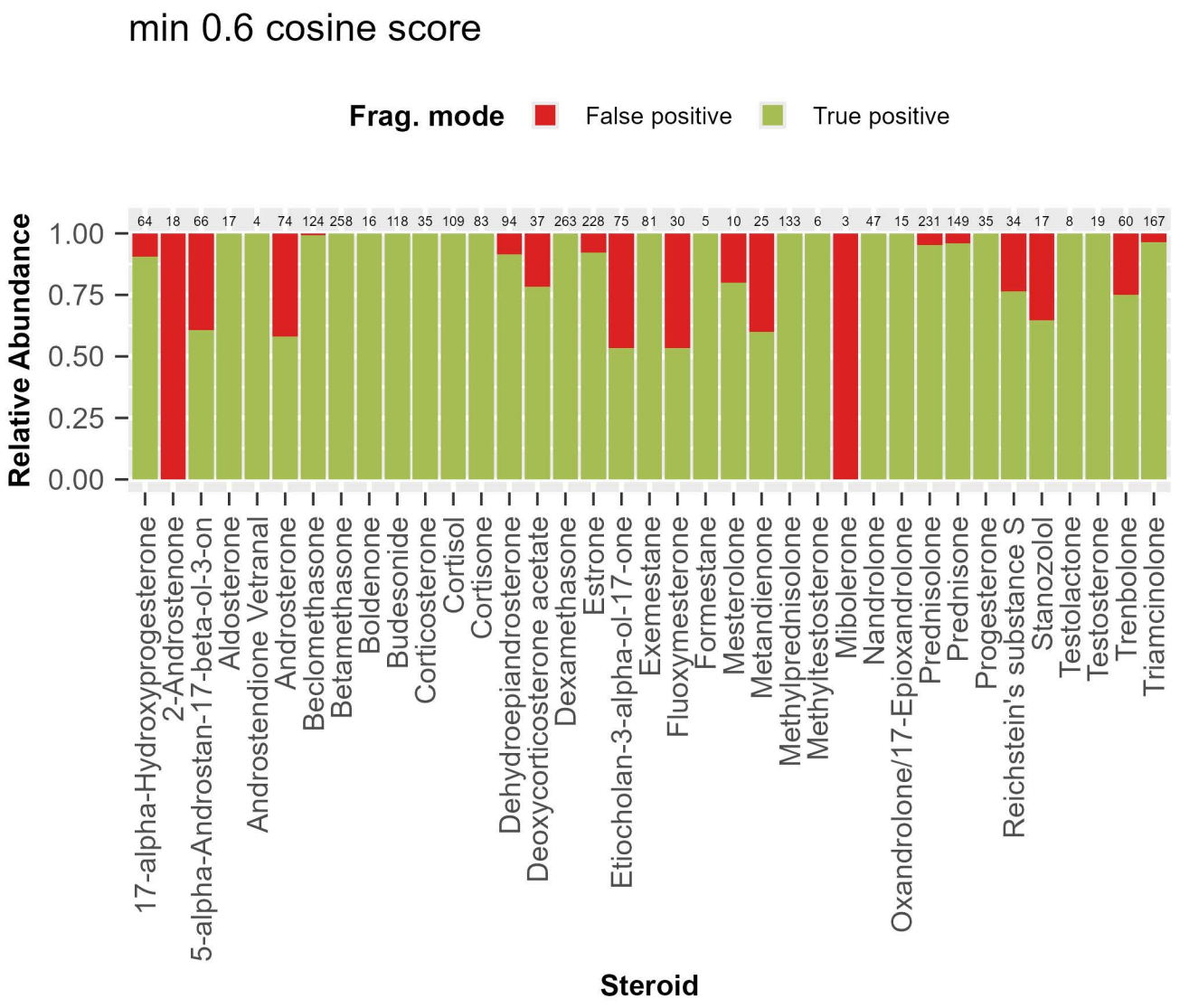
min 0.6 cosine score
Frag. mode
False positive
True positive
Relative Abundance
1.00
0.75
0.50
0.25
0.00
64 18 66 17 4 74 124 258 16 118 35 109 83 94 37 263 228 75 81 30 5 10 25 133 6 3 47 15 231 149 35 34 17 8 19 60 167
17-alpha-Hydroxyprogesterone
2-Androstenone
5-alpha-Androstan-17-beta-ol-3-on
Aldosterone
Androstendione Vetranal
Androsterone
Beclomethasone
Betamethasone
Boldenone
Budesonide
Corticosterone
Cortisol
Cortisone
Dehydroepiandrosterone
Deoxycorticosterone acetate
Dexamethasone
Estrone
Etiocholan-3-alpha-ol-17-one
Exemestane
Fluoxymesterone
Formestane
Mesterolone
Metandienone
Methylprednisolone
Methyltestosterone
Mibolerone
Nandrolone
Oxandrolone/17-Epioxandrolone
Prednisolone
Prednisone
Progesterone
Reichstein's substance S
Stanozolol
Testolactone
Testosterone
Trenbolone
Triamcinolone
Steroid

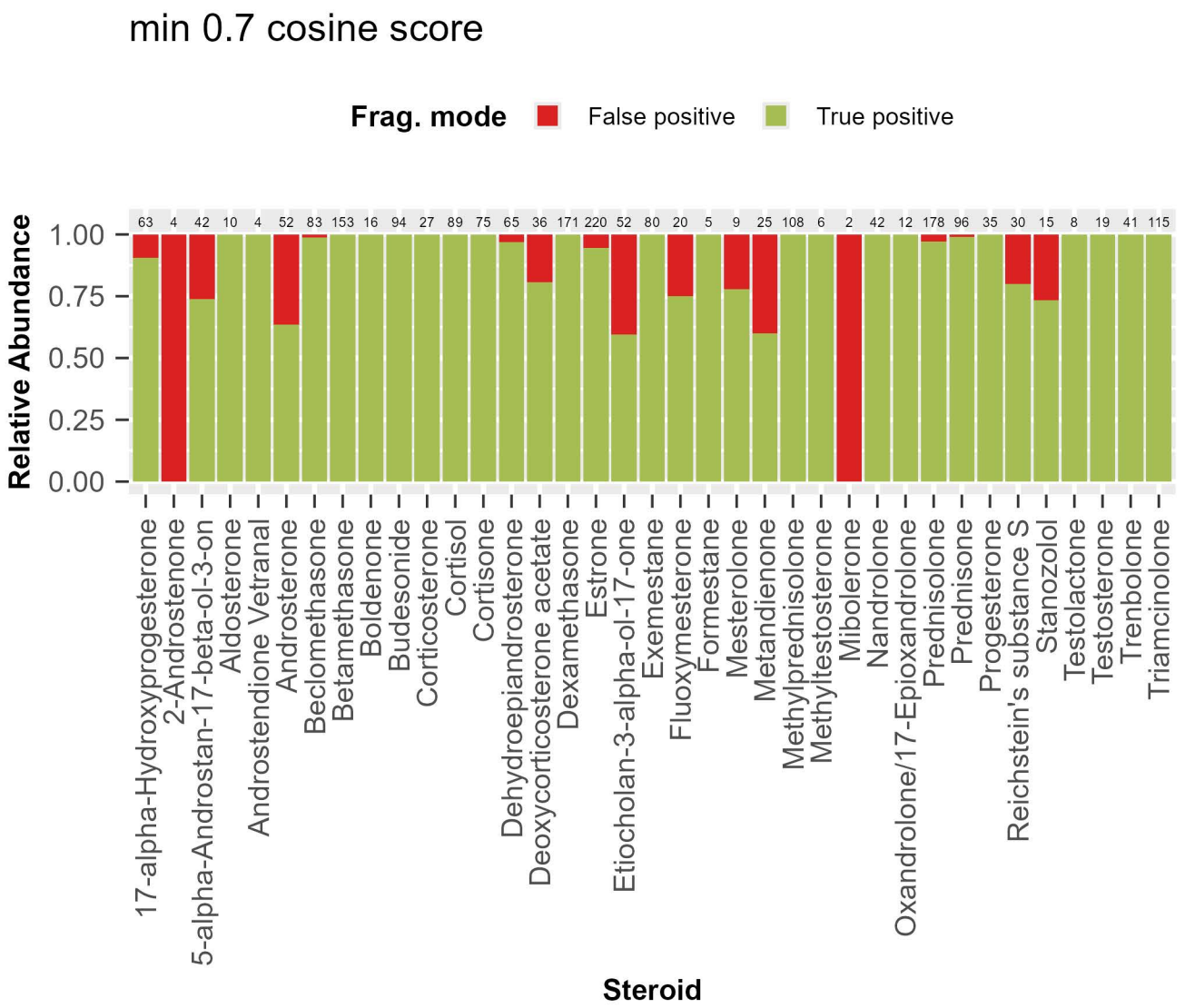
min 0.7 cosine score
Frag. mode
False positive
True positive
Relative Abundance
1.00
0.75
0.50
0.25
0.00
63 4 42 10 4 52 83 153 16 94 27 89 75 65 36 171 220 52 80 20 5 9 25 108 6 2 42 12 178 96 35 30 15 8 19 41 115
17-alpha-Hydroxyprogesterone
2-Androstenone
5-alpha-Androstan-17-beta-ol-3-on
Aldosterone
Androstendione Vetranal
Androsterone
Beclomethasone
Betamethasone
Boldenone
Budesonide
Corticosterone
Cortisol
Cortisone
Dehydroepiandrosterone
Deoxycorticosterone acetate
Dexamethasone
Estrone
Etiocholan-3-alpha-ol-17-one
Exemestane
Fluoxymesterone
Formestane
Mesterolone
Metandienone
Methylprednisolone
Methyltestosterone
Mibolerone
Nandrolone
Oxandrolone/17-Epioxandrolone
Prednisolone
Prednisone
Progesterone
Reichstein's substance S
Stanozolol
Testolactone
Testosterone
Trenbolone
Triamcinolone
Steroid

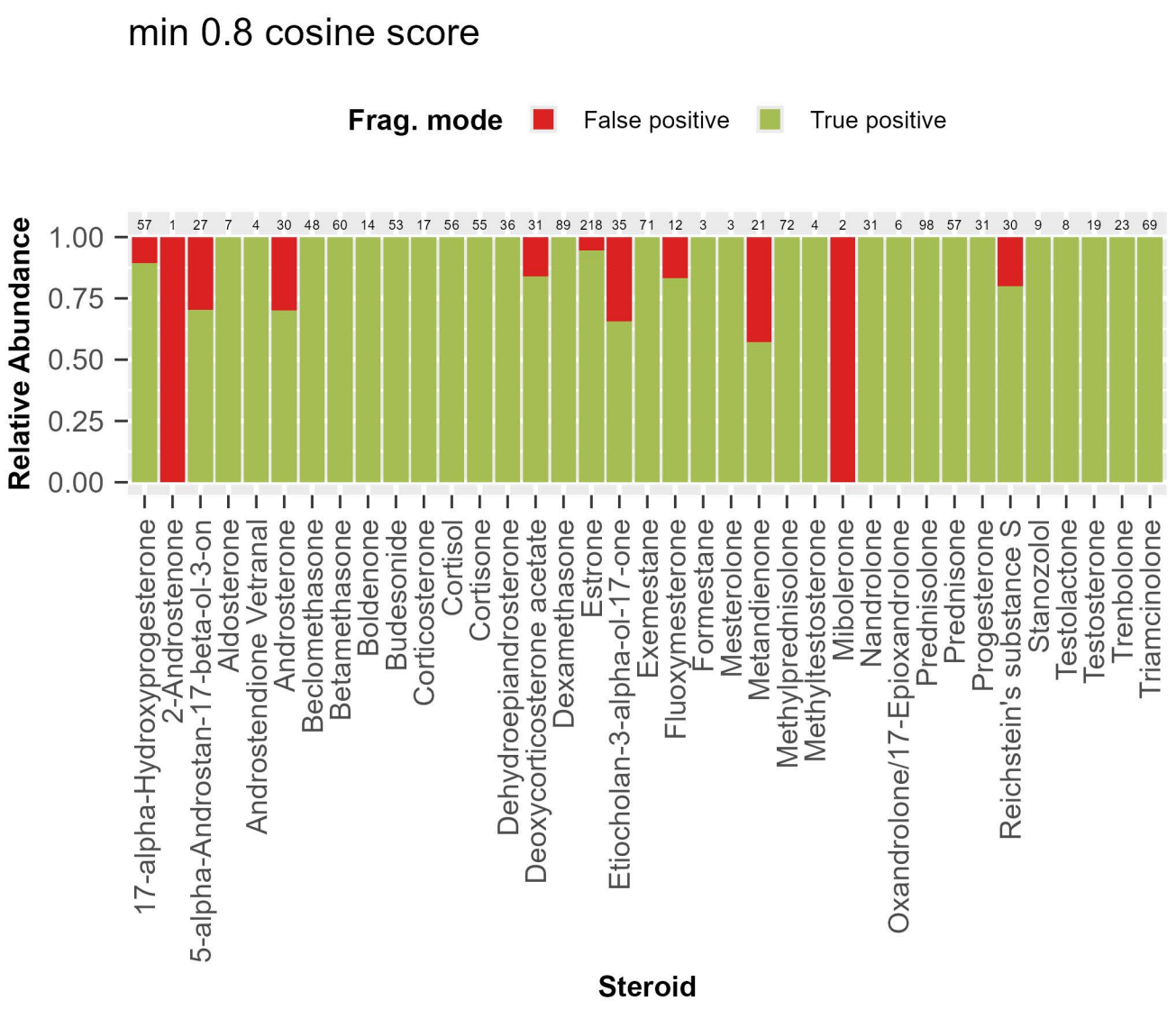
min 0.8 cosine score
Frag. mode
False positive
True positive
Relative Abundance
1.00
0.75
0.50
0.25
0.00
57 1 27 7 4 30 48 60 14 53 17 56 55 36 31 89 218 35 71 12 3 3 21 72 4 2 31 6 98 57 31 30 9 8 19 23 69
17-alpha-Hydroxyprogesterone
2-Androstenone
5-alpha-Androstan-17-beta-ol-3-on
Aldosterone
Androstendione Vetranal
Androsterone
Beclomethasone
Betamethasone
Boldenone
Budesonide
Corticosterone
Cortisol
Cortisone
Dehydroepiandrosterone
Deoxycorticosterone acetate
Dexamethasone
Estrone
Etiocholan-3-alpha-ol-17-one
Exemestane
Fluoxymesterone
Formestane
Mesterolone
Metandienone
Methylprednisolone
Methyltestosterone
Mibolerone
Nandrolone
Oxandrolone/17-Epioxandrolone
Prednisolone
Prednisone
Progesterone
Reichstein's substance S
Stanozolol
Testolactone
Testosterone
Trenbolone
Triamcinolone
Steroid

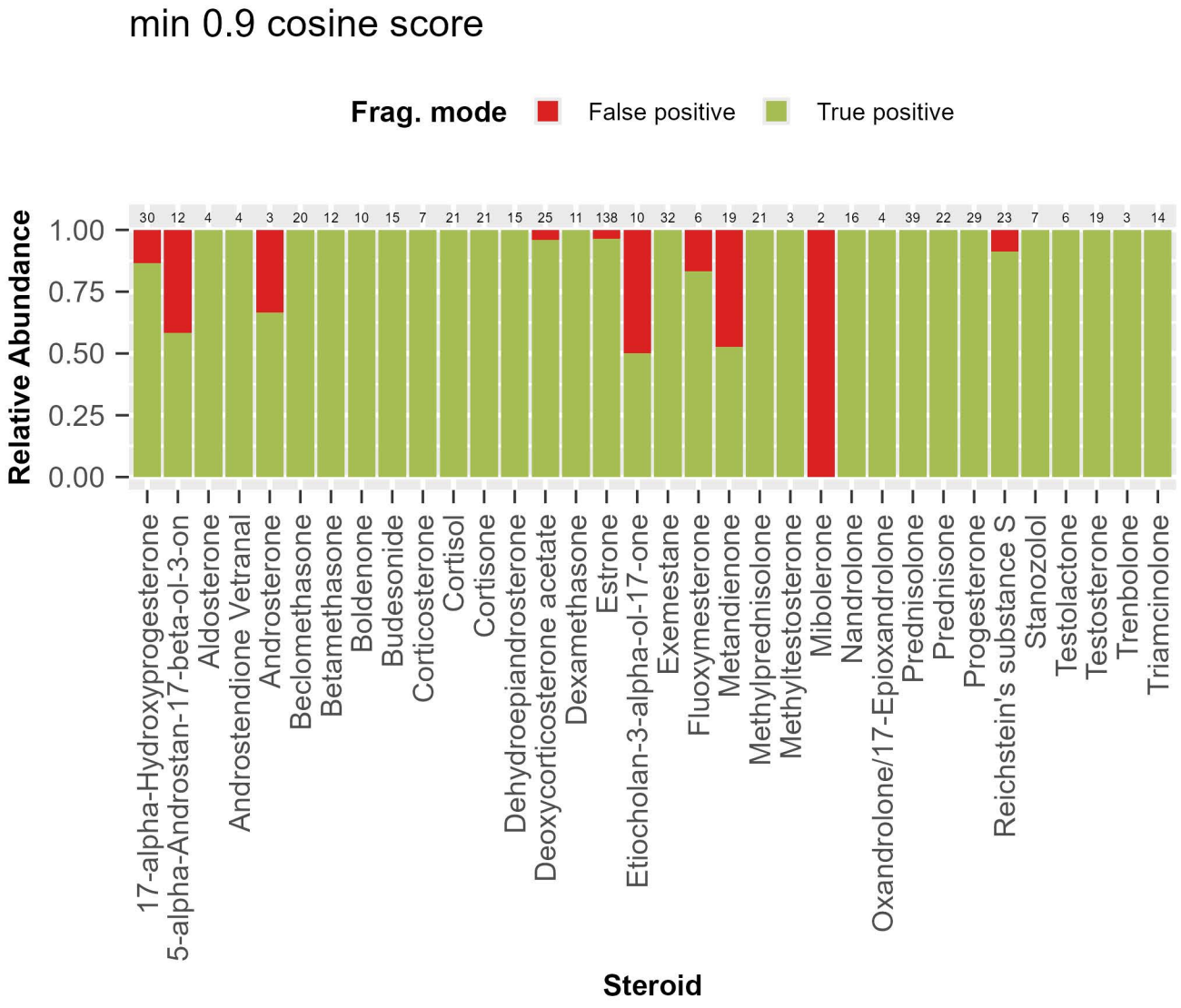

min 0.9 cosine score
Frag. mode
False positive
True positive
Relative Abundance
1.00
0.75
0.50
0.25
0.00
30 12 4 4 3 20 12 10 15 7 21 21 15 25 11 138 10 32 6 19 21 3 2 16 4 39 22 29 23 7 6 19 3 14
17-alpha-Hydroxyprogesterone
5-alpha-Androstan-17-beta-ol-3-on
Aldosterone
Androstendione Vetranal
Androsterone
Beclomethasone
Betamethasone
Boldenone
Budesonide
Corticosterone
Cortisol
Cortisone
Dehydroepiandrosterone
Deoxycorticosterone acetate
Dexamethasone
Estrone
Etiocholan-3-alpha-ol-17-one
Exemestane
Fluoxymesterone
Metandienone
Methylprednisolone
Methyltestosterone
Mibolerone
Nandrolone
Oxandrolone/17-Epioxandrolone
Prednisolone
Prednisone
Progesterone
Reichstein's substance S
Stanozolol
Testolactone
Testosterone
Trenbolone
Triamcinolone
Steroid

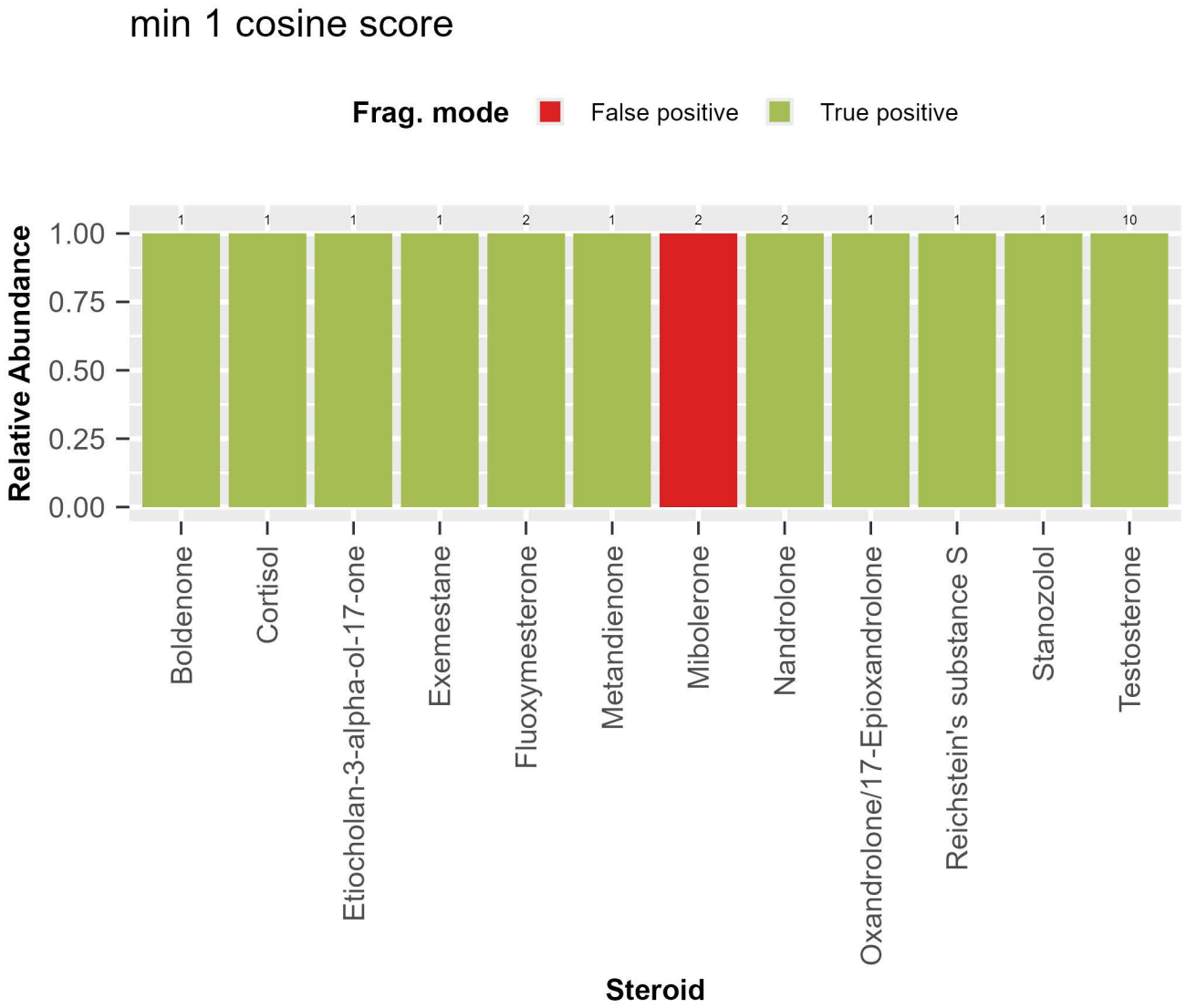

min 1 cosine score
Frag. mode
False positive
True positive
Relative Abundance
1.00
0.75
0.50
0.25
0.00
1
1
1
1
2
1
2
2
1
1
1
10
Boldenone
Cortisol
Etiocholan-3-alpha-ol-17-one
Exemestane
Fluoxymesterone
Metandienone
Mibolerone
Nandrolone
Oxandrolone/17-Epioxandrolone
Reichstein's substance S
Stanozolol
Testosterone
Steroid

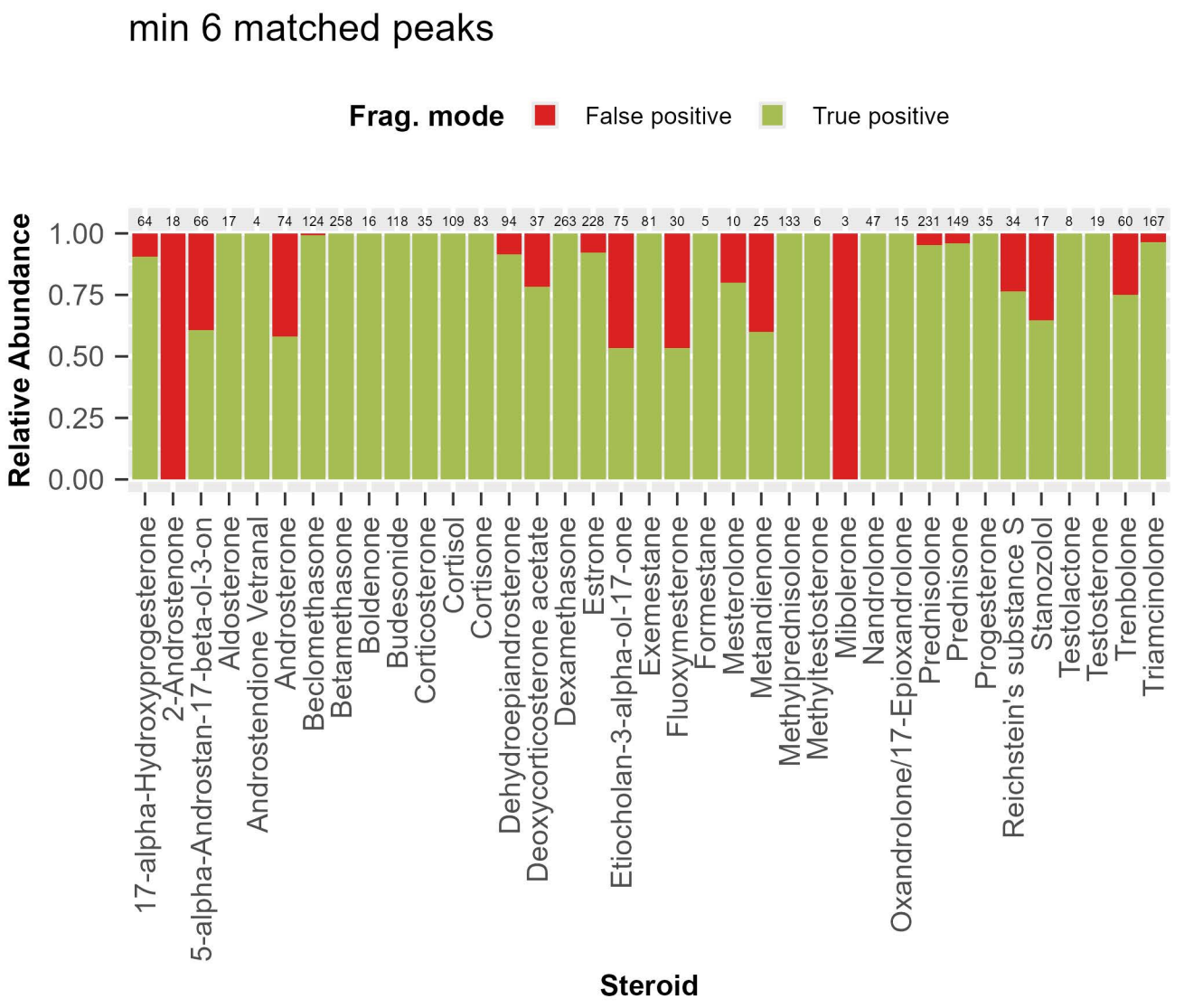
min 6 matched peaks
Frag. mode
False positive
True positive
Relative Abundance
1.00
0.75
0.50
0.25
0.00
64 18 66 17 4 74 124 258 16 118 35 109 83 94 37 263 228 75 81 30 5 10 25 133 6 3 47 15 231 149 35 34 17 8 19 60 167
17-alpha-Hydroxyprogesterone
2-Androstenone
5-alpha-Androstan-17-beta-ol-3-on
Aldosterone
Androstendione Vetranal
Androsterone
Beclomethasone
Betamethasone
Boldenone
Budesonide
Corticosterone
Cortisol
Cortisone
Dehydroepiandrosterone
Deoxycorticosterone acetate
Dexamethasone
Estrone
Etiocholan-3-alpha-ol-17-one
Exemestane
Fluoxymesterone
Formestane
Mesterolone
Metandienone
Methylprednisolone
Methyltestosterone
Mibolerone
Nandrolone
Oxandrolone/17-Epioxandrolone
Prednisolone
Prednisone
Progesterone
Reichstein's substance S
Stanozolol
Testolactone
Testosterone
Trenbolone
Triamcinolone
Steroid

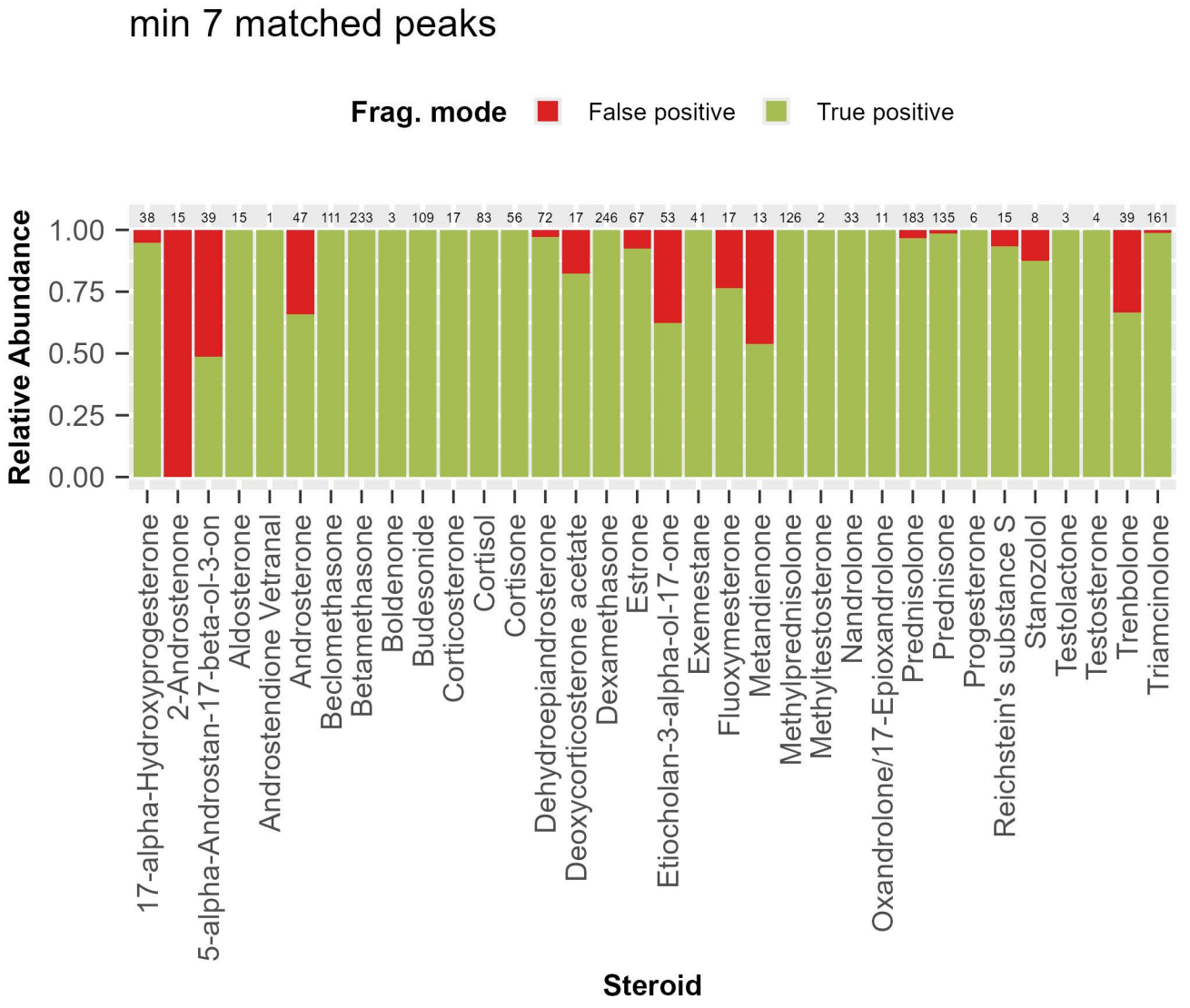

min 7 matched peaks
Frag. mode
False positive
True positive
Relative Abundance
1.00
0.75
0.50
0.25
0.00
38 15 39 15 1 47 111 233 3 109 17 83 56 72 17 246 67 53 41 17 13 126 2 33 11 183 135 6 15 8 3 4 39 161
17-alpha-Hydroxyprogesterone
2-Androstenone
5-alpha-Androstan-17-beta-ol-3-on
Aldosterone
Androstendione Vetranal
Androsterone
Beclomethasone
Betamethasone
Boldenone
Budesonide
Corticosterone
Cortisol
Cortisone
Dehydroepiandrosterone
Deoxycorticosterone acetate
Dexamethasone
Estrone
Etiocholan-3-alpha-ol-17-one
Exemestane
Fluoxymesterone
Metandienone
Methylprednisolone
Methyltestosterone
Nandrolone
Oxandrolone/17-Epioxandrolone
Prednisolone
Prednisone
Progesterone
Reichstein's substance S
Stanozolol
Testolactone
Testosterone
Trenbolone
Triamcinolone
Steroid

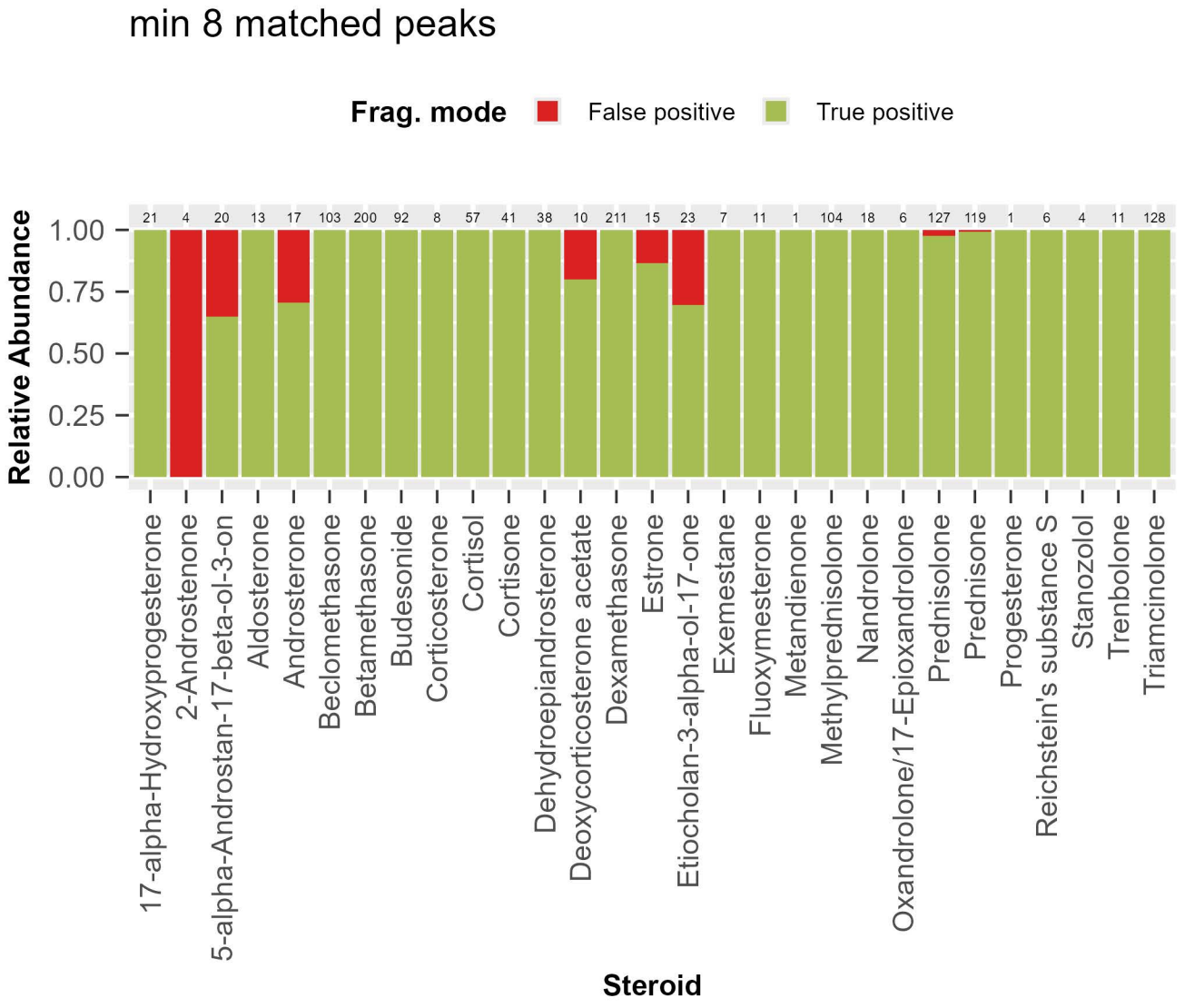
min 8 matched peaks
Frag. mode
False positive
True positive
Relative Abundance
1.00
0.75
0.50
0.25
0.00
21
4
20
13
17
103
200
92
8
57
41
38
10
211
15
23
7
11
1
104
18
6
127
119
1
6
4
11
128
17-alpha-Hydroxyprogesterone
2-Androstenone
5-alpha-Androstan-17-beta-ol-3-on
Aldosterone
Androsterone
Beclomethasone
Betamethasone
Budesonide
Corticosterone
Cortisol
Cortisone
Dehydroepiandrosterone
Deoxycorticosterone acetate
Dexamethasone
Estrone
Etiocholan-3-alpha-ol-17-one
Exemestane
Fluoxymesterone
Metandienone
Methylprednisolone
Nandrolone
Oxandrolone/17-Epioxandrolone
Prednisolone
Prednisone
Progesterone
Reichstein's substance S
Stanozolol
Trenbolone
Triamcinolone
Steroid

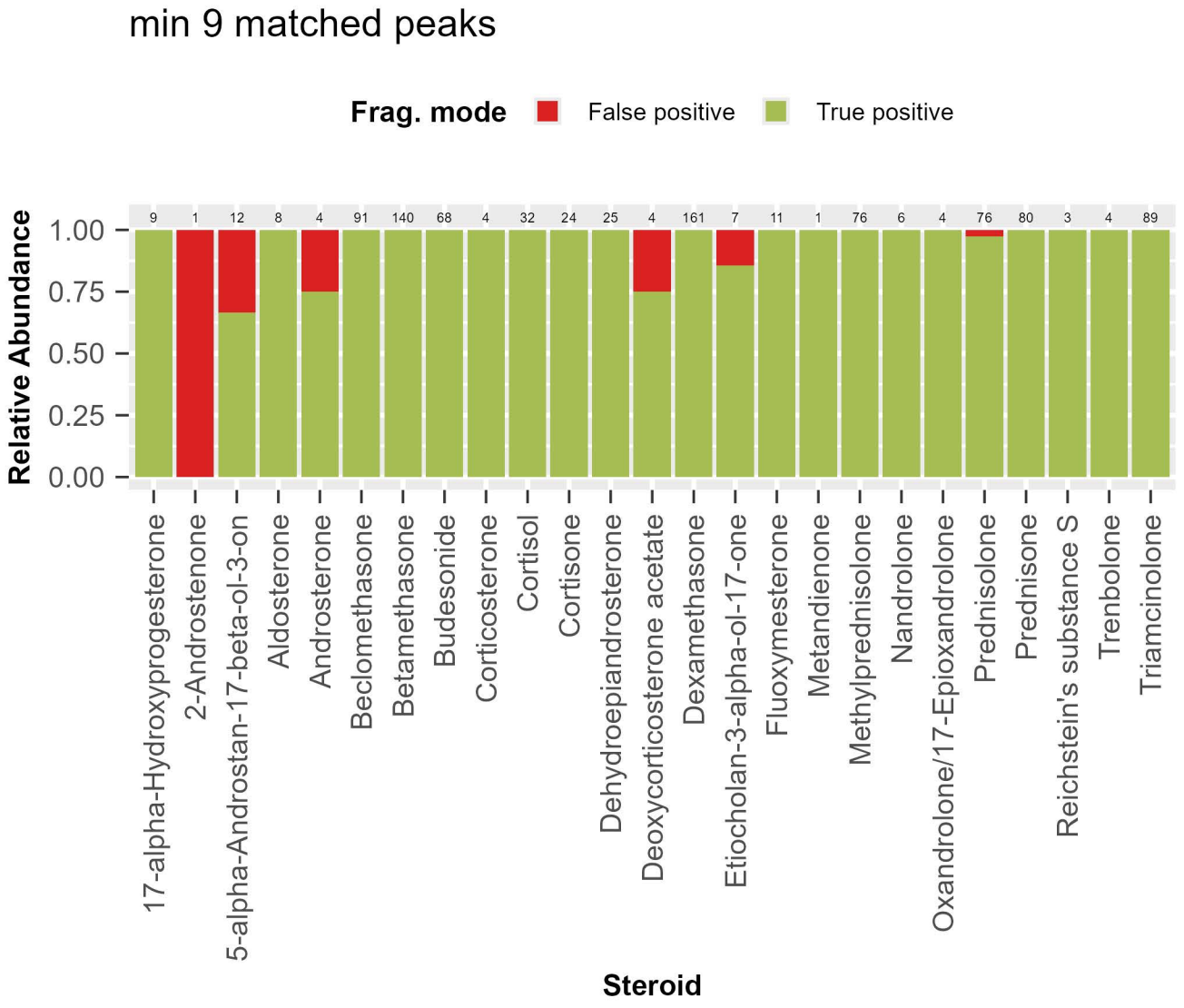

min 9 matched peaks
Frag. mode
False positive
True positive
Relative Abundance
1.00
0.75
0.50
0.25
0.00
9
1
12
8
4
91
140
68
4
32
24
25
4
161
7
11
1
76
6
4
76
80
3
4
89
17-alpha-Hydroxyprogesterone
2-Androstenone
5-alpha-Androstan-17-beta-ol-3-on
Aldosterone
Androsterone
Beclomethasone
Betamethasone
Budesonide
Corticosterone
Cortisol
Cortisone
Dehydroepiandrosterone
Deoxycorticosterone acetate
Dexamethasone
Etiocholan-3-alpha-ol-17-one
Fluoxymesterone
Metandienone
Methylprednisolone
Nandrolone
Oxandrolone/17-Epioxandrolone
Prednisolone
Prednisone
Reichstein's substance S
Trenbolone
Triamcinolone
Steroid

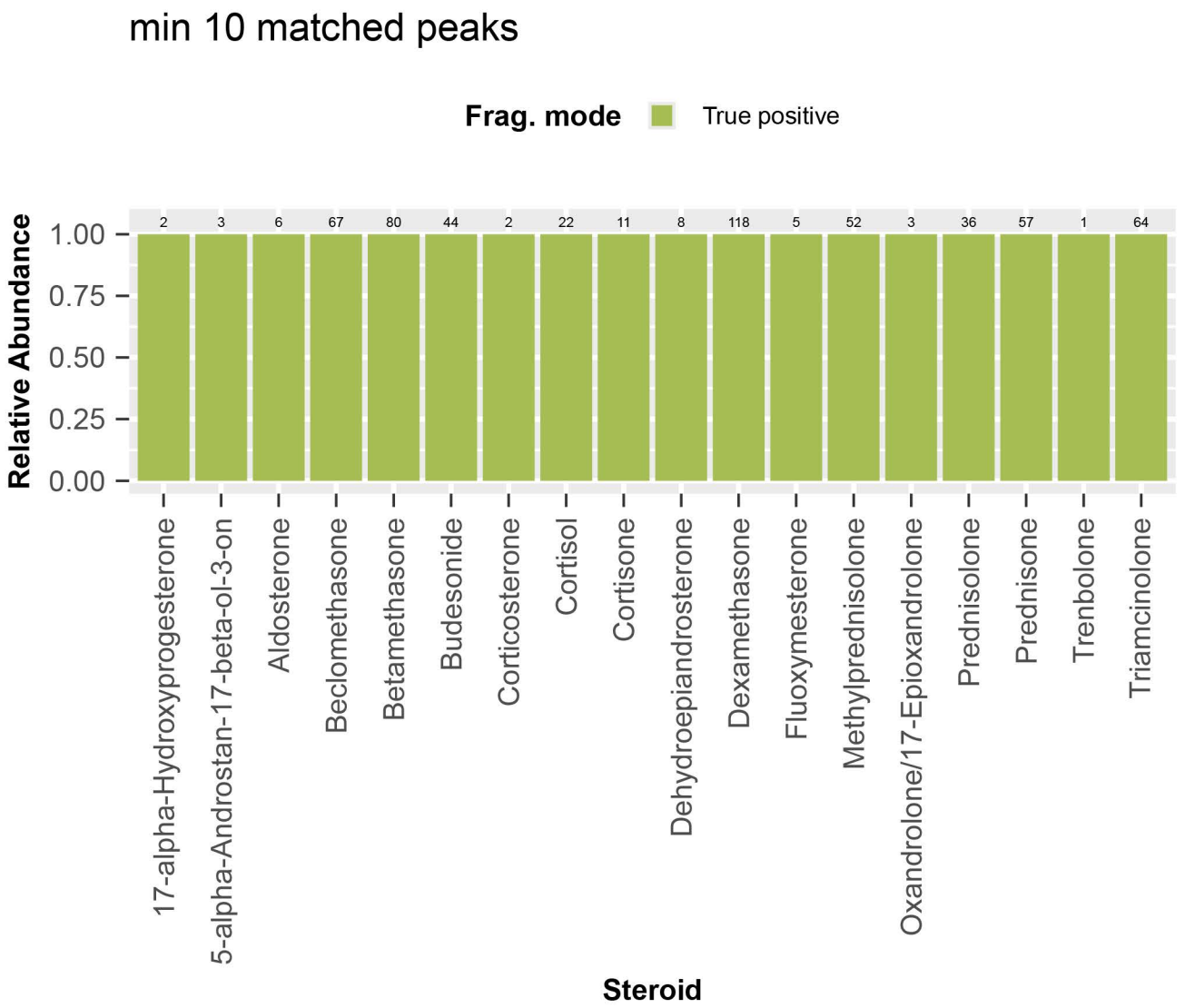

min 10 matched peaks
Frag. mode
True positive
Relative Abundance
1.00
0.75
0.50
0.25
0.00
2
3
6
67
80
44
2
22
11
8
118
5
52
3
36
57
1
64
17-alpha-Hydroxyprogesterone
5-alpha-Androstan-17-beta-ol-3-on
Aldosterone
Beclomethasone
Betamethasone
Budesonide
Corticosterone
Cortisol
Cortisone
Dehydroepiandrosterone
Dexamethasone
Fluoxymesterone
Methylprednisolone
Oxandrolone/17-Epioxandrolone
Prednisolone
Prednisone
Trenbolone
Triamcinolone
Steroid

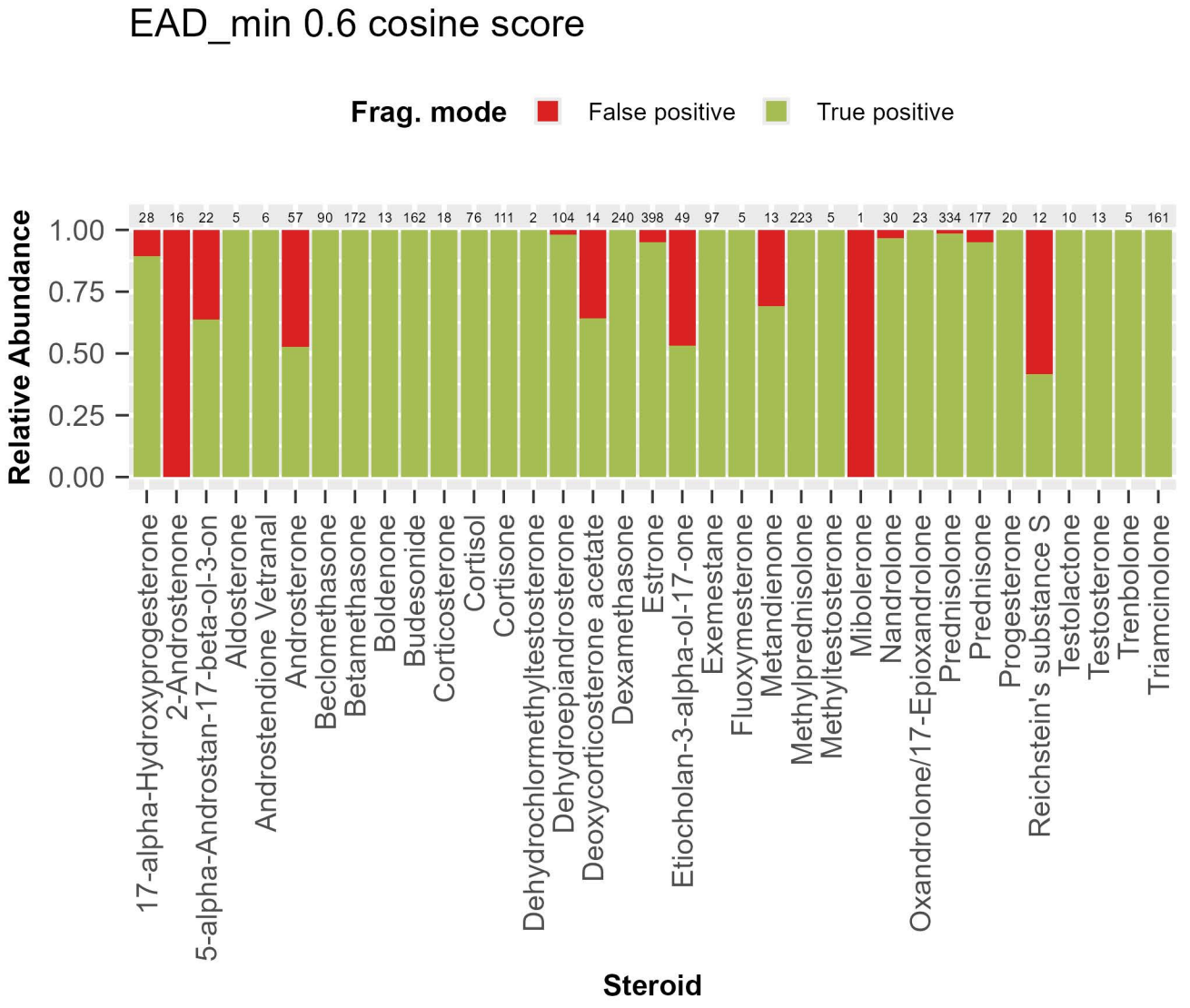

EAD_min 0.6 cosine score
Frag. mode
False positive
True positive
Relative Abundance
1.00
0.75
0.50
0.25
0.00
28 16 22 5 6 57 90 172 13 162 18 76 111 2 104 14 240 398 49 97 5 13 223 5 1 30 23 334 177 20 12 10 13 5 161
17-alpha-Hydroxyprogesterone
2-Androstenone
5-alpha-Androstan-17-beta-ol-3-on
Aldosterone
Androstendione Vetranal
Androsterone
Beclomethasone
Betamethasone
Boldenone
Budesonide
Corticosterone
Cortisol
Cortisone
Dehydrochlormethyltestosterone
Dehydroepiandrosterone
Deoxycorticosterone acetate
Dexamethasone
Estrone
Etiocholan-3-alpha-ol-17-one
Exemestane
Fluoxymesterone
Metandienone
Methylprednisolone
Methyltestosterone
Mibolerone
Nandrolone
Oxandrolone/17-Epioxandrolone
Prednisolone
Prednisone
Progesterone
Reichstein's substance S
Testolactone
Testosterone
Trenbolone
Triamcinolone
Steroid

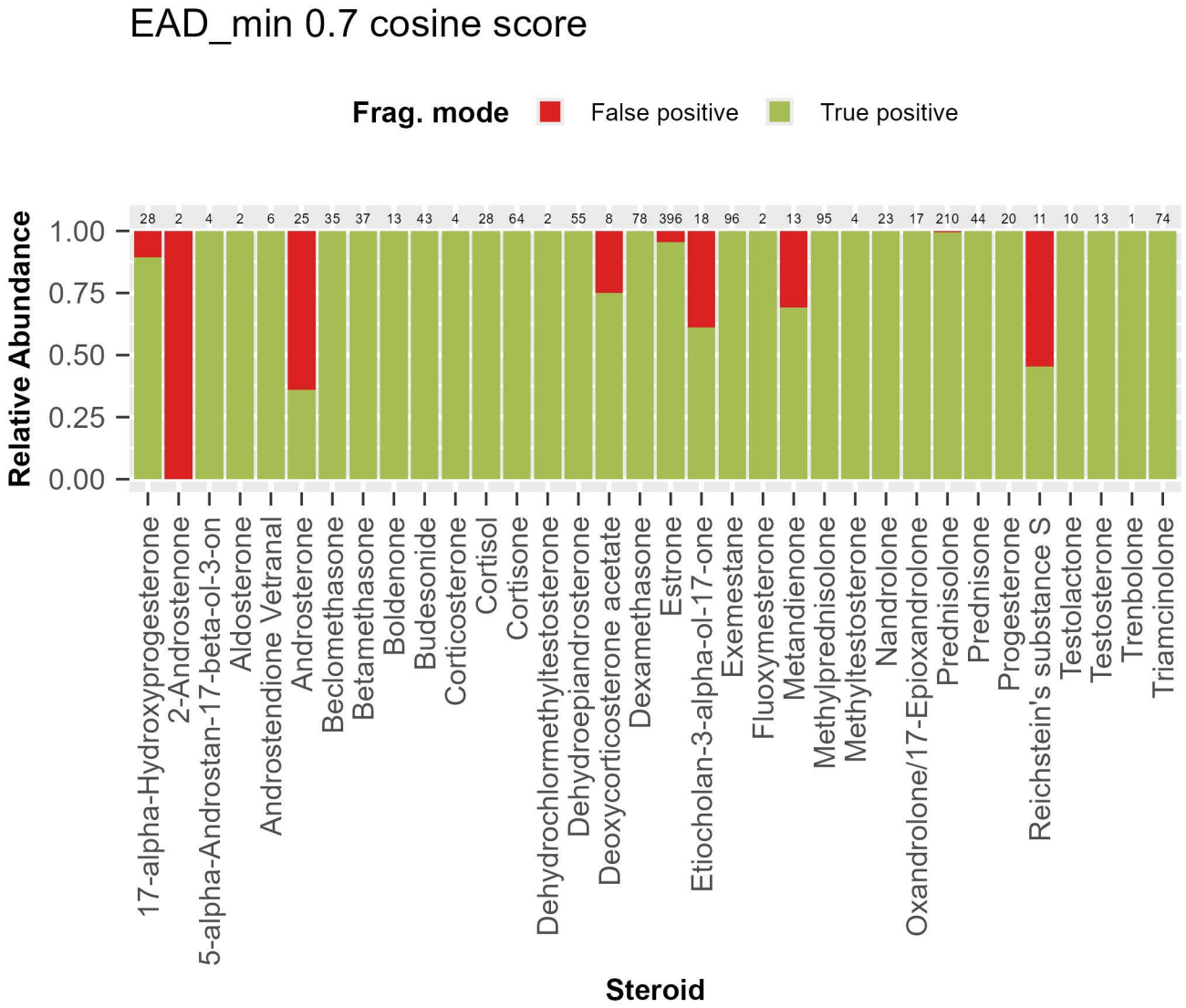
EAD_min 0.7 cosine score
Frag. mode
False positive
True positive
Relative Abundance
1.00
0.75
0.50
0.25
0.00
28 2 4 2 6 25 35 37 13 43 4 28 64 2 55 8 78 396 18 96 2 13 95 4 23 17 210 44 20 11 10 13 1 74
17-alpha-Hydroxyprogesterone
2-Androstenone
5-alpha-Androstan-17-beta-ol-3-on
Aldosterone
Androstendione Vetranal
Androsterone
Beclomethasone
Betamethasone
Boldenone
Budesonide
Corticosterone
Cortisol
Cortisone
Dehydrochlormethyltestosterone
Dehydroepiandrosterone
Deoxycorticosterone acetate
Dexamethasone
Estrone
Etiocholan-3-alpha-ol-17-one
Exemestane
Fluoxymesterone
Metandienone
Methylprednisolone
Methyltestosterone
Nandrolone
Oxandrolone/17-Epioxandrolone
Prednisolone
Prednisone
Progesterone
Reichstein's substance S
Testolactone
Testosterone
Trenbolone
Triamcinolone
Steroid

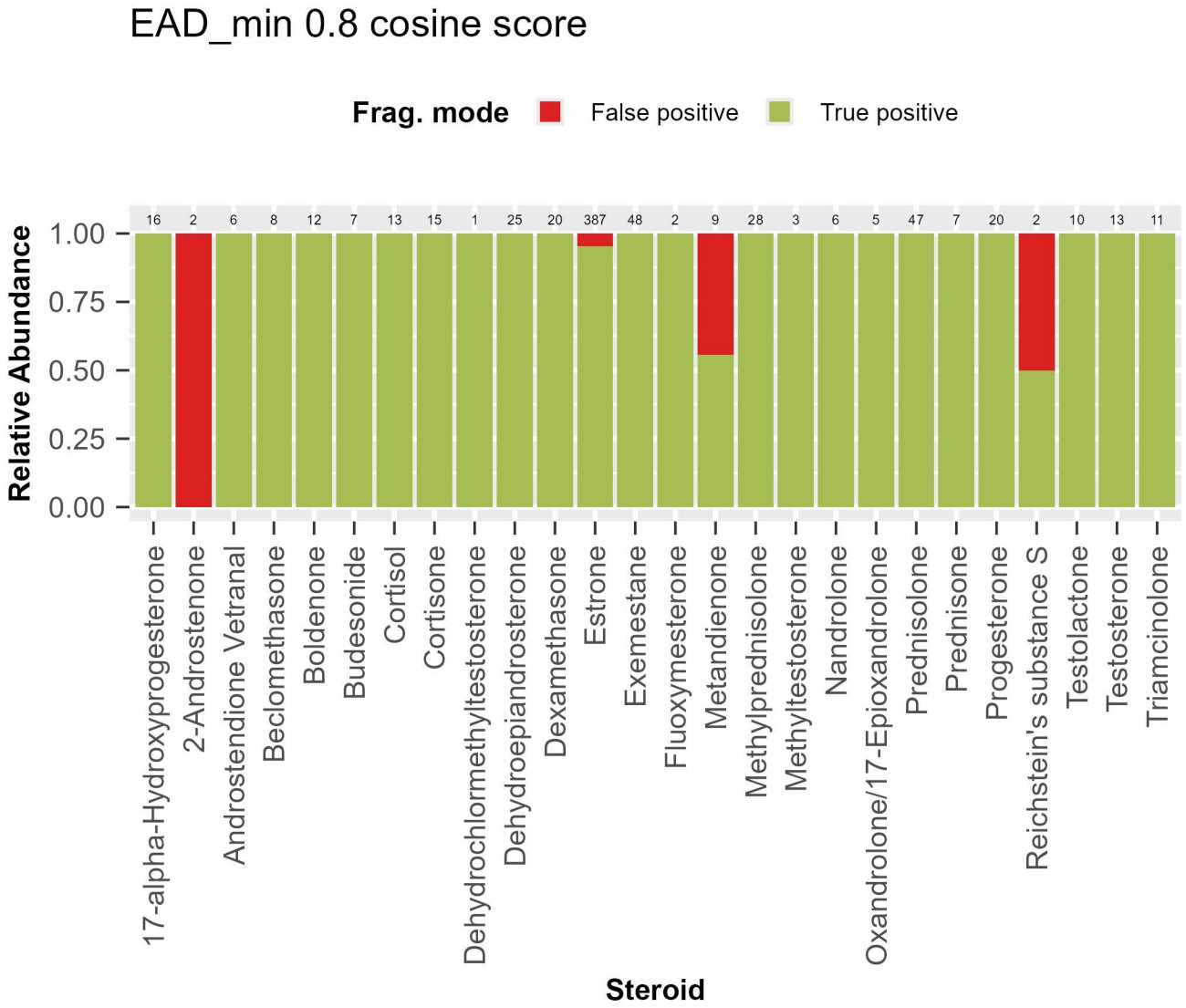

EAD_min 0.8 cosine score
Frag. mode
False positive
True positive
Relative Abundance
1.00
0.75
0.50
0.25
0.00
16
2
6
8
12
7
13
15
1
25
20
387
48
2
9
28
3
6
5
47
7
20
2
10
13
11
17-alpha-Hydroxyprogesterone
2-Androstenone
Androstendione Vetranal
Beclomethasone
Boldenone
Budesonide
Cortisol
Cortisone
Dehydrochlormethyltestosterone
Dehydroepiandrosterone
Dexamethasone
Estrone
Exemestane
Fluoxymesterone
Metandienone
Methylprednisolone
Methyltestosterone
Nandrolone
Oxandrolone/17-Epioxandrolone
Prednisolone
Prednisone
Progesterone
Reichstein's substance S
Testolactone
Testosterone
Triamcinolone
Steroid

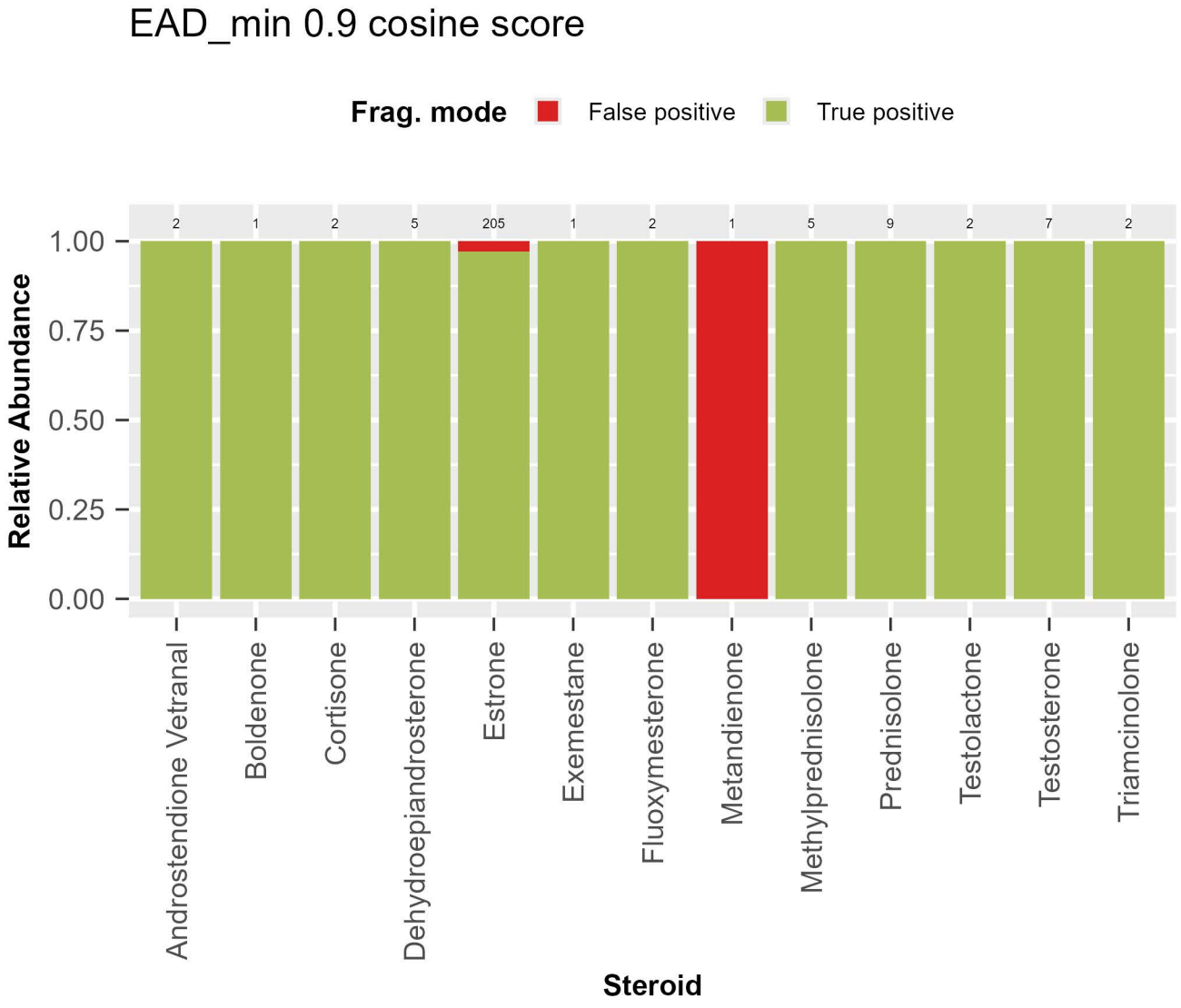

EAD_min 0.9 cosine score
Frag. mode
False positive
True positive
Relative Abundance
1.00
0.75
0.50
0.25
0.00
2
1
2
5
205
1
2
1
5
9
2
7
2
Androstendione Vetranal
Boldenone
Cortisone
Dehydroepiandrosterone
Estrone
Exemestane
Fluoxymesterone
Metandienone
Methylprednisolone
Prednisolone
Testolactone
Testosterone
Triamcinolone
Steroid

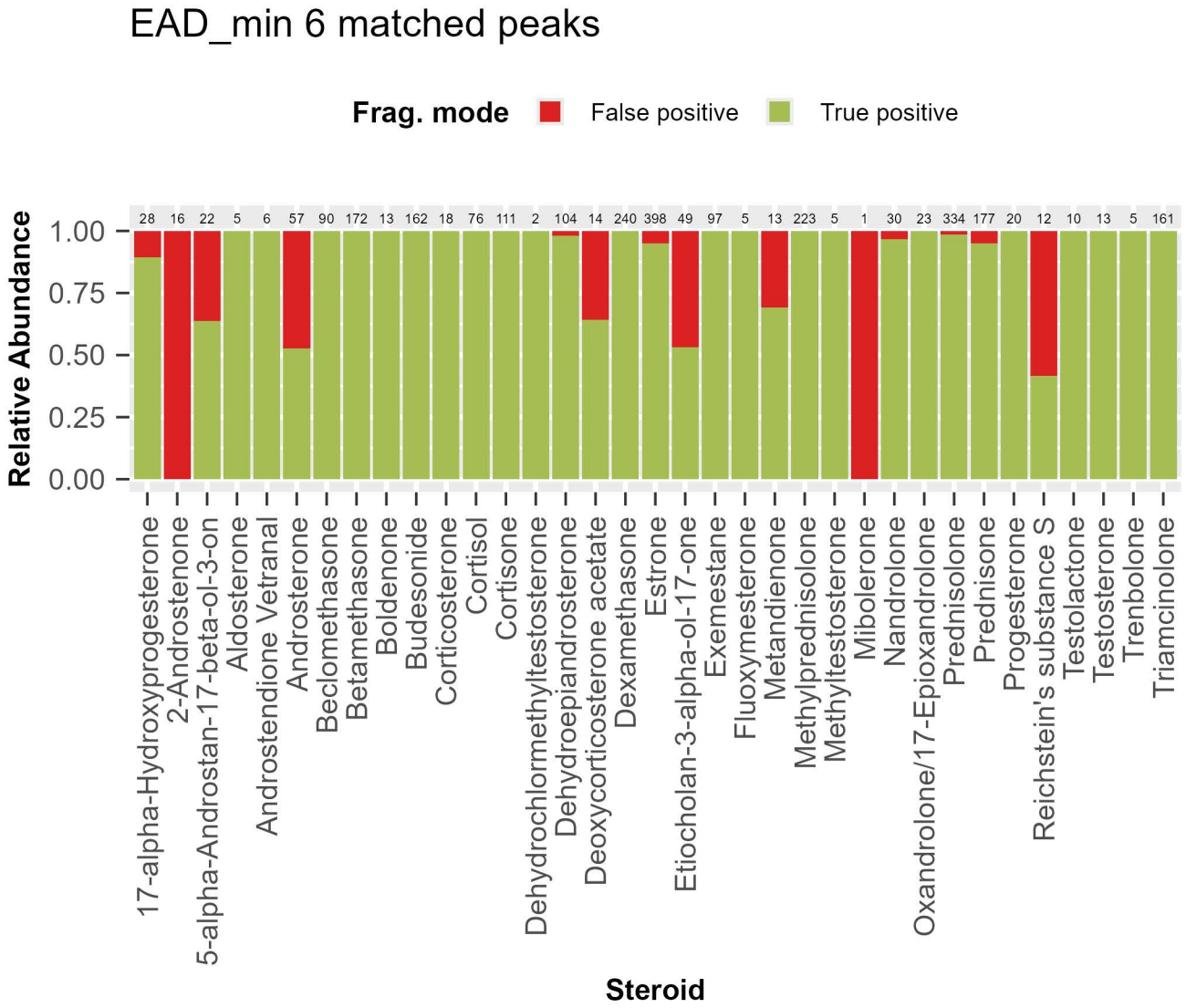

EAD_min 6 matched peaks
Frag. mode
False positive
True positive
Relative Abundance
1.00
0.75
0.50
0.25
0.00
28 16 22 5 6 57 90 172 13 162 18 76 111 2 104 14 240 398 49 97 5 13 223 5 1 30 23 334 177 20 12 10 13 5 161
17-alpha-Hydroxyprogesterone
2-Androstenone
5-alpha-Androstan-17-beta-ol-3-on
Aldosterone
Androstendione Vetranal
Androsterone
Beclomethasone
Betamethasone
Boldenone
Budesonide
Corticosterone
Cortisol
Cortisone
Dehydrochlormethyltestosterone
Dehydroepiandrosterone
Deoxycorticosterone acetate
Dexamethasone
Estrone
Etiocholan-3-alpha-ol-17-one
Exemestane
Fluoxymesterone
Metandienone
Methylprednisolone
Methyltestosterone
Mibolerone
Nandrolone
Oxandrolone/17-Epioxandrolone
Prednisolone
Prednisone
Progesterone
Reichstein's substance S
Testolactone
Testosterone
Trenbolone
Triamcinolone
Steroid

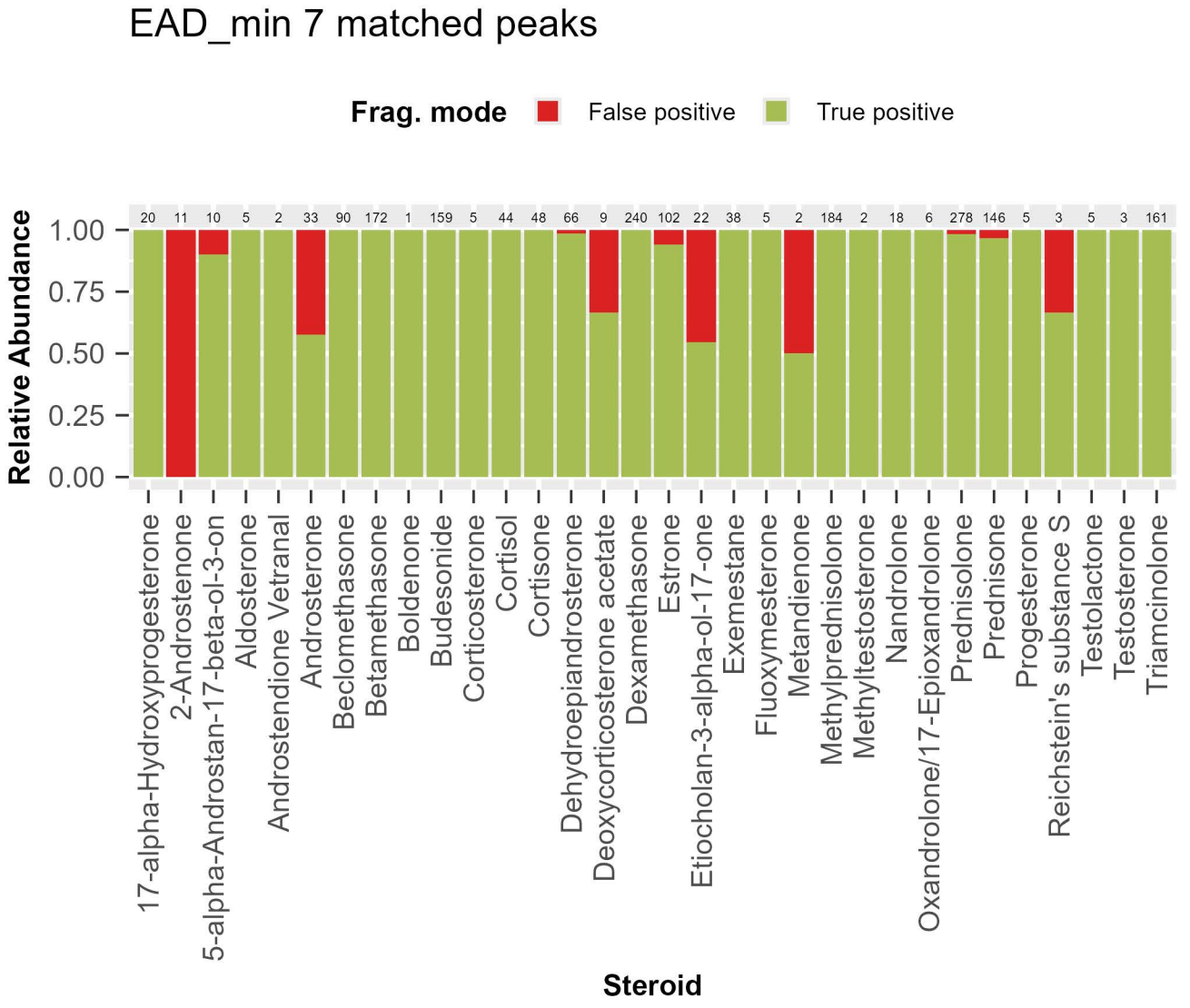

EAD_min 7 matched peaks
Frag. mode
False positive
True positive
Relative Abundance
1.00
0.75
0.50
0.25
0.00
20
11
10
5
2
33
90
172
1
159
5
44
48
66
9
240
102
22
38
5
2
184
2
18
6
278
146
5
3
5
3
161
17-alpha-Hydroxyprogesterone
2-Androstenone
5-alpha-Androstan-17-beta-ol-3-on
Aldosterone
Androstendione Vetranal
Androsterone
Beclomethasone
Betamethasone
Boldenone
Budesonide
Corticosterone
Cortisol
Cortisone
Dehydroepiandrosterone
Deoxycorticosterone acetate
Dexamethasone
Estrone
Etiocholan-3-alpha-ol-17-one
Exemestane
Fluoxymesterone
Metandienone
Methylprednisolone
Methyltestosterone
Nandrolone
Oxandrolone/17-Epioxandrolone
Prednisolone
Prednisone
Progesterone
Reichstein's substance S
Testolactone
Testosterone
Triamcinolone
Steroid

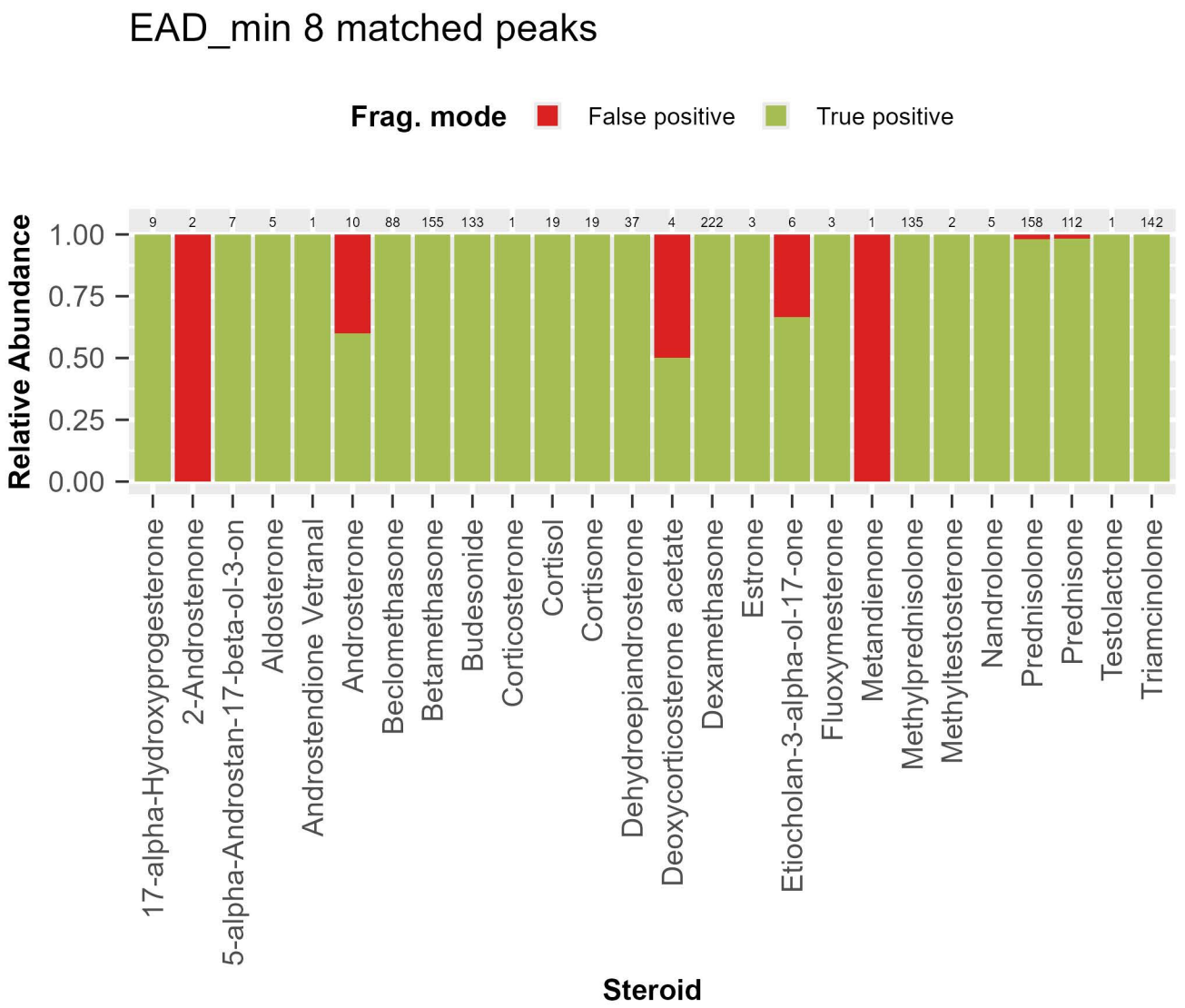
EAD_min 8 matched peaks
Frag. mode
False positive
True positive
Relative Abundance
1.00
0.75
0.50
0.25
0.00
9
2
7
5
1
10
88
155
133
1
19
19
37
4
222
3
6
3
1
135
2
5
158
112
1
142
17-alpha-Hydroxyprogesterone
2-Androstenone
5-alpha-Androstan-17-beta-ol-3-on
Aldosterone
Androstendione Vetranal
Androsterone
Beclomethasone
Betamethasone
Budesonide
Corticosterone
Cortisol
Cortisone
Dehydroepiandrosterone
Deoxycorticosterone acetate
Dexamethasone
Estrone
Etiocholan-3-alpha-ol-17-one
Fluoxymesterone
Metandienone
Methylprednisolone
Methyltestosterone
Nandrolone
Prednisolone
Prednisone
Testolactone
Triamcinolone
Steroid

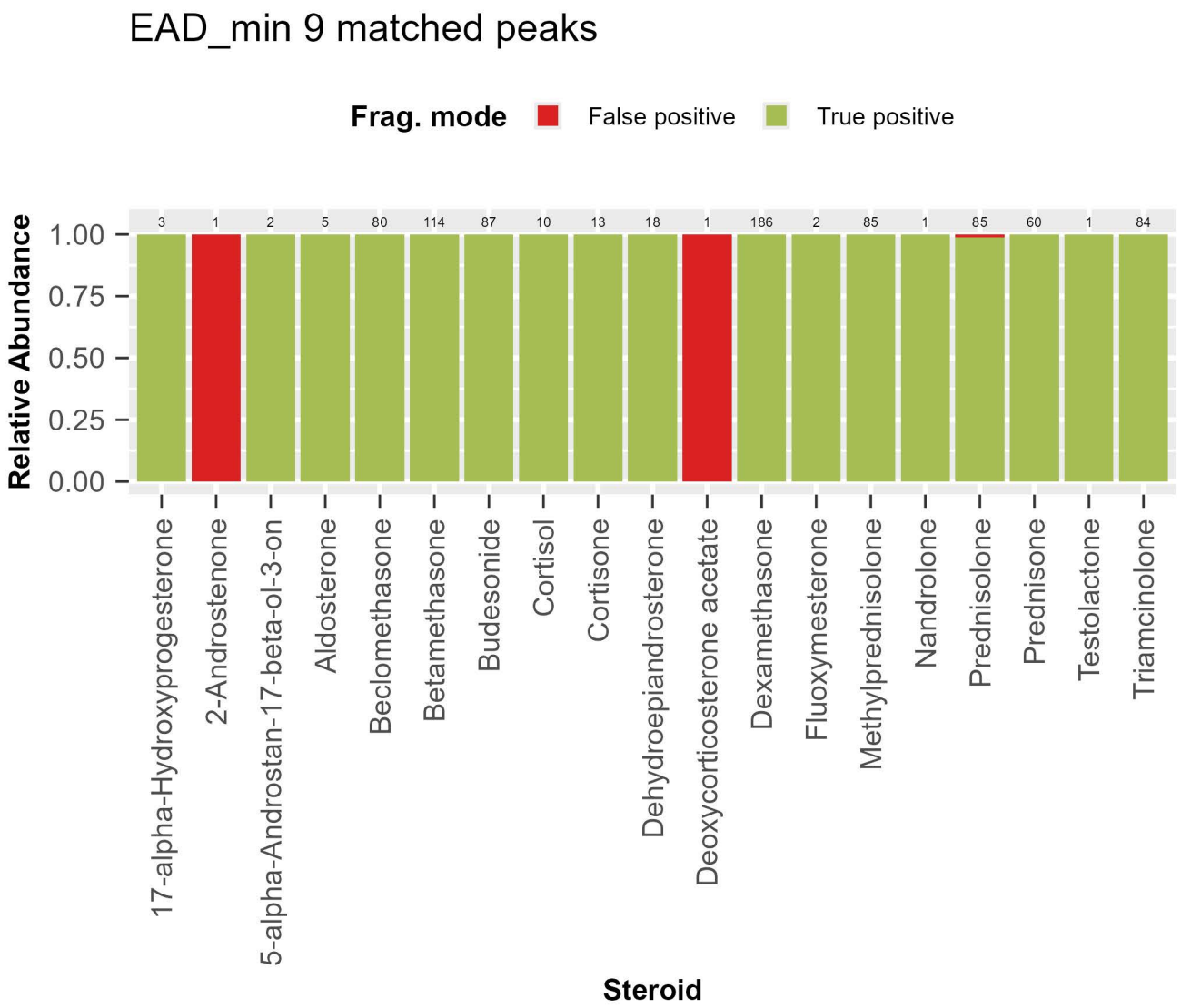
EAD_min 9 matched peaks
Frag. mode
False positive
True positive
Relative Abundance
1.00
0.75
0.50
0.25
0.00
3
1
2
5
80
114
87
10
13
18
1
186
2
85
1
85
60
1
84
17-alpha-Hydroxyprogesterone
2-Androstenone
5-alpha-Androstan-17-beta-ol-3-on
Aldosterone
Beclomethasone
Betamethasone
Budesonide
Cortisol
Cortisone
Dehydroepiandrosterone
Deoxycorticosterone acetate
Dexamethasone
Fluoxymesterone
Methylprednisolone
Nandrolone
Prednisolone
Prednisone
Testolactone
Triamcinolone
Steroid

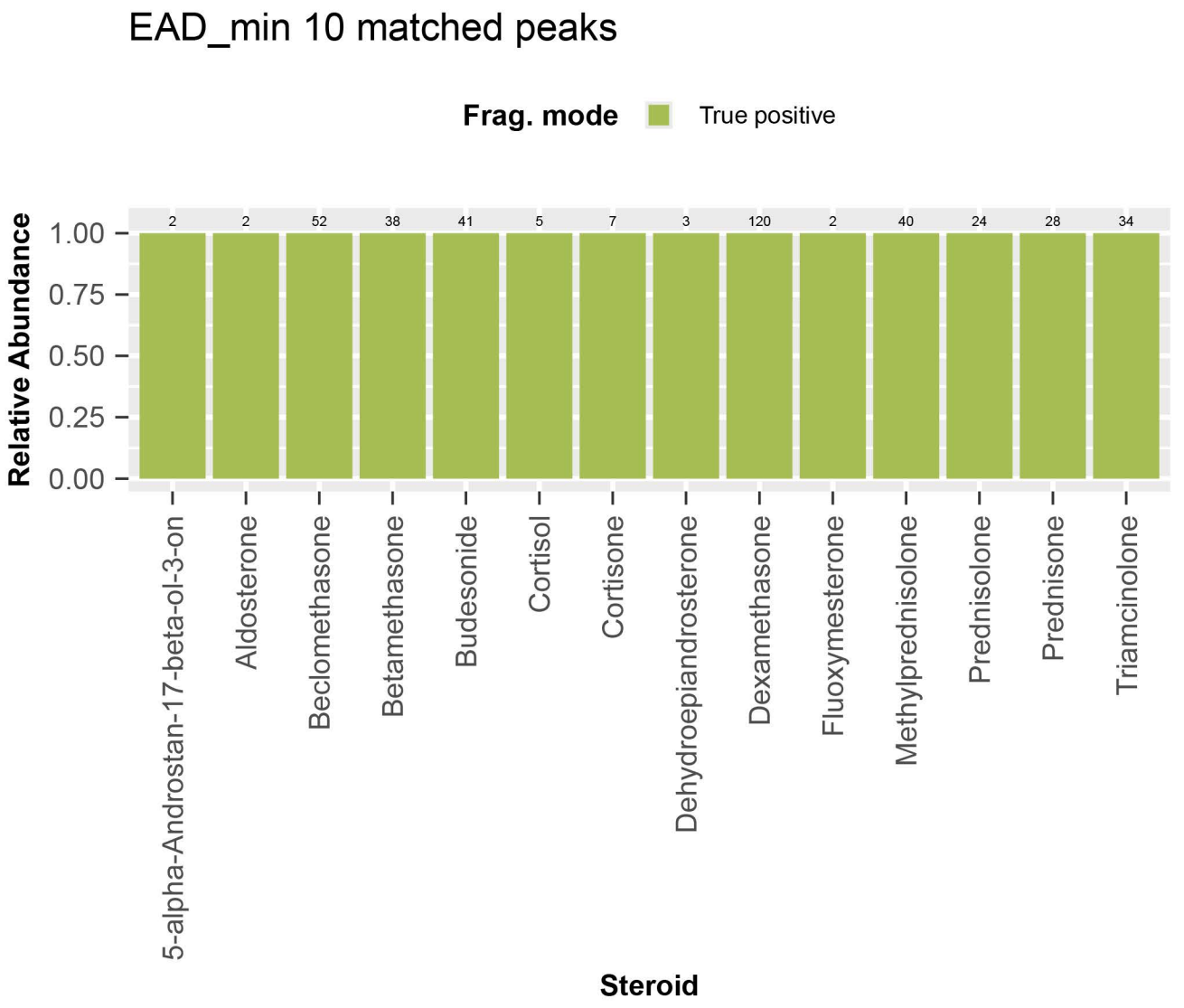

EAD_min 10 matched peaks
Frag. mode
True positive
Relative Abundance
1.00
0.75
0.50
0.25
0.00
2
2
52
38
41
5
7
3
120
2
40
24
28
34
5-alpha-Androstan-17-beta-ol-3-on
Aldosterone
Beclomethasone
Betamethasone
Budesonide
Cortisol
Cortisone
Dehydroepiandrosterone
Dexamethasone
Fluoxymesterone
Methylprednisolone
Prednisolone
Prednisone
Triamcinolone
Steroid
