# Supplementary material for: From Single Injection to Dual Insights: Integrating EAD and CID Acquisition for Spectral Library Generation and EAD–CID Spectral Matching
Source: Anal Chem. 2026 Oct 2;98(41):29433–44. doi: 10.1021/acs.analchem.6c01204 (PMC13689410; doi:10.1021/acs.analchem.6c01204)
Supplement: Supplementary file 3 [file ac6c01204_si_004.pdf]

Supporting Information

From Single Injection to Dual Insights: Integrating EAD and CID acquisition for Spectral Library Generation and EAD–CID Spectral Matching

Lisa Panzenboeck[1,2], Christina Brenner[1,2], Veronika Fitz[1,2], Mingxun Wang[3], Patrick Mueller[1], Evelyn Rampler[1,2], Yasin El Abiead[4]* and Gunda Koellensperger[1,5]*

1 Department of Analytical Chemistry, Faculty of Chemistry, University of Vienna, 1090 Vienna, Austria

2 Vienna Doctoral School in Chemistry (DoSChem), University of Vienna, 1090 Vienna, Austria

3 Department of Computer Science and Engineering, University of California Riverside, Riverside, California 92521, United States

4 BOKU University, Department of Chemistry, Institute of Analytical Chemistry, Muthgasse 18, 1190, Vienna, Austria

5 Vienna Metabolomics Center (VIME), University of Vienna, 1090, Vienna, Austria

* Corresponding authors:

Gunda Koellensperger: gunda.koellensperger@univie.ac.at, +43-1-0277-52303

Yasin El Abiead: yasin.el-abiead@boku.ac.at, +43-1-47654-77109

Table of Contents

Supplementary Experimental Section S2

Supplementary Results S4

Adaptation of the GNPS Dashboard S4

Assessment of data quality S5

Application of steroid MS/MS libraries S8

Supplementary References S13

Supplementary Experimental Section

The following MS-acquisition parameters were used for the LC-MS measurements: curtain gas 35, CAD gas: 7, ion source gas 1: 40 psi, ion source gas 2: 50 psi, temperature: 650 °C, spray voltage: 4500 V, TOFMS scan range: 90–1350 Da, accumulation time: 0.2 s, DP: 100 V, CE 10 V. For the IDA scans dynamic background subtraction was used with a maximum of 10 candidate ions, an intensity threshold of 100 cps and a Zeno threshold of 20000 cps. The TOFMSMS scan range was set from 80 to 500 Da using an accumulation time of 0.15 s, a DP of 100 V, and a CID CE of 20 V. For some steroids, LC-MS files were acquired in MRM^{HR} mode using selected fragmentation energies for CID and EAD and reduced accumulation times of 0.1 s for CID compared to the original FI-MRM^{HR} measurements.

To ensure the high quality of the generated MS/MS scans, fragmentation patterns were compared to in silico predictions using SCIEX OS. This evaluation was performed at a CID collision energy of 30 V and an EAD electron kinetic energy of 14 eV, considering only fragments with an intensity above approximately 50 counts per second, using the following settings: only break single bonds: false, break ring bonds: true, maximum number of bonds to break: 4, maximum number of C-C bonds to break: 4, allow one bond closure: true, include brute force rearrangements: false, allow radicals: true, mass tolerance: 20 ppm., constrain using peak list: true, require evidence for previous step when breaking bond: true.

To generate MS/MS spectral libraries from centroided data mzmine[1,2] processing settings were as follows:

1. Import MS data: Import centroided .mzML files to mzmine.
   Advanced import: Scan filter RT 0.9580-3.5105 min. All MS levels, Polarity: positive, Spectrum type: centroided. MS1 detector noise level: 5E2, MS2 detector noise level: 5E1. Sort and color applied. (Noise levels must be adapted by the user. Especially for EAD, it might be relevant to set lower thresholds.)
2. Mass detection: Scans were filtered for MS1 scans with a noise level of 5E2 and subsequently for MS2 scans (MSn, level $\geq$ 2) with a noise level of 5E1.
3. MSn tree feature list builder was applied for MS1 EIC scans with a scan-to-scan m/z tolerance of 0.005 m/z or 10.0 ppm.
4. Local compound database search was performed using a custom metadata file, importing “mz”, “smiles”, “inchi”, “inchi_key”, and “name”. The m/z tolerance was set to 0.004 m/z or 8.0 ppm and file name headers were filtered for “unique_sample_id”. (Additionally, RT tolerance was 9999999.0 minutes, and Mobility, as well as CCS tolerance (which were not important in this case), were left unchanged at 0.01 and 5%.
5. Batch spectral library generation was performed using an MS level filter $\geq$ 2 without intensity normalization but with average fragment scan intensity merging for each sample and each energy with an m/z tolerance of 0.005 m/z or 20.0 ppm. Chimeric spectra were flagged. At least three signals needed to be present, and the minimum explained intensity was set to 35%, m/z tolerances were set as before. Spectral libraries were exported to .mgf and .json format. For S24 no library entry at a collision energy of 10 V could be exported for CID.
6. The generated .mgf output was used for the FASST[3,4]-search.

We highly recommend that users of this workflow ensure that the mzmine version of their choice performs average-scan merging separately for CID and EAD, rather than merging both fragmentation modes when the same energy was applied.

The FASST search of the novel CID-EAD steroid MS/MS library against publicly available spectral libraries was performed using a cosine threshold of 0.6 and requiring a minimum of 6 matched peaks with “gnpslibrary” as library name. The results were checked for true/false positive hits by comparing the first 14 letters of the respective InChIKeys, followed by manual evaluation and adjustments (e.g., based on compound names deposited in the public libraries). Due to the lack of any MS/MS technique to resolve epimers, epimers (e.g. Betamethasone and Dexamethasone) were counted as true positive hits in this case. Unique true/false positive hits were defined by combinations of a given steroid, hit quality (true/false), fragmentation mode, and InChIKey. Moreover, we provide an overview on the impact of cosine score and matched peaks thresholds on the total number and distribution of true and false positive hits per steroid. (For the additional search of Calusterone "gnpslibrary_no_filters" was used as index.) Thresholds were adapted for the pesticide library, setting a cosine score threshold of 0.7 and requiring a minimum of 3 matched peaks.

Searching the novel CID-EAD steroid MS/MS library against publicly available datasets with FASST, “metabolomicspanrepo_index_nightly” was used as index. Searches against previously existing spectral libraries were performed after deriving the structures of interest based on corresponding SMILES and resulting InChiKeys (converted with rdkit[5]), keeping only data for which ReDU metadata was available. To ensure that the corresponding library entries could be found, we compared the resulting InChiKeys to the masst_records library table (https://masst-records.gnps2.org/masst_records/library_table). If an entry was found with a differing double bond position based on the InChIKey, no further action was taken, as this can be regarded as an artifact originating from the rdkit conversion. If no entry could be found for the respective InChIKey, an additional manual search was performed, and the corresponding results were added to the output table. If it could be deduced from the file name (“neg”, “NEG”, “Neg”), any files recorded in negative ion mode were excluded. Body parts needed to be given; otherwise, the entry was removed to generate the results shown in Figure 3C and Figure 4 of the main text. Both for the library against library and the library against raw data searches Epiandrosterone and Androsterone were both processed as Androsterone. To allow the upload of the created library.mgf file to GNPS, metadata was adjusted to fulfill the requirements of the “SCANS” metadata starting at 1. An overview of the workflow is provided in **Figure S1**.

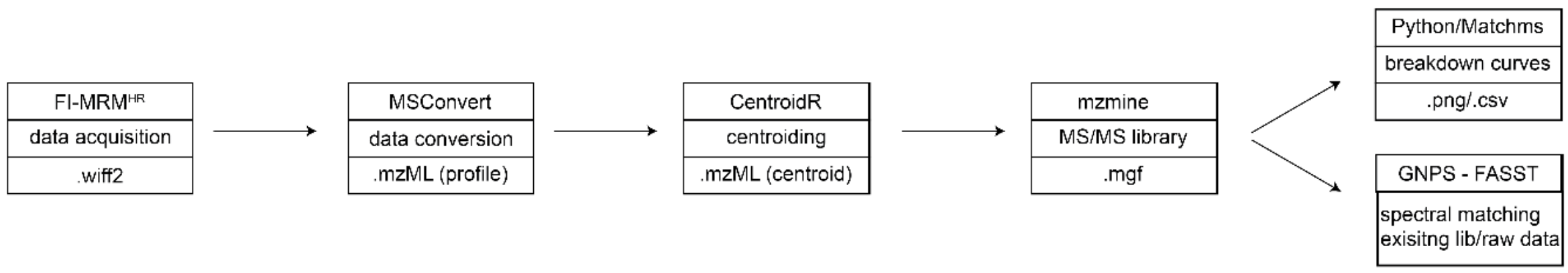

Figure S1: Schematic overview of the workflow. The workflow/program (top bar) is used to perform a specific task/ generate a desired output (middle bar), which results in a file (bottom bar) used in the following step.

Supplementary Results

Adaptation of the GNPS Dashboard

In order to support the distribution of raw data generated through the FI-high-resolution targeted MS/MS workflow presented here we have adapted the GNPS Dashboard[6] to allow the interactive visualization and sharing of FI-high-resolution targeted MS/MS data. For instance, following this link [https://dashboard.gnps2.org//?xic_mz=361.2009&xic_formula=&xic_peptide=&xic_tolerance=0.5&xic_ppm_tolerance=10&xic_tolerance_unit=Da&xic_rt_window=0.587816666667&xic_norm=False&xic_file_grouping=MZ&xic_integration_type=AUC&show_ms2_markers=True&ms2marker_color=blue&ms2marker_size=5&ms2_identifier=MS2%3Asample%3D1+period%3D1+cycle%3D2+experiment%3D17&show_lcms_2nd_map=False&map_plot_zoom=%7B%7D&polarity_filtering=None&polarity_filtering2=None&tic_option=TIC&overlay_usi=None&overlay_mz=None&overlay_rt=None&overlay_color=&overlay_size=&overlay_hover=&overlay_filter_column=&overlay_filter_value=&feature_finding_type=Off&feature_finding_ppm=10&feature_finding_noise=10000&feature_finding_min_peak_rt=0.05&feature_finding_max_peak_rt=1.5&feature_finding_rt_tolerance=0.3&massql_statement=QUERY+scaninfo%28MS2DATA%29&sychronization_session_id=a7d4acffdd5a4356a112f2454011a5f8&chromatogram_options=%5B%5D&comment=&map_plot_color_scale=Hot_r&map_plot_quantization_level=Medium&plot_theme=plotly_white#%7B%22usi%22%3A%20%22mzspec%3AMSV000100658%3Apeak/Peak%20List%20Files/S15_00_062025.mzML%22%2C%20%22usi_select%22%3A%20%22mzspec%3AMSV000100658%3Apeak/Peak%20List%20Files/S15_00_062025.mzML%22%2C%20%22usi2%22%3A%20%22%22%7D] allows to visualize the acquired data as heatmap. Further, for the display of MS/MS scans we have adapted the parsing of scan headers to allow displaying scans given their “samples”, “period”, “cycle”, and “experiment” which is the scan indexing employed by the manufacturer. We also extract the fragmentation type EAD/CID employed from the scan header. For example, here the MS/MS scan for MS2:sample=1 period=1 cycle=5 experiment=16 is visualized in: [https://dashboard.gnps2.org//?xic_mz=361.2009&xic_formula=&xic_peptide=&xic_tolerance=0.5&xic_ppm_tolerance=10&xic_tolerance_unit=Da&xic_rt_window=0.587816666667&xic_norm=False&xic_file_grouping=MZ&xic_integration_type=AUC&show_ms2_markers=True&ms2marker_color=blue&ms2marker_size=5&ms2_identifier=MS2%3Asample%3D1+period%3D1+cycle%3D5+experiment%3D16&show_lcms_2nd_map=False&map_plot_zoom=%7B%7D&polarity_filtering=None&polarity_filtering2=None&tic_option=TIC&overlay_usi=None&overlay_mz=None&overlay_rt=None&overlay_color=&overlay_size=&overlay_hover=&overlay_filter_column=&overlay_filter_value=&feature_finding_type=Off&feature_finding_ppm=10&feature_finding_noise=10000&feature_finding_min_peak_rt=0.05&feature_finding_max_peak_rt=1.5&feature_finding_rt_tolerance=0.3&massql_statement=QUERY+scaninfo%28MS2DATA%29&sychronization_session_id=a7d4acffdd5a4356a112f2454011a5f8&chromatogram_options=%5B%5D&comment=&map_plot_color_scale=Hot_r&map_plot_quantization_level=Medium&plot_theme=plotly_white#%7B%22usi%22%3A%20%22mzspec%3AMSV000100658%3Apeak/Peak%20List%20Files/S15_00_062025.mzML%22%2C%20%22usi_select%22%3A%20%22mzspec%3AMSV000100658%3Apeak/Peak%20List%20Files/S15_00_062025.mzML%22%2C%20%22usi2%22%3A%20%22%22%7D]

Assessment of data quality

For the vast majority of steroids, more than 90% of the experimentally observed fragments agreed with theoretical predictions, as indicated by the green bars in **Figure S2**. Only Aldosterone, Exemestane, Stanozolol, and Budesonide had more than 10% unassigned fragments (yellow bars – **Figure S2**) in CID. For EAD, there were eight steroids in total (Cortisol, Beclomethasone, Boldenone, Androstendione Vetranal, Budesonide, Metenolone, Arimistane, and Stanozolol) for which less than 90% of the experimental fragments could be matched to in silico predicted ones, with an overall maximum percentage of unannotated fragments of 23% for Stanozolol. While a high ratio of unassigned fragments is generally undesirable for high-quality spectral libraries, their corresponding relative intensities should also be considered when thinking about their contribution to the overall fragment pattern and similarity score calculations. For five steroids in total, namely Androsta-1, 4, 6-triene-3, 17-dione, Budesonide (both CID), Boldenone, Metandienone, and Stanozolol (all EAD), unassigned fragments with relative intensities above 10% (up to 28%), could be found.

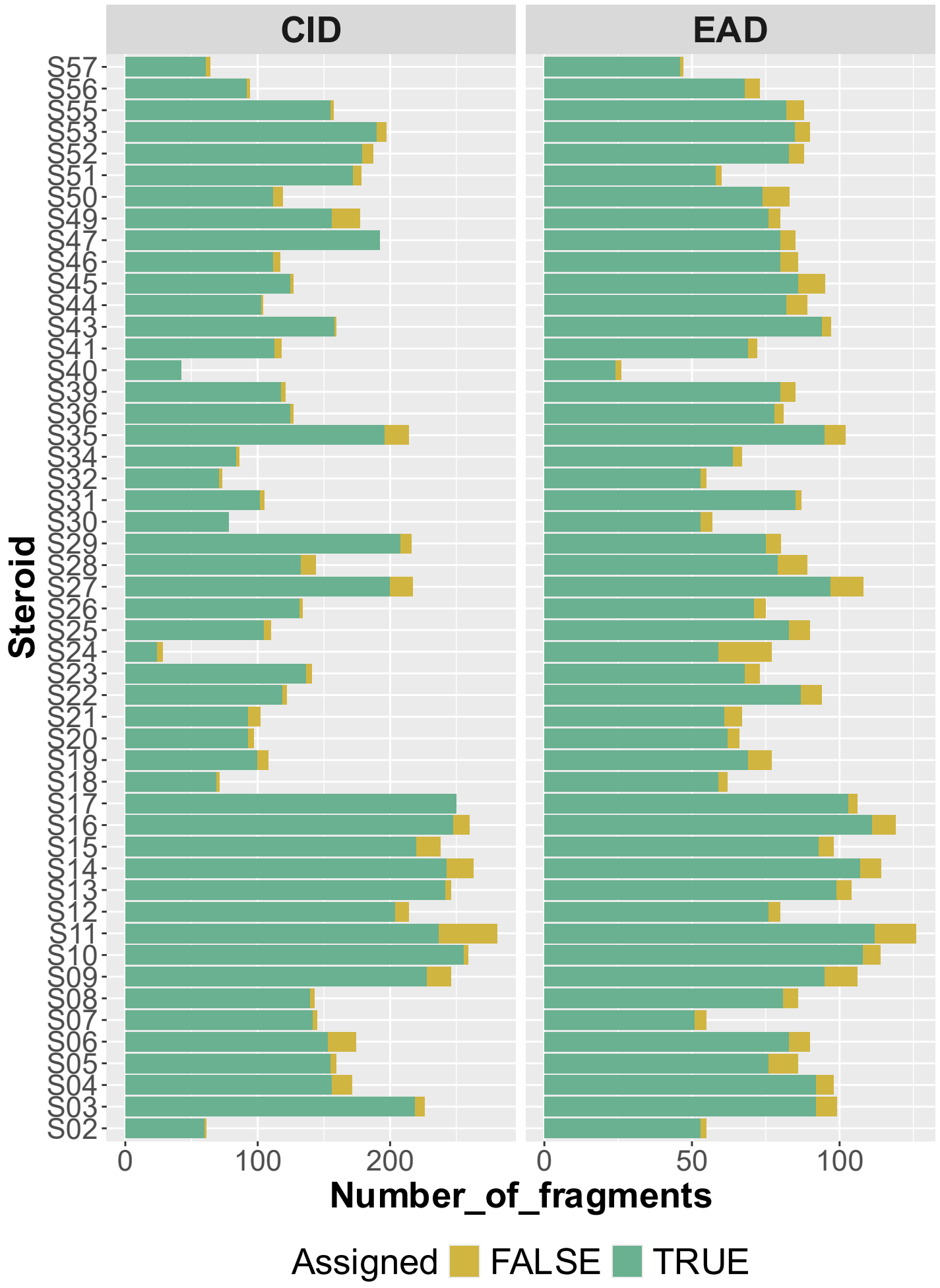

*Figure S2: Experimental MS/MS spectra were matched against in silico predictions for CID (30 V) and EAD (14 eV) with a mass error tolerance of 20 ppm. Fragments that could be assigned to theoretical ones are represented in green; those that could not be matched are shown in yellow. An overview of steroids and corresponding numbers is given in **Supporting Information_Table (SA1).***

Excellent MS/MS mass accuracy could be achieved for CID-based fragmentation, as indicated by sub-7.5 ppm mass errors for all assigned fragments above 25% relative intensity. While mass errors observed in EAD are considerably higher than for CID-based fragments, they are still within the expected mass accuracy regime of QTOF mass spectrometers. As displayed in

Figure S3, fragments originating from CID (violet) have lower mass errors than those originating from EAD (teal).

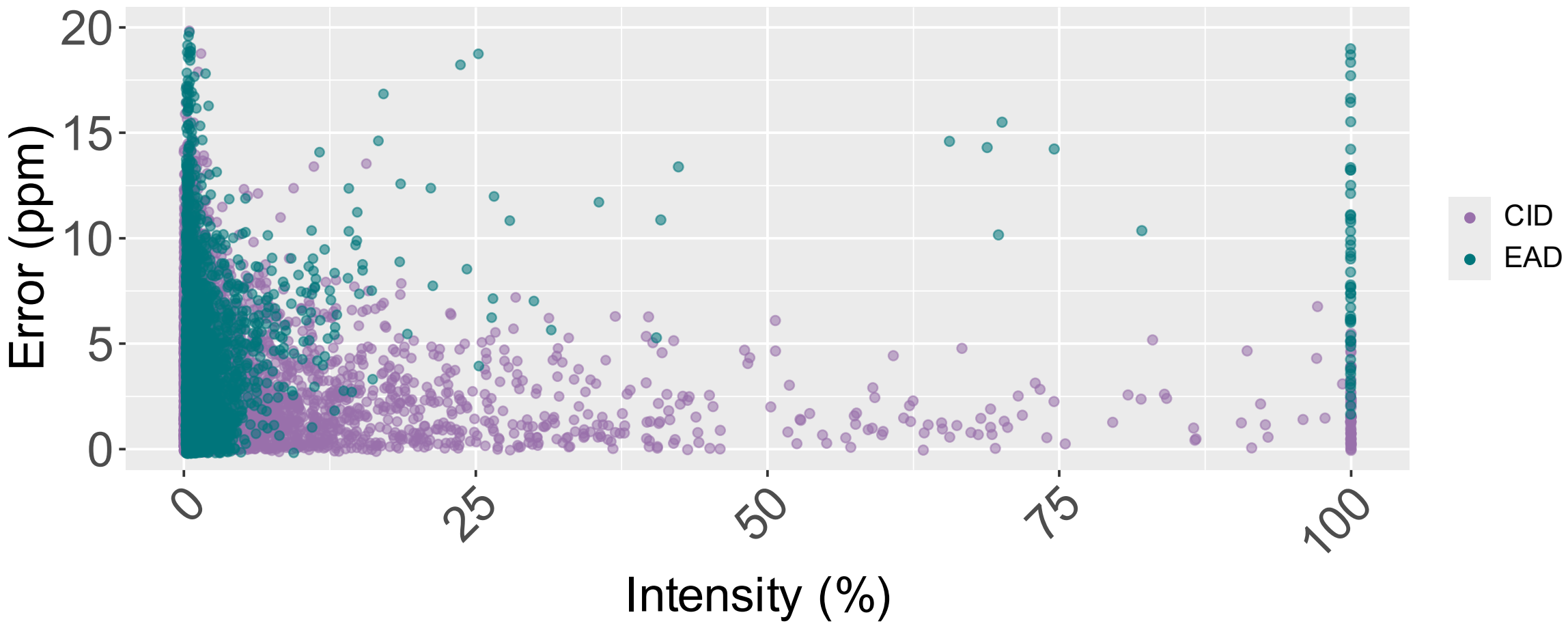

Figure S3: Distribution of mass errors of in silico assigned fragments for CID (30 V, violet) and EAD (14 eV, teal). While excellent mass accuracy is achieved for CID, higher deviations from the accurate mass are seen for EAD.

Cosine scores were first calculated across all eight MS/MS triggering events for each steroid acquired under the same fragmentation parameters. For each steroid and fragmentation mode (CID/EAD), average cosine scores across fragmentation energies were calculated. As shown in **Figure S4**, MS/MS spectra are highly reproducible across MS/MS triggering events, with mean cosine scores >0.99 (see **Supporting Information Table (SA2)**).

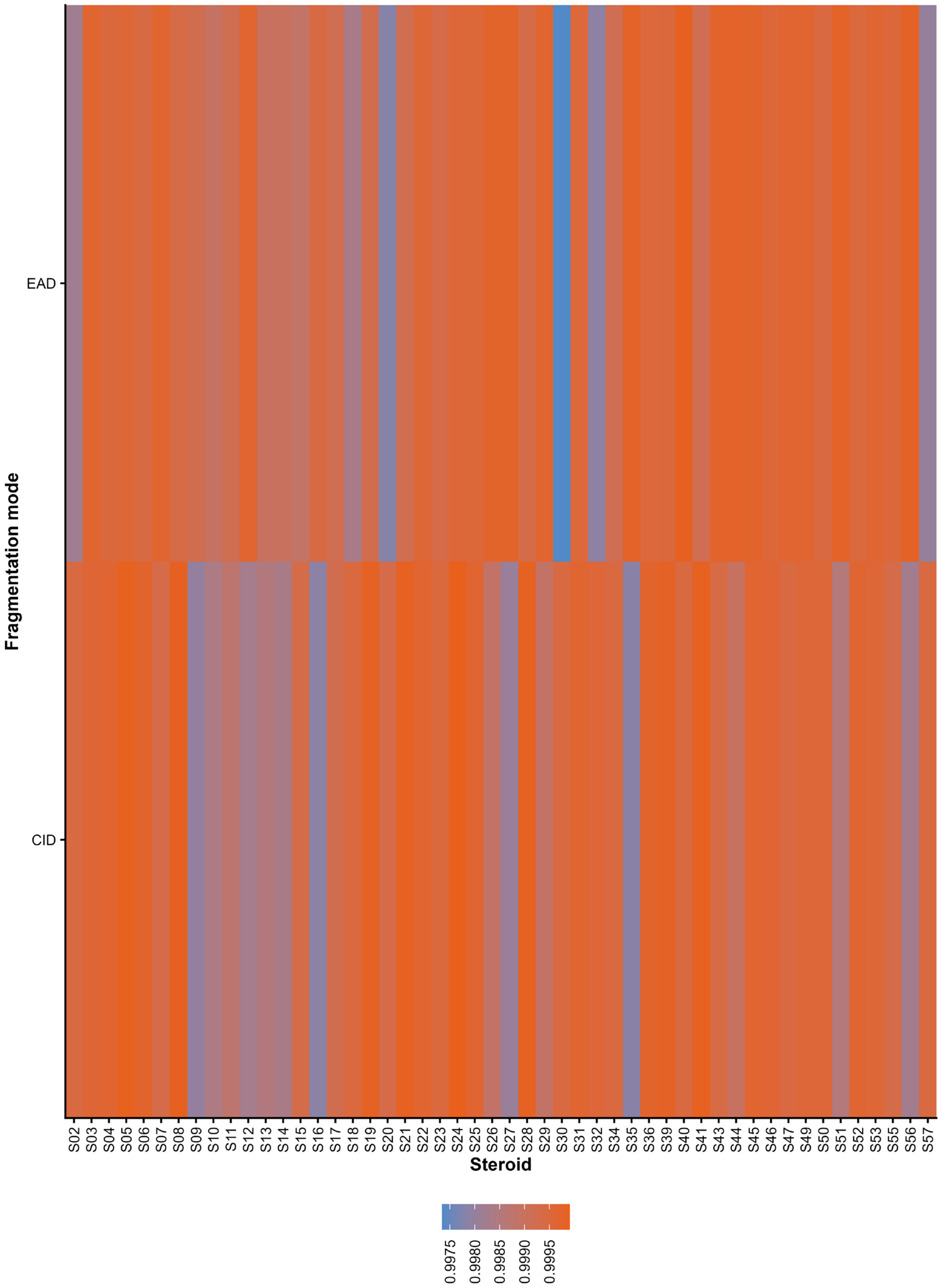

Figure S4: Mean cosine scores per steroid and fragmentation mode (CID/EAD), calculated from cosine scores across triggering events of the same steroid acquired under the same fragmentation settings.

Application of steroid MS/MS libraries

True positive hits were found when matching the acquired EAD MS/MS spectra against existing public spectral libraries. **Figure S5** depicts the distribution of true positive hits (green) across steroids and electron kinetic energies. The fact that true positive matches are not only associated with low electron kinetic energies in the hot electron-capture dissociation region (≤10 eV), but also at fragmentation energies above 10 eV indicates the potential of EAD MS/MS data acquisition for real-life non-targeted metabolomics studies. These MS/MS spectra can be used in two ways – for detailed structural elucidation (characteristic for EAD), and state-of-the-art spectral library matching.

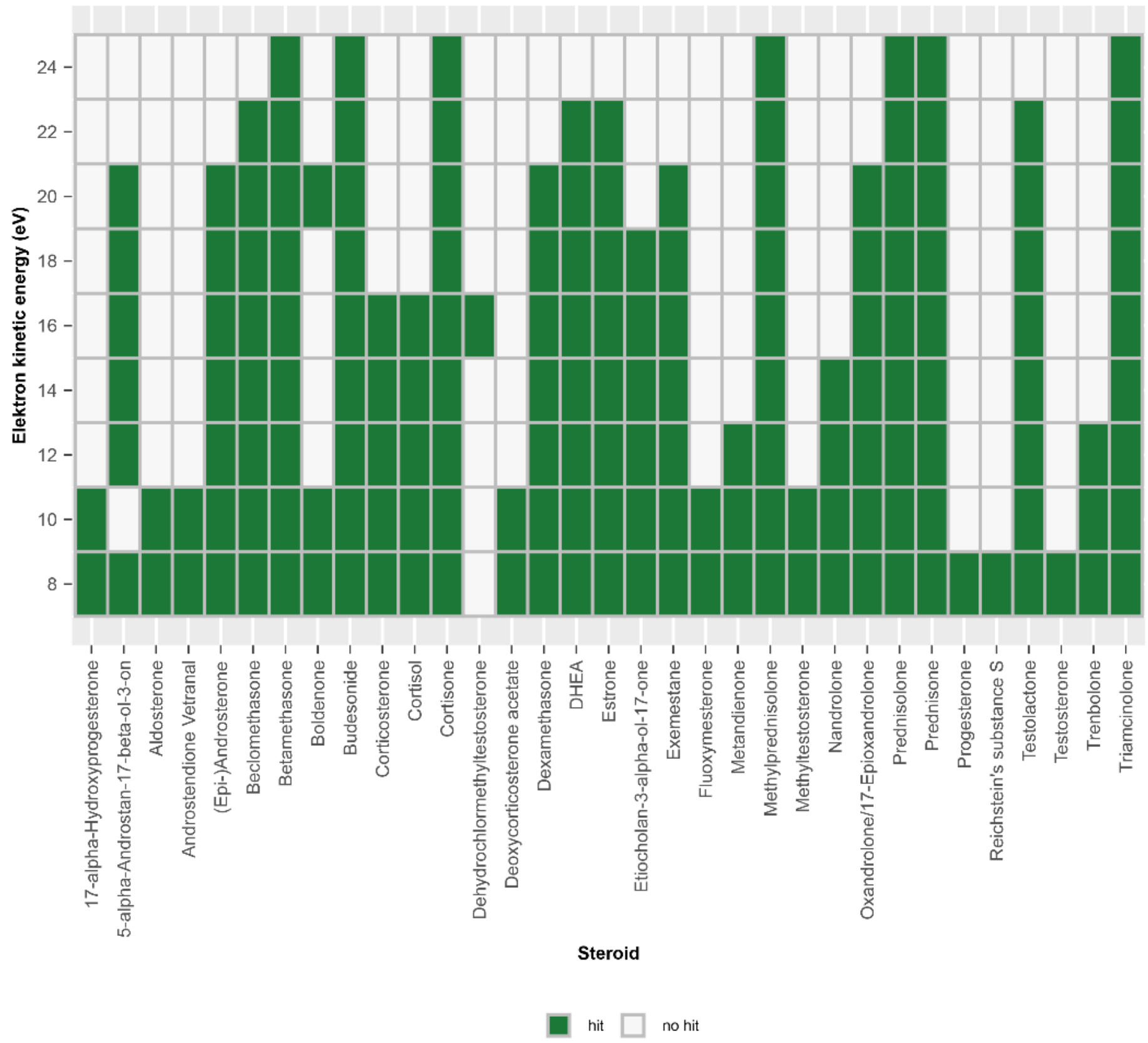

Figure S5: True positive hits (green) of acquired EAD MS/MS spectra matched against existing public libraries per steroid and electron kinetic energy. While the presence of a true positive hit implies the potential of EAD data for spectral library matching, the absence of a true positive hit could also be caused by biases in existing spectral libraries.

The following **Figures** (**S6-S11**) provide exemplary mirror plots of spectral matches of acquired EAD and CID spectra to existing public spectral libraries for Prednisone, Estrone, and Budesonide.

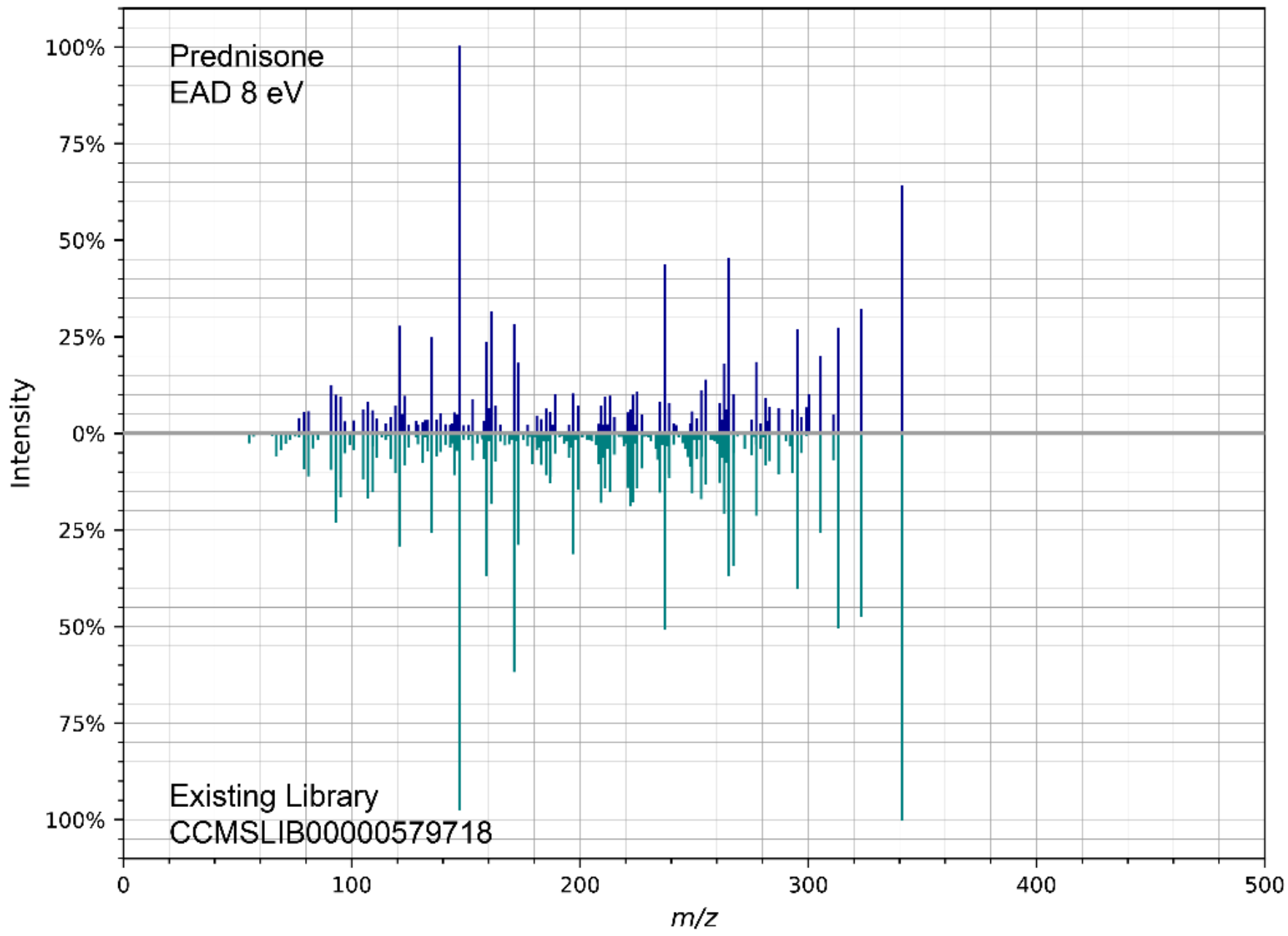

Figure S6: Mirror plot of the MS/MS spectrum of Prednisone acquired at 8 eV in EAD and an existing public library entry. The precursor m/z -17 Da were excluded from the plot.

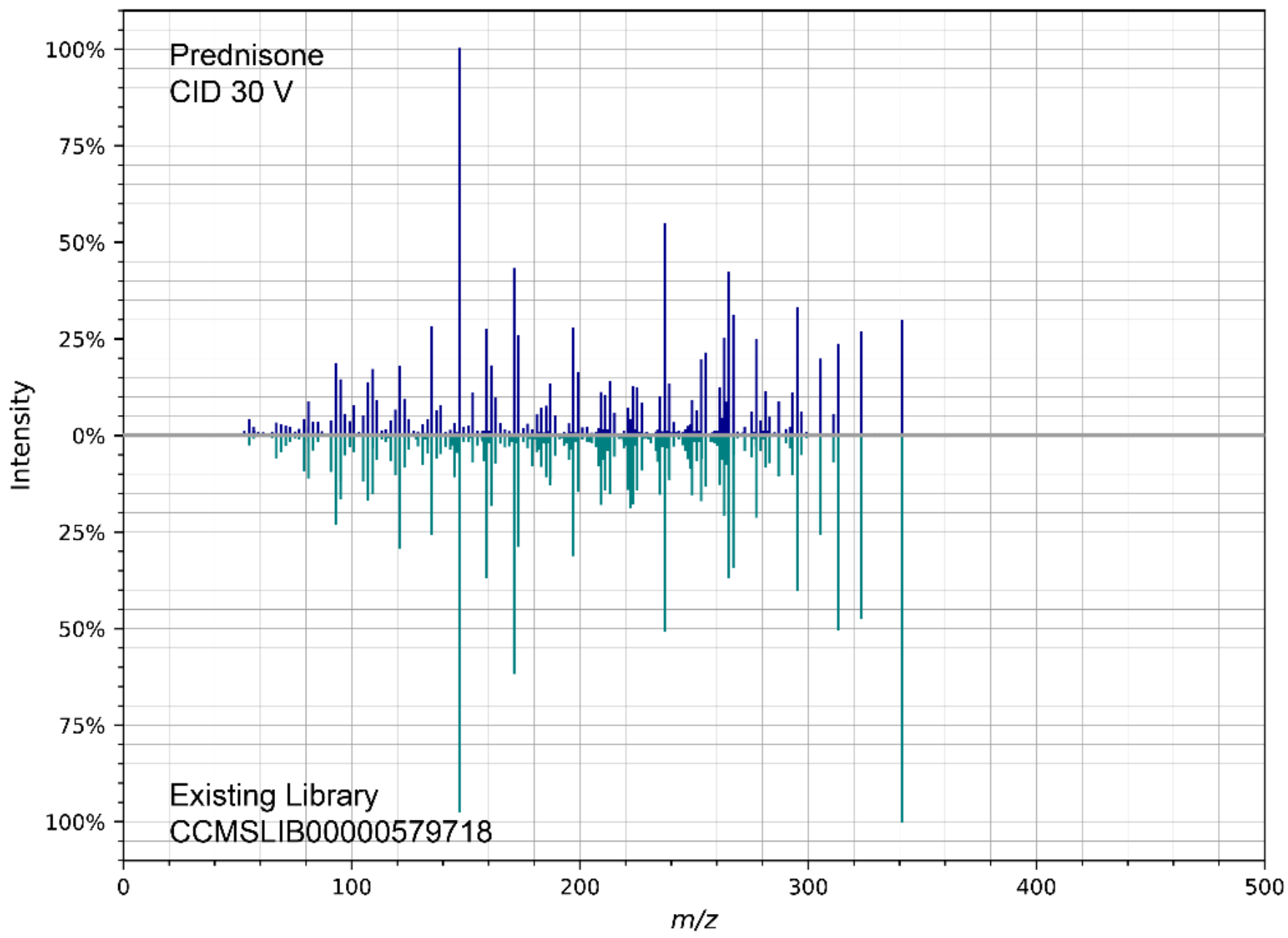

Figure S7: Mirror plot of the MS/MS spectrum of Prednisone acquired at 30 V in CID and an existing public library entry. The precursor m/z -17 Da were excluded from the plot.

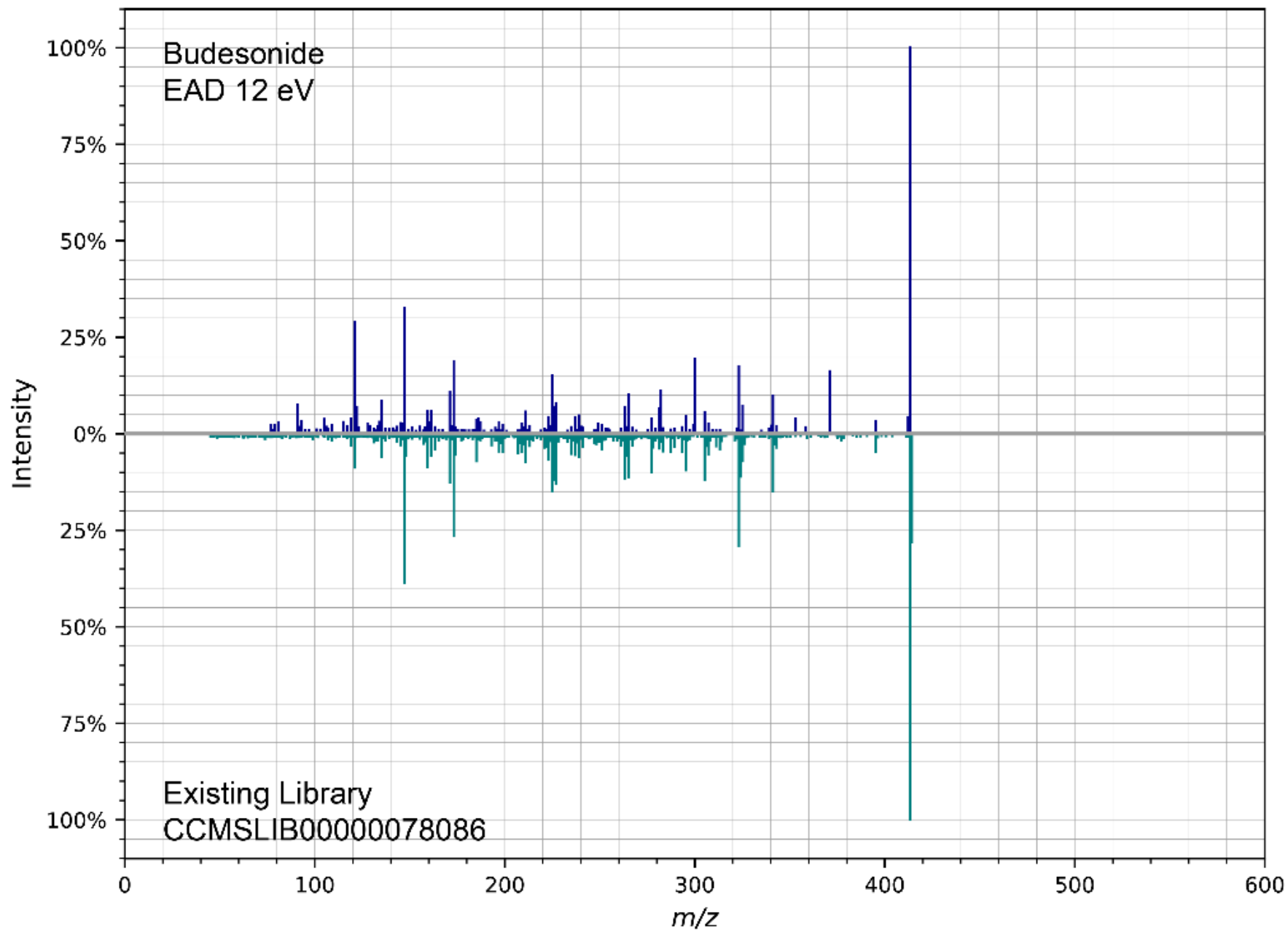

Figure S8: Mirror plot of the MS/MS spectrum of Budesonide acquired at 12 eV in EAD and an existing public library entry. The precursor m/z -17 Da were excluded from the plot.

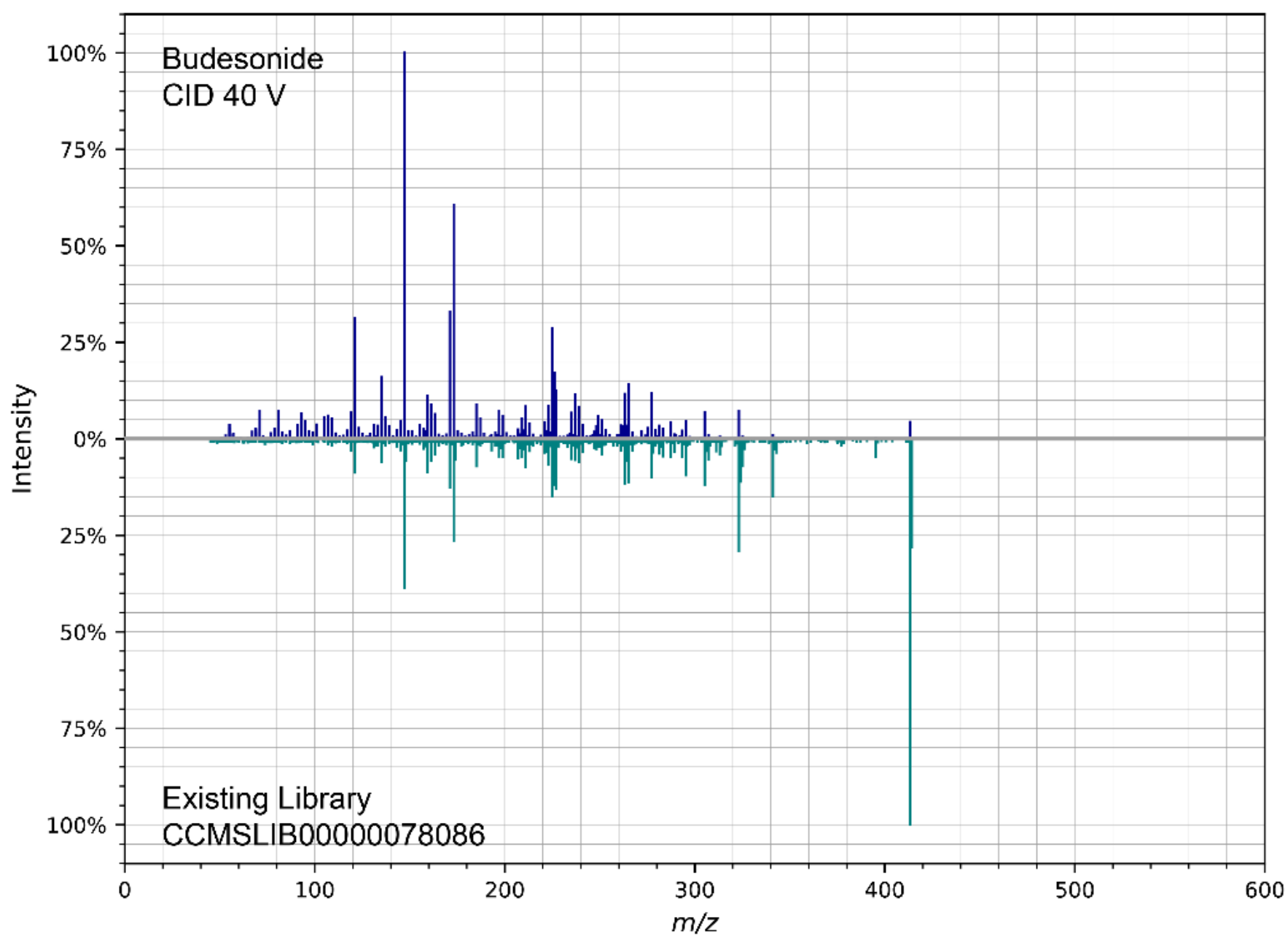

Figure S9: Mirror plot of the MS/MS spectrum of Budesonide acquired at 40 V in CID and an existing public library entry. The precursor m/z -17 Da were excluded from the plot.

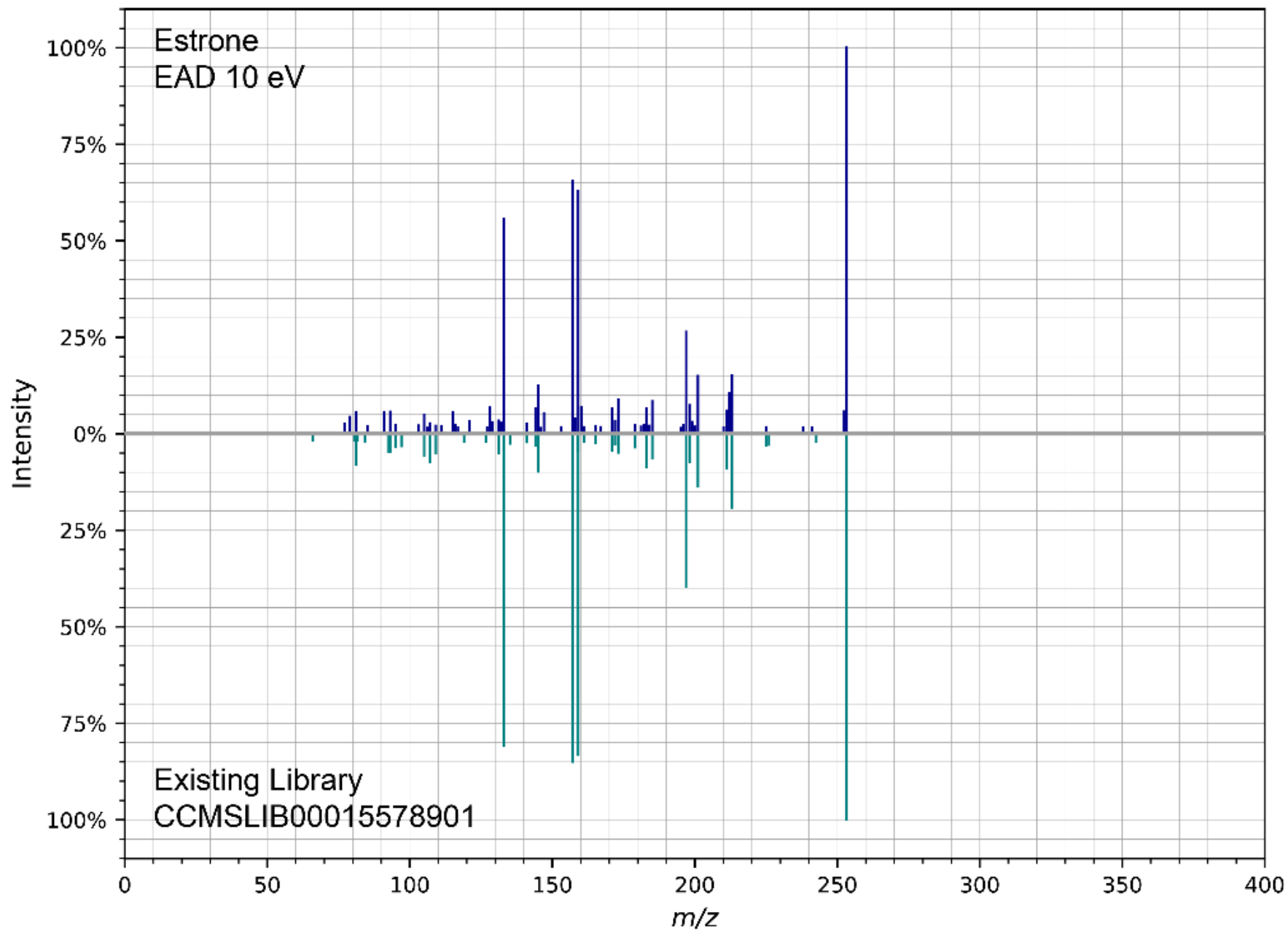

Figure S10: Mirror plot of the MS/MS spectrum of Estrone acquired at 10 eV in EAD and an existing public library entry. The precursor m/z -17 Da were excluded from the plot.

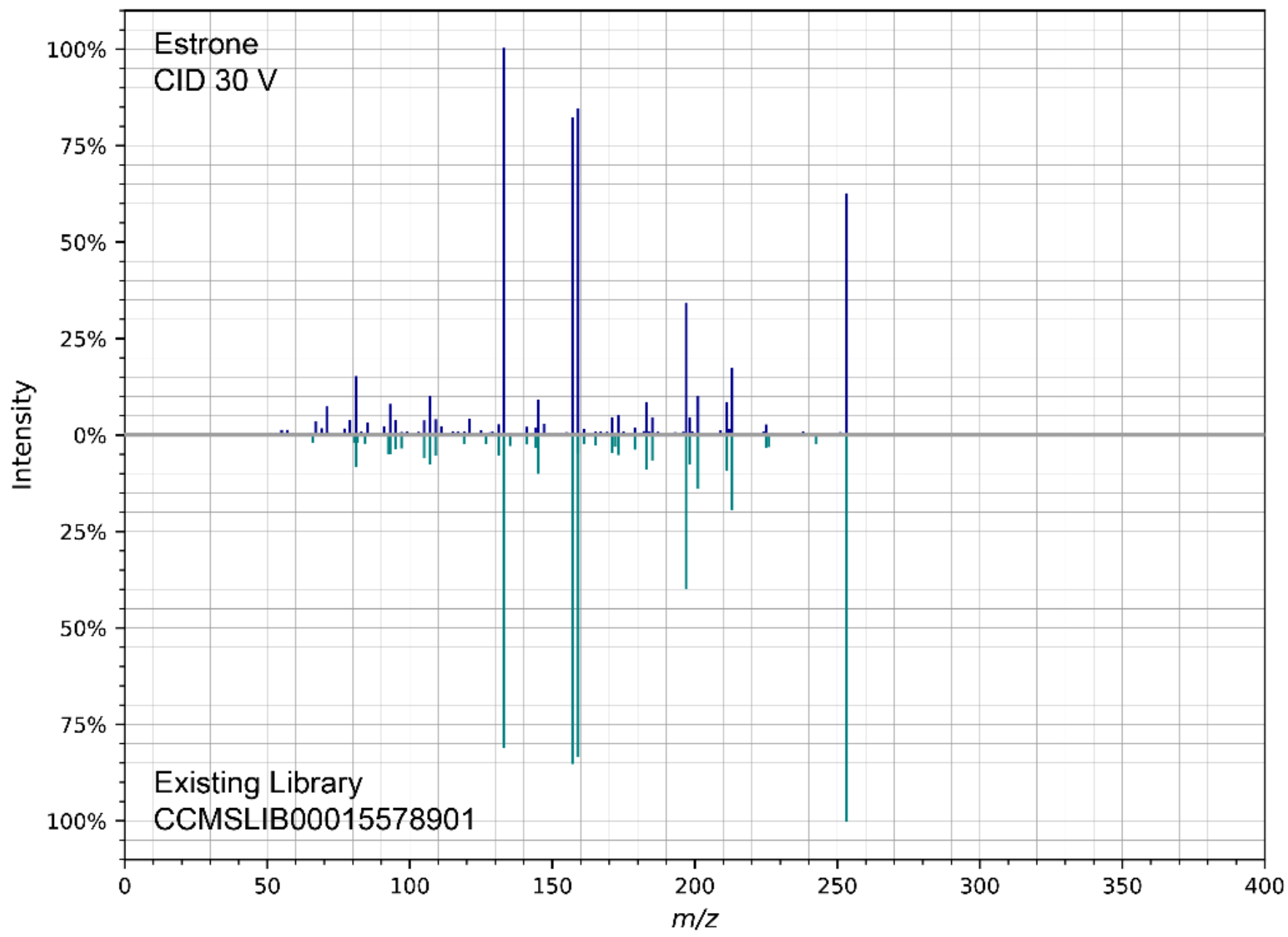

Figure S11: Mirror plot of the MS/MS spectrum of Estrone acquired at 30 V in CID and an existing public library entry. The precursor m/z -17 Da were excluded from the plot.

To assess the transferability of our findings to other compound classes, we additionally performed library matching of publicly available CID and EAD MS/MS spectra of 168 pesticides[7,8]. Of the 168 compounds under investigation, true positive library matches were returned for 68 in CID and 23 in EAD, as illustrated in more detail in **Figure S12**.

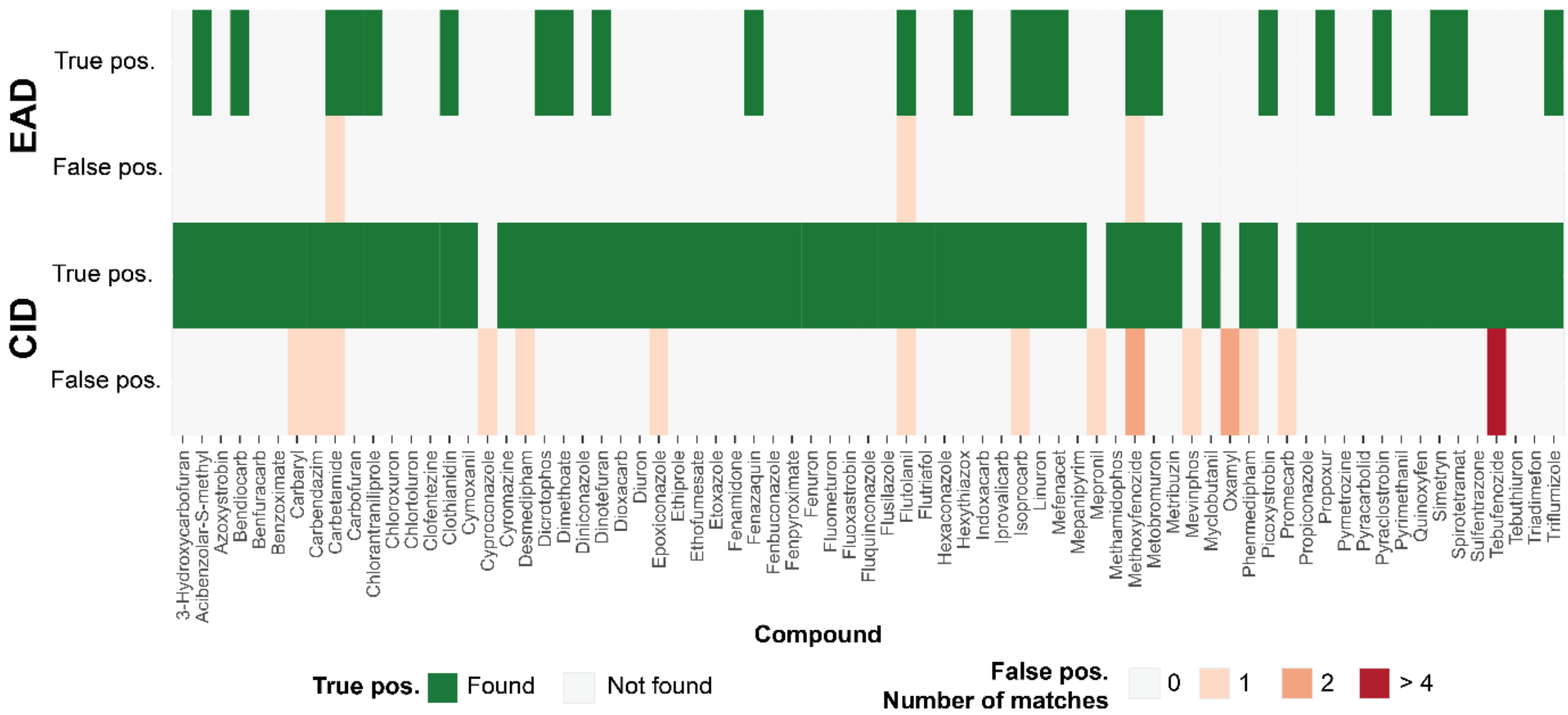

Figure S12: Results of library searches based on a publicly available set of CID and EAD MS/MS spectra of 168 pesticides[7,8]. True positive matches were returned for both CID (68 compounds) and EAD (23 compounds). Only compounds for which matches were returned are displayed.

Given the variety of energies covered by the novel steroid spectral library, we were interested to see which energies were relevant for spectral matches to raw data deposited in public repositories. Spectral matches were filtered as described previously in the **Supplementary Experimental Section**. While for many steroids, a median of 30-40 V can be observed, it can clearly be deduced from **Figure S13** that additional collision energies are required to increase spectral coverage.

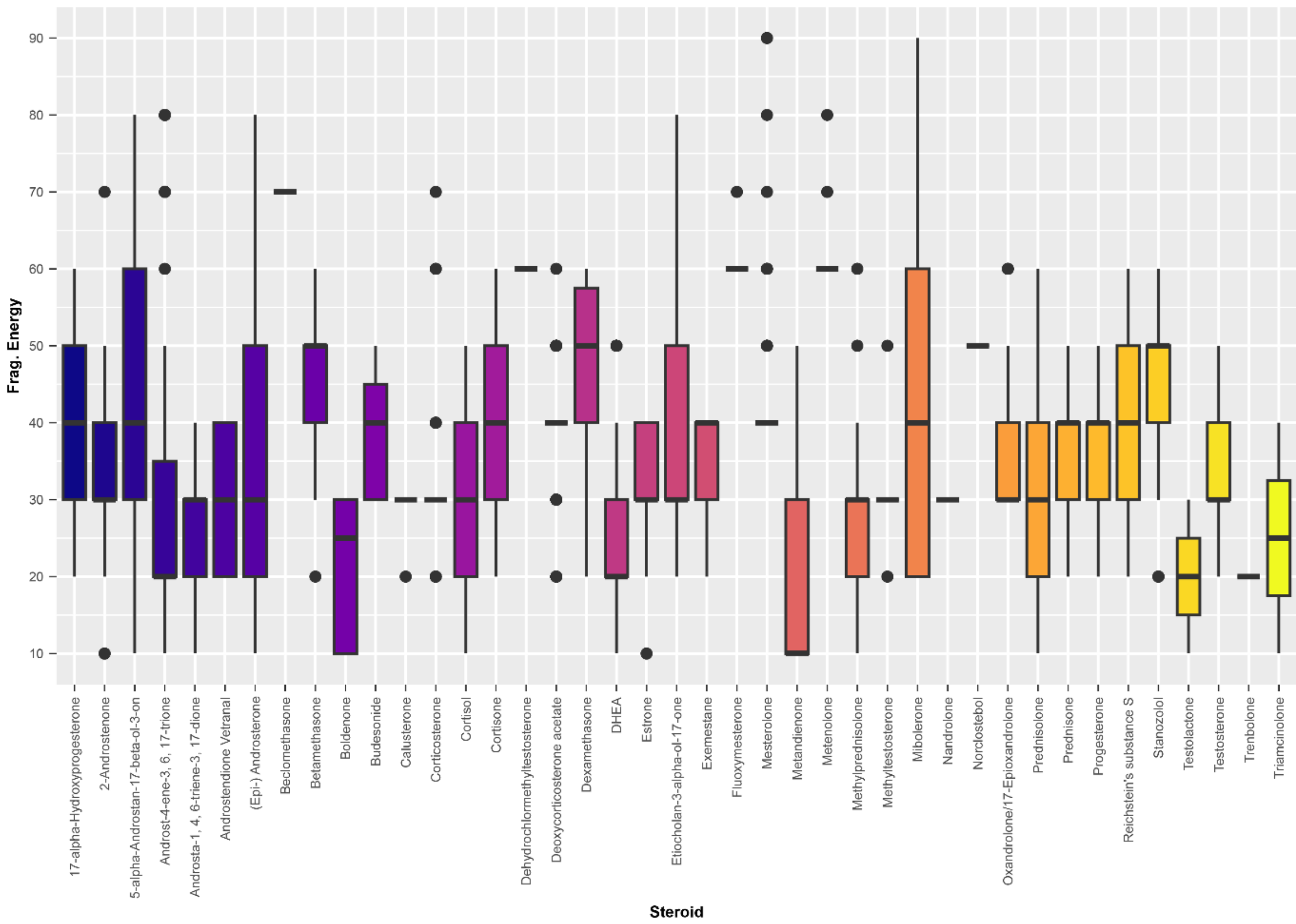

Figure S13: Distribution of CID fragmentation energies returning spectral matches across different steroids. While most annotations are enabled by collision energies in the range of 20 V to 50 V, covering additional fragmentation energies allows for more comprehensive steroid assignments.

Supplementary References

(1) Schmid, R.; Heuckeroth, S.; Korf, A.; Smirnov, A.; Myers, O.; Dyrlund, T. S.; Bushuiev, R.; Murray, K. J.; Hoffmann, N.; Lu, M.; Sarvepalli, A.; Zhang, Z.; Fleischauer, M.; Dührkop, K.; Wesner, M.; Hoogstra, S. J.; Rudt, E.; Mokshyna, O.; Brungs, C.; Ponomarov, K.; Mutabdžija, L.; Damiani, T.; Pudney, C. J.; Earll, M.; Helmer, P. O.; Fallon, T. R.; Schulze, T.; Rivas-Ubach, A.; Bilbao, A.; Richter, H.; Nothias, L. F.; Wang, M.; Orešič, M.; Weng, J. K.; Böcker, S.; Jeibmann, A.; Hayen, H.; Karst, U.; Dorrestein, P. C.; Petras, D.; Du, X.; Pluskal, T. Integrative Analysis of Multimodal Mass Spectrometry Data in MZmine 3. *Nat. Biotechnol.* **2023**, *41* (4), 447–449. https://doi.org/10.1038/s41587-023-01690-2.

(2) Brungs, C.; Schmid, R.; Heuckeroth, S.; Mazumdar, A.; Drexler, M.; Šácha, P.; Dorrestein, P. C.; Petras, D.; Nothias, L.-F.; Veverka, V.; Nencka, R.; Kameník, Z.; Pluskal, T. MSnLib: Efficient Generation of Open Multi-Stage Fragmentation Mass Spectral Libraries. *Nat. Methods* **2025**. https://doi.org/10.1038/s41592-025-02813-0.

(3) Wang, M.; Jarmusch, A. K.; Vargas, F.; Aksenov, A. A.; Gauglitz, J. M.; Weldon, K.; Petras, D.; da Silva, R.; Quinn, R.; Melnik, A. V.; van der Hooft, J. J. J.; Caraballo-Rodríguez, A. M.; Nothias, L. F.; Aceves, C. M.; Panitchpakdi, M.; Brown, E.; Di Ottavio, F.; Sikora, N.; Elijah, E. O.; Labarta-Bajo, L.; Gentry, E. C.; Shalapour, S.; Kyle, K. E.; Puckett, S. P.; Watrous, J. D.; Carpenter, C. S.; Bouslimani, A.; Ernst, M.; Swafford, A. D.; Zúñiga, E. I.; Balunas, M. J.; Klassen, J. L.; Loomba, R.; Knight, R.; Bandeira, N.; Dorrestein, P. C. Mass Spectrometry Searches Using MASST. *Nat. Biotechnol.* **2020**, *38* (1), 22–26. https://doi.org/10.1038/s41587-019-0375-9.

(4) Mongia, M.; Yasaka, T. M.; Liu, Y.; Guler, M.; Lu, L.; Bhagwat, A.; Behsaz, B.; Wang, M.; Dorrestein, P. C.; Mohimani, H. Fast Mass Spectrometry Search and Clustering of Untargeted Metabolomics Data. *Nat. Biotechnol.* **2024**, *42* (11), 1672–1677. https://doi.org/10.1038/s41587-023-01985-4.

(5) RDKit: Open-Source Cheminformatics Version: 2024.03.5. https://www.rdkit.org.

(6) Petras, D.; Phelan, V. V; Acharya, D.; Allen, A. E.; Aron, A. T.; Bandeira, N.; Bowen, B. P.; Belle-Oudry, D.; Boecker, S.; Cummings, D. A.; Deutsch, J. M.; Fahy, E.; Garg, N.; Gregor, R.; Handelsman, J.; Navarro-Hoyos, M.; Jarmusch, A. K.; Jarmusch, S. A.; Louie, K.; Maloney, K. N.; Marty, M. T.; Meijler, M. M.; Mizrahi, I.; Neve, R. L.; Northen, T. R.; Molina-Santiago, C.; Panitchpakdi, M.; Pullman, B.; Puri, A. W.; Schmid, R.; Subramaniam, S.; Thukral, M.; Vasquez-Castro, F.; Dorrestein, P. C.; Wang, M. GNPS Dashboard: Collaborative Exploration of Mass Spectrometry Data in the Web Browser. *Nat. Methods* **2022**, *19* (2), 134–136. https://doi.org/10.1038/s41592-021-01339-5.

(7) Giraud, R.; Le Blanc, J. C. Y.; Guna, M.; Hopfgartner, G. General Screening and Multiple Dissociation Methods for Complementary LC-MS Analysis of Pesticides in Beverages: Potential and Pitfalls. *Anal. Chem.* **2025**, *97* (27), 14503–14511. https://doi.org/10.1021/acs.analchem.5c01831.

(8) Hopfgartner, G.; Giraud, R. *CID-EAD-UVPD MS/MS Spectra of Pesticides*. https://doi.org/10.26037/yareta:idzwd42n4feaddt7fydqefavve (accessed 2026-06-08).
